# Supplementary material for: Identification of Novel Mutations in Colorectal Cancer Patients Using AmpliSeq Comprehensive Cancer Panel
Source: J Pers Med. 2021 Jun 9;11(6):535. doi: 10.3390/jpm11060535 (PMC8230213; doi:10.3390/jpm11060535)
Supplement: Supplementary file 1 [file jpm-11-00535-s001.zip › jpm-1202870-supplementary/supplementary tables/Supplementary Table S1.pdf]

Supplementary Table 1|Ion AmpliSeq comprehensive cancer panel genes list (409) used on this study

ABL1	BLM	CRTC1	EXT1	HIF1A	LAMP1	MSH6	PAX5	RAD50	STK36	WHSC1
ABL2	BLNK	CSF1R	EXT2	HLF	LCK	MTOR	PAX7	RAF1	SUFU	WRN
ACVR2A	BMPR1A	CSMD3	EZH2	HNF1A	LIFR	MTR	PAX8	RALGDS	SYK	WT1
ADAMTS20	BRAF	CTNNA1	FAM123B	HOOK3	LPHN3	MTRR	PBRM1	RARA	SYNE1	XPA
AFF1	BRD3	CTNNB1	FANCA	HRAS	POT1	MUC1	PBX1	RB1	TAF1	XPC
AFF3	BRIP1	CYLD	FANCC	HSP90AA1	LPP	MUTYH	PDE4DIP	RECQL4	TAF1L	XPO1
AKAP9	BTK	CYP2C19	FANCD2	HSP90AB1	LRP1B	MYB	PDGFB	REL	TAL1	XRCC2
AKT1	BUB1B	CYP2D6	FANCF	ICK	LTF	MYC	PDGFRA	RET	TBX22	ZNF384
AKT2	CARD11	DAXX	FANCG	IDH1	LTK	MYCL1	PDGFRB	RHOH	TCF12	ZNF521
AKT3	CASC5	DCC	FAS	IDH2	MAF	MYCN	PER1	RNASEL	TCF3	
ALK	CBL	DDB2	FBXW7	IGF1R	MAFB	MYD88	PGAP3	RNF2	TCF7L1	
APC	CCND1	DDIT3	FGFR1	IGF2	MAGEA1	MYH11	PHOX2B	RNF213	TCF7L2	
AR	CCND2	DDR2	FGFR2	IGF2R	MAGI1	MYH9	PIK3C2B	ROS1	TCL1A	
ARID1A	CCNE1	DEK	FGFR3	IKBKB	MALT1	NBN	PIK3CA	RPS6KA2	TET1	
ARID2	CD79A	DICER1	FGFR4	IKBKE	MAML2	NCOA1	PIK3CB	RRM1	TET2	
ARNT	CD79B	DNMT3A	FH	IKZF1	MAP2K1	NCOA2	PIK3CD	RUNX1	TFE3	
ASXL1	CDC73	DPYD	FLCN	IL2	MAP2K2	NCOA4	PIK3CG	RUNX1T1	TGFBR2	
ATF1	CDH1	DST	FLI1	IL21R	MAP2K4	NF1	PIK3R1	SAMD9	TGM7	
ATM	CDH11	EGFR	FLT1	IL6ST	MAP3K7	NF2	PIK3R2	SBDS	THBS1	
ATR	CDH2	EML4	FLT3	IL7R	MAPK1	NFE2L2	PIM1	SDHA	TIMP3	
ATRX	CDH20	EP300	FLT4	ING4	MAPK8	NFKB1	PKHD1	SDHB	TLR4	
AURKA	CDH5	EP400	FN1	IRF4	MARK1	NFKB2	PLAG1	SDHC	TLX1	
AURKB	CDK12	EPHA3	FOXL2	IRS2	MARK4	NIN	PLCG1	SDHD	TNFAIP3	
AURKC	CDK4	EPHA7	FOXO1	ITGA10	MBD1	NKX2-1	PLEKHG5	SEPT9	TNFRSF14	
AXL	CDK6	EPHB1	FOXO3	ITGA9	MCL1	NLRP1	PML	SETD2	TNK2	
BAI3	CDK8	EPHB4	FOXP1	ITGB2	MDM2	NOTCH1	PMS1	SF3B1	TOP1	
BAP1	CDKN2A	EPHB6	FOXP4	ITGB3	MDM4	NOTCH2	PMS2	SGK1	TP53	
BCL10	CDKN2B	ERBB2	FZR1	JAK1	MEN1	NOTCH4	POU5F1	SH2D1A	TPR	
BCL11A	CDKN2C	ERBB3	G6PD	JAK2	MET	NPM1	PPARG	SMAD2	TRIM24	
BCL11B	CEBPA	ERBB4	GATA1	JAK3	MITF	NRAS	PPP2R1A	SMAD4	TRIM33	
BCL2	CHEK1	ERCC1	GATA2	JUN	MLH1	NSD1	PRDM1	SMARCA4	TRIP11	
BCL2L1	CHEK2	ERCC2	GATA3	KAT6A	MLL	NTRK1	PRKAR1A	SMARCB1	TRRAP	
BCL2L2	CIC	ERCC3	GDNF	KAT6B	MLL2	NTRK3	PRKDC	SMO	TSC1	
BCL3	CKS1B	ERCC4	GNA11	KDM5C	MLL3	NUMA1	PSIP1	SMUG1	TSC2	
BCL6	CMPK1	ERCC5	GNAQ	KDM6A	MLLT10	NUP214	PTCH1	SOCS1	TSHR	
BCL9	COL1A1	ERG	GNAS	KDR	MMP2	NUP98	PTEN	SOX11	UBR5	
BCR	CRBN	ESR1	GPR124	KEAP1	MN1	PAK3	PTGS2	SOX2	UGT1A1	
BIRC2	CREB1	ETS1	GRM8	KIT	MPL	PALB2	PTPN11	SRC	USP9X	
BIRC3	CREBBP	ETV1	GUCY1A2	KLF6	MRE11A	PARP1	PTPRD	SSX1	VHL	
BIRC5	CRKL	ETV4	HCAR1	KRAS	MSH2	PAX3	PTPRT	STK11	WAS	
