# Supplementary material for: Identification of Novel Mutations in Colorectal Cancer Patients Using AmpliSeq Comprehensive Cancer Panel
Source: J Pers Med. 2021 Jun 9;11(6):535. doi: 10.3390/jpm11060535 (PMC8230213; doi:10.3390/jpm11060535)
Supplement: Supplementary file 1 [file jpm-11-00535-s001.zip › jpm-1202870-supplementary/supplementary tables/Supplementary Table S2.pdf]

Supplementary Table S2

sn	CHR	SNP	Gene ID_Cl	A1	A2	MAF	A1A1	A1A2
665	2	chr2:148672817	ACVR2A	T	G	0.005556	0	1
2899	12	chr12:43846296	ADAMTS2C	T	G	0.005556	0	1
1154	4	chr4:87967279	AFF1	C	A	0.005556	0	1
1155	4	chr4:87967936	AFF1	T	C	0.005556	0	1
1912	7	chr7:91631234	AKAP9	C	A	0.005556	0	1
1915	7	chr7:91641671	AKAP9	TGC	CAA	0.005556	0	1
1919	7	chr7:91670064	AKAP9	C	T	0.005556	0	1
1921	7	chr7:91672096	AKAP9	G	T	0.005556	0	1
1923	7	chr7:91699372	AKAP9	A	-	0.005556	0	1
1927	7	chr7:91709156	AKAP9	G	T	0.005556	0	1
1929	7	chr7:91712728	AKAP9	A	C	0.005556	0	1
3951	19	chr19:40741244	AKT2	A	G	0.005556	0	1
486	1	chr1:243727211	AKT3	-	T	0.005556	0	1
520	2	chr2:29498305	ALK	-	A	0.005556	0	1
1299	5	chr5:112164622	APC	A	G	0.005556	0	1
1300	5	chr5:112164623	APC	-	T	0.005556	0	1
1303	5	chr5:112173971	APC	TA	GT	0.005556	0	1
1307	5	chr5:112175208	APC	-	A	0.005556	0	1
1316	5	chr5:112175611	APC	-	ACCACCTC(	0.005556	0	1
4226	23	chrX:66943601	AR	-	A	0.088	4	0
62	1	chr1:27092820	ARID1A	-	A	0.005556	0	1
63	1	chr1:27092822	ARID1A	T	A	0.005556	0	1
64	1	chr1:27097699	ARID1A	A	T	0.02222	1	2
65	1	chr1:27099010	ARID1A	-	G	0.01111	1	0
70	1	chr1:27106343	ARID1A	T	C	0.005556	0	1
2915	12	chr12:46285667	ARID2	C	A	0.005556	0	1
312	1	chr1:150808978	ARNT	A	G	0.005556	0	1
2814	11	chr11:108117897	ATM	TTA	ATT	0.005556	0	1
2833	11	chr11:108175596	ATM	G	T	0.005556	0	1
2835	11	chr11:108201010	ATM	C	T	0.005556	0	1
979	3	chr3:142176618	ATR	-	T	0.005556	0	1
980	3	chr3:142176649	ATR	A	C	0.005556	0	1
982	3	chr3:142186845	ATR	-	T	0.005556	0	1
991	3	chr3:142269112	ATR	G	C	0.005556	0	1
994	3	chr3:142272647	ATR	A	T	0.01111	0	2
995	3	chr3:142274652	ATR	A	C	0.005556	0	1
4236	23	chrX:76849165	ATRX	T	C	0.008	0	1
4046	20	chr20:54959349	AURKA	T	-	0.005556	0	1
3586	17	chr17:8109871	AURKB	C	T	0.005556	0	1
3999	19	chr19:57744792	AURKC	T	C	0.005556	0	1
1601	6	chr6:69349192	BAI3	A	G	0.005556	0	1
1615	6	chr6:70071243	BAI3	G	A	0.005556	0	1
111	1	chr1:85733370	BCL10	C	A	0.005556	0	1
113	1	chr1:85736596	BCL10	T	G	0.005556	0	1
558	2	chr2:60773062	BCL11A	G	A	0.005556	0	1

1030	3	**chr3:187447019**	BCL6	A	G	0.005556	0	1
293	1	**chr1:147090697**	BCL9	T	C	0.005556	0	1
298	1	**chr1:147094034**	BCL9	T	A	0.005556	0	1
2793	11	**chr11:102195293**	BIRC3	C	A	0.005556	0	1
2795	11	**chr11:102195716**	BIRC3	T	G	0.005556	0	1
2796	11	**chr11:102201890**	BIRC3	C	T	0.005556	0	1
2797	11	**chr11:102207645**	BIRC3	A	C	0.01111	0	2
2798	11	**chr11:102207653**	BIRC3	C	A	0.005556	0	1
3719	17	**chr17:76210389**	BIRC5	A	G	0.005556	0	1
3722	17	**chr17:76219645**	BIRC5	T	C	0.02778	0	5
3291	15	**chr15:91304145**	BLM	T	A	0.01111	0	2
3299	15	**chr15:91347493**	BLM	A	C	0.005556	0	1
2657	10	**chr10:97975168**	BLNK	-	TA	0.03333	0	6
3691	17	**chr17:59760648**	BRIP1	C	T	0.005556	0	1
3697	17	**chr17:59858190**	BRIP1	T	G	0.005556	0	1
3698	17	**chr17:59861596**	BRIP1	A	T	0.005556	0	1
1855	7	**chr7:2968269**	CARD11	C	T	0.005556	0	1
3230	15	**chr15:40913884**	CASC5	T	G	0.005556	0	1
3244	15	**chr15:40949531**	CASC5	-	TTTT	0.005556	0	1
2849	11	**chr11:119145464**	CBL	-	TT	0.005556	0	1
391	1	**chr1:193110968**	CDC73	G	A	0.005556	0	1
393	1	**chr1:193111251**	CDC73	A	G	0.005556	0	1
3462	16	**chr16:68844081**	CDH1	T	C	0.005556	0	1
3448	16	**chr16:65026991**	CDH11	G	T	0.005556	0	1
3801	18	**chr18:25589869**	CDH2	A	G	0.005556	0	1
3804	18	**chr18:25593716**	CDH2	A	C	0.005556	0	1
3857	18	**chr18:59221876**	CDH20	A	G	0.02778	0	5
3617	17	**chr17:37627782**	CDK12	G	T	0.005556	0	1
2960	12	**chr12:58143112**	CDK4	-	AGG	0.005556	0	1
2403	9	**chr9:21994158**	CDKN2A	C	T	0.005556	0	1
3961	19	**chr19:42791062**	CIC	T	C	0.005556	0	1
3965	19	**chr19:42798455**	CIC	G	A	0.005556	0	1
3689	17	**chr17:48277061**	COL1A1	-	CCCCAGGC	0.01667	1	1
763	3	**chr3:3194271**	CRBN	C	A	0.005556	0	1
766	3	**chr3:3197988**	CRBN	G	C	0.005556	0	1
3360	16	**chr16:3843610**	CREBBP	G	T	0.005556	0	1
2291	8	**chr8:113254080**	CSMD3	-	CACACACG	0.01111	0	2
2296	8	**chr8:113301795**	CSMD3	TTA	ATT	0.005556	0	1
2302	8	**chr8:113323397**	CSMD3	G	T	0.005556	0	1
2311	8	**chr8:113516216**	CSMD3	G	A	0.01667	0	3
2313	8	**chr8:113568949**	CSMD3	G	T	0.005556	0	1
1340	5	**chr5:138266523**	CTNNA1	G	A	0.005556	0	1
1343	5	**chr5:138269717**	CTNNA1	-	AGA	0.005556	0	1
853	3	**chr3:41267196**	CTNNB1	A	C	0.005556	0	1
2651	10	**chr10:96541627**	CYP2C19	G	A	0.005556	0	1
4176	22	**chr22:42524893**	CYP2D6	T	C	0.005556	0	1
3830	18	**chr18:50451642**	DCC	G	T	0.005556	0	1

3831	18	**chr18:50451662**	DCC	C	G	0.005556	0	1
3844	18	**chr18:50942526**	DCC	T	G	0.005556	0	1
3845	18	**chr18:50976845**	DCC	C	A	0.005556	0	1
341	1	**chr1:162737153**	DDR2	T	A	0.01111	0	2
343	1	**chr1:162740326**	DDR2	CG	GT	0.01111	0	2
349	1	**chr1:162748385**	DDR2	T	G	0.005556	0	1
3153	14	**chr14:95569893**	DICER1	A	G	0.005556	0	1
499	2	**chr2:25457148**	DNMT3A	G	T	0.005556	0	1
506	2	**chr2:25523013**	DNMT3A	A	G	0.005556	0	1
119	1	**chr1:97564040**	DPYD	G	T	0.005556	0	1
128	1	**chr1:98144767**	DPYD	T	A	0.005556	0	1
133	1	**chr1:98206046**	DPYD	C	A	0.005556	0	1
135	1	**chr1:98386487**	DPYD	A	C	0.005556	0	1
1548	6	**chr6:56341107**	DST	A	T	0.08889	0	16
1549	6	**chr6:56347533**	DST	C	A	0.005556	0	1
1550	6	**chr6:56350158**	DST	C	A	0.005556	0	1
1562	6	**chr6:56417148**	DST	G	T	0.005556	0	1
1566	6	**chr6:56417448**	DST	C	A	0.005556	0	1
1588	6	**chr6:56485091**	DST	-	AAA	0.01111	1	0
1590	6	**chr6:56485266**	DST	C	G	0.005556	0	1
1593	6	**chr6:56489467**	DST	-	TTACTC	0.005556	0	1
4153	22	**chr22:41542838**	EP300	-	T	0.03333	3	0
4157	22	**chr22:41550944**	EP300	A	T	0.005556	0	1
2992	12	**chr12:132466141**	EP400	A	G	0.005556	0	1
2996	12	**chr12:132490800**	EP400	A	G	0.005556	0	1
2997	12	**chr12:132497625**	EP400	T	C	0.005556	0	1
3004	12	**chr12:132529321**	EP400	C	A	0.005556	0	1
3008	12	**chr12:132535176**	EP400	G	A	0.005556	0	1
3014	12	**chr12:132562069**	EP400	C	A	0.01111	1	0
942	3	**chr3:89448462**	EPHA3	C	T	0.005556	0	1
944	3	**chr3:89462276**	EPHA3	C	T	0.005556	0	1
945	3	**chr3:89462294**	EPHA3	C	A	0.005556	0	1
1626	6	**chr6:93968004**	EPHA7	C	T	0.05556	3	4
1627	6	**chr6:93973530**	EPHA7	G	T	0.005556	0	1
1634	6	**chr6:94128960**	EPHA7	C	T	0.005556	0	1
964	3	**chr3:134885838**	EPHB1	T	C	0.005556	0	1
3627	17	**chr17:37868327**	ERBB2	A	G	0.005556	0	1
3629	17	**chr17:37868741**	ERBB2	C	G	0.005556	0	1
3636	17	**chr17:37883680**	ERBB2	T	C	0.005556	0	1
2948	12	**chr12:56479046**	ERBB3	T	C	0.005556	0	1
700	2	**chr2:212248634**	ERBB4	T	G	0.005556	0	1
704	2	**chr2:212495163**	ERBB4	T	G	0.005556	0	1
709	2	**chr2:212989451**	ERBB4	T	G	0.005556	0	1
4068	21	**chr21:39817470**	ERG	C	T	0.01111	1	0
1878	7	**chr7:13950941**	ETV1	T	G	0.005556	0	1
3645	17	**chr17:41610590**	ETV4	G	C	0.005556	0	1
2329	8	**chr8:118825146**	EXT1	A	T	0.005556	0	1

2333	8	**chr8:119122431**	EXT1	A	G	0.005556	0	1
2746	11	**chr11:44228576**	EXT2	-	A	0.005556	0	1
2088	7	**chr7:148523504**	EZH2	A	T	0.01667	0	3
4218	23	**chrX:63411640**	FAM123B	ATAGAACT	-	0.008	0	1
3505	16	**chr16:89857743**	FANCA	C	G	0.005556	0	1
3516	16	**chr16:89877320**	FANCA	A	C	0.005556	0	1
780	3	**chr3:10088295**	FANCD2	G	A	0.005556	0	1
791	3	**chr3:10132031**	FANCD2	-	A	0.01111	0	2
3895	19	**chr19:8146019**	FBN3	G	T	0.01111	1	0
1196	4	**chr4:153244077**	FBXW7	A	C	0.005556	0	1
2165	8	**chr8:38272357**	FGFR1	A	T	0.04444	0	8
2688	10	**chr10:123246886**	FGFR2	C	T	0.005556	0	1
1065	4	**chr4:1803326**	FGFR3	-	C	0.005556	0	1
1073	4	**chr4:1807545**	FGFR3	-	C	0.01111	0	2
1377	5	**chr5:176520463**	FGFR4	A	G	0.005556	0	1
3021	13	**chr13:28597449**	FLT3	-	CT	0.02222	2	0
1403	5	**chr5:180035995**	FLT4	G	C	0.005556	0	1
712	2	**chr2:216232553**	FN1	G	A	0.005556	0	1
717	2	**chr2:216237084**	FN1	C	G	0.005556	0	1
737	2	**chr2:216272993**	FN1	A	C	0.005556	0	1
3051	13	**chr13:41134840**	FOXO1	C	A	0.005556	0	1
1642	6	**chr6:108985258**	FOXO3	G	A	0.005556	0	1
936	3	**chr3:71179709**	FOXP1	C	-	0.005556	0	1
1471	6	**chr6:41559050**	FOXP4	T	C	0.005556	0	1
3885	19	**chr19:3531825**	FZR1	G	C	0.005556	0	1
955	3	**chr3:128202760**	GATA2	C	G	0.03889	1	5
4052	20	**chr20:57428437**	GNAS	G	C	0.1222	0	22
2162	8	**chr8:37691418**	GPR124	A	C	0.005556	0	1
489	2	**chr2:11751005**	GREB1	T	G	0.01111	1	0
2803	11	**chr11:106579288**	GUCY1A2	A	G	0.005556	0	1
2189	8	**chr8:42868456**	HOOK3	-	TTTTTT	0.005556	0	1
3167	14	**chr14:102548088**	HSP90AA1	G	T	0.005556	0	1
3170	14	**chr14:102548797**	HSP90AA1	C	G	0.005556	0	1
3286	15	**chr15:90628243**	IDH2	A	C	0.005556	0	1
3305	15	**chr15:99250742**	IGF1R	C	A	0.005556	0	1
3313	15	**chr15:99460151**	IGF1R	T	G	0.005556	0	1
1809	6	**chr6:160412166**	IGF2R	T	A	0.01111	0	2
1819	6	**chr6:160491086**	IGF2R	G	A	0.005556	0	1
2184	8	**chr8:42175297**	IKBKB	C	T	0.005556	0	1
426	1	**chr1:206647652**	IKBKE	T	A	0.005556	0	1
1266	5	**chr5:55237092**	IL6ST	C	A	0.005556	0	1
1267	5	**chr5:55237596**	IL6ST	A	C	0.005556	0	1
1269	5	**chr5:55247262**	IL6ST	-	GAAA	0.005556	0	1
1273	5	**chr5:55256341**	IL6ST	T	C	0.005556	0	1
1277	5	**chr5:55264283**	IL6ST	G	T	0.005556	0	1
1422	6	**chr6:397155**	IRF4	T	C	0.005556	0	1
3073	13	**chr13:110434840**	IRS2	T	C	0.005556	0	1

279	1	chr1:145528319	ITGA10	T	G	0.005556	0	1
280	1	chr1:145528348	ITGA10	A	G	0.005556	0	1
282	1	chr1:145530939	ITGA10	T	C	0.02222	0	4
286	1	chr1:145533894	ITGA10	-	C	0.01667	0	3
838	3	chr3:37567515	ITGA9	C	A	0.005556	0	1
848	3	chr3:37791907	ITGA9	A	C	0.005556	0	1
4083	21	chr21:46327067	ITGB2	A	-	0.02222	2	0
3651	17	chr17:45360865	ITGB3	A	C	0.005556	0	1
3652	17	chr17:45361773	ITGB3	T	G	0.005556	0	1
3662	17	chr17:45377857	ITGB3	C	T	0.005556	0	1
3663	17	chr17:45377881	ITGB3	A	C	0.005556	0	1
2368	9	chr9:5081778	JAK2	TA	CT	0.01111	1	0
2370	9	chr9:5090443	JAK2	G	A	0.005556	0	1
2171	8	chr8:41791041	KAT6A	A	-	0.01111	1	0
2615	10	chr10:76735416	KAT6B	-	T	0.06667	1	10
2617	10	chr10:76736004	KAT6B	C	T	0.005556	0	1
2618	10	chr10:76744954	KAT6B	-	A	0.005556	0	1
2619	10	chr10:76744958	KAT6B	T	A	0.005556	0	1
2620	10	chr10:76744965	KAT6B	G	A	0.01111	0	2
4214	23	chrX:53245059	KDM5C	T	C	0	0	0
4205	23	chrX:44942869	KDM6A	C	T	0.008	0	1
1122	4	chr4:55953747	KDR	-	CG	0.03333	3	0
1126	4	chr4:55970908	KDR	G	T	0.005556	0	1
1130	4	chr4:55980293	KDR	A	C	0.005556	0	1
1131	4	chr4:55981509	KDR	T	-	0.005556	0	1
3901	19	chr19:10600522	KEAP1	A	G	0.005556	0	1
2561	10	chr10:3824270	KLF6	C	T	0.005556	0	1
2879	12	chr12:25398279	KRAS	CGC	-	0.005556	0	1
1249	5	chr5:38481879	LIFR	G	T	0.005556	0	1
1250	5	chr5:38482324	LIFR	T	G	0.005556	0	1
1252	5	chr5:38486091	LIFR	T	A	0.005556	0	1
1256	5	chr5:38499715	LIFR	A	G	0.005556	0	1
1258	5	chr5:38502822	LIFR	C	G	0.005556	0	1
1137	4	chr4:62599099	LPHN3	G	A	0.005556	0	1
1150	4	chr4:62897317	LPHN3	A	C	0.005556	0	1
597	2	chr2:141079482	LRP1B	G	T	0.005556	0	1
606	2	chr2:141202026	LRP1B	CA	AT	0.07778	7	0
607	2	chr2:141214069	LRP1B	G	C	0.005556	0	1
609	2	chr2:141232924	LRP1B	C	T	0.005556	0	1
624	2	chr2:141299350	LRP1B	T	C	0.005556	0	1
633	2	chr2:141598592	LRP1B	C	A	0.005556	0	1
637	2	chr2:141607906	LRP1B	T	A	0.005556	0	1
640	2	chr2:141680645	LRP1B	C	A	0.005556	0	1
652	2	chr2:141819743	LRP1B	T	C	0.005556	0	1
655	2	chr2:141986696	LRP1B	A	G	0.005556	0	1
656	2	chr2:142004935	LRP1B	C	A	0.005556	0	1
661	2	chr2:142888193	LRP1B	TA	GG	0.005556	0	1

881	3	chr3:46501310	LTF	A	C	0.005556	0	1
920	3	chr3:65376926	MAGI1	T	C	0.005556	0	1
2785	11	chr11:95724845	MAML2	C	T	0.005556	0	1
2790	11	chr11:95826226	MAML2	A	G	0.005556	0	1
2791	11	chr11:95826390	MAML2	A	G	0.005556	0	1
3270	15	chr15:66782073	MAP2K1	T	C	0.005556	0	1
441	1	chr1:220808683	MARK1	GC	TT	0.01111	0	2
3976	19	chr19:45805895	MARK4	A	T	0.005556	0	1
3807	18	chr18:47793930	MBD1	G	A	0.03333	0	6
3813	18	chr18:47801260	MBD1	C	A	0.01111	1	0
300	1	chr1:150550722	MCL1	G	A	0.005556	0	1
2755	11	chr11:64572073	MEN1	A	G	0.005556	0	1
2021	7	chr7:116339867	MET	G	T	0.005556	0	1
2025	7	chr7:116397717	MET	G	C	0.06111	0	11
929	3	chr3:69985829	MITF	C	T	0.005556	0	1
932	3	chr3:70000910	MITF	G	T	0.005556	0	1
833	3	chr3:37090047	MLH1	A	T	0.005556	0	1
2844	11	chr11:118376178	MLL	T	G	0.005556	0	1
2846	11	chr11:118390491	MLL	A	G	0.005556	0	1
2931	12	chr12:49433170	MLL2	C	-	0.01111	1	0
2096	7	chr7:151859655	MLL3	A	G	0.005556	0	1
2107	7	chr7:151879244	MLL3	-	T	0.04444	4	0
2112	7	chr7:151902163	MLL3	T	A	0.01111	1	0
2113	7	chr7:151902164	MLL3	T	A	0.005556	0	1
2114	7	chr7:151902167	MLL3	-	TT	0.005556	0	1
2117	7	chr7:151947941	MLL3	T	C	0.005556	0	1
2127	7	chr7:151971024	MLL3	A	G	0.005556	0	1
2567	10	chr10:21901248	MLLT10	T	-	0.01667	0	3
2568	10	chr10:21901253	MLLT10	T	G	0.01667	0	3
2572	10	chr10:22022026	MLLT10	T	A	0.005556	0	1
76	1	chr1:43803502	MPL	T	C	0.005556	0	1
82	1	chr1:43818486	MPL	T	C	0.005556	0	1
2781	11	chr11:94224026	MRE11A	G	T	0.005556	0	1
538	2	chr2:47639586	MSH2	T	A	0.005556	0	1
540	2	chr2:47641570	MSH2	G	A	0.005556	0	1
542	2	chr2:47643502	MSH2	C	A	0.02222	1	2
548	2	chr2:47705460	MSH2	-	ACTTCT	0.005556	0	1
35	1	chr1:11167502	MTOR	C	A	0.005556	0	1
457	1	chr1:236966821	MTR	TT	GG	0.02778	0	5
466	1	chr1:237016242	MTR	A	C	0.005556	0	1
469	1	chr1:237038012	MTR	T	A	0.005556	0	1
475	1	chr1:237058719	MTR	G	A	0.005556	0	1
322	1	chr1:155160052	MUC1	GAC	ACT	0.01111	1	0
325	1	chr1:155160768	MUC1	G	A	0.005556	0	1
92	1	chr1:45799181	MUTYH	T	G	0.01111	1	0
2338	8	chr8:128753001	MYC	TGA	-	0.01111	1	0
2340	8	chr8:128753260	MYC	T	G	0.005556	0	1

75	1	chr1:40363605	MYCL1	A	C	0.005556	0	1
490	2	chr2:16086048	MYCN	A	G	0.005556	0	1
3376	16	chr16:15808865	MYH11	G	A	0.005556	0	1
3391	16	chr16:15826573	MYH11	A	G	0.005556	0	1
4122	22	chr22:36690119	MYH9	CC	GT	0.005556	0	1
2225	8	chr8:71041018	NCOA2	A	C	0.005556	0	1
2226	8	chr8:71041202	NCOA2	C	T	0.005556	0	1
2231	8	chr8:71075661	NCOA2	G	A	0.005556	0	1
2232	8	chr8:71075779	NCOA2	C	G	0.01111	1	0
2234	8	chr8:71128917	NCOA2	G	A	0.005556	0	1
2593	10	chr10:51584910	NCOA4	A	G	0.01111	0	2
2595	10	chr10:51586458	NCOA4	ATAT	-	0.005556	0	1
3599	17	chr17:29527684	NF1	A	G	0.005556	0	1
3604	17	chr17:29563074	NF1	TTG	-	0.01111	0	2
3615	17	chr17:29684016	NF1	G	T	0.005556	0	1
670	2	chr2:178096253	NFE2L2	G	A	0.005556	0	1
1176	4	chr4:103533242	NFKB1	G	T	0.005556	0	1
1177	4	chr4:103533291	NFKB1	A	C	0.005556	0	1
1178	4	chr4:103533656	NFKB1	A	C	0.005556	0	1
1180	4	chr4:103537741	NFKB1	T	G	0.005556	0	1
2663	10	chr10:104156192	NFKB2	A	C	0.005556	0	1
3091	14	chr14:51221316	NIN	G	C	0.01111	1	0
3097	14	chr14:51225320	NIN	C	T	0.005556	0	1
3546	17	chr17:5487204	NLRP1	A	T	0.005556	0	1
147	1	chr1:120477935	NOTCH2	A	G	0.005556	0	1
159	1	chr1:120539655	NOTCH2	C	G	0.005556	0	1
1438	6	chr6:32164107	NOTCH4	A	G	0.005556	0	1
1442	6	chr6:32166734	NOTCH4	A	G	0.005556	0	1
1384	5	chr5:176637204	NSD1	C	T	0.01111	0	2
328	1	chr1:156811985	NTRK1	C	G	0.005556	0	1
329	1	chr1:156841496	NTRK1	A	G	0.005556	0	1
3280	15	chr15:88669573	NTRK3	A	G	0.005556	0	1
2506	9	chr9:134090676	NUP214	G	A	0.01111	1	0
2714	11	chr11:3794852	NUP98	A	T	0.005556	0	1
4245	23	chrX:110463549	PAK3	T	G	0	0	0
2430	9	chr9:37006542	PAX5	A	C	0.005556	0	1
57	1	chr1:19018422	PAX7	T	C	0.005556	0	1
901	3	chr3:52610602	PBRM1	C	A	0.005556	0	1
902	3	chr3:52610619	PBRM1	G	A	0.005556	0	1
161	1	chr1:144852315	PDE4DIP	T	C	0.04444	1	6
181	1	chr1:144859929	PDE4DIP	T	C	0.02778	0	5
182	1	chr1:144863360	PDE4DIP	C	T	0.02222	0	4
183	1	chr1:144863366	PDE4DIP	A	G	0.005556	0	1
184	1	chr1:144863384	PDE4DIP	A	C	0.005556	0	1
185	1	chr1:144863388	PDE4DIP	TT	GA	0.005556	0	1
192	1	chr1:144866660	PDE4DIP	T	G	0.005556	0	1
194	1	chr1:144866673	PDE4DIP	A	G	0.01111	0	2

208	1	**chr1:144879054**	PDE4DIP	C	T	0.02222	0	4
209	1	**chr1:144879086**	PDE4DIP	-	T	0.005556	0	1
216	1	**chr1:144881463**	PDE4DIP	T	C	0.02778	0	5
217	1	**chr1:144881547**	PDE4DIP	A	G	0.05	0	9
225	1	**chr1:144886092**	PDE4DIP	T	C	0.3667	0	66
227	1	**chr1:144886267**	PDE4DIP	T	G	0.02222	0	4
246	1	**chr1:144923712**	PDE4DIP	C	T	0.005556	0	1
247	1	**chr1:144930571**	PDE4DIP	A	C	0.4222	0	76
250	1	**chr1:144930977**	PDE4DIP	C	A	0.01111	0	2
255	1	**chr1:144931423**	PDE4DIP	C	T	0.005556	0	1
259	1	**chr1:144994617**	PDE4DIP	T	C	0.01667	0	3
261	1	**chr1:144994670**	PDE4DIP	A	C	0.03333	0	6
262	1	**chr1:144994694**	PDE4DIP	C	T	0.005556	0	1
1095	4	**chr4:55129892**	PDGFRA	C	T	0.01111	1	0
1096	4	**chr4:55129894**	PDGFRA	G	A	0.02222	2	0
1360	5	**chr5:149499530**	PDGFRB	T	A	0.1111	0	20
1366	5	**chr5:149514424**	PDGFRB	A	C	0.005556	0	1
3571	17	**chr17:8045566**	PER1	GC	AA	0.005556	0	1
418	1	**chr1:204438894**	PIK3C2B	G	A	0.005556	0	1
1012	3	**chr3:178916986**	PIK3CA	A	C	0.005556	0	1
1014	3	**chr3:178922273**	PIK3CA	AA	CC	0.005556	0	1
975	3	**chr3:138461524**	PIK3CB	G	A	0.005556	0	1
2019	7	**chr7:106545513**	PIK3CG	G	A	0.005556	0	1
1281	5	**chr5:67522726**	PIK3R1	T	G	0.005556	0	1
1286	5	**chr5:67590496**	PIK3R1	T	G	0.005556	0	1
1487	6	**chr6:51524653**	PKHD1	T	A	0.005556	0	1
1490	6	**chr6:51613058**	PKHD1	T	G	0.005556	0	1
1508	6	**chr6:51889459**	PKHD1	A	T	0.005556	0	1
1513	6	**chr6:51890498**	PKHD1	A	C	0.005556	0	1
1527	6	**chr6:51921815**	PKHD1	GCCTT	-	0.02222	2	0
1528	6	**chr6:51923126**	PKHD1	T	C	0.005556	0	1
677	2	**chr2:190719333**	PMS1	C	A	0.005556	0	1
678	2	**chr2:190719788**	PMS1	A	G	0.005556	0	1
1866	7	**chr7:6027040**	PMS2	C	A	0.005556	0	1
1867	7	**chr7:6029511**	PMS2	T	A	0.01111	0	2
1868	7	**chr7:6035156**	PMS2	G	T	0.005556	0	1
2033	7	**chr7:124475481**	POT1	G	T	0.005556	0	1
3704	17	**chr17:66511530**	PRKAR1A	G	C	0.01111	1	0
2190	8	**chr8:48686929**	PRKDC	C	T	0.005556	0	1
2199	8	**chr8:48736499**	PRKDC	G	C	0.005556	0	1
2205	8	**chr8:48776046**	PRKDC	G	A	0.005556	0	1
2212	8	**chr8:48825021**	PRKDC	C	G	0.005556	0	1
2213	8	**chr8:48828031**	PRKDC	C	A	0.005556	0	1
2453	9	**chr9:98212211**	PTCH1	T	G	0.005556	0	1
2467	9	**chr9:98241253**	PTCH1	C	T	0.005556	0	1
388	1	**chr1:186646996**	PTGS2	A	C	0.005556	0	1
389	1	**chr1:186647541**	PTGS2	C	A	0.005556	0	1

2971	12	**chr12:112910806**	PTPN11	A	-	0.005556	0	1
2382	9	**chr9:8460647**	PTPRD	-	A	0.01111	1	0
2390	9	**chr9:8485904**	PTPRD	T	A	0.005556	0	1
2391	9	**chr9:8485984**	PTPRD	T	C	0.005556	0	1
4030	20	**chr20:40743814**	PTPRT	G	T	0.01111	1	0
1330	5	**chr5:131924445**	RAD50	A	C	0.005556	0	1
804	3	**chr3:12626400**	RAF1	T	C	0.005556	0	1
805	3	**chr3:12626737**	RAF1	A	G	0.005556	0	1
808	3	**chr3:12641349**	RAF1	GG	AA	0.005556	0	1
2519	9	**chr9:135981338**	RALGDS	T	C	0.005556	0	1
3638	17	**chr17:38504723**	RARA	C	T	0.005556	0	1
2342	8	**chr8:145737307**	RECQL4	A	G	0.005556	0	1
563	2	**chr2:61147771**	REL	-	A	0.01111	0	2
564	2	**chr2:61147775**	REL	G	A	0.01111	0	2
362	1	**chr1:185056670**	RNF2	G	T	0.01111	0	2
363	1	**chr1:185067185**	RNF2	-	T	0.005556	0	1
3728	17	**chr17:78262057**	RNF213	T	A	0.005556	0	1
3730	17	**chr17:78264358**	RNF213	G	T	0.005556	0	1
3734	17	**chr17:78268717**	RNF213	T	A	0.005556	0	1
3754	17	**chr17:78319102**	RNF213	T	C	0.005556	0	1
3777	17	**chr17:78351613**	RNF213	T	A	0.01111	0	2
3782	17	**chr17:78357518**	RNF213	C	-	0.005556	0	1
1656	6	**chr6:117710658**	ROS1	G	T	0.005556	0	1
1838	6	**chr6:166952128**	RPS6KA2	T	C	0.005556	0	1
2246	8	**chr8:92972668**	RUNX1T1	C	G	0.01111	1	0
2248	8	**chr8:93026955**	RUNX1T1	A	G	0.005556	0	1
2249	8	**chr8:93029480**	RUNX1T1	A	C	0.005556	0	1
1209	5	**chr5:233504**	SDHA	C	T	0.01667	0	3
887	3	**chr3:47155487**	SETD2	A	G	0.005556	0	1
892	3	**chr3:47163994**	SETD2	G	A	0.005556	0	1
682	2	**chr2:198262720**	SF3B1	G	T	0.005556	0	1
1674	6	**chr6:134498787**	SGK1	T	C	0.005556	0	1
1675	6	**chr6:134528524**	SGK1	A	G	0.005556	0	1
3818	18	**chr18:48575204**	SMAD4	T	A	0.005556	0	1
3824	18	**chr18:48593440**	SMAD4	C	T	0.005556	0	1
3921	19	**chr19:11170500**	SMARCA4	T	C	0.005556	0	1
4101	22	**chr22:24145672**	SMARCB1	C	-	0.03333	3	0
4106	22	**chr22:24167632**	SMARCB1	C	T	0.03333	0	6
487	2	**chr2:5833816**	SOX11	T	-	0.01111	1	0
1021	3	**chr3:181430876**	SOX2	C	T	0.005556	0	1
4010	20	**chr20:36031361**	SRC	T	C	0.005556	0	1
4011	20	**chr20:36031767**	SRC	G	C	0.01111	0	2
746	2	**chr2:219544800**	STK36	T	C	0.005556	0	1
2675	10	**chr10:104357022**	SUFU	T	C	0.005556	0	1
2433	9	**chr9:93606313**	SYK	A	C	0.005556	0	1
1703	6	**chr6:152457915**	SYNE1	T	G	0.005556	0	1
1712	6	**chr6:152470784**	SYNE1	T	G	0.005556	0	1

1713	6	chr6:152472812	SYNE1	C	T	0.005556	0	1
1721	6	chr6:152529254	SYNE1	A	G	0.005556	0	1
1725	6	chr6:152542688	SYNE1	C	G	0.005556	0	1
1740	6	chr6:152631545	SYNE1	A	G	0.005556	0	1
1741	6	chr6:152631970	SYNE1	A	C	0.005556	0	1
1742	6	chr6:152639288	SYNE1	A	C	0.005556	0	1
1744	6	chr6:152644654	SYNE1	A	C	0.005556	0	1
1752	6	chr6:152652197	SYNE1	C	A	0.005556	0	1
1764	6	chr6:152674791	SYNE1	C	T	0.005556	0	1
1782	6	chr6:152712677	SYNE1	A	C	0.005556	0	1
1805	6	chr6:152823897	SYNE1	T	A	0.005556	0	1
4229	23	chrX:70597573	TAF1	C	G	0.008	0	1
4235	23	chrX:70683793	TAF1	T	C	0	0	0
2411	9	chr9:32631748	TAF1L	A	G	0.005556	0	1
4240	23	chrX:79279609	TBX22	C	G	0.008	0	1
3877	19	chr19:1646325	TCF3	CGAG	-	0.01111	0	2
573	2	chr2:85534779	TCF7L1	-	GAGG	0.005556	0	1
2680	10	chr10:114710518	TCF7L2	T	G	0.005556	0	1
2684	10	chr10:114911539	TCF7L2	T	C	0.005556	0	1
2599	10	chr10:70332342	TET1	G	A	0.005556	0	1
2609	10	chr10:70406386	TET1	A	C	0.01111	1	0
2614	10	chr10:70451510	TET1	A	T	0.005556	0	1
1187	4	chr4:106158026	TET2	T	A	0.005556	0	1
1191	4	chr4:106193885	TET2	-	T	0.005556	0	1
4213	23	chrX:48896874	TFE3	T	C	0.008	0	1
3203	15	chr15:39884882	THBS1	T	G	0.01111	0	2
3569	17	chr17:7579634	TP53	-	C	0.005556	0	1
370	1	chr1:186301473	TPR	T	A	0.005556	0	1
372	1	chr1:186304551	TPR	G	A	0.005556	0	1
374	1	chr1:186315243	TPR	T	-	0.01111	0	2
136	1	chr1:114940315	TRIM33	A	G	0.005556	0	1
141	1	chr1:114976234	TRIM33	T	C	0.005556	0	1
3132	14	chr14:92454746	TRIP11	T	-	0.005556	0	1
3135	14	chr14:92461911	TRIP11	T	A	0.01111	0	2
3141	14	chr14:92471880	TRIP11	T	C	0.005556	0	1
1971	7	chr7:98543325	TRRAP	G	A	0.005556	0	1
1973	7	chr7:98552756	TRRAP	C	A	0.005556	0	1
1974	7	chr7:98552760	TRRAP	T	C	0.005556	0	1
1975	7	chr7:98552779	TRRAP	T	C	0.005556	0	1
1982	7	chr7:98579357	TRRAP	T	C	0.005556	0	1
3336	16	chr16:2129453	TSC2	T	-	0.02222	2	0
3115	14	chr14:81554286	TSHR	C	T	0.005556	0	1
3116	14	chr14:81554303	TSHR	G	T	0.005556	0	1
3121	14	chr14:81606071	TSHR	A	G	0.005556	0	1
2255	8	chr8:103284936	UBR5	T	C	0.005556	0	1
2257	8	chr8:103289351	UBR5	C	T	0.005556	0	1
2261	8	chr8:103297404	UBR5	G	T	0.005556	0	1

2266	8	**chr8:103307466**	UBR5	C	T	0.005556	0	1
2280	8	**chr8:103341456**	UBR5	-	T	0.01111	0	2
2281	8	**chr8:103354860**	UBR5	C	G	0.005556	0	1
4197	23	**chrX:41088723**	USP9X	T	A	0.008	0	1
1083	4	**chr4:1961342**	WHSC1	T	C	0.005556	0	1
1084	4	**chr4:1976481**	WHSC1	G	T	0.04444	4	0
1085	4	**chr4:1976483**	WHSC1	G	C	0.03333	3	0
1086	4	**chr4:1976485**	WHSC1	GG	AC	0.005556	0	1
1087	4	**chr4:1976486**	WHSC1	G	C	0.005556	0	1
2140	8	**chr8:30938754**	WRN	-	T	0.005556	0	1
2145	8	**chr8:30989869**	WRN	C	A	0.005556	0	1
2152	8	**chr8:31004834**	WRN	A	T	0.005556	0	1
2157	8	**chr8:31024684**	WRN	T	G	0.005556	0	1
2870	12	**chr12:6777111**	ZNF384	-	C	0.06667	6	0
2873	12	**chr12:6788242**	ZNF384	C	T	0.005556	0	1

Variants reported in ICGC

A2A2	O(HET)	E(HET)	P	Location	Allele	Consequen	IMPACT	SYMBOL
89	0.01111	0.01105	1	2:1486728	T	stop_gaine	HIGH	ACVR2A
89	0.01111	0.01105	1	12:438462	T	intron_vari	MODIFIER	ADAMTS20
89	0.01111	0.01105	1	4:8796727	C	intron_vari	MODIFIER	AFF1
89	0.01111	0.01105	1	4:8796793	T	synonymou	LOW	AFF1
89	0.01111	0.01105	1	7:9163123	C	missense_v	MODERATE	AKAP9
89	0.01111	0.01105	1	7:9164167	TGC	intron_vari	MODIFIER	AKAP9
89	0.01111	0.01105	1	7:9167006	C	missense_v	MODERATE	AKAP9
89	0.01111	0.01105	1	7:9167209	G	intron_vari	MODIFIER	AKAP9
89	0.01111	0.01105	1	7:9169937	A	frameshift_	HIGH	AKAP9
89	0.01111	0.01105	1	7:9170915	G	missense_v	MODERATE	AKAP9
89	0.01111	0.01105	1	7:9171272	A	missense_v	MODERATE	AKAP9
89	0.01111	0.01105	1	19:407412	A	missense_v	MODERATE	AKT2
89	0.01111	0.01105	1	1:2437272	-	intron_vari	MODIFIER	AKT3
89	0.01111	0.01105	1	2:2949830	-	frameshift_	HIGH	ALK
89	0.01111	0.01105	1	5:1121646	A	missense_v	MODERATE	APC
89	0.01111	0.01105	1	5:1121646	-	frameshift_	HIGH	APC
89	0.01111	0.01105	1	5:1121739	TA	stop_gaine	HIGH	APC
89	0.01111	0.01105	1	5:1121752	-	frameshift_	HIGH	APC
89	0.01111	0.01105	1	5:1121756	-	frameshift_	HIGH	APC
31	0	0.2024	5.546e-006	X:6694360	-	frameshift_	HIGH	AR
89	0.01111	0.01105	1	1:2709281	-	frameshift_	HIGH	ARID1A
89	0.01111	0.01105	1	1:2709282	T	missense_v	MODERATE	ARID1A
87	0.02222	0.04346	0.03342	1:2709769	A	stop_gaine	HIGH	ARID1A
89	0	0.02198	0.005587	1:2709900	-	frameshift_	HIGH	ARID1A
89	0.01111	0.01105	1	1:2710634	T	missense_v	MODERATE	ARID1A
89	0.01111	0.01105	1	12:462856	C	missense_v	MODERATE	ARID2
89	0.01111	0.01105	1	1:1508089	A	intron_vari	MODIFIER	ARNT
89	0.01111	0.01105	1	11:108117	TTA	intron_vari	MODIFIER	ATM
89	0.01111	0.01105	1	11:108175	G	intron_vari	MODIFIER	ATM
89	0.01111	0.01105	1	11:108201	C	synonymou	LOW	ATM
89	0.01111	0.01105	1	3:1421766	-	intron_vari	MODIFIER	ATR
89	0.01111	0.01105	1	3:1421766	A	intron_vari	MODIFIER	ATR
89	0.01111	0.01105	1	3:1421868	-	frameshift_	HIGH	ATR
89	0.01111	0.01105	1	3:1422691	G	missense_v	MODERATE	ATR
88	0.02222	0.02198	1	3:1422726	A	intron_vari	MODIFIER	ATR
89	0.01111	0.01105	1	3:1422746	A	intron_vari	MODIFIER	ATR
34	0.02857	0.02816	1	X:7684916	T	splice_don	HIGH	ATRX
89	0.01111	0.01105	1	20:549593	T	frameshift_	HIGH	AURKA
89	0.01111	0.01105	1	17:810987	C	synonymou	LOW	AURKB
89	0.01111	0.01105	1	19:577447	T	intron_vari	MODIFIER	AURKC
89	0.01111	0.01105	1	6:6934919	A	missense_v	MODERATE	BAI3
89	0.01111	0.01105	1	6:7007124	G	missense_v	MODERATE	BAI3
89	0.01111	0.01105	1	1:8573337	C	synonymou	LOW	BCL10
89	0.01111	0.01105	1	1:8573659	T	splice_regi	LOW	BCL10
89	0.01111	0.01105	1	2:6077306	G	intron_vari	MODIFIER	BCL11A

89	0.01111	0.01105	1	3:1874470	A	missense_\	MODERATE	BCL6
89	0.01111	0.01105	1	1:1470906	T	stop_gaine	HIGH	BCL9
89	0.01111	0.01105	1	1:1470940	T	intron_vari	MODIFIER	BCL9
89	0.01111	0.01105	1	11:102195	C	missense_\	MODERATE	BIRC3
89	0.01111	0.01105	1	11:102195	T	missense_\	MODERATE	BIRC3
89	0.01111	0.01105	1	11:102201	C	synonymou	LOW	BIRC3
88	0.02222	0.02198	1	11:102207	A	missense_\	MODERATE	BIRC3
89	0.01111	0.01105	1	11:102207	C	missense_\	MODERATE	BIRC3
89	0.01111	0.01105	1	17:762103	A	5_prime_U	MODIFIER	BIRC5
85	0.05556	0.05401	1	17:762196	T	3_prime_U	MODIFIER	BIRC5
88	0.02222	0.02198	1	15:913041	T	missense_\	MODERATE	BLM
89	0.01111	0.01105	1	15:913474	A	missense_\	MODERATE	BLM
84	0.06667	0.06444	1	10:979751	-	intron_vari	MODIFIER	BLNK
89	0.01111	0.01105	1	17:597606	C	3_prime_U	MODIFIER	BRIP1
89	0.01111	0.01105	1	17:598581	T	intron_vari	MODIFIER	BRIP1
89	0.01111	0.01105	1	17:598615	A	intron_vari	MODIFIER	BRIP1
89	0.01111	0.01105	1	7:2968269	C	missense_\	MODERATE	CARD11
89	0.01111	0.01105	1	15:409138	T	missense_\	MODERATE	CASC5
89	0.01111	0.01105	1	15:409495	-	intron_vari	MODIFIER	CASC5
89	0.01111	0.01105	1	11:119145	-	intron_vari	MODIFIER	CBL
89	0.01111	0.01105	1	1:1931109	G	intron_vari	MODIFIER	CDC73
89	0.01111	0.01105	1	1:1931112	A	intron_vari	MODIFIER	CDC73
89	0.01111	0.01105	1	16:688440	T	intron_vari	MODIFIER	CDH1
89	0.01111	0.01105	1	16:650269	G	intron_vari	MODIFIER	CDH11
89	0.01111	0.01105	1	18:255898	A	intron_vari	MODIFIER	CDH2
89	0.01111	0.01105	1	18:255937	A	missense_\	MODERATE	CDH2
85	0.05556	0.05401	1	18:592218	A	missense_\	MODERATE	CDH20
89	0.01111	0.01105	1	17:376277	G	missense_\	MODERATE	CDK12
89	0.01111	0.01105	1	12:581431	-	intron_vari	MODIFIER	CDK4
89	0.01111	0.01105	1	9:2199415	C	intron_vari	MODIFIER	RP11-145E
89	0.01111	0.01105	1	19:427910	T	synonymou	LOW	CIC
89	0.01111	0.01105	1	19:427984	G	splice_regi	LOW	CIC
88	0.01111	0.03278	0.01676	17:482770	-	intron_vari	MODIFIER	COL1A1
89	0.01111	0.01105	1	3:3194271	C	missense_\	MODERATE	CRBN
89	0.01111	0.01105	1	3:3197988	G	intron_vari	MODIFIER	CRBN
89	0.01111	0.01105	1	16:384361	G	synonymou	LOW	CREBBP
88	0.02222	0.02198	1	8:1132540	-	intron_vari	MODIFIER	CSMD3
89	0.01111	0.01105	1	8:1133017	TTA	intron_vari	MODIFIER	CSMD3
89	0.01111	0.01105	1	8:1133233	G	splice_acce	HIGH	CSMD3
87	0.03333	0.03278	1	8:1135162	G	intron_vari	MODIFIER	CSMD3
89	0.01111	0.01105	1	8:1135689	G	intron_vari	MODIFIER	CSMD3
89	0.01111	0.01105	1	5:1382665	G	missense_\	MODERATE	CTNNA1
89	0.01111	0.01105	1	5:1382697	-	inframe_de	MODERATE	CTNNA1
89	0.01111	0.01105	1	3:4126719	A	missense_\	MODERATE	CTNNB1
89	0.01111	0.01105	1	10:965416	G	missense_\	MODERATE	CYP2C19
89	0.01111	0.01105	1	22:425248	T	missense_\	MODERATE	CYP2D6
89	0.01111	0.01105	1	18:504516	G	missense_\	MODERATE	DCC

89	0.01111	0.01105	1	18:504516	C	missense_	MODERATE	DCC
89	0.01111	0.01105	1	18:509425	T	missense_	MODERATE	DCC
89	0.01111	0.01105	1	18:509768	C	intron_vari	MODIFIER	DCC
88	0.02222	0.02198	1	1:1627371	T	splice_regi	LOW	DDR2
88	0.02222	0.02198	1	1:1627403	CG	intron_vari	MODIFIER	DDR2
89	0.01111	0.01105	1	1:1627483	T	missense_	MODERATE	DDR2
89	0.01111	0.01105	1	14:955698	A	synonymou	LOW	DICER1
89	0.01111	0.01105	1	2:2545714	G	stop_lost	HIGH	DNMT3A
89	0.01111	0.01105	1	2:2552301	A	missense_	MODERATE	DNMT3A
89	0.01111	0.01105	1	1:9756404	G	splice_regi	LOW	DPYD
89	0.01111	0.01105	1	1:9814476	T	intron_vari	MODIFIER	DPYD
89	0.01111	0.01105	1	1:9820604	C	intron_vari	MODIFIER	DPYD
89	0.01111	0.01105	1	1:9838648	A	5_prime_U	MODIFIER	DPYD
74	0.1778	0.162	1	6:5634110	A	missense_	MODERATE	DST
89	0.01111	0.01105	1	6:5634753	C	missense_	MODERATE	DST
89	0.01111	0.01105	1	6:5635015	C	synonymou	LOW	DST
89	0.01111	0.01105	1	6:5641714	G	missense_	MODERATE	DST
89	0.01111	0.01105	1	6:5641744	C	missense_	MODERATE	DST
89	0	0.02198	0.005587	6:5648509	-	intron_vari	MODIFIER	DST
89	0.01111	0.01105	1	6:5648526	C	intron_vari	MODIFIER	DST
89	0.01111	0.01105	1	6:5648946	-	intron_vari	MODIFIER	DST
87	0	0.06444	2.705e-00	22:415428	-	intron_vari	MODIFIER	EP300
89	0.01111	0.01105	1	22:415509	A	intron_vari	MODIFIER	EP300
89	0.01111	0.01105	1	12:132466	A	missense_	MODERATE	EP400
89	0.01111	0.01105	1	12:132490	A	missense_	MODERATE	EP400
89	0.01111	0.01105	1	12:132497	T	synonymou	LOW	EP400
89	0.01111	0.01105	1	12:132529	C	intron_vari	MODIFIER	EP400
89	0.01111	0.01105	1	12:132535	G	missense_	MODERATE	EP400
89	0	0.02198	0.005587	12:132562	C	missense_	MODERATE	EP400
89	0.01111	0.01105	1	3:8944846	C	splice_regi	LOW	EPHA3
89	0.01111	0.01105	1	3:8946227	C	intron_vari	MODIFIER	EPHA3
89	0.01111	0.01105	1	3:8946229	C	missense_	MODERATE	EPHA3
83	0.04444	0.1049	0.0005534	6:9396800	C	splice_acce	HIGH	EPHA7
89	0.01111	0.01105	1	6:9397353	G	intron_vari	MODIFIER	EPHA7
89	0.01111	0.01105	1	6:9412896	C	splice_regi	LOW	EPHA7
89	0.01111	0.01105	1	3:1348858	T	synonymou	LOW	EPHB1
89	0.01111	0.01105	1	17:378683	A	intron_vari	MODIFIER	ERBB2
89	0.01111	0.01105	1	17:378687	C	intron_vari	MODIFIER	ERBB2
89	0.01111	0.01105	1	17:378836	T	synonymou	LOW	ERBB2
89	0.01111	0.01105	1	12:564790	T	intron_vari	MODIFIER	ERBB3
89	0.01111	0.01105	1	2:2122486	T	synonymou	LOW	ERBB4
89	0.01111	0.01105	1	2:2124951	T	intron_vari	MODIFIER	ERBB4
89	0.01111	0.01105	1	2:2129894	T	intron_vari	MODIFIER	ERBB4
89	0	0.02198	0.005587	21:398174	C	synonymou	LOW	ERG
89	0.01111	0.01105	1	7:1395094	T	intron_vari	MODIFIER	ETV1
89	0.01111	0.01105	1	17:416105	G	missense_	MODERATE	ETV4
89	0.01111	0.01105	1	8:1188251	A	missense_	MODERATE	EXT1

89	0.01111	0.01105	1	8:1191224	A	synonymou	LOW	EXT1
89	0.01111	0.01105	1	11:442285	-	intron_vari	MODIFIER	EXT2
87	0.03333	0.03278	1	7:1485235	A	intron_vari	MODIFIER	EZH2
34	0.02857	0.02816	1	X:6341163	ATAGAACT	frameshift_	HIGH	AMER1
89	0.01111	0.01105	1	16:898577	C	intron_vari	MODIFIER	FANCA
89	0.01111	0.01105	1	16:898773	A	intron_vari	MODIFIER	FANCA
89	0.01111	0.01105	1	3:1008829	G	missense_v	MODERATE	FANCD2
88	0.02222	0.02198	1	3:1013203	-	frameshift_	HIGH	FANCD2
89	0	0.02198	0.005587	19:814601	G	intron_vari	MODIFIER	FBN3
89	0.01111	0.01105	1	4:1532440	A	stop_gaine	HIGH	FBXW7
82	0.08889	0.08494	1	8:3827235	A	synonymou	LOW	FGFR1
89	0.01111	0.01105	1	10:123246	C	missense_v	MODERATE	FGFR2
89	0.01111	0.01105	1	4:1803325	-	intron_vari	MODIFIER	FGFR3
88	0.02222	0.02198	1	4:1807544	-	frameshift_	HIGH	FGFR3
89	0.01111	0.01105	1	5:1765204	A	synonymou	LOW	FGFR4
88	0	0.04346	9.469e-005	13:285974	-	intron_vari	MODIFIER	FLT3
89	0.01111	0.01105	1	5:1800359	G	missense_v	MODERATE	FLT4
89	0.01111	0.01105	1	2:2162325	G	intron_vari	MODIFIER	FN1
89	0.01111	0.01105	1	2:2162370	C	missense_v	MODERATE	FN1
89	0.01111	0.01105	1	2:2162729	A	intron_vari	MODIFIER	FN1
89	0.01111	0.01105	1	13:411348	C	missense_v	MODERATE	FOXO1
89	0.01111	0.01105	1	6:1089852	G	missense_v	MODERATE	FOXO3
89	0.01111	0.01105	1	3:7117970	C	frameshift_	HIGH	FOXP1
89	0.01111	0.01105	1	6:4155905	T	missense_v	MODERATE	FOXP4
89	0.01111	0.01105	1	19:353182	G	intron_vari	MODIFIER	FZR1
84	0.05556	0.07475	0.114	3:1282027	C	synonymou	LOW	GATA2
68	0.2444	0.2146	0.3487	20:574284	G	synonymou	LOW	GNAS
89	0.01111	0.01105	1	8:3769141	A	intron_vari	MODIFIER	GPR124
89	0	0.02198	0.005587	2:1175100	T	missense_v	MODERATE	GREB1
89	0.01111	0.01105	1	11:106579	A	synonymou	LOW	GUCY1A2
89	0.01111	0.01105	1	8:4286845	-	intron_vari	MODIFIER	HOOK3
89	0.01111	0.01105	1	14:102548	G	missense_v	MODERATE	HSP90AA1
89	0.01111	0.01105	1	14:102548	C	intron_vari	MODIFIER	HSP90AA1
89	0.01111	0.01105	1	15:906282	A	missense_v	MODERATE	IDH2
89	0.01111	0.01105	1	15:992507	C	intron_vari	MODIFIER	IGF1R
89	0.01111	0.01105	1	15:994601	T	intron_vari	MODIFIER	IGF1R
88	0.02222	0.02198	1	6:1604121	T	intron_vari	MODIFIER	IGF2R
89	0.01111	0.01105	1	6:1604910	G	missense_v	MODERATE	IGF2R
89	0.01111	0.01105	1	8:4217529	C	splice_regi	LOW	IKBKB
89	0.01111	0.01105	1	1:2066476	T	intron_vari	MODIFIER	IKBKE
89	0.01111	0.01105	1	5:5523709	C	missense_v	MODERATE	IL6ST
89	0.01111	0.01105	1	5:5523759	A	missense_v	MODERATE	IL6ST
89	0.01111	0.01105	1	5:5524726	-	intron_vari	MODIFIER	IL6ST
89	0.01111	0.01105	1	5:5525634	T	missense_v	MODERATE	IL6ST
89	0.01111	0.01105	1	5:5526428	G	intron_vari	MODIFIER	IL6ST
89	0.01111	0.01105	1	6:397155-3	T	synonymou	LOW	IRF4
89	0.01111	0.01105	1	13:110434	T	synonymou	LOW	IRS2

89	0.01111	0.01105	1	1:1455283	T	stop_gaine	HIGH	ITGA10
89	0.01111	0.01105	1	1:1455283	A	splice_regi	LOW	ITGA10
86	0.04444	0.04346	1	1:1455309	T	missense_v	MODERATE	ITGA10
87	0.03333	0.03278	1	1:1455338	-	frameshift_	HIGH	ITGA10
89	0.01111	0.01105	1	3:3756751	C	splice_acce	HIGH	ITGA9
89	0.01111	0.01105	1	3:3779190	A	intron_vari	MODIFIER	ITGA9
88	0	0.04346	9.469e-005	21:463270	A	intron_vari	MODIFIER	ITGB2
89	0.01111	0.01105	1	17:453608	A	missense_v	MODERATE	ITGB3
89	0.01111	0.01105	1	17:453617	T	intron_vari	MODIFIER	ITGB3
89	0.01111	0.01105	1	17:453778	C	missense_v	MODERATE	ITGB3
89	0.01111	0.01105	1	17:453778	A	missense_v	MODERATE	ITGB3
89	0	0.02198	0.005587	9:5081778	TA	stop_gaine	HIGH	JAK2
89	0.01111	0.01105	1	9:5090443	G	splice_regi	LOW	JAK2
89	0	0.02198	0.005587	8:4179104	A	frameshift_	HIGH	KAT6A
79	0.1111	0.1244	0.3243	10:767354	-	frameshift_	HIGH	KAT6B
89	0.01111	0.01105	1	10:767360	C	missense_v	MODERATE	KAT6B
89	0.01111	0.01105	1	10:767449	-	frameshift_	HIGH	KAT6B
89	0.01111	0.01105	1	10:767449	T	missense_v	MODERATE	KAT6B
88	0.02222	0.02198	1	10:767449	G	missense_v	MODERATE	KAT6B
35	0	0	1	X:5324505	T	missense_v	MODERATE	KDM5C
34	0.02857	0.02816	1	X:4494286	C	intron_vari	MODIFIER	KDM6A
87	0	0.06444	2.705e-006	4:5595374	-	intron_vari	MODIFIER	KDR
89	0.01111	0.01105	1	4:5597090	G	missense_v	MODERATE	KDR
89	0.01111	0.01105	1	4:5598029	A	missense_v	MODERATE	KDR
89	0.01111	0.01105	1	4:5598150	T	frameshift_	HIGH	KDR
89	0.01111	0.01105	1	19:106005	A	missense_v	MODERATE	KEAP1
89	0.01111	0.01105	1	10:382427	C	missense_v	MODERATE	KLF6
89	0.01111	0.01105	1	12:253982	CGC	inframe_in	MODERATE	KRAS
89	0.01111	0.01105	1	5:3848187	G	missense_v	MODERATE	LIFR
89	0.01111	0.01105	1	5:3848232	T	splice_regi	LOW	LIFR
89	0.01111	0.01105	1	5:3848609	T	intron_vari	MODIFIER	LIFR
89	0.01111	0.01105	1	5:3849971	A	intron_vari	MODIFIER	LIFR
89	0.01111	0.01105	1	5:3850282	C	missense_v	MODERATE	LIFR
89	0.01111	0.01105	1	4:6259909	G	missense_v	MODERATE	LPHN3
89	0.01111	0.01105	1	4:6289731	A	missense_v	MODERATE	LPHN3
89	0.01111	0.01105	1	2:1410794	G	intron_vari	MODIFIER	LRP1B
83	0	0.1435	2.919e-011	2:1412020	CA	splice_regi	LOW	LRP1B
89	0.01111	0.01105	1	2:1412140	G	synonymou	LOW	LRP1B
89	0.01111	0.01105	1	2:1412329	C	intron_vari	MODIFIER	LRP1B
89	0.01111	0.01105	1	2:1412993	T	missense_v	MODERATE	LRP1B
89	0.01111	0.01105	1	2:1415985	C	missense_v	MODERATE	LRP1B
89	0.01111	0.01105	1	2:1416079	T	splice_regi	LOW	LRP1B
89	0.01111	0.01105	1	2:1416806	C	missense_v	MODERATE	LRP1B
89	0.01111	0.01105	1	2:1418197	T	synonymou	LOW	LRP1B
89	0.01111	0.01105	1	2:1419866	A	intron_vari	MODIFIER	LRP1B
89	0.01111	0.01105	1	2:1420049	C	intron_vari	MODIFIER	LRP1B
89	0.01111	0.01105	1	2:1428881	TA	intron_vari	MODIFIER	LRP1B

89	0.01111	0.01105	1	3:4650131(	A	splice_acce	HIGH	LTF
89	0.01111	0.01105	1	3:6537692(	T	synonymou	LOW	MAGI1
89	0.01111	0.01105	1	11:957248	C	missense_v	MODERATE	MAML2
89	0.01111	0.01105	1	11:958262	A	synonymou	LOW	MAML2
89	0.01111	0.01105	1	11:958263	A	missense_v	MODERATE	MAML2
89	0.01111	0.01105	1	15:667820	T	missense_v	MODERATE	MAP2K1
88	0.02222	0.02198	1	1:2208086	GC	intron_vari	MODIFIER	MARK1
89	0.01111	0.01105	1	19:458058	A	missense_v	MODERATE	MARK4
84	0.06667	0.06444	1	18:477939	G	upstream_	MODIFIER	CCDC11
89	0	0.02198	0.005587	18:478012(	C	intron_vari	MODIFIER	MBD1
89	0.01111	0.01105	1	1:1505507	G	missense_v	MODERATE	MCL1
89	0.01111	0.01105	1	11:645720	A	synonymou	LOW	MEN1
89	0.01111	0.01105	1	7:1163398(	G	missense_v	MODERATE	MET
79	0.1222	0.1148	1	7:1163977	G	missense_v	MODERATE	MET
89	0.01111	0.01105	1	3:6998582	C	intron_vari	MODIFIER	MITF
89	0.01111	0.01105	1	3:7000091(	G	intron_vari	MODIFIER	MITF
89	0.01111	0.01105	1	3:3709004	A	missense_v	MODERATE	MLH1
89	0.01111	0.01105	1	11:118376	T	missense_v	MODERATE	KMT2A
89	0.01111	0.01105	1	11:118390	A	missense_v	MODERATE	KMT2A
89	0	0.02198	0.005587	12:494331(	C	intron_vari	MODIFIER	KMT2D
89	0.01111	0.01105	1	7:1518596	A	synonymou	LOW	KMT2C
86	0	0.08494	1.095e-007	7:1518792	-	frameshift_	HIGH	KMT2C
89	0	0.02198	0.005587	7:1519021(	T	intron_vari	MODIFIER	KMT2C
89	0.01111	0.01105	1	7:1519021(	T	intron_vari	MODIFIER	KMT2C
89	0.01111	0.01105	1	7:1519021(	-	intron_vari	MODIFIER	KMT2C
89	0.01111	0.01105	1	7:1519479	T	missense_v	MODERATE	KMT2C
89	0.01111	0.01105	1	7:1519710	A	intron_vari	MODIFIER	KMT2C
87	0.03333	0.03278	1	10:219012	T	intron_vari	MODIFIER	MLLT10
87	0.03333	0.03278	1	10:219012	T	intron_vari	MODIFIER	MLLT10
89	0.01111	0.01105	1	10:220220	T	intron_vari	MODIFIER	MLLT10
89	0.01111	0.01105	1	1:4380350	T	5_prime_U	MODIFIER	MPL
89	0.01111	0.01105	1	1:4381848(	T	downstrea	MODIFIER	MPL
89	0.01111	0.01105	1	11:942240	G	missense_v	MODERATE	MRE11A
89	0.01111	0.01105	1	2:4763958(	T	stop_gaine	HIGH	MSH2
89	0.01111	0.01105	1	2:4764157(	G	intron_vari	MODIFIER	MSH2
87	0.02222	0.04346	0.03342	2:4764350	C	missense_v	MODERATE	MSH2
89	0.01111	0.01105	1	2:4770545	-	inframe_de	MODERATE	MSH2
89	0.01111	0.01105	1	1:1116750	C	3_prime_U	MODIFIER	MTOR
85	0.05556	0.05401	1	1:2369668	TT	missense_v	MODERATE	MTR
89	0.01111	0.01105	1	1:2370162	A	splice_regi	LOW	MTR
89	0.01111	0.01105	1	1:2370380	T	intron_vari	MODIFIER	MTR
89	0.01111	0.01105	1	1:2370587	G	missense_v	MODERATE	MTR
89	0	0.02198	0.005587	1:1551600	GAC	splice_acce	HIGH	MUC1
89	0.01111	0.01105	1	1:1551607(	G	synonymou	LOW	MUC1
89	0	0.02198	0.005587	1:4579918	T	stop_gaine	HIGH	MUTYH
89	0	0.02198	0.005587	8:1287530(	TGA	stop_gaine	HIGH	MYC
89	0.01111	0.01105	1	8:1287532(	T	3_prime_U	MODIFIER	MYC

89	0.01111	0.01105	1	1:4036360!	A	missense_\	MODERATI	MYCL
89	0.01111	0.01105	1	2:1608604:	A	synonymou	LOW	MYCN
89	0.01111	0.01105	1	16:158088(	G	missense_\	MODERATI	MYH11
89	0.01111	0.01105	1	16:158265'	A	splice_regi	LOW	MYH11
89	0.01111	0.01105	1	22:366901:	CC	intron_vari	MODIFIER	MYH9
89	0.01111	0.01105	1	8:7104101:	A	synonymou	LOW	NCOA2
89	0.01111	0.01105	1	8:7104120:	C	missense_\	MODERATI	NCOA2
89	0.01111	0.01105	1	8:7107566:	G	intron_vari	MODIFIER	NCOA2
89	0	0.02198	0.005587	8:7107577!	C	missense_\	MODERATI	NCOA2
89	0.01111	0.01105	1	8:7112891'	G	missense_\	MODERATI	NCOA2
88	0.02222	0.02198	1	10:515849:	A	missense_\	MODERATI	NCOA4
89	0.01111	0.01105	1	10:515864!	ATAT	intron_vari	MODIFIER	NCOA4
89	0.01111	0.01105	1	17:295276:	A	intron_vari	MODIFIER	NF1
88	0.02222	0.02198	1	17:295630'	TTG	intron_vari	MODIFIER	NF1
89	0.01111	0.01105	1	17:296840:	G	missense_\	MODERATI	NF1
89	0.01111	0.01105	1	2:1780962!	G	missense_\	MODERATI	NFE2L2
89	0.01111	0.01105	1	4:1035332,	G	missense_\	MODERATI	NFKB1
89	0.01111	0.01105	1	4:1035332!	A	intron_vari	MODIFIER	NFKB1
89	0.01111	0.01105	1	4:1035336!	A	missense_\	MODERATI	NFKB1
89	0.01111	0.01105	1	4:1035377,	T	missense_\	MODERATI	NFKB1
89	0.01111	0.01105	1	10:104156:	A	intron_vari	MODIFIER	NFKB2
89	0	0.02198	0.005587	14:512213:	G	missense_\	MODERATI	NIN
89	0.01111	0.01105	1	14:512253:	C	missense_\	MODERATI	NIN
89	0.01111	0.01105	1	17:548720,	A	missense_\	MODERATI	NLRP1
89	0.01111	0.01105	1	1:1204779:	A	intron_vari	MODIFIER	NOTCH2
89	0.01111	0.01105	1	1:1205396!	C	missense_\	MODERATI	NOTCH2
89	0.01111	0.01105	1	6:3216410'	A	synonymou	LOW	NOTCH4
89	0.01111	0.01105	1	6:3216673,	A	missense_\	MODERATI	NOTCH4
88	0.02222	0.02198	1	5:1766372(	C	missense_\	MODERATI	NSD1
89	0.01111	0.01105	1	1:1568119:	C	missense_\	MODERATI	INSRR
89	0.01111	0.01105	1	1:1568414!	A	missense_\	MODERATI	NTRK1
89	0.01111	0.01105	1	15:886695'	A	missense_\	MODERATI	NTRK3
89	0	0.02198	0.005587	9:1340906'	G	synonymou	LOW	NUP214
89	0.01111	0.01105	1	11:379485:	A	intron_vari	MODIFIER	NUP98
35	0	0	1	X:1104635,	T	intron_vari	MODIFIER	PAK3
89	0.01111	0.01105	1	9:3700654:	A	splice_regi	LOW	PAX5
89	0.01111	0.01105	1	1:1901842:	T	missense_\	MODERATI	PAX7
89	0.01111	0.01105	1	3:5261060:	C	missense_\	MODERATI	PBRM1
89	0.01111	0.01105	1	3:5261061!	G	missense_\	MODERATI	PBRM1
83	0.06667	0.08494	0.1499	1:1448523:	T	3_prime_U	MODIFIER	PDE4DIP
85	0.05556	0.05401	1	1:1448599:	T	missense_\	MODERATI	PDE4DIP
86	0.04444	0.04346	1	1:1448633(	C	missense_\	MODERATI	PDE4DIP
89	0.01111	0.01105	1	1:1448633(	A	synonymou	LOW	PDE4DIP
89	0.01111	0.01105	1	1:1448633:	A	stop_gaine	HIGH	PDE4DIP
89	0.01111	0.01105	1	1:1448633:	TT	missense_\	MODERATI	PDE4DIP
89	0.01111	0.01105	1	1:1448666(	T	missense_\	MODERATI	PDE4DIP
88	0.02222	0.02198	1	1:1448666'	A	missense_\	MODERATI	PDE4DIP

86	0.04444	0.04346	1	1:1448790	C	missense_	MODERAT	PDE4DIP
89	0.01111	0.01105	1	1:1448790	-	frameshift_	HIGH	PDE4DIP
85	0.05556	0.05401	1	1:1448814	T	missense_	MODERAT	PDE4DIP
81	0.1	0.095	1	1:1448815	A	missense_	MODERAT	PDE4DIP
24	0.7333	0.4644	2.399e-00	1:1448860	T	splice_regi	LOW	PDE4DIP
86	0.04444	0.04346	1	1:1448862	T	synonymou	LOW	PDE4DIP
89	0.01111	0.01105	1	1:1449237	C	missense_	MODERAT	PDE4DIP
14	0.8444	0.4879	8.431e-014	1:1449305	A	intron_vari	MODIFIER	PDE4DIP
88	0.02222	0.02198	1	1:1449309	C	intron_vari	MODIFIER	PDE4DIP
89	0.01111	0.01105	1	1:1449314	C	intron_vari	MODIFIER	PDE4DIP
87	0.03333	0.03278	1	1:1449946	T	missense_	MODERAT	PDE4DIP
84	0.06667	0.06444	1	1:1449946	A	missense_	MODERAT	PDE4DIP
89	0.01111	0.01105	1	1:1449946	C	missense_	MODERAT	PDE4DIP
89	0	0.02198	0.005587	4:5512989	C	synonymou	LOW	PDGFRA
88	0	0.04346	9.469e-005	4:5512989	G	missense_	MODERAT	PDGFRA
70	0.2222	0.1975	0.5916	5:1494995	T	intron_vari	MODIFIER	PDGFRB
89	0.01111	0.01105	1	5:1495144	A	missense_	MODERAT	PDGFRB
89	0.01111	0.01105	1	17:804556	GC	intron_vari	MODIFIER	PER1
89	0.01111	0.01105	1	1:2044388	G	missense_	MODERAT	PIK3C2B
89	0.01111	0.01105	1	3:1789169	A	intron_vari	MODIFIER	PIK3CA
89	0.01111	0.01105	1	3:1789222	AA	intron_vari	MODIFIER	PIK3CA
89	0.01111	0.01105	1	3:1384615	G	missense_	MODERAT	PIK3CB
89	0.01111	0.01105	1	7:1065455	G	intron_vari	MODIFIER	PIK3CG
89	0.01111	0.01105	1	5:6752272	T	stop_gaine	HIGH	PIK3R1
89	0.01111	0.01105	1	5:6759049	T	stop_gaine	HIGH	PIK3R1
89	0.01111	0.01105	1	6:5152465	T	missense_	MODERAT	PKHD1
89	0.01111	0.01105	1	6:5161305	T	missense_	MODERAT	PKHD1
89	0.01111	0.01105	1	6:5188945	A	stop_gaine	HIGH	PKHD1
89	0.01111	0.01105	1	6:5189049	A	missense_	MODERAT	PKHD1
88	0	0.04346	9.469e-005	6:5192181	GCCTT	intron_vari	MODIFIER	PKHD1
89	0.01111	0.01105	1	6:5192312	T	missense_	MODERAT	PKHD1
89	0.01111	0.01105	1	2:1907193	C	synonymou	LOW	PMS1
89	0.01111	0.01105	1	2:1907197	A	missense_	MODERAT	PMS1
89	0.01111	0.01105	1	7:6027040	C	synonymou	LOW	PMS2
88	0.02222	0.02198	1	7:6029511	T	stop_gaine	HIGH	PMS2
89	0.01111	0.01105	1	7:6035156	G	intron_vari	MODIFIER	PMS2
89	0.01111	0.01105	1	7:1244754	G	intron_vari	MODIFIER	POT1
89	0	0.02198	0.005587	17:665115	G	splice_regi	LOW	PRKAR1A
89	0.01111	0.01105	1	8:4868692	C	synonymou	LOW	PRKDC
89	0.01111	0.01105	1	8:4873649	G	missense_	MODERAT	PRKDC
89	0.01111	0.01105	1	8:4877604	G	synonymou	LOW	PRKDC
89	0.01111	0.01105	1	8:4882502	C	synonymou	LOW	PRKDC
89	0.01111	0.01105	1	8:4882803	C	intron_vari	MODIFIER	PRKDC
89	0.01111	0.01105	1	9:9821221	T	missense_	MODERAT	PTCH1
89	0.01111	0.01105	1	9:9824125	C	intron_vari	MODIFIER	PTCH1
89	0.01111	0.01105	1	1:1866469	A	intron_vari	MODIFIER	PTGS2
89	0.01111	0.01105	1	1:1866475	C	splice_regi	LOW	PTGS2

89	0.01111	0.01105	1	12:112910:	A	frameshift_	HIGH	PTPN11
89	0	0.02198	0.005587	9:8460646	-	intron_vari	MODIFIER	PTPRD
89	0.01111	0.01105	1	9:8485904	T	synonymou	LOW	PTPRD
89	0.01111	0.01105	1	9:8485984	T	missense_\	MODERATI	PTPRD
89	0	0.02198	0.005587	20:407438:	G	intron_vari	MODIFIER	PTPRT
89	0.01111	0.01105	1	5:1319244	A	missense_\	MODERATI	RAD50
89	0.01111	0.01105	1	3:1262640	T	synonymou	LOW	RAF1
89	0.01111	0.01105	1	3:1262673	A	stop_gaine	HIGH	RAF1
89	0.01111	0.01105	1	3:1264134	GG	intron_vari	MODIFIER	RAF1
89	0.01111	0.01105	1	9:1359813	T	missense_\	MODERATI	RALGDS
89	0.01111	0.01105	1	17:385047	C	splice_regi	LOW	RARA
89	0.01111	0.01105	1	8:1457373	A	downstrea	MODIFIER	GPT
88	0.02222	0.02198	1	2:6114777	-	frameshift_	HIGH	REL
88	0.02222	0.02198	1	2:6114777	G	missense_\	MODERATI	REL
88	0.02222	0.02198	1	1:1850566	G	intron_vari	MODIFIER	RNF2
89	0.01111	0.01105	1	1:1850671	-	intron_vari	MODIFIER	RNF2
89	0.01111	0.01105	1	17:782620	T	missense_\	MODERATI	RNF213
89	0.01111	0.01105	1	17:782643	G	intron_vari	MODIFIER	RNF213
89	0.01111	0.01105	1	17:782687	T	missense_\	MODERATI	RNF213
89	0.01111	0.01105	1	17:783191	T	stop_gaine	HIGH	RNF213
88	0.02222	0.02198	1	17:783516	T	intron_vari	MODIFIER	RNF213
89	0.01111	0.01105	1	17:783575	C	frameshift_	HIGH	RNF213
89	0.01111	0.01105	1	6:1177106	G	missense_\	MODERATI	ROS1
89	0.01111	0.01105	1	6:1669521	T	intron_vari	MODIFIER	RPS6KA2
89	0	0.02198	0.005587	8:9297266	C	missense_\	MODERATI	RUNX1T1
89	0.01111	0.01105	1	8:9302695	A	missense_\	MODERATI	RUNX1T1
89	0.01111	0.01105	1	8:9302948	A	missense_\	MODERATI	RUNX1T1
87	0.03333	0.03278	1	5:233504-2	C	intron_vari	MODIFIER	SDHA
89	0.01111	0.01105	1	3:4715548	A	missense_\	MODERATI	SETD2
89	0.01111	0.01105	1	3:4716399	G	missense_\	MODERATI	SETD2
89	0.01111	0.01105	1	2:1982627	G	synonymou	LOW	SF3B1
89	0.01111	0.01105	1	6:1344987	T	intron_vari	MODIFIER	SGK1
89	0.01111	0.01105	1	6:1345285	A	missense_\	MODERATI	SGK1
89	0.01111	0.01105	1	18:485752	T	missense_\	MODERATI	SMAD4
89	0.01111	0.01105	1	18:485934	C	synonymou	LOW	SMAD4
89	0.01111	0.01105	1	19:111705	T	synonymou	LOW	SMARCA4
87	0	0.06444	2.705e-006	22:241456	C	intron_vari	MODIFIER	SMARCB1
84	0.06667	0.06444	1	22:241676	C	intron_vari	MODIFIER	SMARCB1
89	0	0.02198	0.005587	2:5833815	T	frameshift_	HIGH	SOX11
89	0.01111	0.01105	1	3:1814308	C	missense_\	MODERATI	SOX2
89	0.01111	0.01105	1	20:360313	T	intron_vari	MODIFIER	SRC
88	0.02222	0.02198	1	20:360317	G	synonymou	LOW	SRC
89	0.01111	0.01105	1	2:2195448	T	missense_\	MODERATI	STK36
89	0.01111	0.01105	1	10:104357	T	synonymou	LOW	SUFU
89	0.01111	0.01105	1	9:9360631	A	missense_\	MODERATI	SYK
89	0.01111	0.01105	1	6:1524579	T	synonymou	LOW	SYNE1
89	0.01111	0.01105	1	6:1524707	T	stop_gaine	HIGH	SYNE1

89	0.01111	0.01105	1	6:1524728	C	missense_v	MODERATE	SYNE1
89	0.01111	0.01105	1	6:1525292	A	synonymou	LOW	SYNE1
89	0.01111	0.01105	1	6:1525426	C	missense_v	MODERATE	SYNE1
89	0.01111	0.01105	1	6:1526315	A	missense_v	MODERATE	SYNE1
89	0.01111	0.01105	1	6:1526319	A	synonymou	LOW	SYNE1
89	0.01111	0.01105	1	6:1526392	A	missense_v	MODERATE	SYNE1
89	0.01111	0.01105	1	6:1526446	A	synonymou	LOW	SYNE1
89	0.01111	0.01105	1	6:1526521	C	missense_v	MODERATE	SYNE1
89	0.01111	0.01105	1	6:1526747	C	missense_v	MODERATE	SYNE1
89	0.01111	0.01105	1	6:1527126	A	missense_v	MODERATE	SYNE1
89	0.01111	0.01105	1	6:1528238	T	intron_vari	MODIFIER	SYNE1
34	0.02857	0.02816	1	X:7059757	C	missense_v	MODERATE	TAF1
35	0	0	1	X:7068379	T	missense_v	MODERATE	TAF1
89	0.01111	0.01105	1	9:3263174	A	missense_v	MODERATE	TAF1L
34	0.02857	0.02816	1	X:7927960	C	missense_v	MODERATE	TBX22
88	0.02222	0.02198	1	19:164632	CGAG	intron_vari	MODIFIER	TCF3
89	0.01111	0.01105	1	2:8553477	-	frameshift_	HIGH	TCF7L1
89	0.01111	0.01105	1	10:114710	T	start_lost	HIGH	TCF7L2
89	0.01111	0.01105	1	10:114911	T	missense_v	MODERATE	TCF7L2
89	0.01111	0.01105	1	10:703323	G	missense_v	MODERATE	TET1
89	0	0.02198	0.005587	10:704063	A	synonymou	LOW	TET1
89	0.01111	0.01105	1	10:704515	A	missense_v	MODERATE	TET1
89	0.01111	0.01105	1	4:1061580	T	missense_v	MODERATE	TET2
89	0.01111	0.01105	1	4:1061938	-	frameshift_	HIGH	TET2
34	0.02857	0.02816	1	X:4889687	T	missense_v	MODERATE	TFE3
88	0.02222	0.02198	1	15:398848	T	missense_v	MODERATE	THBS1
89	0.01111	0.01105	1	17:757963	-	intron_vari	MODIFIER	TP53
89	0.01111	0.01105	1	1:1863014	T	missense_v	MODERATE	TPR
89	0.01111	0.01105	1	1:1863045	G	synonymou	LOW	TPR
88	0.02222	0.02198	1	1:1863152	T	intron_vari	MODIFIER	TPR
89	0.01111	0.01105	1	1:1149403	A	synonymou	LOW	TRIM33
89	0.01111	0.01105	1	1:1149762	T	splice_regi	LOW	TRIM33
89	0.01111	0.01105	1	14:924547	T	intron_vari	MODIFIER	TRIP11
88	0.02222	0.02198	1	14:924619	T	intron_vari	MODIFIER	TRIP11
89	0.01111	0.01105	1	14:924718	T	missense_v	MODERATE	TRIP11
89	0.01111	0.01105	1	7:9854332	G	intron_vari	MODIFIER	TRRAP
89	0.01111	0.01105	1	7:9855275	C	missense_v	MODERATE	TRRAP
89	0.01111	0.01105	1	7:9855276	T	stop_gaine	HIGH	TRRAP
89	0.01111	0.01105	1	7:9855277	T	missense_v	MODERATE	TRRAP
89	0.01111	0.01105	1	7:9857935	T	intron_vari	MODIFIER	TRRAP
88	0	0.04346	9.469e-005	16:212945	T	intron_vari	MODIFIER	TSC2
89	0.01111	0.01105	1	14:815542	C	intron_vari	MODIFIER	TSHR
89	0.01111	0.01105	1	14:815543	G	missense_v	MODERATE	TSHR
89	0.01111	0.01105	1	14:816060	A	synonymou	LOW	TSHR
89	0.01111	0.01105	1	8:1032849	T	missense_v	MODERATE	UBR5
89	0.01111	0.01105	1	8:1032893	C	missense_v	MODERATE	UBR5
89	0.01111	0.01105	1	8:1032974	G	synonymou	LOW	UBR5

89	0.01111	0.01105	1	8:1033074(	C	missense_\	MODERATI	UBR5
88	0.02222	0.02198	1	8:1033414!	-	intron_vari	MODIFIER	UBR5
89	0.01111	0.01105	1	8:1033548(	C	synonymou	LOW	UBR5
34	0.02857	0.02816	1	X:4108872:	T	intron_vari	MODIFIER	USP9X
89	0.01111	0.01105	1	4:1961342·	T	stop_gaine	HIGH	WHSC1
86	0	0.08494	1.095e-007	4:1976481·	G	intron_vari	MODIFIER	WHSC1
87	0	0.06444	2.705e-006	4:1976483·	G	intron_vari	MODIFIER	WHSC1
89	0.01111	0.01105	1	4:1976485·	GG	intron_vari	MODIFIER	WHSC1
89	0.01111	0.01105	1	4:1976486·	G	intron_vari	MODIFIER	WHSC1
89	0.01111	0.01105	1	8:3093875:	-	frameshift_	HIGH	WRN
89	0.01111	0.01105	1	8:3098986!	C	intron_vari	MODIFIER	WRN
89	0.01111	0.01105	1	8:3100483·	A	intron_vari	MODIFIER	WRN
89	0.01111	0.01105	1	8:3102468·	T	missense_\	MODERATI	WRN
84	0	0.1244	3.75e-010	12:677711(	-	frameshift_	HIGH	ZNF384
89	0.01111	0.01105	1	12:678824:	C	synonymou	LOW	ZNF384

Gene	Feature_ty	Feature	BIOTYPE	EXON	INTRON	HGVSc	HGVSp	cDNA_posi
ENSG0000(	Transcript	ENST0000C	protein_co	5/11	-	ENST0000C	ENSP0000C	1222
ENSG0000(	Transcript	ENST0000C	protein_co	-	13/38	ENST0000C	-	-
ENSG0000(	Transcript	ENST0000C	protein_co	-	2/20	ENST0000C	-	-
ENSG0000(	Transcript	ENST0000C	protein_co	4/21	-	ENST0000C	ENSP0000C	524
ENSG0000(	Transcript	ENST0000C	protein_co	8/50	-	ENST0000C	ENSP0000C	2236
ENSG0000(	Transcript	ENST0000C	protein_co	-	8/49	ENST0000C	-	-
ENSG0000(	Transcript	ENST0000C	protein_co	18/50	-	ENST0000C	ENSP0000C	5002
ENSG0000(	Transcript	ENST0000C	protein_co	-	20/49	ENST0000C	-	-
ENSG0000(	Transcript	ENST0000C	protein_co	27/50	-	ENST0000C	ENSP0000C	6591-6592
ENSG0000(	Transcript	ENST0000C	protein_co	31/50	-	ENST0000C	ENSP0000C	7942
ENSG0000(	Transcript	ENST0000C	protein_co	33/50	-	ENST0000C	ENSP0000C	8638
ENSG0000(	Transcript	ENST0000C	protein_co	12/14	-	ENST0000C	ENSP0000C	1488
ENSG0000(	Transcript	ENST0000C	protein_co	-	9/13	ENST0000C	-	-
ENSG0000(	Transcript	ENST0000C	protein_co	10/29	-	ENST0000C	ENSP0000C	2782
ENSG0000(	Transcript	ENST0000C	protein_co	14/16	-	ENST0000C	ENSP0000C	2076
ENSG0000(	Transcript	ENST0000C	protein_co	14/16	-	ENST0000C	ENSP0000C	2077
ENSG0000(	Transcript	ENST0000C	protein_co	16/16	-	ENST0000C	ENSP0000C	3060-3061
ENSG0000(	Transcript	ENST0000C	protein_co	16/16	-	ENST0000C	ENSP0000C	4297
ENSG0000(	Transcript	ENST0000C	protein_co	16/16	-	ENST0000C	ENSP0000C	4700-4721
ENSG0000(	Transcript	ENST0000C	protein_co	8/8	-	ENST0000C	ENSP0000C	3205
ENSG0000(	Transcript	ENST0000C	protein_co	9/20	-	ENST0000C	ENSP0000C	3212
ENSG0000(	Transcript	ENST0000C	protein_co	9/20	-	ENST0000C	ENSP0000C	3214
ENSG0000(	Transcript	ENST0000C	protein_co	12/20	-	ENST0000C	ENSP0000C	3659
ENSG0000(	Transcript	ENST0000C	protein_co	13/20	-	ENST0000C	ENSP0000C	3797
ENSG0000(	Transcript	ENST0000C	protein_co	20/20	-	ENST0000C	ENSP0000C	6325
ENSG0000(	Transcript	ENST0000C	protein_co	17/21	-	ENST0000C	ENSP0000C	5199
ENSG0000(	Transcript	ENST0000C	protein_co	-	6/21	ENST0000C	-	-
ENSG0000(	Transcript	ENST0000C	protein_co	-	8/62	ENST0000C	-	-
ENSG0000(	Transcript	ENST0000C	protein_co	-	37/62	ENST0000C	-	-
ENSG0000(	Transcript	ENST0000C	protein_co	50/63	-	ENST0000C	ENSP0000C	7762
ENSG0000(	Transcript	ENST0000C	protein_co	-	44/46	ENST0000C	-	-
ENSG0000(	Transcript	ENST0000C	protein_co	-	44/46	ENST0000C	-	-
ENSG0000(	Transcript	ENST0000C	protein_co	39/47	-	ENST0000C	ENSP0000C	6740
ENSG0000(	Transcript	ENST0000C	protein_co	14/47	-	ENST0000C	ENSP0000C	2960
ENSG0000(	Transcript	ENST0000C	protein_co	-	11/46	ENST0000C	-	-
ENSG0000(	Transcript	ENST0000C	protein_co	-	10/46	ENST0000C	-	-
ENSG0000(	Transcript	ENST0000C	protein_co	-	26/34	ENST0000C	-	-
ENSG0000(	Transcript	ENST0000C	protein_co	6/11	-	ENST0000C	ENSP0000C	917-918
ENSG0000(	Transcript	ENST0000C	protein_co	7/9	-	ENST0000C	ENSP0000C	718
ENSG0000(	Transcript	ENST0000C	protein_co	-	4/6	ENST0000C	-	-
ENSG0000(	Transcript	ENST0000C	protein_co	3/32	-	ENST0000C	ENSP0000C	1446
ENSG0000(	Transcript	ENST0000C	protein_co	29/32	-	ENST0000C	ENSP0000C	4899
ENSG0000(	Transcript	ENST0000C	protein_co	3/3	-	ENST0000C	ENSP0000C	1380
ENSG0000(	Transcript	ENST0000C	protein_co	-	1/2	ENST0000C	-	-
ENSG0000(	Transcript	ENST0000C	protein_co	-	2/3	ENST0000C	-	-

ENSG0000(	Transcript	ENST0000C	protein_co	5/10	-	ENST0000C	ENSP0000C	1541
ENSG0000(	Transcript	ENST0000C	protein_co	8/10	-	ENST0000C	ENSP0000C	1476
ENSG0000(	Transcript	ENST0000C	protein_co	-	8/9	ENST0000C	-	-
ENSG0000(	Transcript	ENST0000C	protein_co	2/9	-	ENST0000C	ENSP0000C	2803
ENSG0000(	Transcript	ENST0000C	protein_co	2/9	-	ENST0000C	ENSP0000C	3226
ENSG0000(	Transcript	ENST0000C	protein_co	6/9	-	ENST0000C	ENSP0000C	3992
ENSG0000(	Transcript	ENST0000C	protein_co	9/9	-	ENST0000C	ENSP0000C	4377
ENSG0000(	Transcript	ENST0000C	protein_co	9/9	-	ENST0000C	ENSP0000C	4385
ENSG0000(	Transcript	ENST0000C	protein_co	1/5	-	ENST0000C	-	123
ENSG0000(	Transcript	ENST0000C	protein_co	5/5	-	ENST0000C	-	639
ENSG0000(	Transcript	ENST0000C	protein_co	7/22	-	ENST0000C	ENSP0000C	1660
ENSG0000(	Transcript	ENST0000C	protein_co	19/22	-	ENST0000C	ENSP0000C	3773
ENSG0000(	Transcript	ENST0000C	protein_co	-	16-Jul	ENST0000C	-	-
ENSG0000(	Transcript	ENST0000C	protein_co	20/20	-	ENST0000C	-	4027
ENSG0000(	Transcript	ENST0000C	protein_co	-	12/19	ENST0000C	-	-
ENSG0000(	Transcript	ENST0000C	protein_co	-	11/19	ENST0000C	-	-
ENSG0000(	Transcript	ENST0000C	protein_co	13/25	-	ENST0000C	ENSP0000C	2121
ENSG0000(	Transcript	ENST0000C	protein_co	11/27	-	ENST0000C	ENSP0000C	1890
ENSG0000(	Transcript	ENST0000C	protein_co	-	24/26	ENST0000C	-	-
ENSG0000(	Transcript	ENST0000C	protein_co	-	15-Apr	ENST0000C	-	-
ENSG0000(	Transcript	ENST0000C	protein_co	-	6/16	ENST0000C	-	-
ENSG0000(	Transcript	ENST0000C	protein_co	-	7/16	ENST0000C	-	-
ENSG0000(	Transcript	ENST0000C	protein_co	-	5/15	ENST0000C	-	-
ENSG0000(	Transcript	ENST0000C	protein_co	-	4/12	ENST0000C	-	-
ENSG0000(	Transcript	ENST0000C	protein_co	-	4/15	ENST0000C	-	-
ENSG0000(	Transcript	ENST0000C	protein_co	3/16	-	ENST0000C	ENSP0000C	754
ENSG0000(	Transcript	ENST0000C	protein_co	12/12	-	ENST0000C	ENSP0000C	2752
ENSG0000(	Transcript	ENST0000C	protein_co	2/14	-	ENST0000C	ENSP0000C	1730
ENSG0000(	Transcript	ENST0000C	protein_co	-	6/7	ENST0000C	-	-
ENSG0000(	Transcript	ENST0000C	nonsense_	-	4/5	ENST0000C	-	-
ENSG0000(	Transcript	ENST0000C	protein_co	2/20	-	ENST0000C	ENSP0000C	247
ENSG0000(	Transcript	ENST0000C	protein_co	18/20	-	ENST0000C	ENSP0000C	4366
ENSG0000(	Transcript	ENST0000C	protein_co	-	Feb-50	ENST0000C	-	-
ENSG0000(	Transcript	ENST0000C	protein_co	10/11	-	ENST0000C	ENSP0000C	1040
ENSG0000(	Transcript	ENST0000C	protein_co	-	5/10	ENST0000C	-	-
ENSG0000(	Transcript	ENST0000C	protein_co	4/31	-	ENST0000C	ENSP0000C	1803
ENSG0000(	Transcript	ENST0000C	protein_co	-	65/70	ENST0000C	-	-
ENSG0000(	Transcript	ENST0000C	protein_co	-	56/70	ENST0000C	-	-
ENSG0000(	Transcript	ENST0000C	protein_co	-	49/70	ENST0000C	-	-
ENSG0000(	Transcript	ENST0000C	protein_co	-	29/70	ENST0000C	-	-
ENSG0000(	Transcript	ENST0000C	protein_co	-	25/70	ENST0000C	-	-
ENSG0000(	Transcript	ENST0000C	protein_co	16/18	-	ENST0000C	ENSP0000C	2287
ENSG0000(	Transcript	ENST0000C	protein_co	18/18	-	ENST0000C	ENSP0000C	2750-2752
ENSG0000(	Transcript	ENST0000C	protein_co	6/15	-	ENST0000C	ENSP0000C	1060
ENSG0000(	Transcript	ENST0000C	protein_co	5/9	-	ENST0000C	ENSP0000C	774
ENSG0000(	Transcript	ENST0000C	protein_co	4/9	-	ENST0000C	ENSP0000C	674
ENSG0000(	Transcript	ENST0000C	protein_co	5/29	-	ENST0000C	ENSP0000C	1503

ENSG0000(	Transcript	ENST0000C	protein_co	5/29	-	ENST0000C	ENSP0000(	1523
ENSG0000(	Transcript	ENST0000C	protein_co	21/29	-	ENST0000C	ENSP0000(	3779
ENSG0000(	Transcript	ENST0000C	protein_co	-	22/28	ENST0000C	-	-
ENSG0000(	Transcript	ENST0000C	protein_co	-	12/18	ENST0000C	-	-
ENSG0000(	Transcript	ENST0000C	protein_co	-	13/18	ENST0000C	-	-
ENSG0000(	Transcript	ENST0000C	protein_co	18/19	-	ENST0000C	ENSP0000(	2737
ENSG0000(	Transcript	ENST0000C	protein_co	23/29	-	ENST0000C	ENSP0000(	4132
ENSG0000(	Transcript	ENST0000C	protein_co	23/23	-	ENST0000C	ENSP0000(	3077
ENSG0000(	Transcript	ENST0000C	protein_co	3/23	-	ENST0000C	ENSP0000(	510
ENSG0000(	Transcript	ENST0000C	protein_co	-	21/22	ENST0000C	-	-
ENSG0000(	Transcript	ENST0000C	protein_co	-	7/22	ENST0000C	-	-
ENSG0000(	Transcript	ENST0000C	protein_co	-	3/22	ENST0000C	-	-
ENSG0000(	Transcript	ENST0000C	protein_co	1/23	-	ENST0000C	-	93
ENSG0000(	Transcript	ENST0000C	protein_co	73/84	-	ENST0000C	ENSP0000(	14043
ENSG0000(	Transcript	ENST0000C	protein_co	70/84	-	ENST0000C	ENSP0000(	13689
ENSG0000(	Transcript	ENST0000C	protein_co	69/84	-	ENST0000C	ENSP0000(	13498
ENSG0000(	Transcript	ENST0000C	protein_co	42/84	-	ENST0000C	ENSP0000(	8781
ENSG0000(	Transcript	ENST0000C	protein_co	42/84	-	ENST0000C	ENSP0000(	8481
ENSG0000(	Transcript	ENST0000C	protein_co	-	22/83	ENST0000C	-	-
ENSG0000(	Transcript	ENST0000C	protein_co	-	22/83	ENST0000C	-	-
ENSG0000(	Transcript	ENST0000C	protein_co	-	21/83	ENST0000C	-	-
ENSG0000(	Transcript	ENST0000C	protein_co	-	11/30	ENST0000C	-	-
ENSG0000(	Transcript	ENST0000C	protein_co	-	16/30	ENST0000C	-	-
ENSG0000(	Transcript	ENST0000C	protein_co	4/53	-	ENST0000C	ENSP0000(	1652
ENSG0000(	Transcript	ENST0000C	protein_co	14/53	-	ENST0000C	ENSP0000(	3188
ENSG0000(	Transcript	ENST0000C	protein_co	17/53	-	ENST0000C	ENSP0000(	3622
ENSG0000(	Transcript	ENST0000C	protein_co	-	36/52	ENST0000C	-	-
ENSG0000(	Transcript	ENST0000C	protein_co	41/53	-	ENST0000C	ENSP0000(	7479
ENSG0000(	Transcript	ENST0000C	protein_co	53/53	-	ENST0000C	ENSP0000(	9332
ENSG0000(	Transcript	ENST0000C	protein_co	-	6/16	ENST0000C	-	-
ENSG0000(	Transcript	ENST0000C	protein_co	-	9/16	ENST0000C	-	-
ENSG0000(	Transcript	ENST0000C	protein_co	10/17	-	ENST0000C	ENSP0000(	1991
ENSG0000(	Transcript	ENST0000C	protein_co	-	10/16	ENST0000C	-	-
ENSG0000(	Transcript	ENST0000C	protein_co	-	9/16	ENST0000C	-	-
ENSG0000(	Transcript	ENST0000C	protein_co	-	1/16	ENST0000C	-	-
ENSG0000(	Transcript	ENST0000C	protein_co	9/16	-	ENST0000C	ENSP0000(	2119
ENSG0000(	Transcript	ENST0000C	protein_co	-	8/26	ENST0000C	-	-
ENSG0000(	Transcript	ENST0000C	protein_co	-	9/26	ENST0000C	-	-
ENSG0000(	Transcript	ENST0000C	protein_co	26/27	-	ENST0000C	ENSP0000(	3451
ENSG0000(	Transcript	ENST0000C	protein_co	-	3/27	ENST0000C	-	-
ENSG0000(	Transcript	ENST0000C	protein_co	28/28	-	ENST0000C	ENSP0000(	3944
ENSG0000(	Transcript	ENST0000C	protein_co	-	17/27	ENST0000C	-	-
ENSG0000(	Transcript	ENST0000C	protein_co	-	2/27	ENST0000C	-	-
ENSG0000(	Transcript	ENST0000C	protein_co	4/12	-	ENST0000C	ENSP0000(	300
ENSG0000(	Transcript	ENST0000C	protein_co	-	9/13	ENST0000C	-	-
ENSG0000(	Transcript	ENST0000C	protein_co	7/13	-	ENST0000C	ENSP0000(	809
ENSG0000(	Transcript	ENST0000C	protein_co	8/11	-	ENST0000C	ENSP0000(	2494

ENSG0000(	Transcript	ENST0000C	protein_co	1/11	-	ENST0000C	ENSP0000C	1662
ENSG0000(	Transcript	ENST0000C	protein_co	-	10/13	ENST0000C	-	-
ENSG0000(	Transcript	ENST0000C	protein_co	-	8/19	ENST0000C	-	-
ENSG0000(	Transcript	ENST0000C	protein_co	2/2	-	ENST0000C	ENSP0000C	1800-1801
ENSG0000(	Transcript	ENST0000C	protein_co	-	14/42	ENST0000C	-	-
ENSG0000(	Transcript	ENST0000C	protein_co	-	4/42	ENST0000C	-	-
ENSG0000(	Transcript	ENST0000C	protein_co	15/43	-	ENST0000C	ENSP0000C	1259
ENSG0000(	Transcript	ENST0000C	protein_co	37/43	-	ENST0000C	ENSP0000C	3832
ENSG0000(	Transcript	ENST0000C	protein_co	-	58/63	ENST0000C	-	-
ENSG0000(	Transcript	ENST0000C	protein_co	12/12	-	ENST0000C	ENSP0000C	3310
ENSG0000(	Transcript	ENST0000C	protein_co	15/19	-	ENST0000C	ENSP0000C	2333
ENSG0000(	Transcript	ENST0000C	protein_co	15/18	-	ENST0000C	ENSP0000C	2634
ENSG0000(	Transcript	ENST0000C	protein_co	-	5/17	ENST0000C	-	-
ENSG0000(	Transcript	ENST0000C	protein_co	13/18	-	ENST0000C	ENSP0000C	1976
ENSG0000(	Transcript	ENST0000C	protein_co	10/18	-	ENST0000C	ENSP0000C	1553
ENSG0000(	Transcript	ENST0000C	protein_co	-	19/23	ENST0000C	-	-
ENSG0000(	Transcript	ENST0000C	protein_co	29/30	-	ENST0000C	ENSP0000C	3945
ENSG0000(	Transcript	ENST0000C	protein_co	-	42/45	ENST0000C	-	-
ENSG0000(	Transcript	ENST0000C	protein_co	40/46	-	ENST0000C	ENSP0000C	6632
ENSG0000(	Transcript	ENST0000C	protein_co	-	16/45	ENST0000C	-	-
ENSG0000(	Transcript	ENST0000C	protein_co	2/3	-	ENST0000C	ENSP0000C	1173
ENSG0000(	Transcript	ENST0000C	protein_co	2/3	-	ENST0000C	ENSP0000C	1565
ENSG0000(	Transcript	ENST0000C	protein_co	16-Jan	-	ENST0000C	ENSP0000C	280-281
ENSG0000(	Transcript	ENST0000C	protein_co	13/17	-	ENST0000C	ENSP0000C	1984
ENSG0000(	Transcript	ENST0000C	protein_co	-	8/12	ENST0000C	-	-
ENSG0000(	Transcript	ENST0000C	protein_co	4/6	-	ENST0000C	ENSP0000C	1292
ENSG0000(	Transcript	ENST0000C	protein_co	1/13	-	ENST0000C	ENSP0000C	669
ENSG0000(	Transcript	ENST0000C	protein_co	-	10/18	ENST0000C	-	-
ENSG0000(	Transcript	ENST0000C	protein_co	18/33	-	ENST0000C	ENSP0000C	3158
ENSG0000(	Transcript	ENST0000C	protein_co	8/9	-	ENST0000C	ENSP0000C	2424
ENSG0000(	Transcript	ENST0000C	protein_co	-	20/21	ENST0000C	-	-
ENSG0000(	Transcript	ENST0000C	protein_co	12/12	-	ENST0000C	ENSP0000C	2808
ENSG0000(	Transcript	ENST0000C	protein_co	-	10/11	ENST0000C	-	-
ENSG0000(	Transcript	ENST0000C	protein_co	9/11	-	ENST0000C	ENSP0000C	1282
ENSG0000(	Transcript	ENST0000C	protein_co	-	1/20	ENST0000C	-	-
ENSG0000(	Transcript	ENST0000C	protein_co	-	10/20	ENST0000C	-	-
ENSG0000(	Transcript	ENST0000C	protein_co	-	1/47	ENST0000C	-	-
ENSG0000(	Transcript	ENST0000C	protein_co	31/48	-	ENST0000C	ENSP0000C	4587
ENSG0000(	Transcript	ENST0000C	protein_co	-	12/21	ENST0000C	-	-
ENSG0000(	Transcript	ENST0000C	protein_co	-	3/21	ENST0000C	-	-
ENSG0000(	Transcript	ENST0000C	protein_co	17/17	-	ENST0000C	ENSP0000C	2888
ENSG0000(	Transcript	ENST0000C	protein_co	17/17	-	ENST0000C	ENSP0000C	2384
ENSG0000(	Transcript	ENST0000C	protein_co	-	14/16	ENST0000C	-	-
ENSG0000(	Transcript	ENST0000C	protein_co	8/17	-	ENST0000C	ENSP0000C	1175
ENSG0000(	Transcript	ENST0000C	protein_co	-	4/16	ENST0000C	-	-
ENSG0000(	Transcript	ENST0000C	protein_co	5/9	-	ENST0000C	ENSP0000C	666
ENSG0000(	Transcript	ENST0000C	protein_co	1/2	-	ENST0000C	ENSP0000C	4076

ENSG0000(	Transcript	ENST0000C	protein_co	4/30	-	ENST0000C	ENSP0000C	515
ENSG0000(	Transcript	ENST0000C	protein_co	-	4/29	ENST0000C	-	-
ENSG0000(	Transcript	ENST0000C	protein_co	7/30	-	ENST0000C	ENSP0000C	846
ENSG0000(	Transcript	ENST0000C	protein_co	13/30	-	ENST0000C	ENSP0000C	1715
ENSG0000(	Transcript	ENST0000C	protein_co	-	12/27	ENST0000C	-	-
ENSG0000(	Transcript	ENST0000C	protein_co	-	22/27	ENST0000C	-	-
ENSG0000(	Transcript	ENST0000C	protein_co	-	4/16	ENST0000C	-	-
ENSG0000(	Transcript	ENST0000C	protein_co	3/15	-	ENST0000C	ENSP0000C	327
ENSG0000(	Transcript	ENST0000C	protein_co	-	3/14	ENST0000C	-	-
ENSG0000(	Transcript	ENST0000C	protein_co	12/15	-	ENST0000C	ENSP0000C	1943
ENSG0000(	Transcript	ENST0000C	protein_co	12/15	-	ENST0000C	ENSP0000C	1967
ENSG0000(	Transcript	ENST0000C	protein_co	19/25	-	ENST0000C	ENSP0000C	2982-2983
ENSG0000(	Transcript	ENST0000C	protein_co	-	20/24	ENST0000C	-	-
ENSG0000(	Transcript	ENST0000C	protein_co	18/18	-	ENST0000C	ENSP0000C	5241-5242
ENSG0000(	Transcript	ENST0000C	protein_co	18-Aug	-	ENST0000C	ENSP0000C	1810
ENSG0000(	Transcript	ENST0000C	protein_co	8/18	-	ENST0000C	ENSP0000C	2398
ENSG0000(	Transcript	ENST0000C	protein_co	12/18	-	ENST0000C	ENSP0000C	2979
ENSG0000(	Transcript	ENST0000C	protein_co	12/18	-	ENST0000C	ENSP0000C	2983
ENSG0000(	Transcript	ENST0000C	protein_co	12/18	-	ENST0000C	ENSP0000C	2990
ENSG0000(	Transcript	ENST0000C	protein_co	7/26	-	ENST0000C	ENSP0000C	1414
ENSG0000(	Transcript	ENST0000C	protein_co	-	23/28	ENST0000C	-	-
ENSG0000(	Transcript	ENST0000C	protein_co	-	27/29	ENST0000C	-	-
ENSG0000(	Transcript	ENST0000C	protein_co	13/30	-	ENST0000C	ENSP0000C	2185
ENSG0000(	Transcript	ENST0000C	protein_co	6/30	-	ENST0000C	ENSP0000C	1094
ENSG0000(	Transcript	ENST0000C	protein_co	4/30	-	ENST0000C	ENSP0000C	724-725
ENSG0000(	Transcript	ENST0000C	protein_co	4/6	-	ENST0000C	ENSP0000C	1881
ENSG0000(	Transcript	ENST0000C	protein_co	2/4	-	ENST0000C	ENSP0000C	500
ENSG0000(	Transcript	ENST0000C	protein_co	2/6	-	ENST0000C	ENSP0000C	104-105
ENSG0000(	Transcript	ENST0000C	protein_co	20/20	-	ENST0000C	ENSP0000C	3275
ENSG0000(	Transcript	ENST0000C	protein_co	-	19/19	ENST0000C	-	-
ENSG0000(	Transcript	ENST0000C	protein_co	-	16/19	ENST0000C	-	-
ENSG0000(	Transcript	ENST0000C	protein_co	-	11/19	ENST0000C	-	-
ENSG0000(	Transcript	ENST0000C	protein_co	11/20	-	ENST0000C	ENSP0000C	1680
ENSG0000(	Transcript	ENST0000C	protein_co	7/25	-	ENST0000C	ENSP0000C	1351
ENSG0000(	Transcript	ENST0000C	protein_co	22/25	-	ENST0000C	ENSP0000C	3705
ENSG0000(	Transcript	ENST0000C	protein_co	-	82/90	ENST0000C	-	-
ENSG0000(	Transcript	ENST0000C	protein_co	-	64/90	ENST0000C	-	-
ENSG0000(	Transcript	ENST0000C	protein_co	62/91	-	ENST0000C	ENSP0000C	10890
ENSG0000(	Transcript	ENST0000C	protein_co	-	59/90	ENST0000C	-	-
ENSG0000(	Transcript	ENST0000C	protein_co	44/91	-	ENST0000C	ENSP0000C	8357
ENSG0000(	Transcript	ENST0000C	protein_co	30/91	-	ENST0000C	ENSP0000C	5981
ENSG0000(	Transcript	ENST0000C	protein_co	-	28/90	ENST0000C	-	-
ENSG0000(	Transcript	ENST0000C	protein_co	21/91	-	ENST0000C	ENSP0000C	4180
ENSG0000(	Transcript	ENST0000C	protein_co	8/91	-	ENST0000C	ENSP0000C	2085
ENSG0000(	Transcript	ENST0000C	protein_co	-	6/90	ENST0000C	-	-
ENSG0000(	Transcript	ENST0000C	protein_co	-	4/90	ENST0000C	-	-
ENSG0000(	Transcript	ENST0000C	protein_co	-	1/90	ENST0000C	-	-

ENSG0000(	Transcript	ENST0000C	protein_co	-	1/16	ENST0000C	-	-
ENSG0000(	Transcript	ENST0000C	protein_co	14/23	-	ENST0000C	ENSP0000C	2307
ENSG0000(	Transcript	ENST0000C	protein_co	3/5	-	ENST0000C	ENSP0000C	3467
ENSG0000(	Transcript	ENST0000C	protein_co	2/5	-	ENST0000C	ENSP0000C	2254
ENSG0000(	Transcript	ENST0000C	protein_co	2/5	-	ENST0000C	ENSP0000C	2090
ENSG0000(	Transcript	ENST0000C	protein_co	10/11	-	ENST0000C	ENSP0000C	1571
ENSG0000(	Transcript	ENST0000C	protein_co	-	11/17	ENST0000C	-	-
ENSG0000(	Transcript	ENST0000C	protein_co	17/17	-	ENST0000C	ENSP0000C	2517
ENSG0000(	Transcript	ENST0000C	protein_co	-	-	-	-	-
ENSG0000(	Transcript	ENST0000C	protein_co	-	10/15	ENST0000C	-	-
ENSG0000(	Transcript	ENST0000C	protein_co	2/3	-	ENST0000C	ENSP0000C	994
ENSG0000(	Transcript	ENST0000C	protein_co	10/10	-	ENST0000C	ENSP0000C	2085
ENSG0000(	Transcript	ENST0000C	protein_co	2/21	-	ENST0000C	ENSP0000C	916
ENSG0000(	Transcript	ENST0000C	protein_co	8/21	-	ENST0000C	ENSP0000C	2178
ENSG0000(	Transcript	ENST0000C	protein_co	-	2/9	ENST0000C	-	-
ENSG0000(	Transcript	ENST0000C	protein_co	-	6/9	ENST0000C	-	-
ENSG0000(	Transcript	ENST0000C	protein_co	17/19	-	ENST0000C	ENSP0000C	2152
ENSG0000(	Transcript	ENST0000C	protein_co	27/36	-	ENST0000C	ENSP0000C	9594
ENSG0000(	Transcript	ENST0000C	protein_co	32/36	-	ENST0000C	ENSP0000C	11328
ENSG0000(	Transcript	ENST0000C	protein_co	-	32/53	ENST0000C	-	-
ENSG0000(	Transcript	ENST0000C	protein_co	43/59	-	ENST0000C	ENSP0000C	11226
ENSG0000(	Transcript	ENST0000C	protein_co	36/59	-	ENST0000C	ENSP0000C	5920
ENSG0000(	Transcript	ENST0000C	protein_co	-	25/58	ENST0000C	-	-
ENSG0000(	Transcript	ENST0000C	protein_co	-	25/58	ENST0000C	-	-
ENSG0000(	Transcript	ENST0000C	protein_co	-	25/58	ENST0000C	-	-
ENSG0000(	Transcript	ENST0000C	protein_co	12/59	-	ENST0000C	ENSP0000C	1951
ENSG0000(	Transcript	ENST0000C	protein_co	-	6/58	ENST0000C	-	-
ENSG0000(	Transcript	ENST0000C	protein_co	-	22-May	ENST0000C	-	-
ENSG0000(	Transcript	ENST0000C	protein_co	-	5/22	ENST0000C	-	-
ENSG0000(	Transcript	ENST0000C	protein_co	-	18/22	ENST0000C	-	-
ENSG0000(	Transcript	ENST0000C	protein_co	1/12	-	ENST0000C	-	25
ENSG0000(	Transcript	ENST0000C	protein_co	-	-	-	-	-
ENSG0000(	Transcript	ENST0000C	protein_co	3/20	-	ENST0000C	ENSP0000C	349
ENSG0000(	Transcript	ENST0000C	protein_co	4/16	-	ENST0000C	ENSP0000C	902
ENSG0000(	Transcript	ENST0000C	protein_co	-	5/15	ENST0000C	-	-
ENSG0000(	Transcript	ENST0000C	protein_co	6/16	-	ENST0000C	ENSP0000C	1233
ENSG0000(	Transcript	ENST0000C	protein_co	14/16	-	ENST0000C	ENSP0000C	2483-2488
ENSG0000(	Transcript	ENST0000C	protein_co	58/58	-	ENST0000C	-	7767
ENSG0000(	Transcript	ENST0000C	protein_co	2/33	-	ENST0000C	ENSP0000C	522-523
ENSG0000(	Transcript	ENST0000C	protein_co	-	17/32	ENST0000C	-	-
ENSG0000(	Transcript	ENST0000C	protein_co	-	23/32	ENST0000C	-	-
ENSG0000(	Transcript	ENST0000C	protein_co	31/33	-	ENST0000C	ENSP0000C	3861
ENSG0000(	Transcript	ENST0000C	protein_co	6/8	5/7	ENST0000C	-	?-1154
ENSG0000(	Transcript	ENST0000C	protein_co	3/8	-	ENST0000C	ENSP0000C	831
ENSG0000(	Transcript	ENST0000C	protein_co	3/16	-	ENST0000C	ENSP0000C	377
ENSG0000(	Transcript	ENST0000C	protein_co	3/3	-	ENST0000C	ENSP0000C	1671-1672
ENSG0000(	Transcript	ENST0000C	protein_co	3/3	-	ENST0000C	-	1931

ENSG0000(	Transcript	ENST0000C	protein_co	3/3	-	ENST0000C	ENSP0000(	749
ENSG0000(	Transcript	ENST0000C	protein_co	3/3	-	ENST0000C	ENSP0000(	1521
ENSG0000(	Transcript	ENST0000C	protein_co	41/42	-	ENST0000C	ENSP0000(	5796
ENSG0000(	Transcript	ENST0000C	protein_co	-	27/41	ENST0000C	-	-
ENSG0000(	Transcript	ENST0000C	protein_co	-	28/40	ENST0000C	-	-
ENSG0000(	Transcript	ENST0000C	protein_co	17/23	-	ENST0000C	ENSP0000(	3704
ENSG0000(	Transcript	ENST0000C	protein_co	17/23	-	ENST0000C	ENSP0000(	3520
ENSG0000(	Transcript	ENST0000C	protein_co	-	8/22	ENST0000C	-	-
ENSG0000(	Transcript	ENST0000C	protein_co	8/23	-	ENST0000C	ENSP0000(	935
ENSG0000(	Transcript	ENST0000C	protein_co	3/23	-	ENST0000C	ENSP0000(	246
ENSG0000(	Transcript	ENST0000C	protein_co	9/12	-	ENST0000C	ENSP0000(	1309
ENSG0000(	Transcript	ENST0000C	protein_co	-	10/11	ENST0000C	-	-
ENSG0000(	Transcript	ENST0000C	protein_co	-	9/57	ENST0000C	-	-
ENSG0000(	Transcript	ENST0000C	protein_co	-	29/57	ENST0000C	-	-
ENSG0000(	Transcript	ENST0000C	protein_co	53/58	-	ENST0000C	ENSP0000(	8160
ENSG0000(	Transcript	ENST0000C	protein_co	5/5	-	ENST0000C	ENSP0000(	1633
ENSG0000(	Transcript	ENST0000C	protein_co	21/24	-	ENST0000C	ENSP0000(	2861
ENSG0000(	Transcript	ENST0000C	protein_co	-	21/23	ENST0000C	-	-
ENSG0000(	Transcript	ENST0000C	protein_co	22/24	-	ENST0000C	ENSP0000(	2952
ENSG0000(	Transcript	ENST0000C	protein_co	24/24	-	ENST0000C	ENSP0000(	3367
ENSG0000(	Transcript	ENST0000C	protein_co	-	3/22	ENST0000C	-	-
ENSG0000(	Transcript	ENST0000C	protein_co	20/30	-	ENST0000C	ENSP0000(	4890
ENSG0000(	Transcript	ENST0000C	protein_co	18/30	-	ENST0000C	ENSP0000(	2619
ENSG0000(	Transcript	ENST0000C	protein_co	1/17	-	ENST0000C	ENSP0000(	74
ENSG0000(	Transcript	ENST0000C	protein_co	-	22/33	ENST0000C	-	-
ENSG0000(	Transcript	ENST0000C	protein_co	4/34	-	ENST0000C	ENSP0000(	936
ENSG0000(	Transcript	ENST0000C	protein_co	29/30	-	ENST0000C	ENSP0000(	5431
ENSG0000(	Transcript	ENST0000C	protein_co	24/30	-	ENST0000C	ENSP0000(	4643
ENSG0000(	Transcript	ENST0000C	protein_co	5/23	-	ENST0000C	ENSP0000(	1849
ENSG0000(	Transcript	ENST0000C	protein_co	19/22	-	ENST0000C	ENSP0000(	3713
ENSG0000(	Transcript	ENST0000C	protein_co	7/17	-	ENST0000C	ENSP0000(	840
ENSG0000(	Transcript	ENST0000C	protein_co	12/19	-	ENST0000C	ENSP0000(	1487
ENSG0000(	Transcript	ENST0000C	protein_co	31/36	-	ENST0000C	ENSP0000(	5814
ENSG0000(	Transcript	ENST0000C	protein_co	-	6/32	ENST0000C	-	-
ENSG0000(	Transcript	ENST0000C	protein_co	-	15/15	ENST0000C	-	-
ENSG0000(	Transcript	ENST0000C	protein_co	-	3/9	ENST0000C	-	-
ENSG0000(	Transcript	ENST0000C	protein_co	5/8	-	ENST0000C	ENSP0000(	1359
ENSG0000(	Transcript	ENST0000C	protein_co	23/30	-	ENST0000C	ENSP0000(	3671
ENSG0000(	Transcript	ENST0000C	protein_co	23/30	-	ENST0000C	ENSP0000(	3654
ENSG0000(	Transcript	ENST0000C	protein_co	44/44	-	ENST0000C	-	7419
ENSG0000(	Transcript	ENST0000C	protein_co	38/44	-	ENST0000C	ENSP0000(	6446
ENSG0000(	Transcript	ENST0000C	protein_co	37/44	-	ENST0000C	ENSP0000(	6334
ENSG0000(	Transcript	ENST0000C	protein_co	37/44	-	ENST0000C	ENSP0000(	6328
ENSG0000(	Transcript	ENST0000C	protein_co	37/44	-	ENST0000C	ENSP0000(	6310
ENSG0000(	Transcript	ENST0000C	protein_co	37/44	-	ENST0000C	ENSP0000(	6305-6306
ENSG0000(	Transcript	ENST0000C	protein_co	34/44	-	ENST0000C	ENSP0000(	5873
ENSG0000(	Transcript	ENST0000C	protein_co	34/44	-	ENST0000C	ENSP0000(	5860

ENSG0000(	Transcript	ENST0000C	protein_co	27/44	-	ENST0000C	ENSP0000C	4687
ENSG0000(	Transcript	ENST0000C	protein_co	27/44	-	ENST0000C	ENSP0000C	4655
ENSG0000(	Transcript	ENST0000C	protein_co	25/44	-	ENST0000C	ENSP0000C	4024
ENSG0000(	Transcript	ENST0000C	protein_co	25/44	-	ENST0000C	ENSP0000C	3940
ENSG0000(	Transcript	ENST0000C	protein_co	-	23/43	ENST0000C	-	-
ENSG0000(	Transcript	ENST0000C	protein_co	23/44	-	ENST0000C	ENSP0000C	3258
ENSG0000(	Transcript	ENST0000C	protein_co	6/44	-	ENST0000C	ENSP0000C	1037
ENSG0000(	Transcript	ENST0000C	protein_co	-	5/43	ENST0000C	-	-
ENSG0000(	Transcript	ENST0000C	protein_co	-	5/43	ENST0000C	-	-
ENSG0000(	Transcript	ENST0000C	protein_co	-	5/43	ENST0000C	-	-
ENSG0000(	Transcript	ENST0000C	protein_co	1/44	-	ENST0000C	ENSP0000C	406
ENSG0000(	Transcript	ENST0000C	protein_co	1/44	-	ENST0000C	ENSP0000C	353
ENSG0000(	Transcript	ENST0000C	protein_co	1/44	-	ENST0000C	ENSP0000C	329
ENSG0000(	Transcript	ENST0000C	protein_co	4/23	-	ENST0000C	ENSP0000C	757
ENSG0000(	Transcript	ENST0000C	protein_co	4/23	-	ENST0000C	ENSP0000C	759
ENSG0000(	Transcript	ENST0000C	protein_co	-	19/22	ENST0000C	-	-
ENSG0000(	Transcript	ENST0000C	protein_co	4/23	-	ENST0000C	ENSP0000C	990
ENSG0000(	Transcript	ENST0000C	protein_co	-	21/22	ENST0000C	-	-
ENSG0000(	Transcript	ENST0000C	protein_co	3/34	-	ENST0000C	ENSP0000C	594
ENSG0000(	Transcript	ENST0000C	protein_co	-	2/20	ENST0000C	-	-
ENSG0000(	Transcript	ENST0000C	protein_co	-	5/20	ENST0000C	-	-
ENSG0000(	Transcript	ENST0000C	protein_co	4/23	-	ENST0000C	ENSP0000C	571
ENSG0000(	Transcript	ENST0000C	protein_co	-	10/10	ENST0000C	-	-
ENSG0000(	Transcript	ENST0000C	protein_co	2/16	-	ENST0000C	ENSP0000C	839
ENSG0000(	Transcript	ENST0000C	protein_co	12/16	-	ENST0000C	ENSP0000C	2174
ENSG0000(	Transcript	ENST0000C	protein_co	61/67	-	ENST0000C	ENSP0000C	10547
ENSG0000(	Transcript	ENST0000C	protein_co	58/67	-	ENST0000C	ENSP0000C	9632
ENSG0000(	Transcript	ENST0000C	protein_co	32/67	-	ENST0000C	ENSP0000C	5425
ENSG0000(	Transcript	ENST0000C	protein_co	32/67	-	ENST0000C	ENSP0000C	4386
ENSG0000(	Transcript	ENST0000C	protein_co	-	16/66	ENST0000C	-	-
ENSG0000(	Transcript	ENST0000C	protein_co	16/67	-	ENST0000C	ENSP0000C	1783
ENSG0000(	Transcript	ENST0000C	protein_co	9/13	-	ENST0000C	ENSP0000C	1568
ENSG0000(	Transcript	ENST0000C	protein_co	9/13	-	ENST0000C	ENSP0000C	2023
ENSG0000(	Transcript	ENST0000C	protein_co	11/15	-	ENST0000C	ENSP0000C	1462
ENSG0000(	Transcript	ENST0000C	protein_co	10/15	-	ENST0000C	ENSP0000C	1170
ENSG0000(	Transcript	ENST0000C	protein_co	-	8/14	ENST0000C	-	-
ENSG0000(	Transcript	ENST0000C	protein_co	-	14/18	ENST0000C	-	-
ENSG0000(	Transcript	ENST0000C	protein_co	-	1/10	ENST0000C	-	-
ENSG0000(	Transcript	ENST0000C	protein_co	87/87	-	ENST0000C	ENSP0000C	12246
ENSG0000(	Transcript	ENST0000C	protein_co	65/87	-	ENST0000C	ENSP0000C	8896
ENSG0000(	Transcript	ENST0000C	protein_co	43/87	-	ENST0000C	ENSP0000C	5718
ENSG0000(	Transcript	ENST0000C	protein_co	25/87	-	ENST0000C	ENSP0000C	2940
ENSG0000(	Transcript	ENST0000C	protein_co	-	22/86	ENST0000C	-	-
ENSG0000(	Transcript	ENST0000C	protein_co	21/24	-	ENST0000C	ENSP0000C	3761
ENSG0000(	Transcript	ENST0000C	protein_co	-	8/23	ENST0000C	-	-
ENSG0000(	Transcript	ENST0000C	protein_co	-	4/9	ENST0000C	-	-
ENSG0000(	Transcript	ENST0000C	protein_co	-	3/9	ENST0000C	-	-

ENSG0000(	Transcript	ENST0000C	protein_co	7/16	-	ENST0000C	ENSP0000(	1012-1013
ENSG0000(	Transcript	ENST0000C	protein_co	-	29/42	ENST0000C	-	-
ENSG0000(	Transcript	ENST0000C	protein_co	25/43	-	ENST0000C	ENSP0000(	3457
ENSG0000(	Transcript	ENST0000C	protein_co	25/43	-	ENST0000C	ENSP0000(	3377
ENSG0000(	Transcript	ENST0000C	protein_co	-	22/30	ENST0000C	-	-
ENSG0000(	Transcript	ENST0000C	protein_co	8/25	-	ENST0000C	ENSP0000(	1505
ENSG0000(	Transcript	ENST0000C	protein_co	16/17	-	ENST0000C	ENSP0000(	2189
ENSG0000(	Transcript	ENST0000C	protein_co	15/17	-	ENST0000C	ENSP0000(	1992
ENSG0000(	Transcript	ENST0000C	protein_co	-	9/16	ENST0000C	-	-
ENSG0000(	Transcript	ENST0000C	protein_co	9/18	-	ENST0000C	ENSP0000(	1605
ENSG0000(	Transcript	ENST0000C	protein_co	-	3/8	ENST0000C	-	-
ENSG0000(	Transcript	ENST0000C	protein_co	-	-	-	-	-
ENSG0000(	Transcript	ENST0000C	protein_co	10/11	-	ENST0000C	ENSP0000(	1401
ENSG0000(	Transcript	ENST0000C	protein_co	10/11	-	ENST0000C	ENSP0000(	1405
ENSG0000(	Transcript	ENST0000C	protein_co	-	1/6	ENST0000C	-	-
ENSG0000(	Transcript	ENST0000C	protein_co	-	4/6	ENST0000C	-	-
ENSG0000(	Transcript	ENST0000C	protein_co	4/68	-	ENST0000C	ENSP0000(	848
ENSG0000(	Transcript	ENST0000C	protein_co	-	6/67	ENST0000C	-	-
ENSG0000(	Transcript	ENST0000C	protein_co	9/68	-	ENST0000C	ENSP0000(	1813
ENSG0000(	Transcript	ENST0000C	protein_co	29/68	-	ENST0000C	ENSP0000(	7110
ENSG0000(	Transcript	ENST0000C	protein_co	-	54/67	ENST0000C	-	-
ENSG0000(	Transcript	ENST0000C	protein_co	59/68	-	ENST0000C	ENSP0000(	14254-142
ENSG0000(	Transcript	ENST0000C	protein_co	12/43	-	ENST0000C	ENSP0000(	1813
ENSG0000(	Transcript	ENST0000C	protein_co	-	3/21	ENST0000C	-	-
ENSG0000(	Transcript	ENST0000C	protein_co	11/11	-	ENST0000C	ENSP0000(	1661
ENSG0000(	Transcript	ENST0000C	protein_co	3/11	-	ENST0000C	ENSP0000(	364
ENSG0000(	Transcript	ENST0000C	protein_co	2/11	-	ENST0000C	ENSP0000(	244
ENSG0000(	Transcript	ENST0000C	protein_co	-	7/14	ENST0000C	-	-
ENSG0000(	Transcript	ENST0000C	protein_co	5/21	-	ENST0000C	ENSP0000(	4637
ENSG0000(	Transcript	ENST0000C	protein_co	3/21	-	ENST0000C	ENSP0000(	2175
ENSG0000(	Transcript	ENST0000C	protein_co	22/25	-	ENST0000C	ENSP0000(	3347
ENSG0000(	Transcript	ENST0000C	protein_co	-	3/13	ENST0000C	-	-
ENSG0000(	Transcript	ENST0000C	protein_co	3/14	-	ENST0000C	ENSP0000(	929
ENSG0000(	Transcript	ENST0000C	protein_co	3/12	-	ENST0000C	ENSP0000(	936
ENSG0000(	Transcript	ENST0000C	protein_co	10/12	-	ENST0000C	ENSP0000(	1729
ENSG0000(	Transcript	ENST0000C	protein_co	34/36	-	ENST0000C	ENSP0000(	4988
ENSG0000(	Transcript	ENST0000C	protein_co	-	5/8	ENST0000C	-	-
ENSG0000(	Transcript	ENST0000C	protein_co	-	7/8	ENST0000C	-	-
ENSG0000(	Transcript	ENST0000C	protein_co	1/1	-	ENST0000C	ENSP0000(	1017-1018
ENSG0000(	Transcript	ENST0000C	protein_co	1/1	-	ENST0000C	ENSP0000(	1155
ENSG0000(	Transcript	ENST0000C	protein_co	-	13/13	ENST0000C	-	-
ENSG0000(	Transcript	ENST0000C	protein_co	14/14	-	ENST0000C	ENSP0000(	1945
ENSG0000(	Transcript	ENST0000C	protein_co	9/27	-	ENST0000C	ENSP0000(	1412
ENSG0000(	Transcript	ENST0000C	protein_co	7/12	-	ENST0000C	ENSP0000(	1048
ENSG0000(	Transcript	ENST0000C	protein_co	2/14	-	ENST0000C	ENSP0000(	281
ENSG0000(	Transcript	ENST0000C	protein_co	141/146	-	ENST0000C	ENSP0000(	26099
ENSG0000(	Transcript	ENST0000C	protein_co	136/146	-	ENST0000C	ENSP0000(	25072

ENSG0000(	Transcript	ENST0000C	protein_co	135/146	-	ENST0000C	ENSP0000C	24928
ENSG0000(	Transcript	ENST0000C	protein_co	125/146	-	ENST0000C	ENSP0000C	23279
ENSG0000(	Transcript	ENST0000C	protein_co	118/146	-	ENST0000C	ENSP0000C	22131
ENSG0000(	Transcript	ENST0000C	protein_co	89/146	-	ENST0000C	ENSP0000C	17607
ENSG0000(	Transcript	ENST0000C	protein_co	88/146	-	ENST0000C	ENSP0000C	17351
ENSG0000(	Transcript	ENST0000C	protein_co	86/146	-	ENST0000C	ENSP0000C	17102
ENSG0000(	Transcript	ENST0000C	protein_co	82/146	-	ENST0000C	ENSP0000C	16478
ENSG0000(	Transcript	ENST0000C	protein_co	78/146	-	ENST0000C	ENSP0000C	14225
ENSG0000(	Transcript	ENST0000C	protein_co	68/146	-	ENST0000C	ENSP0000C	11617
ENSG0000(	Transcript	ENST0000C	protein_co	52/146	-	ENST0000C	ENSP0000C	8341
ENSG0000(	Transcript	ENST0000C	protein_co	-	9/145	ENST0000C	-	-
ENSG0000(	Transcript	ENST0000C	protein_co	6/38	-	ENST0000C	ENSP0000C	905
ENSG0000(	Transcript	ENST0000C	protein_co	38/38	-	ENST0000C	ENSP0000C	5589
ENSG0000(	Transcript	ENST0000C	protein_co	1/1	-	ENST0000C	ENSP0000C	3920
ENSG0000(	Transcript	ENST0000C	protein_co	3/8	-	ENST0000C	ENSP0000C	432
ENSG0000(	Transcript	ENST0000C	protein_co	-	3/18	ENST0000C	-	-
ENSG0000(	Transcript	ENST0000C	protein_co	11/12	-	ENST0000C	ENSP0000C	1547-1550
ENSG0000(	Transcript	ENST0000C	protein_co	1/14	-	ENST0000C	ENSP0000C	510
ENSG0000(	Transcript	ENST0000C	protein_co	10/14	-	ENST0000C	ENSP0000C	1564
ENSG0000(	Transcript	ENST0000C	protein_co	2/12	-	ENST0000C	ENSP0000C	456
ENSG0000(	Transcript	ENST0000C	protein_co	4/12	-	ENST0000C	ENSP0000C	4109
ENSG0000(	Transcript	ENST0000C	protein_co	12/12	-	ENST0000C	ENSP0000C	6559
ENSG0000(	Transcript	ENST0000C	protein_co	3/11	-	ENST0000C	ENSP0000C	3787
ENSG0000(	Transcript	ENST0000C	protein_co	10/11	-	ENST0000C	ENSP0000C	5207
ENSG0000(	Transcript	ENST0000C	protein_co	3/10	-	ENST0000C	ENSP0000C	552
ENSG0000(	Transcript	ENST0000C	protein_co	17/22	-	ENST0000C	ENSP0000C	2811
ENSG0000(	Transcript	ENST0000C	protein_co	-	10-Mar	ENST0000C	-	-
ENSG0000(	Transcript	ENST0000C	protein_co	38/51	-	ENST0000C	ENSP0000C	5755
ENSG0000(	Transcript	ENST0000C	protein_co	34/51	-	ENST0000C	ENSP0000C	5127
ENSG0000(	Transcript	ENST0000C	protein_co	-	23/50	ENST0000C	-	-
ENSG0000(	Transcript	ENST0000C	protein_co	20/20	-	ENST0000C	ENSP0000C	3423
ENSG0000(	Transcript	ENST0000C	protein_co	-	5/19	ENST0000C	-	-
ENSG0000(	Transcript	ENST0000C	protein_co	-	15/20	ENST0000C	-	-
ENSG0000(	Transcript	ENST0000C	protein_co	-	13/20	ENST0000C	-	-
ENSG0000(	Transcript	ENST0000C	protein_co	11/21	-	ENST0000C	ENSP0000C	2814
ENSG0000(	Transcript	ENST0000C	protein_co	-	31/71	ENST0000C	-	-
ENSG0000(	Transcript	ENST0000C	protein_co	40/72	-	ENST0000C	ENSP0000C	5954
ENSG0000(	Transcript	ENST0000C	protein_co	40/72	-	ENST0000C	ENSP0000C	5958
ENSG0000(	Transcript	ENST0000C	protein_co	40/72	-	ENST0000C	ENSP0000C	5977
ENSG0000(	Transcript	ENST0000C	protein_co	-	57/71	ENST0000C	-	-
ENSG0000(	Transcript	ENST0000C	protein_co	-	28/41	ENST0000C	-	-
ENSG0000(	Transcript	ENST0000C	protein_co	-	4/10	ENST0000C	-	-
ENSG0000(	Transcript	ENST0000C	protein_co	5/11	-	ENST0000C	ENSP0000C	645
ENSG0000(	Transcript	ENST0000C	protein_co	10/11	-	ENST0000C	ENSP0000C	1063
ENSG0000(	Transcript	ENST0000C	protein_co	48/59	-	ENST0000C	ENSP0000C	7401
ENSG0000(	Transcript	ENST0000C	protein_co	45/59	-	ENST0000C	ENSP0000C	6965
ENSG0000(	Transcript	ENST0000C	protein_co	40/59	-	ENST0000C	ENSP0000C	6254

ENSG0000(	Transcript	ENST0000C	protein_co	31/59	-	ENST0000C	ENSP0000C	4631
ENSG0000(	Transcript	ENST0000C	protein_co	-	10/58	ENST0000C	-	-
ENSG0000(	Transcript	ENST0000C	protein_co	9/59	-	ENST0000C	ENSP0000C	1546
ENSG0000(	Transcript	ENST0000C	protein_co	-	42/44	ENST0000C	-	-
ENSG0000(	Transcript	ENST0000C	protein_co	19/24	-	ENST0000C	ENSP0000C	3561
ENSG0000(	Transcript	ENST0000C	protein_co	-	20/23	ENST0000C	-	-
ENSG0000(	Transcript	ENST0000C	protein_co	-	20/23	ENST0000C	-	-
ENSG0000(	Transcript	ENST0000C	protein_co	-	20/23	ENST0000C	-	-
ENSG0000(	Transcript	ENST0000C	protein_co	-	20/23	ENST0000C	-	-
ENSG0000(	Transcript	ENST0000C	protein_co	9/35	-	ENST0000C	ENSP0000C	1460
ENSG0000(	Transcript	ENST0000C	protein_co	-	23/34	ENST0000C	-	-
ENSG0000(	Transcript	ENST0000C	protein_co	-	29/34	ENST0000C	-	-
ENSG0000(	Transcript	ENST0000C	protein_co	34/35	-	ENST0000C	ENSP0000C	4378
ENSG0000(	Transcript	ENST0000C	protein_co	11/11	-	ENST0000C	ENSP0000C	1711
ENSG0000(	Transcript	ENST0000C	protein_co	4/11	-	ENST0000C	ENSP0000C	382

CDS_positi	Protein_po	Amino_aci	Codons	Existing_va	DISTANCE	STRAND	FLAGS	SYMBOL_S
586	196	E/*	Gaa/Taa	-	-	1	-	HGNC
-	-	-	-	-	-	-1	-	HGNC
-	-	-	-	-	-	1	-	HGNC
249	83	H	caC/caT	-	-	1	-	HGNC
2003	668	D/A	gAt/gCt	-	-	1	-	HGNC
-	-	-	-	-	-	1	-	HGNC
4769	1590	M/T	aTg/aCg	-	-	1	-	HGNC
-	-	-	-	-	-	1	-	HGNC
6358-6359	2120	E/EX	gaa/gAaa	-	-	1	-	HGNC
7709	2570	F/C	tTc/tGc	-	-	1	-	HGNC
8405	2802	A/E	gCa/gAa	-	-	1	-	HGNC
1189	397	P/S	Ccc/Tcc	-	-	-1	-	HGNC
-	-	-	-	-	-	-1	-	HGNC
1875	625	F/X	ttT/tt	-	-	-1	-	HGNC
1696	566	V/I	Gtt/Att	-	-	1	-	HGNC
1697	566	V/X	gTt/gt	-	-	1	-	HGNC
2680-2681	894	V/*	GTg/TAg	-	-	1	-	HGNC
3917	1306	E/X	gAa/ga	-	-	1	-	HGNC
4320-4341	1440-1447	PPPPQTAQ	ccACCACCT	-	-	1	-	HGNC
2681	894	E/X	gAa/ga	-	-	1	-	HGNC
2841	947	P/X	ccA/cc	-	-	1	-	HGNC
2843	948	Y/F	tAt/tTt	-	-	1	-	HGNC
3288	1096	Y/*	taT/taA	-	-	1	-	HGNC
3426	1142	Q/X	caG/ca	-	-	1	-	HGNC
5954	1985	S/F	tCc/tTc	-	-	1	-	HGNC
5027	1676	Q/P	cAg/cCg	-	-	1	-	HGNC
-	-	-	-	-	-	-1	-	HGNC
-	-	-	-	-	-	1	-	HGNC
-	-	-	-	-	-	1	-	HGNC
7377	2459	R	cgT/cgC	-	-	1	-	HGNC
-	-	-	-	-	-	-1	-	HGNC
-	-	-	-	-	-	-1	-	HGNC
6618	2206	K/X	aaA/aa	-	-	-1	-	HGNC
2838	946	M/I	atG/atC	-	-	-1	-	HGNC
-	-	-	-	-	-	-1	-	HGNC
-	-	-	-	-	-	-1	-	HGNC
-	-	-	-	-	-	-1	-	HGNC
351-352	117-118	-/X	-/A	-	-	-1	-	HGNC
624	208	L	ttA/ttG	-	-	-1	-	HGNC
-	-	-	-	-	-	1	-	HGNC
625	209	D/N	Gac/Aac	-	-	1	-	HGNC
4078	1360	N/D	Aat/Gat	-	-	1	-	HGNC
642	214	T	acT/acG	-	-	-1	-	HGNC
-	-	-	-	-	-	-1	-	HGNC
-	-	-	-	-	-	-1	-	HGNC

1174	392	P/S	Cca/Tca	-	-	-1		-	HGNC
736	246	Q/*	Cag/Tag	-	-	1		-	HGNC
-	-	-	-	-	-	1		-	HGNC
53	18	N/T	aAc/aCc	-	-	1		-	HGNC
476	159	R/I	aGa/aTa	-	-	1		-	HGNC
1242	414	N	aaT/aaC	-	-	1		-	HGNC
1627	543	P/T	Cca/Aca	-	-	1		-	HGNC
1635	545	E/D	gaA/gaC	-	-	1		-	HGNC
-	-	-	-	-	-	1		-	HGNC
-	-	-	-	-	-	1		-	HGNC
1542	514	K/N	aaA/aaT	-	-	1		-	HGNC
3655	1219	L/I	Ctt/Att	-	-	1		-	HGNC
-	-	-	-	-	-		-1	-	HGNC
-	-	-	-	-	-	-1		-	HGNC
-	-	-	-	-	-	-1		-	HGNC
-	-	-	-	-	-	-1		-	HGNC
1717	573	I/V	Atc/Gtc	-	-	-1		-	HGNC
1500	500	E/D	gaG/gaT	-	-	1		-	HGNC
-	-	-	-	-	-	1		-	HGNC
-	-	-	-	-	-		1	-	HGNC
-	-	-	-	-	-	1		-	HGNC
-	-	-	-	-	-	1		-	HGNC
-	-	-	-	-	-	1		-	HGNC
-	-	-	-	-	-	-1		-	HGNC
-	-	-	-	-	-	-1		-	HGNC
330	110	E/D	gaG/gaT	-	-	-1		-	HGNC
2354	785	R/Q	cGg/cAg	-	-	1		-	HGNC
1697	566	L/R	cTg/cGg	-	-	1		-	HGNC
-	-	-	-	-	-	-1		-	HGNC
-	-	-	-	-	-	1		-	Clone_base
207	69	N	aaC/aaT	-	-	1		-	HGNC
4326	1442	T	acA/acG	-	-	1		-	HGNC
-	-	-	-	-	-		-1	-	HGNC
1017	339	S/R	agT/agG	-	-	-1		-	HGNC
-	-	-	-	-	-	-1		-	HGNC
993	331	S	tcA/tcC	-	-	-1		-	HGNC
-	-	-	-	-	-	-1		-	HGNC
-	-	-	-	-	-	-1		-	HGNC
-	-	-	-	-	-	-1		-	HGNC
-	-	-	-	-	-	-1		-	HGNC
-	-	-	-	-	-	-1		-	HGNC
2197	733	K/E	Aaa/Gaa	-	-	1		-	HGNC
2660-2662	887-888	QK/Q	cAGAag/ca	-	-	1		-	HGNC
780	260	H/Q	caC/caA	-	-	1		-	HGNC
692	231	N/S	aAc/aGc	-	-	1		-	HGNC
559	187	A/T	Gcc/Acc	-	-	-1		-	HGNC
887	296	L/R	cTt/cGt	-	-	1		-	HGNC

907	303	D/H	Gat/Cat	-	-	1	-	HGNC
3163	1055	G/C	Ggt/Tgt	-	-	1	-	HGNC
-	-	-	-	-	-	1	-	HGNC
-	-	-	-	-	-	1	-	HGNC
-	-	-	-	-	-	1	-	HGNC
2299	767	A/S	Gca/Tca	-	-	1	-	HGNC
3840	1280	S	agC/agT	-	-	-1	-	HGNC
2739	913	*/Y	taA/taC	-	-	-1	-	HGNC
172	58	P/S	Ccc/Tcc	-	-	-1	-	HGNC
-	-	-	-	-	-	-1	-	HGNC
-	-	-	-	-	-	-1	-	HGNC
-	-	-	-	-	-	-1	-	HGNC
-	-	-	-	-	-	-1	-	HGNC
13835	4612	Q/L	cAg/cTg	-	-	-1	-	HGNC
13481	4494	V/G	gTg/gGg	-	-	-1	-	HGNC
13290	4430	P	ccT/ccG	-	-	-1	-	HGNC
8573	2858	Q/P	cAa/cCa	-	-	-1	-	HGNC
8273	2758	L/R	cTt/cGt	-	-	-1	-	HGNC
-	-	-	-	-	-	-1	-	HGNC
-	-	-	-	-	-	-1	-	HGNC
-	-	-	-	-	-	-1	-	HGNC
-	-	-	-	-	-	1	-	HGNC
-	-	-	-	-	-	1	-	HGNC
1543	515	G/R	Gga/Aga	-	-	1	-	HGNC
3079	1027	V/M	Gtg/Atg	-	-	1	-	HGNC
3513	1171	A	gcC/gcT	-	-	1	-	HGNC
-	-	-	-	-	-	1	-	HGNC
7370	2457	K/R	aAg/aGg	-	-	1	-	HGNC
9223	3075	T/P	Acc/Ccc	-	-	1	-	HGNC
-	-	-	-	-	-	1	-	HGNC
-	-	-	-	-	-	1	-	HGNC
1766	589	K/T	aAa/aCa	-	-	1	-	HGNC
-	-	-	-	-	-	-1	-	HGNC
-	-	-	-	-	-	-1	-	HGNC
-	-	-	-	-	-	-1	-	HGNC
1749	583	S	agC/agT	-	-	1	-	HGNC
-	-	-	-	-	-	1	-	HGNC
-	-	-	-	-	-	1	-	HGNC
3292	1098	L	Ctg/Ttg	-	-	1	-	HGNC
-	-	-	-	-	-	1	-	HGNC
3633	1211	T	acC/acA	-	-	-1	-	HGNC
-	-	-	-	-	-	-1	-	HGNC
-	-	-	-	-	-	-1	-	HGNC
114	38	T	acA/acG	-	-	-1	-	HGNC
-	-	-	-	-	-	-1	-	HGNC
510	170	Q/H	caG/caC	-	-	-1	-	HGNC
1687	563	S/C	Agc/Tgc	-	-	-1	-	HGNC

855	285	H	caC/caT	-	-	-1	-	HGNC
-	-	-	-	-	-	1	-	HGNC
-	-	-	-	-	-	-1	-	HGNC
1527-1528	509-510	-/MSSX	-/ATGAGTT	-	-	-1	-	HGNC
-	-	-	-	-	-	-1	-	HGNC
-	-	-	-	-	-	-1	-	HGNC
1166	389	Y/C	tAt/tGt	-	-	1	-	HGNC
3739	1247	K/X	Aaa/aa	-	-	1	-	HGNC
-	-	-	-	-	-	-1	-	HGNC
2080	694	E/*	Gaa/Taa	-	-	-1	-	HGNC
2010	670	I	atA/atT	-	-	-1	-	HGNC
2042	681	Y/C	tAc/tGc	-	-	-1	-	HGNC
-	-	-	-	-	-	1	-	HGNC
1720	574	P/X	Ccc/cc	-	-	1	-	HGNC
1308	436	V	gtG/gtA	-	-	1	-	HGNC
-	-	-	-	-	-	-1	-	HGNC
3866	1289	S/T	aGc/aCc	-	-	-1	-	HGNC
-	-	-	-	-	-	-1	-	HGNC
6262	2088	L/V	Ctg/Gtg	-	-	-1	-	HGNC
-	-	-	-	-	-	-1	-	HGNC
788	263	F/C	tTt/tGt	-	-	-1	-	HGNC
1222	408	M/V	Atg/Gtg	-	-	1	-	HGNC
126-127	42-43	-/X	-/G	-	-	-1	-	HGNC
1526	509	A/V	gCc/gTc	-	-	1	-	HGNC
-	-	-	-	-	-	1	-	HGNC
960	320	G	ggC/ggG	-	-	-1	-	HGNC
117	39	P	ccC/ccG	-	-	1	-	HGNC
-	-	-	-	-	-	1	-	HGNC
2858	953	R/L	cGg/cTg	-	-	1	-	HGNC
2034	678	F	ttC/ttT	-	-	-1	-	HGNC
-	-	-	-	-	-	1	-	HGNC
2526	842	E/D	gaA/gaC	-	-	-1	-	HGNC
-	-	-	-	-	-	-1	-	HGNC
1168	390	D/Y	Gac/Tac	-	-	-1	-	HGNC
-	-	-	-	-	-	1	-	HGNC
-	-	-	-	-	-	1	-	HGNC
-	-	-	-	-	-	1	-	HGNC
4439	1480	Q/R	cAa/cGa	-	-	1	-	HGNC
-	-	-	-	-	-	1	-	HGNC
-	-	-	-	-	-	1	-	HGNC
2575	859	S/A	Tcc/Gcc	-	-	-1	-	HGNC
2071	691	V/L	Gta/Tta	-	-	-1	-	HGNC
-	-	-	-	-	-	-1	-	HGNC
862	288	D/N	Gac/Aac	-	-	-1	-	HGNC
-	-	-	-	-	-	-1	-	HGNC
540	180	D	gaC/gaT	-	-	1	-	HGNC
3561	1187	E	gaG/gaA	-	-	-1	-	HGNC

340	114	E/*	Gag/Tag	-	-	1	-	HGNC
-	-	-	-	-	-	1	-	HGNC
671	224	T/M	aCg/aTg	-	-	1	-	HGNC
1540	514	P/X	Ccc/cc	-	-	1	-	HGNC
-	-	-	-	-	-	1	-	HGNC
-	-	-	-	-	-	1	-	HGNC
-	-	-	-	-	-	-1	-	HGNC
311	104	S/Y	tCc/tAc	-	-	1	-	HGNC
-	-	-	-	-	-	1	-	HGNC
1927	643	C/R	Tgt/Cgt	-	-	1	-	HGNC
1951	651	L/I	Cta/Ata	-	-	1	-	HGNC
2488-2489	830	L/*	CTg/TAg	-	-	1	-	HGNC
-	-	-	-	-	-	1	-	HGNC
4697-4698	1566	P/PX	cca/ccTa	-	-	-1	-	HGNC
1321	441	F/X	Ttt/tt	-	-	1	-	HGNC
1909	637	Y/H	Tat/Cat	-	-	1	-	HGNC
2490	830	T/X	acA/ac	-	-	1	-	HGNC
2494	832	N/Y	Aat/Tat	-	-	1	-	HGNC
2501	834	E/G	gAa/gGa	-	-	1	-	HGNC
881	294	S/N	aGc/aAc	-	-	-1	-	HGNC
-	-	-	-	-	-	1	-	HGNC
-	-	-	-	-	-	-1	-	HGNC
1889	630	K/T	aAg/aCg	-	-	-1	-	HGNC
798	266	K/N	aaG/aaT	-	-	-1	-	HGNC
428-429	143	N/KX	aac/aaAc	-	-	-1	-	HGNC
1333	445	P/S	Cca/Tca	-	-	-1	-	HGNC
239	80	K/R	aAg/aGg	-	-	-1	-	HGNC
40-41	14	V/GV	gta/gGCGta	-	-	-1	-	HGNC
3112	1038	N/H	Aat/Cat	-	-	-1	-	HGNC
-	-	-	-	-	-	-1	-	HGNC
-	-	-	-	-	-	-1	-	HGNC
-	-	-	-	-	-	-1	-	HGNC
1517	506	T/S	aCt/aGt	-	-	-1	-	HGNC
1022	341	Y/C	tAc/tGc	-	-	1	-	HGNC
3376	1126	L/I	Cta/Ata	-	-	1	-	HGNC
-	-	-	-	-	-	-1	-	HGNC
-	-	-	-	-	-	-1	-	HGNC
9918	3306	L	ctG/ctC	-	-	-1	-	HGNC
-	-	-	-	-	-	-1	-	HGNC
7385	2462	S/N	aGc/aAc	-	-	-1	-	HGNC
5009	1670	F/C	tTt/tGt	-	-	-1	-	HGNC
-	-	-	-	-	-	-1	-	HGNC
3208	1070	L/V	Ttg/Gtg	-	-	-1	-	HGNC
1113	371	Q	caG/caA	-	-	-1	-	HGNC
-	-	-	-	-	-	-1	-	HGNC
-	-	-	-	-	-	-1	-	HGNC
-	-	-	-	-	-	-1	-	HGNC

-	-	-	-	-	-	-1	-	HGNC
2307	769	E	gaG/gaA	-	-	-1	-	HGNC
2182	728	S/G	Agt/Ggt	-	-	-1	-	HGNC
969	323	D	gaC/gaT	-	-	-1	-	HGNC
805	269	P/S	Cca/Tca	-	-	-1	-	HGNC
1040	347	A/V	gCa/gTa	-	-	1	-	HGNC
-	-	-	-	-	-	1	-	HGNC
2186	729	F/Y	tTc/tAc	-	-	1	-	HGNC
-	-	-	-	-	1038	-1	-	HGNC
-	-	-	-	-	-	-1	-	HGNC
934	312	W/R	Tgg/Cgg	-	-	-1	-	HGNC
1581	527	V	gtC/gtT	-	-	-1	-	HGNC
729	243	D/E	gaT/gaG	-	-	1	-	HGNC
1991	664	P/R	cCg/cGg	-	-	1	-	HGNC
-	-	-	-	-	-	1	-	HGNC
-	-	-	-	-	-	1	-	HGNC
1936	646	Y/N	Tat/Aat	-	-	1	-	HGNC
9571	3191	V/F	Gtt/Ttt	-	-	1	-	HGNC
11305	3769	A/T	Gct/Act	-	-	1	-	HGNC
-	-	-	-	-	-	-1	-	HGNC
11007	3669	P	ccC/ccT	-	-	-1	-	HGNC
5701	1901	M/X	Atg/tg	-	-	-1	-	HGNC
-	-	-	-	-	-	-1	-	HGNC
-	-	-	-	-	-	-1	-	HGNC
-	-	-	-	-	-	-1	-	HGNC
1732	578	D/N	Gat/Aat	-	-	-1	-	HGNC
-	-	-	-	-	-	-1	-	HGNC
-	-	-	-	-	-	1	-	HGNC
-	-	-	-	-	-	1	-	HGNC
-	-	-	-	-	-	1	-	HGNC
-	-	-	-	-	-	1	-	HGNC
-	-	-	-	-	43	1	-	HGNC
126	42	E/D	gaA/gaC	-	-	-1	-	HGNC
679	227	R/*	Aga/Tga	-	-	1	-	HGNC
-	-	-	-	-	-	1	-	HGNC
1010	337	Q/P	cAa/cCa	-	-	1	-	HGNC
2260-2265	754-755	TS/-	ACTTCT/-	-	-	1	-	HGNC
-	-	-	-	-	-	-1	-	HGNC
128-129	43	R/L	cGG/cTT	-	-	1	-	HGNC
-	-	-	-	-	-	1	-	HGNC
-	-	-	-	-	-	1	-	HGNC
3467	1156	E/G	gAg/gGg	-	-	1	-	HGNC
?-1082	?-361	-	-	-	-	-1	-	HGNC
759	253	S	tcT/tcC	-	-	-1	-	HGNC
243	81	Y/*	taC/taA	-	-	-1	-	HGNC
1161-1162	387-388	-/*	-/TGA	-	-	1	-	HGNC
-	-	-	-	-	-	1	-	HGNC

624	208	R/S	agG/agT	-	-	-1	-	HGNC	
1224	408	V	gtG/gtA	-	-	1	-	HGNC	
5708	1903	I/T	aTc/aCc	-	-	-1	-	HGNC	
-	-	-	-	-	-	-1	-	HGNC	
-	-	-	-	-	-	-1	-	HGNC	
3522	1174	P	ccG/ccT	-	-	-1	-	HGNC	
3338	1113	D/G	gAt/gGt	-	-	-1	-	HGNC	
-	-	-	-	-	-	-1	-	HGNC	
753	251	C/W	tgC/tgG	-	-	-1	-	HGNC	
64	22	C/R	Tgt/Cgt	-	-	-1	-	HGNC	
1057	353	V/M	Gtg/Atg	-	-	1	-	HGNC	
-	-	-	-	-	-	1	-	HGNC	
-	-	-	-	-	-	1	-	HGNC	
-	-	-	-	-	-	1	-	HGNC	
7777	2593	L/V	Tta/Gta	-	-	1	-	HGNC	
1078	360	S/P	Tca/Cca	-	-	-1	-	HGNC	
2394	798	F/L	ttT/ttG	-	-	1	-	HGNC	
-	-	-	-	-	-	1	-	HGNC	
2485	829	P/T	Cca/Aca	-	-	1	-	HGNC	
2900	967	G/V	gGc/gTc	-	-	1	-	HGNC	
-	-	-	-	-	-	1	-	HGNC	
4699	1567	A/P	Gca/Cca	-	-	-1	-	HGNC	
2428	810	R/G	Aga/Gga	-	-	-1	-	HGNC	
74	25	Q/L	cAg/cTg	-	-	-1	-	HGNC	
-	-	-	-	-	-	-1	-	HGNC	
716	239	T/S	aCt/aGt	-	-	-1	-	HGNC	
5292	1764	D	gaC/gaT	-	-	-1	-	HGNC	
4504	1502	P/S	Ccc/Tcc	-	-	-1	-	HGNC	
1804	602	C/R	Tgt/Cgt	-	-	1	-	HGNC	
3316	1106	L/V	Ctt/Gtt	-	-	-1	-	HGNC	
799	267	A/T	Gca/Aca	-	-	1	-	HGNC	
1325	442	A/V	gCc/gTc	-	-	-1	-	HGNC	
5670	1890	G	ggA/ggG	-	-	1	-	HGNC	
-	-	-	-	-	-	-1	-	HGNC	
-	-	-	-	-	-	1	-	HGNC	
-	-	-	-	-	-	-1	-	HGNC	
761	254	T/I	aCc/aTc	-	-	1	-	HGNC	
3571	1191	F/V	Ttt/Gtt	-	-	-1	-	HGNC	
3554	1185	F/S	tTc/tCc	-	-	-1	-	HGNC	
-	-	-	-	-	-	-1	-	HGNC	
6155	2052	R/Q	cGa/cAa	-	-	-1	-	HGNC	
6043	2015	I/V	Atc/Gtc	-	-	-1	-	HGNC	
6037	2013	L	Cta/Tta	-	-	-1	-	HGNC	
6019	2007	E/*	Gag/Tag	-	-	-1	-	HGNC	
6014-6015	2005	L/Q	cTC/cAA	-	-	-1	-	HGNC	
5582	1861	S/Y	tCc/tAc	-	-	-1	-	HGNC	
5569	1857	R/C	Cgc/Tgc	-	-	-1	-	HGNC	

4396	1466	R/G	Aga/Gga	-	-	-1	-	HGNC
4364	1455	N/X	aAt/at	-	-	-1	-	HGNC
3733	1245	A/T	Gct/Act	-	-	-1	-	HGNC
3649	1217	R/C	Cgc/Tgc	-	-	-1	-	HGNC
-	-	-	-	-	-	-1	-	HGNC
2967	989	S	tcC/tcA	-	-	-1	-	HGNC
746	249	Q/R	cAg/cGg	-	-	-1	-	HGNC
-	-	-	-	-	-	-1	-	HGNC
-	-	-	-	-	-	-1	-	HGNC
-	-	-	-	-	-	-1	-	HGNC
115	39	A/T	Gcc/Acc	-	-	-1	-	HGNC
62	21	S/I	aGc/aTc	-	-	-1	-	HGNC
38	13	N/S	aAt/aGt	-	-	-1	-	HGNC
426	142	D	gaT/gaC	-	-	1	-	HGNC
428	143	D/G	gAt/gGt	-	-	1	-	HGNC
-	-	-	-	-	-	-1	-	HGNC
520	174	D/Y	Gat/Tat	-	-	-1	-	HGNC
-	-	-	-	-	-	-1	-	HGNC
37	13	S/P	Tcc/Ccc	-	-	-1	-	HGNC
-	-	-	-	-	-	1	-	HGNC
-	-	-	-	-	-	1	-	HGNC
497	166	L/S	tTg/tCg	-	-	-1	-	HGNC
-	-	-	-	-	-	1	-	HGNC
223	75	E/*	Gaa/Taa	-	-	1	-	HGNC
1558	520	E/*	Gaa/Taa	-	-	1	-	HGNC
10271	3424	I/N	aTt/aAt	-	-	-1	-	HGNC
9356	3119	S/Y	tCt/tAt	-	-	-1	-	HGNC
5149	1717	R/*	Aga/Tga	-	-	-1	-	HGNC
4110	1370	Q/H	caG/caT	-	-	-1	-	HGNC
-	-	-	-	-	-	-1	-	HGNC
1507	503	V/I	Gta/Ata	-	-	-1	-	HGNC
1335	445	S	tcA/tcC	-	-	1	-	HGNC
1790	597	S/N	aGt/aAt	-	-	1	-	HGNC
1356	452	G	ggT/ggG	-	-	-1	-	HGNC
1064	355	L/*	tTa/tAa	-	-	-1	-	HGNC
-	-	-	-	-	-	-1	-	HGNC
-	-	-	-	-	-	-1	-	HGNC
-	-	-	-	-	-	1	-	HGNC
12189	4063	L	ctA/ctG	-	-	-1	-	HGNC
8839	2947	G/R	Gga/Cga	-	-	-1	-	HGNC
5661	1887	D	gaT/gaC	-	-	-1	-	HGNC
2883	961	L	ctC/ctG	-	-	-1	-	HGNC
-	-	-	-	-	-	-1	-	HGNC
3461	1154	A/D	gCt/gAt	-	-	-1	-	HGNC
-	-	-	-	-	-	-1	-	HGNC
-	-	-	-	-	-	-1	-	HGNC
-	-	-	-	-	-	-1	-	HGNC

814-815	272	E/EX	gaa/gAaa	-	-	1	-	HGNC	
-	-	-	-	-	-	-1	-	HGNC	
2913	971	L	ctT/ctA	-	-	-1	-	HGNC	
2833	945	A/T	Gca/Aca	-	-	-1	-	HGNC	
-	-	-	-	-	-	-1	-	HGNC	
1118	373	S/Y	tCt/tAt	-	-	1	-	HGNC	
1749	583	R	agG/agA	-	-	-1	-	HGNC	
1552	518	R/*	Cga/Tga	-	-	-1	-	HGNC	
-	-	-	-	-	-	-1	-	HGNC	
1583	528	S/N	aGc/aAc	-	-	-1	-	HGNC	
-	-	-	-	-	-	1	-	HGNC	
-	-	-	-	-	4753	1	-	HGNC	
1081	361	K/X	Aaa/aa	-	-	1	-	HGNC	
1085	362	K/R	aAa/aGa	-	-	1	-	HGNC	
-	-	-	-	-	-	1	-	HGNC	
-	-	-	-	-	-	1	-	HGNC	
705	235	E/D	gaA/gaT	-	-	1	-	HGNC	
-	-	-	-	-	-	1	-	HGNC	
1670	557	E/V	gAg/gTg	-	-	1	-	HGNC	
6967	2323	Q/*	Cag/Tag	-	-	1	-	HGNC	
-	-	-	-	-	-	1	-	HGNC	
14111-141	4704	S/SX	agc/agCc	-	-	1	-	HGNC	
1614	538	E/D	gaA/gaC	-	-	-1	-	HGNC	
-	-	-	-	-	-	-1	-	HGNC	
1650	550	C/W	tgC/tgG	-	-	-1	-	HGNC	
353	118	S/F	tCc/tTc	-	-	-1	-	HGNC	
233	78	R/I	aGa/aTa	-	-	-1	-	HGNC	
-	-	-	-	-	-	1	-	HGNC	
4594	1532	R/W	Cgg/Tgg	-	-	-1	-	HGNC	
2132	711	V/A	gTc/gCc	-	-	-1	-	HGNC	
3255	1085	A	gcA/gcC	-	-	-1	-	HGNC	
-	-	-	-	-	-	-1	-	HGNC	
331	111	P/S	Cca/Tca	-	-	-1	-	HGNC	
398	133	Y/F	tAc/tTc	-	-	1	-	HGNC	
1191	397	V	gtT/gtC	-	-	1	-	HGNC	
4707	1569	D	gaC/gaT	-	-	1	-	HGNC	
-	-	-	-	-	-	1	-	HGNC	
-	-	-	-	-	-	1	-	HGNC	
962-963	321	Q/HX	cag/caTg	-	-	1	-	HGNC	
728	243	V/A	gTg/gCg	-	-	1	-	HGNC	
-	-	-	-	-	-	1	-	HGNC	
1596	532	P	ccC/ccG	-	-	1	-	HGNC	
1133	378	P/L	cCt/cTt	-	-	1	-	HGNC	
882	294	G	ggC/ggT	-	-	1	-	HGNC	
133	45	R/S	Cgc/Agc	-	-	1	-	HGNC	
25497	8499	I	atC/atA	-	-	-1	-	HGNC	
24470	8157	S/*	tCa/tAa	-	-	-1	-	HGNC	

24326	8109	Q/R	cAg/cGg	-	-	-1	-	HGNC
22677	7559	D	gaC/gaT	-	-	-1	-	HGNC
21529	7177	Q/E	Caa/Gaa	-	-	-1	-	HGNC
17005	5669	L/F	Ctc/Ttc	-	-	-1	-	HGNC
16749	5583	L	ctG/ctT	-	-	-1	-	HGNC
16500	5500	K/N	aaG/aaT	-	-	-1	-	HGNC
15876	5292	P	ccG/ccT	-	-	-1	-	HGNC
13623	4541	S/R	agT/agG	-	-	-1	-	HGNC
11015	3672	N/S	aAc/aGc	-	-	-1	-	HGNC
7739	2580	R/I	aGa/aTa	-	-	-1	-	HGNC
-	-	-	-	-	-	-1	-	HGNC
895	299	V/L	Gtg/Ctg	-	-	1	-	HGNC
5579	1860	S/F	tCt/tTt	-	-	1	-	HGNC
3830	1277	P/L	cCt/cTt	-	-	-1	-	HGNC
404	135	G/A	gGg/gCg	-	-	1	-	HGNC
-	-	-	-	-	-	-1	-	HGNC
1272-1275	424-425	KR/X	aaGAGG/aa	-	-	1	-	HGNC
3	1	M/I	atG/atT	-	-	1	-	HGNC
1057	353	P/S	Cct/Tct	-	-	1	-	HGNC
247	83	N/D	Aat/Gat	-	-	1	-	HGNC
3900	1300	T	acC/acA	-	-	1	-	HGNC
6350	2117	V/D	gTt/gAt	-	-	1	-	HGNC
2927	976	Q/L	cAg/cTg	-	-	1	-	HGNC
4347	1449	S/X	tcT/tc	-	-	1	-	HGNC
292	98	A/T	Gca/Aca	-	-	-1	-	HGNC
2646	882	Q/H	caG/caT	-	-	1	-	HGNC
-	-	-	-	-	-	-1	-	HGNC
5458	1820	S/T	Tcg/Acg	-	-	-1	-	HGNC
4830	1610	I	atT/atC	-	-	-1	-	HGNC
-	-	-	-	-	-	-1	-	HGNC
3339	1113	R	cgC/cgT	-	-	-1	-	HGNC
-	-	-	-	-	-	-1	-	HGNC
-	-	-	-	-	-	-1	-	HGNC
-	-	-	-	-	-	-1	-	HGNC
2440	814	E/K	Gaa/Aaa	-	-	-1	-	HGNC
-	-	-	-	-	-	1	-	HGNC
5745	1915	E/D	gaA/gaC	-	-	1	-	HGNC
5749	1917	R/*	Cga/Tga	-	-	1	-	HGNC
5768	1923	A/V	gCg/gTg	-	-	1	-	HGNC
-	-	-	-	-	-	1	-	HGNC
-	-	-	-	-	-	1	-	HGNC
-	-	-	-	-	-	1	-	HGNC
323	108	I/S	aTt/aGt	-	-	1	-	HGNC
741	247	E	gaG/gaA	-	-	1	-	HGNC
6794	2265	R/Q	cGa/cAa	-	-	-1	-	HGNC
6358	2120	K/E	Aaa/Gaa	-	-	-1	-	HGNC
5647	1883	R	Agg/Cgg	-	-	-1	-	HGNC

4024	1342	M/V	Atg/Gtg	-	-	-1	-	HGNC
-	-	-	-	-	-	-1	-	HGNC
939	313	S	tcC/tcG	-	-	-1	-	HGNC
-	-	-	-	-	-	1	-	HGNC
3130	1044	Q/*	Cag/Tag	-	-	1	-	HGNC
-	-	-	-	-	-	1	-	HGNC
-	-	-	-	-	-	1	-	HGNC
-	-	-	-	-	-	1	-	HGNC
-	-	-	-	-	-	1	-	HGNC
1211	404	I/X	aTt/at	-	-	1	-	HGNC
-	-	-	-	-	-	1	-	HGNC
-	-	-	-	-	-	1	-	HGNC
4129	1377	G/C	Ggt/Tgt	-	-	1	-	HGNC
1503	501	Q/X	caG/ca	-	-	-1	-	HGNC
174	58	S	tcA/tcG	-	-	-1	-	HGNC

HGNC_ID	CANONICA	MANE	TSL	APPRIS	CCDS	ENSP	SWISSPROT	TREMBL
173	YES	-	-	-	CCDS3330	ENSP0000(	P27037	B4DWQ2
17178	YES	-	-	-	CCDS3177	ENSP0000(	P59510	-
7135	YES	-	-	-	CCDS5477	ENSP0000(	P51825	Q712L1,F5
7135	YES	-	-	-	CCDS5477	ENSP0000(	P51825	Q712L1,F5
379	YES	-	-	-	CCDS5622.	ENSP0000(	Q99996	Q8IW64
379	YES	-	-	-	CCDS5622.	ENSP0000(	Q99996	Q8IW64
379	YES	-	-	-	CCDS5622.	ENSP0000(	Q99996	Q8IW64
379	YES	-	-	-	CCDS5622.	ENSP0000(	Q99996	Q8IW64
379	YES	-	-	-	CCDS5622.	ENSP0000(	Q99996	Q8IW64
379	YES	-	-	-	CCDS5622.	ENSP0000(	Q99996	Q8IW64
379	YES	-	-	-	CCDS5622.	ENSP0000(	Q99996	Q8IW64
392	YES	-	-	-	CCDS1255	ENSP0000(	P31751	Q05BV0,M
393	YES	-	-	-	CCDS3107	ENSP0000(	Q9Y243	F8VS91
427	YES	-	-	-	CCDS3317	ENSP0000(	Q9UM73	Q580I3
583	YES	-	-	-	CCDS4107.	ENSP0000(	P25054	Q9UM98,C
583	YES	-	-	-	CCDS4107.	ENSP0000(	P25054	Q9UM98,C
583	YES	-	-	-	CCDS4107.	ENSP0000(	P25054	Q9UM98,C
583	YES	-	-	-	CCDS4107.	ENSP0000(	P25054	Q9UM98,C
583	YES	-	-	-	CCDS4107.	ENSP0000(	P25054	Q9UM98,C
644	YES	-	-	-	CCDS1438	ENSP0000(	-	Q9NUA2,Q
11110	YES	-	-	-	CCDS285.1	ENSP0000(	O14497	Q96T01,Q
11110	YES	-	-	-	CCDS285.1	ENSP0000(	O14497	Q96T01,Q
11110	YES	-	-	-	CCDS285.1	ENSP0000(	O14497	Q96T01,Q
11110	YES	-	-	-	CCDS285.1	ENSP0000(	O14497	Q96T01,Q
11110	YES	-	-	-	CCDS285.1	ENSP0000(	O14497	Q96T01,Q
18037	YES	-	-	-	CCDS3178	ENSP0000(	Q68CP9	Q96SQ4,F8
700	YES	-	-	-	CCDS970.1	ENSP0000(	P27540	-
795	YES	-	-	-	CCDS3166	ENSP0000(	Q13315	M0QXY8,E
795	YES	-	-	-	CCDS3166	ENSP0000(	Q13315	M0QXY8,E
795	YES	-	-	-	CCDS3166	ENSP0000(	Q13315	M0QXY8,E
882	YES	-	-	-	CCDS3124.	ENSP0000(	Q13535	-
882	YES	-	-	-	CCDS3124.	ENSP0000(	Q13535	-
882	YES	-	-	-	CCDS3124.	ENSP0000(	Q13535	-
882	YES	-	-	-	CCDS3124.	ENSP0000(	Q13535	-
882	YES	-	-	-	CCDS3124.	ENSP0000(	Q13535	-
882	YES	-	-	-	CCDS3124.	ENSP0000(	Q13535	-
886	YES	-	-	-	CCDS1443	ENSP0000(	P46100	B4DLE1
11393	YES	-	-	-	CCDS1345	ENSP0000(	O14965	Q5QPD4,Q
11390	YES	-	-	-	CCDS1113	ENSP0000(	Q96GD4	J3QR41,J3
11391	YES	-	-	-	CCDS3312	ENSP0000(	Q9UQB9	-
945	YES	-	-	-	CCDS4968.	ENSP0000(	O60242	S4R3D0,B7
945	YES	-	-	-	CCDS4968.	ENSP0000(	O60242	S4R3D0,B7
989	YES	-	-	-	CCDS704.1	ENSP0000(	O95999	-
989	YES	-	-	-	CCDS704.1	ENSP0000(	O95999	-
13221	YES	-	-	-	CCDS1862.	ENSP0000(	Q9H165	D6W5D9,Q

1001	YES	-	-	-	CCDS3289.	ENSP00000	P41182	C9JL16,C9J
1008	YES	-	-	-	CCDS30833	ENSP00000	O00512	-
1008	YES	-	-	-	CCDS30833	ENSP00000	O00512	-
591	YES	-	-	-	CCDS8315.	ENSP00000	Q13489	-
591	YES	-	-	-	CCDS8315.	ENSP00000	Q13489	-
591	YES	-	-	-	CCDS8315.	ENSP00000	Q13489	-
591	YES	-	-	-	CCDS8315.	ENSP00000	Q13489	-
591	YES	-	-	-	CCDS8315.	ENSP00000	Q13489	-
593	YES	-	-	-	CCDS32752	ENSP00000	-	H3BLT4
593	YES	-	-	-	CCDS32752	ENSP00000	-	H3BLT4
1058	YES	-	-	-	CCDS10363	ENSP00000	P54132	-
1058	YES	-	-	-	CCDS10363	ENSP00000	P54132	-
14211	YES	-	-	-	CCDS7446.	ENSP00000	Q8WV28	Q2MD56
20473	YES	-	-	-	CCDS11631	ENSP00000	Q9BX63	J3KS24
20473	YES	-	-	-	CCDS11631	ENSP00000	Q9BX63	J3KS24
20473	YES	-	-	-	CCDS11631	ENSP00000	Q9BX63	J3KS24
16393	YES	-	-	-	CCDS5336.	ENSP00000	Q9BXL7	Q8TES3,E2
24054	YES	-	-	-	CCDS42023	ENSP00000	Q8NG31	-
24054	YES	-	-	-	CCDS42023	ENSP00000	Q8NG31	-
1541	YES	-	-	-	CCDS8418.	ENSP00000	P22681	-
16783	YES	-	-	-	CCDS1382.	ENSP00000	Q6P1J9	-
16783	YES	-	-	-	CCDS1382.	ENSP00000	Q6P1J9	-
1748	YES	-	-	-	CCDS10869	ENSP00000	P12830	B3GN61
1750	YES	-	-	-	CCDS10803	ENSP00000	P55287	H3BUU9,H3
1759	YES	-	-	-	CCDS11891	ENSP00000	P19022	C9JMH2,C9
1759	YES	-	-	-	CCDS11891	ENSP00000	P19022	C9JMH2,C9
1760	YES	-	-	-	CCDS11977	ENSP00000	Q9HBT6	Q8N9J3,K7
24224	YES	-	-	-	CCDS11337	ENSP00000	Q9NYV4	-
1773	YES	-	-	-	CCDS8953.	ENSP00000	P11802	Q6LC83,F8
-	YES	-	-	-	-	ENSP00000	-	F2Z2F3
14214	YES	-	-	-	CCDS12601	ENSP00000	Q96RK0	-
14214	YES	-	-	-	CCDS12601	ENSP00000	Q96RK0	-
2197	YES	-	-	-	CCDS11561	ENSP00000	P02452	Q9UMA6,C
30185	YES	-	-	-	CCDS2562.	ENSP00000	Q96SW2	-
30185	YES	-	-	-	CCDS2562.	ENSP00000	Q96SW2	-
2348	YES	-	-	-	CCDS10509	ENSP00000	Q92793	Q75MY6,I3
19291	YES	-	-	-	CCDS6315.	ENSP00000	Q7Z407	-
19291	YES	-	-	-	CCDS6315.	ENSP00000	Q7Z407	-
19291	YES	-	-	-	CCDS6315.	ENSP00000	Q7Z407	-
19291	YES	-	-	-	CCDS6315.	ENSP00000	Q7Z407	-
19291	YES	-	-	-	CCDS6315.	ENSP00000	Q7Z407	-
2509	YES	-	-	-	CCDS34243	ENSP00000	P35221	F8W845,E5
2509	YES	-	-	-	CCDS34243	ENSP00000	P35221	F8W845,E5
2514	YES	-	-	-	CCDS2694.	ENSP00000	P35222	G9GAG7,E9
2621	YES	-	-	-	CCDS7436.	ENSP00000	P33261	-
2625	YES	-	-	-	CCDS46721	ENSP00000	-	Q6NWU0,C
2701	YES	-	-	-	CCDS11952	ENSP00000	P43146	J3QQJ6

2701	YES	-	-	-	CCDS11952	ENSP00000	P43146	J3QQJ6
2701	YES	-	-	-	CCDS11952	ENSP00000	P43146	J3QQJ6
2701	YES	-	-	-	CCDS11952	ENSP00000	P43146	J3QQJ6
2731	YES	-	-	-	CCDS1241.	ENSP00000	Q16832	Q5T245,Q5
2731	YES	-	-	-	CCDS1241.	ENSP00000	Q16832	Q5T245,Q5
2731	YES	-	-	-	CCDS1241.	ENSP00000	Q16832	Q5T245,Q5
17098	YES	-	-	-	CCDS9931.	ENSP00000	Q9UPY3	Q5D0K5,B3
2978	YES	-	-	-	CCDS33157	ENSP00000	Q9Y6K1	Q8WVA9,C
2978	YES	-	-	-	CCDS33157	ENSP00000	Q9Y6K1	Q8WVA9,C
3012	YES	-	-	-	CCDS30777	ENSP00000	Q12882	-
3012	YES	-	-	-	CCDS30777	ENSP00000	Q12882	-
3012	YES	-	-	-	CCDS30777	ENSP00000	Q12882	-
3012	YES	-	-	-	CCDS30777	ENSP00000	Q12882	-
1090	YES	-	-	-	CCDS47443	ENSP00000	Q03001	Q86T18
1090	YES	-	-	-	CCDS47443	ENSP00000	Q03001	Q86T18
1090	YES	-	-	-	CCDS47443	ENSP00000	Q03001	Q86T18
1090	YES	-	-	-	CCDS47443	ENSP00000	Q03001	Q86T18
1090	YES	-	-	-	CCDS47443	ENSP00000	Q03001	Q86T18
1090	YES	-	-	-	CCDS47443	ENSP00000	Q03001	Q86T18
1090	YES	-	-	-	CCDS47443	ENSP00000	Q03001	Q86T18
1090	YES	-	-	-	CCDS47443	ENSP00000	Q03001	Q86T18
3373	YES	-	-	-	CCDS14010	ENSP00000	Q09472	B5A250
3373	YES	-	-	-	CCDS14010	ENSP00000	Q09472	B5A250
11958	YES	-	-	-	CCDS31929	ENSP00000	Q96L91	-
11958	YES	-	-	-	CCDS31929	ENSP00000	Q96L91	-
11958	YES	-	-	-	CCDS31929	ENSP00000	Q96L91	-
11958	YES	-	-	-	CCDS31929	ENSP00000	Q96L91	-
11958	YES	-	-	-	CCDS31929	ENSP00000	Q96L91	-
11958	YES	-	-	-	CCDS31929	ENSP00000	Q96L91	-
3387	YES	-	-	-	CCDS2922.	ENSP00000	P29320	-
3387	YES	-	-	-	CCDS2922.	ENSP00000	P29320	-
3387	YES	-	-	-	CCDS2922.	ENSP00000	P29320	-
3390	YES	-	-	-	CCDS5031.	ENSP00000	Q15375	-
3390	YES	-	-	-	CCDS5031.	ENSP00000	Q15375	-
3390	YES	-	-	-	CCDS5031.	ENSP00000	Q15375	-
3392	YES	-	-	-	CCDS46921	ENSP00000	P54762	C9K090,C9.
3430	YES	-	-	-	CCDS32642	ENSP00000	P04626	Q9NP09,J3
3430	YES	-	-	-	CCDS32642	ENSP00000	P04626	Q9NP09,J3
3430	YES	-	-	-	CCDS32642	ENSP00000	P04626	Q9NP09,J3
3431	YES	-	-	-	CCDS31833	ENSP00000	P21860	Q9NNX2,F8
3432	YES	-	-	-	CCDS2394.	ENSP00000	Q15303	Q580Q7,Q5
3432	YES	-	-	-	CCDS2394.	ENSP00000	Q15303	Q580Q7,Q5
3432	YES	-	-	-	CCDS2394.	ENSP00000	Q15303	Q580Q7,Q5
3446	YES	-	-	-	CCDS46648	ENSP00000	P11308	Q16031,B4
3490	YES	-	-	-	CCDS55088	ENSP00000	P50549	C9J9L1,C9J4
3493	YES	-	-	-	CCDS11465	ENSP00000	P43268	K7EMW0
3512	YES	-	-	-	CCDS6324.	ENSP00000	Q16394	T2FFJ4,S5Y

3512	YES	-	-	-	CCDS6324.	ENSP00000	Q16394	T2FFJ4,S5Y
3513	YES	-	-	-	CCDS5361	ENSP00000	Q93063	E9PNL9,E9
3527	YES	-	-	-	CCDS5891.	ENSP00000	Q15910	Q75MQ0,C
26837	YES	-	-	-	CCDS1437	ENSP00000	Q5JTC6	-
3582	YES	-	-	-	CCDS3251	ENSP00000	O15360	H3BT53
3582	YES	-	-	-	CCDS3251	ENSP00000	O15360	H3BT53
3585	YES	-	-	-	CCDS2595.	ENSP00000	Q9BXW9	-
3585	YES	-	-	-	CCDS2595.	ENSP00000	Q9BXW9	-
18794	YES	-	-	-	CCDS1219	ENSP00000	Q75N90	-
16712	YES	-	-	-	CCDS3777.	ENSP00000	Q969H0	S4R3N3,H9
3688	YES	-	-	-	CCDS5522	ENSP00000	P11362	E9PN14,D3
3689	YES	-	-	-	CCDS7620.	ENSP00000	P21802	Q9UMB0,C
3690	YES	-	-	-	CCDS5470	ENSP00000	P22607	Q8NI16
3690	YES	-	-	-	CCDS5470	ENSP00000	P22607	Q8NI16
3691	YES	-	-	-	CCDS4410.	ENSP00000	P22455	G3JVM2,E7
3765	YES	-	-	-	CCDS3195	ENSP00000	P36888	-
3767	YES	-	-	-	CCDS4457.	ENSP00000	P35916	D6RFF2
3778	YES	-	-	-	CCDS4281	ENSP00000	P02751	Q9H382,Q
3778	YES	-	-	-	CCDS4281	ENSP00000	P02751	Q9H382,Q
3778	YES	-	-	-	CCDS4281	ENSP00000	P02751	Q9H382,Q
3819	YES	-	-	-	CCDS9371.	ENSP00000	Q12778	-
3821	YES	-	-	-	CCDS5068.	ENSP00000	O43524	B4DVZ6
3823	YES	-	-	-	CCDS5883	ENSP00000	-	Q8IXF4,E9
20842	YES	-	-	-	CCDS3444	ENSP00000	Q8IVH2	Q8N4A5,Q
24824	YES	-	-	-	CCDS4591	ENSP00000	Q9UM11	-
4171	YES	-	-	-	CCDS3049.	ENSP00000	P23769	C9J965
4392	YES	-	-	-	CCDS4662	ENSP00000	Q5JWF2	S4R3W4,S4
17849	YES	-	-	-	CCDS6097.	ENSP00000	Q96PE1	-
24885	YES	-	-	-	CCDS4265	ENSP00000	Q4ZG55	-
4684	YES	-	-	-	CCDS5817	ENSP00000	P33402	-
23576	YES	-	-	-	CCDS6139.	ENSP00000	Q86VS8	-
5253	YES	-	-	-	CCDS3216	ENSP00000	P07900	Q96HX7,Q
5253	YES	-	-	-	CCDS3216	ENSP00000	P07900	Q96HX7,Q
5383	YES	-	-	-	CCDS1035	ENSP00000	P48735	H0YLL5,B4
5465	YES	-	-	-	CCDS1037	ENSP00000	P08069	H0YNR0,H
5465	YES	-	-	-	CCDS1037	ENSP00000	P08069	H0YNR0,H
5467	YES	-	-	-	CCDS5273.	ENSP00000	P11717	A0N9R7,A
5467	YES	-	-	-	CCDS5273.	ENSP00000	P11717	A0N9R7,A
5960	YES	-	-	-	CCDS6128.	ENSP00000	O14920	E5RGW5
14552	YES	-	-	-	CCDS3099	ENSP00000	Q14164	-
6021	YES	-	-	-	CCDS3971.	ENSP00000	P40189	-
6021	YES	-	-	-	CCDS3971.	ENSP00000	P40189	-
6021	YES	-	-	-	CCDS3971.	ENSP00000	P40189	-
6021	YES	-	-	-	CCDS3971.	ENSP00000	P40189	-
6021	YES	-	-	-	CCDS3971.	ENSP00000	P40189	-
6119	YES	-	-	-	CCDS4469.	ENSP00000	Q15306	-
6126	YES	-	-	-	CCDS9510.	ENSP00000	Q9Y4H2	Q9UP29,Q

6135	YES	-	-	-	CCDS918.1	ENSP00000	O75578	-
6135	YES	-	-	-	CCDS918.1	ENSP00000	O75578	-
6135	YES	-	-	-	CCDS918.1	ENSP00000	O75578	-
6135	YES	-	-	-	CCDS918.1	ENSP00000	O75578	-
6145	YES	-	-	-	CCDS2669.	ENSP00000	Q13797	-
6145	YES	-	-	-	CCDS2669.	ENSP00000	Q13797	-
6155	YES	-	-	-	CCDS1371	ENSP00000	P05107	Q96PT7,E7
6156	YES	-	-	-	CCDS1151	ENSP00000	P05106	F2X0V0,F1
6156	YES	-	-	-	CCDS1151	ENSP00000	P05106	F2X0V0,F1
6156	YES	-	-	-	CCDS1151	ENSP00000	P05106	F2X0V0,F1
6156	YES	-	-	-	CCDS1151	ENSP00000	P05106	F2X0V0,F1
6192	YES	-	-	-	CCDS6457.	ENSP00000	O60674	Q8IXP2,F5I
6192	YES	-	-	-	CCDS6457.	ENSP00000	O60674	Q8IXP2,F5I
13013	YES	-	-	-	CCDS6124.	ENSP00000	Q92794	A5PKX7
17582	YES	-	-	-	CCDS7345.	ENSP00000	Q8WYB5	-
17582	YES	-	-	-	CCDS7345.	ENSP00000	Q8WYB5	-
17582	YES	-	-	-	CCDS7345.	ENSP00000	Q8WYB5	-
17582	YES	-	-	-	CCDS7345.	ENSP00000	Q8WYB5	-
17582	YES	-	-	-	CCDS7345.	ENSP00000	Q8WYB5	-
11114	YES	-	-	-	CCDS1435	ENSP00000	P41229	-
12637	YES	-	-	-	CCDS1426	ENSP00000	O15550	Q68D33,Q
6307	YES	-	-	-	CCDS3497.	ENSP00000	P35968	B4DEK3
6307	YES	-	-	-	CCDS3497.	ENSP00000	P35968	B4DEK3
6307	YES	-	-	-	CCDS3497.	ENSP00000	P35968	B4DEK3
6307	YES	-	-	-	CCDS3497.	ENSP00000	P35968	B4DEK3
23177	YES	-	-	-	CCDS1223	ENSP00000	Q14145	K7ESE0,K7
2235	YES	-	-	-	CCDS7060.	ENSP00000	Q99612	Q7Z3W8
6407	YES	-	-	-	CCDS8703.	ENSP00000	P01116	Q9UM97,C
6597	YES	-	-	-	CCDS3927.	ENSP00000	P42702	D6RJ94,D6
6597	YES	-	-	-	CCDS3927.	ENSP00000	P42702	D6RJ94,D6
6597	YES	-	-	-	CCDS3927.	ENSP00000	P42702	D6RJ94,D6
6597	YES	-	-	-	CCDS3927.	ENSP00000	P42702	D6RJ94,D6
6597	YES	-	-	-	CCDS3927.	ENSP00000	P42702	D6RJ94,D6
20974	YES	-	-	-	CCDS5476	ENSP00000	-	Q4W5J9,E
20974	YES	-	-	-	CCDS5476	ENSP00000	-	Q4W5J9,E
6693	YES	-	-	-	CCDS2182.	ENSP00000	Q9NZR2	Q8WY27,Q
6693	YES	-	-	-	CCDS2182.	ENSP00000	Q9NZR2	Q8WY27,Q
6693	YES	-	-	-	CCDS2182.	ENSP00000	Q9NZR2	Q8WY27,Q
6693	YES	-	-	-	CCDS2182.	ENSP00000	Q9NZR2	Q8WY27,Q
6693	YES	-	-	-	CCDS2182.	ENSP00000	Q9NZR2	Q8WY27,Q
6693	YES	-	-	-	CCDS2182.	ENSP00000	Q9NZR2	Q8WY27,Q
6693	YES	-	-	-	CCDS2182.	ENSP00000	Q9NZR2	Q8WY27,Q
6693	YES	-	-	-	CCDS2182.	ENSP00000	Q9NZR2	Q8WY27,Q
6693	YES	-	-	-	CCDS2182.	ENSP00000	Q9NZR2	Q8WY27,Q
6693	YES	-	-	-	CCDS2182.	ENSP00000	Q9NZR2	Q8WY27,Q
6693	YES	-	-	-	CCDS2182.	ENSP00000	Q9NZR2	Q8WY27,Q
6693	YES	-	-	-	CCDS2182.	ENSP00000	Q9NZR2	Q8WY27,Q

6720	YES	-	-	-	CCDS33747	ENSP00000	P02788	C9J0S5,A8K
946	YES	-	-	-	CCDS33780	ENSP00000	Q96QZ7	-
16259	YES	-	-	-	CCDS44714	ENSP00000	Q8IZL2	-
16259	YES	-	-	-	CCDS44714	ENSP00000	Q8IZL2	-
16259	YES	-	-	-	CCDS44714	ENSP00000	Q8IZL2	-
6840	YES	-	-	-	CCDS10216	ENSP00000	Q02750	A4QPA9
6896	YES	-	-	-	CCDS31029	ENSP00000	Q9P0L2	B4DIB3
13538	YES	-	-	-	CCDS56097	ENSP00000	Q96L34	Q96GZ3,K7
26530	YES	-	-	-	CCDS11940	ENSP00000	Q96M91	-
6916	YES	-	-	-	CCDS59320	ENSP00000	-	K7ESN0
6943	YES	-	-	-	CCDS957.1	ENSP00000	Q07820	B4DG83
7010	YES	-	-	-	CCDS8083.	ENSP00000	O00255	Q9GZQ5
7029	YES	-	-	-	CCDS47689	ENSP00000	P08581	Q9UEJ3,B4
7029	YES	-	-	-	CCDS47689	ENSP00000	P08581	Q9UEJ3,B4
7105	YES	-	-	-	CCDS43106	ENSP00000	O75030	C9K0S7
7105	YES	-	-	-	CCDS43106	ENSP00000	O75030	C9K0S7
7127	YES	-	-	-	CCDS2663.	ENSP00000	P40692	Q5GJ64,F2
7132	YES	-	-	-	CCDS55791	ENSP00000	Q03164	Q9UPD0,Q
7132	YES	-	-	-	CCDS55791	ENSP00000	Q03164	Q9UPD0,Q
7133	YES	-	-	-	CCDS44873	ENSP00000	O14686	Q6PIA1,Q5
13726	YES	-	-	-	CCDS5931.	ENSP00000	Q8NEZ4	Q6N019,Q
13726	YES	-	-	-	CCDS5931.	ENSP00000	Q8NEZ4	Q6N019,Q
13726	YES	-	-	-	CCDS5931.	ENSP00000	Q8NEZ4	Q6N019,Q
13726	YES	-	-	-	CCDS5931.	ENSP00000	Q8NEZ4	Q6N019,Q
13726	YES	-	-	-	CCDS5931.	ENSP00000	Q8NEZ4	Q6N019,Q
13726	YES	-	-	-	CCDS5931.	ENSP00000	Q8NEZ4	Q6N019,Q
13726	YES	-	-	-	CCDS5931.	ENSP00000	Q8NEZ4	Q6N019,Q
16063	YES	-	-	-	CCDS55708	ENSP00000	P55197	Q71UR7,B4
16063	YES	-	-	-	CCDS55708	ENSP00000	P55197	Q71UR7,B4
16063	YES	-	-	-	CCDS55708	ENSP00000	P55197	Q71UR7,B4
7217	YES	-	-	-	CCDS483.1	ENSP00000	P40238	-
7217	YES	-	-	-	CCDS483.1	ENSP00000	P40238	-
7230	YES	-	-	-	CCDS8299.	ENSP00000	P49959	Q9BS79,F5
7325	YES	-	-	-	CCDS1834.	ENSP00000	P43246	Q53RU4,C9
7325	YES	-	-	-	CCDS1834.	ENSP00000	P43246	Q53RU4,C9
7325	YES	-	-	-	CCDS1834.	ENSP00000	P43246	Q53RU4,C9
7325	YES	-	-	-	CCDS1834.	ENSP00000	P43246	Q53RU4,C9
3942	YES	-	-	-	CCDS127.1	ENSP00000	P42345	Q96QW8,B
7468	YES	-	-	-	CCDS1614.	ENSP00000	Q99707	-
7468	YES	-	-	-	CCDS1614.	ENSP00000	Q99707	-
7468	YES	-	-	-	CCDS1614.	ENSP00000	Q99707	-
7468	YES	-	-	-	CCDS1614.	ENSP00000	Q99707	-
7508	YES	-	-	-	CCDS55640	ENSP00000	-	Q9UMI8,Q
7508	YES	-	-	-	CCDS55640	ENSP00000	-	Q9UMI8,Q
7527	YES	-	-	-	CCDS520.1	ENSP00000	Q9UIF7	E5KP26,Q8
7553	YES	-	-	-	CCDS6359.	ENSP00000	P01106	Q6LBK7,B4
7553	YES	-	-	-	CCDS6359.	ENSP00000	P01106	Q6LBK7,B4

7555	YES	-	-	-	CCDS5330(	ENSP0000(	-	Q86YL9,E9I
7559	YES	-	-	-	CCDS1687.	ENSP0000(	P04198	Q9UMQ5,C
7569	YES	-	-	-	CCDS4542З	ENSP0000(	P35749	Q68D89,Q6
7569	YES	-	-	-	CCDS4542З	ENSP0000(	P35749	Q68D89,Q6
7579	YES	-	-	-	CCDS13927	ENSP0000(	P35579	Q9UMJ0,B1
7669	YES	-	-	-	CCDS47872	ENSP0000(	Q15596	-
7669	YES	-	-	-	CCDS47872	ENSP0000(	Q15596	-
7669	YES	-	-	-	CCDS47872	ENSP0000(	Q15596	-
7669	YES	-	-	-	CCDS47872	ENSP0000(	Q15596	-
7669	YES	-	-	-	CCDS47872	ENSP0000(	Q15596	-
7671	YES	-	-	-	CCDS44394	ENSP0000(	Q13772	-
7671	YES	-	-	-	CCDS44394	ENSP0000(	Q13772	-
7765	YES	-	-	-	CCDS42292	ENSP0000(	P21359	Q9UMU3,C
7765	YES	-	-	-	CCDS42292	ENSP0000(	P21359	Q9UMU3,C
7765	YES	-	-	-	CCDS42292	ENSP0000(	P21359	Q9UMU3,C
7782	YES	-	-	-	CCDS42782	ENSP0000(	Q16236	Q9BZF8,K7
7794	YES	-	-	-	CCDS3657.	ENSP0000(	P19838	D6RF93,D6
7794	YES	-	-	-	CCDS3657.	ENSP0000(	P19838	D6RF93,D6
7794	YES	-	-	-	CCDS3657.	ENSP0000(	P19838	D6RF93,D6
7794	YES	-	-	-	CCDS3657.	ENSP0000(	P19838	D6RF93,D6
7795	YES	-	-	-	CCDS41564	ENSP0000(	Q00653	M0R119,M
14906	YES	-	-	-	CCDS32079	ENSP0000(	Q8N4C6	H7C162,E9
14906	YES	-	-	-	CCDS32079	ENSP0000(	Q8N4C6	H7C162,E9
14374	YES	-	-	-	CCDS42246	ENSP0000(	Q9C000	I3L2G5,I3L(
7882	YES	-	-	-	CCDS908.1	ENSP0000(	Q04721	Q9UFD5,Q1
7882	YES	-	-	-	CCDS908.1	ENSP0000(	Q04721	Q9UFD5,Q1
7884	YES	-	-	-	CCDS3442(	ENSP0000(	Q99466	-
7884	YES	-	-	-	CCDS3442(	ENSP0000(	Q99466	-
14234	YES	-	-	-	CCDS4412.	ENSP0000(	Q96L73	Q9H6H8,Q9
6093	YES	-	-	-	CCDS1160.	ENSP0000(	P14616	I6U4P1
8031	YES	-	-	-	CCDS1161.	ENSP0000(	P04629	-
8033	YES	-	-	-	CCDS32322	ENSP0000(	Q16288	R4GNH5
8064	YES	-	-	-	CCDS6940.	ENSP0000(	P35658	H0YDI2,E9I
8068	YES	-	-	-	CCDS7746.	ENSP0000(	P52948	Q9HDC8
8592	YES	-	-	-	CCDS48151	ENSP0000(	O75914	B1AKS5
8619	YES	-	-	-	CCDS6607.	ENSP0000(	Q02548	E7ERK2,C0I
8621	YES	-	-	-	CCDS186.1	ENSP0000(	P23759	-
30064	YES	-	-	-	CCDS43099	ENSP0000(	Q86U86	C9JQF1,C9J
30064	YES	-	-	-	CCDS43099	ENSP0000(	Q86U86	C9JQF1,C9J
15580	YES	-	-	-	CCDS55627	ENSP0000(	Q5VU43	I1VE15
15580	YES	-	-	-	CCDS55627	ENSP0000(	Q5VU43	I1VE15
15580	YES	-	-	-	CCDS55627	ENSP0000(	Q5VU43	I1VE15
15580	YES	-	-	-	CCDS55627	ENSP0000(	Q5VU43	I1VE15
15580	YES	-	-	-	CCDS55627	ENSP0000(	Q5VU43	I1VE15
15580	YES	-	-	-	CCDS55627	ENSP0000(	Q5VU43	I1VE15
15580	YES	-	-	-	CCDS55627	ENSP0000(	Q5VU43	I1VE15
15580	YES	-	-	-	CCDS55627	ENSP0000(	Q5VU43	I1VE15

15580	YES	-	-	-	CCDS55627	ENSP00000(	Q5VU43	I1VE15
15580	YES	-	-	-	CCDS55627	ENSP00000(	Q5VU43	I1VE15
15580	YES	-	-	-	CCDS55627	ENSP00000(	Q5VU43	I1VE15
15580	YES	-	-	-	CCDS55627	ENSP00000(	Q5VU43	I1VE15
15580	YES	-	-	-	CCDS55627	ENSP00000(	Q5VU43	I1VE15
15580	YES	-	-	-	CCDS55627	ENSP00000(	Q5VU43	I1VE15
15580	YES	-	-	-	CCDS55627	ENSP00000(	Q5VU43	I1VE15
15580	YES	-	-	-	CCDS55627	ENSP00000(	Q5VU43	I1VE15
15580	YES	-	-	-	CCDS55627	ENSP00000(	Q5VU43	I1VE15
15580	YES	-	-	-	CCDS55627	ENSP00000(	Q5VU43	I1VE15
15580	YES	-	-	-	CCDS55627	ENSP00000(	Q5VU43	I1VE15
15580	YES	-	-	-	CCDS55627	ENSP00000(	Q5VU43	I1VE15
15580	YES	-	-	-	CCDS55627	ENSP00000(	Q5VU43	I1VE15
8803	YES	-	-	-	CCDS3495.	ENSP00000(	P16234	D6RIG5,D6
8803	YES	-	-	-	CCDS3495.	ENSP00000(	P16234	D6RIG5,D6
8804	YES	-	-	-	CCDS4303.	ENSP00000(	P09619	E5RJ14,E5F
8804	YES	-	-	-	CCDS4303.	ENSP00000(	P09619	E5RJ14,E5F
8845	YES	-	-	-	CCDS11131	ENSP00000(	O15534	J3QLQ5,A2
8972	YES	-	-	-	CCDS1446.	ENSP00000(	O00750	Q5SW98,Q
8975	YES	-	-	-	CCDS43171	ENSP00000(	P42336	Q4LE51,C9.
8975	YES	-	-	-	CCDS43171	ENSP00000(	P42336	Q4LE51,C9.
8976	YES	-	-	-	CCDS3104.	ENSP00000(	P42338	Q9BTS4,Q6
8978	YES	-	-	-	CCDS5739.	ENSP00000(	P48736	Q24M88,E!
8979	YES	-	-	-	CCDS3993.	ENSP00000(	P27986	J7GXU7,J7(
8979	YES	-	-	-	CCDS3993.	ENSP00000(	P27986	J7GXU7,J7(
9016	YES	-	-	-	CCDS4935.	ENSP00000(	P08F94	-
9016	YES	-	-	-	CCDS4935.	ENSP00000(	P08F94	-
9016	YES	-	-	-	CCDS4935.	ENSP00000(	P08F94	-
9016	YES	-	-	-	CCDS4935.	ENSP00000(	P08F94	-
9016	YES	-	-	-	CCDS4935.	ENSP00000(	P08F94	-
9016	YES	-	-	-	CCDS4935.	ENSP00000(	P08F94	-
9121	YES	-	-	-	CCDS2302.	ENSP00000(	P54277	Q5FBZ2,C9.
9121	YES	-	-	-	CCDS2302.	ENSP00000(	P54277	Q5FBZ2,C9.
9122	YES	-	-	-	CCDS5343.	ENSP00000(	P54278	-
9122	YES	-	-	-	CCDS5343.	ENSP00000(	P54278	-
9122	YES	-	-	-	CCDS5343.	ENSP00000(	P54278	-
17284	YES	-	-	-	CCDS5793.	ENSP00000(	Q9NUX5	C9JPG9,A8I
9388	YES	-	-	-	CCDS11678	ENSP00000(	P10644	Q96P62,K7
9413	YES	-	-	-	-	ENSP00000(	P78527	F5GX40
9413	YES	-	-	-	-	ENSP00000(	P78527	F5GX40
9413	YES	-	-	-	-	ENSP00000(	P78527	F5GX40
9413	YES	-	-	-	-	ENSP00000(	P78527	F5GX40
9413	YES	-	-	-	-	ENSP00000(	P78527	F5GX40
9585	YES	-	-	-	CCDS6714.	ENSP00000(	Q13635	Q6TKQ0,F8
9585	YES	-	-	-	CCDS6714.	ENSP00000(	Q13635	Q6TKQ0,F8
9605	YES	-	-	-	CCDS1371.	ENSP00000(	P35354	D9MWI3
9605	YES	-	-	-	CCDS1371.	ENSP00000(	P35354	D9MWI3

9644	YES	-	-	-	CCDS9163.	ENSP00000	Q06124	B3GUD4,B3
9668	YES	-	-	-	CCDS4378	ENSP00000	P23468	C9J6E4,B4D
9668	YES	-	-	-	CCDS4378	ENSP00000	P23468	C9J6E4,B4D
9668	YES	-	-	-	CCDS4378	ENSP00000	P23468	C9J6E4,B4D
9682	YES	-	-	-	CCDS4287	ENSP00000	O14522	-
9816	YES	-	-	-	CCDS3423	ENSP00000	Q92878	C9JNH8,A8
9829	YES	-	-	-	CCDS2612.	ENSP00000	P04049	L7RRS6
9829	YES	-	-	-	CCDS2612.	ENSP00000	P04049	L7RRS6
9829	YES	-	-	-	CCDS2612.	ENSP00000	P04049	L7RRS6
9842	YES	-	-	-	CCDS6959.	ENSP00000	Q12967	Q9HAY0,Q
9864	YES	-	-	-	CCDS1136	ENSP00000	P10276	Q6I9R7,J3
4552	YES	-	-	-	CCDS6430.	ENSP00000	P24298	-
9954	YES	-	-	-	CCDS1864.	ENSP00000	Q04864	-
9954	YES	-	-	-	CCDS1864.	ENSP00000	Q04864	-
10061	YES	-	-	-	CCDS1365.	ENSP00000	Q99496	-
10061	YES	-	-	-	CCDS1365.	ENSP00000	Q99496	-
14539	YES	-	-	-	CCDS5860	ENSP00000	Q63HN8	H3BLU6
14539	YES	-	-	-	CCDS5860	ENSP00000	Q63HN8	H3BLU6
14539	YES	-	-	-	CCDS5860	ENSP00000	Q63HN8	H3BLU6
14539	YES	-	-	-	CCDS5860	ENSP00000	Q63HN8	H3BLU6
14539	YES	-	-	-	CCDS5860	ENSP00000	Q63HN8	H3BLU6
14539	YES	-	-	-	CCDS5860	ENSP00000	Q63HN8	H3BLU6
10261	YES	-	-	-	CCDS5116.	ENSP00000	P08922	-
10431	YES	-	-	-	CCDS3457	ENSP00000	Q15349	D6RHW7,D
1535	YES	-	-	-	CCDS5654	ENSP00000	Q06455	Q9HBV9,E5
1535	YES	-	-	-	CCDS5654	ENSP00000	Q06455	Q9HBV9,E5
1535	YES	-	-	-	CCDS5654	ENSP00000	Q06455	Q9HBV9,E5
10680	YES	-	-	-	CCDS3853.	ENSP00000	P31040	Q0QF12,B3
18420	YES	-	-	-	CCDS2749.	ENSP00000	Q9BYW2	C9JG86
18420	YES	-	-	-	CCDS2749.	ENSP00000	Q9BYW2	C9JG86
10768	YES	-	-	-	CCDS3335	ENSP00000	O75533	Q9NTB4,F8
10810	YES	-	-	-	CCDS4747	ENSP00000	O00141	E9PP33,E9
10810	YES	-	-	-	CCDS4747	ENSP00000	O00141	E9PP33,E9
6770	YES	-	-	-	CCDS1195	ENSP00000	Q13485	Q9BYG6,K7
6770	YES	-	-	-	CCDS1195	ENSP00000	Q13485	Q9BYG6,K7
11100	YES	-	-	-	CCDS1225	ENSP00000	P51532	B4DSI8,A7
11103	YES	-	-	-	CCDS1381	ENSP00000	Q12824	-
11103	YES	-	-	-	CCDS1381	ENSP00000	Q12824	-
11191	YES	-	-	-	CCDS1654.	ENSP00000	P35716	Q05CH0
11195	YES	-	-	-	CCDS3239.	ENSP00000	P48431	-
11283	YES	-	-	-	CCDS1329	ENSP00000	P12931	Q9H7V3,Q
11283	YES	-	-	-	CCDS1329	ENSP00000	P12931	Q9H7V3,Q
17209	YES	-	-	-	CCDS2421.	ENSP00000	Q9NRP7	C9JDA4,C9
16466	YES	-	-	-	CCDS7537.	ENSP00000	Q9UMX1	-
11491	YES	-	-	-	CCDS6688.	ENSP00000	P43405	-
17089	YES	-	-	-	CCDS5236.	ENSP00000	Q8NF91	-
17089	YES	-	-	-	CCDS5236.	ENSP00000	Q8NF91	-

17089	YES	-	-	-	CCDS5236.	ENSP00000(	Q8NF91	-
17089	YES	-	-	-	CCDS5236.	ENSP00000(	Q8NF91	-
17089	YES	-	-	-	CCDS5236.	ENSP00000(	Q8NF91	-
17089	YES	-	-	-	CCDS5236.	ENSP00000(	Q8NF91	-
17089	YES	-	-	-	CCDS5236.	ENSP00000(	Q8NF91	-
17089	YES	-	-	-	CCDS5236.	ENSP00000(	Q8NF91	-
17089	YES	-	-	-	CCDS5236.	ENSP00000(	Q8NF91	-
17089	YES	-	-	-	CCDS5236.	ENSP00000(	Q8NF91	-
17089	YES	-	-	-	CCDS5236.	ENSP00000(	Q8NF91	-
17089	YES	-	-	-	CCDS5236.	ENSP00000(	Q8NF91	-
17089	YES	-	-	-	CCDS5236.	ENSP00000(	Q8NF91	-
11535	YES	-	-	-	CCDS1441	ENSP00000(	P21675	-
11535	YES	-	-	-	CCDS1441	ENSP00000(	P21675	-
18056	YES	-	-	-	CCDS3500	ENSP00000(	Q8IZX4	-
11600	YES	-	-	-	CCDS1444	ENSP00000(	Q9Y458	C3TX51
11633	YES	-	-	-	CCDS1207	ENSP00000(	P15923	Q6PJU3,K7
11640	YES	-	-	-	CCDS1971.	ENSP00000(	Q9HCS4	Q53T87,C9
11641	YES	-	-	-	CCDS5357	ENSP00000(	Q9NQB0	E2GH26,C6
11641	YES	-	-	-	CCDS5357	ENSP00000(	Q9NQB0	E2GH26,C6
29484	YES	-	-	-	CCDS7281.	ENSP00000(	Q8NFU7	-
29484	YES	-	-	-	CCDS7281.	ENSP00000(	Q8NFU7	-
29484	YES	-	-	-	CCDS7281.	ENSP00000(	Q8NFU7	-
25941	YES	-	-	-	CCDS4712(	ENSP00000(	Q6N021	E7EPB1,D6
25941	YES	-	-	-	CCDS4712(	ENSP00000(	Q6N021	E7EPB1,D6
11752	YES	-	-	-	CCDS1431	ENSP00000(	P19532	B4DIA5
11785	YES	-	-	-	CCDS3219	ENSP00000(	P07996	Q7KYY3,A8
11998	YES	-	-	-	CCDS1111	ENSP00000(	P04637	S5LQU8,Q7
12017	YES	-	-	-	CCDS4144	ENSP00000(	P12270	Q9UE33
12017	YES	-	-	-	CCDS4144	ENSP00000(	P12270	Q9UE33
12017	YES	-	-	-	CCDS4144	ENSP00000(	P12270	Q9UE33
16290	YES	-	-	-	CCDS872.1	ENSP00000(	Q9UPN9	-
16290	YES	-	-	-	CCDS872.1	ENSP00000(	Q9UPN9	-
12305	YES	-	-	-	CCDS9899.	ENSP00000(	Q15643	G3V4R7
12305	YES	-	-	-	CCDS9899.	ENSP00000(	Q15643	G3V4R7
12305	YES	-	-	-	CCDS9899.	ENSP00000(	Q15643	G3V4R7
12347	YES	-	-	-	CCDS5906	ENSP00000(	Q9Y4A5	C9K0N1
12347	YES	-	-	-	CCDS5906	ENSP00000(	Q9Y4A5	C9K0N1
12347	YES	-	-	-	CCDS5906	ENSP00000(	Q9Y4A5	C9K0N1
12347	YES	-	-	-	CCDS5906	ENSP00000(	Q9Y4A5	C9K0N1
12347	YES	-	-	-	CCDS5906	ENSP00000(	Q9Y4A5	C9K0N1
12363	YES	-	-	-	CCDS1045	ENSP00000(	P49815	-
12373	YES	-	-	-	CCDS9872.	ENSP00000(	-	Q0VAP8,F5
12373	YES	-	-	-	CCDS9872.	ENSP00000(	-	Q0VAP8,F5
12373	YES	-	-	-	CCDS9872.	ENSP00000(	-	Q0VAP8,F5
16806	YES	-	-	-	CCDS3493	ENSP00000(	O95071	Q49A65,E5
16806	YES	-	-	-	CCDS3493	ENSP00000(	O95071	Q49A65,E5
16806	YES	-	-	-	CCDS3493	ENSP00000(	O95071	Q49A65,E5

16806	YES	-	-	-	CCDS34933	ENSP00000	O95071	Q49A65,E5
16806	YES	-	-	-	CCDS34933	ENSP00000	O95071	Q49A65,E5
16806	YES	-	-	-	CCDS34933	ENSP00000	O95071	Q49A65,E5
12632	YES	-	-	-	CCDS43930	ENSP00000	Q93008	-
12766	YES	-	-	-	CCDS33940	ENSP00000	O96028	D6RIS1,D6I
12766	YES	-	-	-	CCDS33940	ENSP00000	O96028	D6RIS1,D6I
12766	YES	-	-	-	CCDS33940	ENSP00000	O96028	D6RIS1,D6I
12766	YES	-	-	-	CCDS33940	ENSP00000	O96028	D6RIS1,D6I
12766	YES	-	-	-	CCDS33940	ENSP00000	O96028	D6RIS1,D6I
12791	YES	-	-	-	CCDS6082.	ENSP00000	Q14191	-
12791	YES	-	-	-	CCDS6082.	ENSP00000	Q14191	-
12791	YES	-	-	-	CCDS6082.	ENSP00000	Q14191	-
12791	YES	-	-	-	CCDS6082.	ENSP00000	Q14191	-
11955	YES	-	-	-	CCDS44817	ENSP00000	Q8TF68	F5H3Z9,F5I
11955	YES	-	-	-	CCDS44817	ENSP00000	Q8TF68	F5H3Z9,F5I

UNIPARC	SIFT	PolyPhen	DOMAINS	HGVS_OFFS	AF	AFR_AF	AMR_AF	EAS_AF
UPI000012	-	-	Superfamily	-				
UPI000045	-	-	-	-				
UPI000013	-	-	-	-				
UPI000013	-	-	Pfam_dom	-				
UPI000002	deleterious	possibly_da	Coiled-coils	-				
UPI000002	-	-	-	-				
UPI000002	deleterious	possibly_da	Coiled-coils	-				
UPI000002	-	-	-	-				
UPI000002	-	-	Coiled-coils	6				
UPI000002	deleterious	possibly_da	hmmpanth	-				
UPI000002	tolerated(0	benign(0.0	hmmpanth	-				
UPI000004	tolerated(0	benign(0.0	Gene3D:1.	-				
UPI000003	-	-	-	-				
UPI000016	-	-	Pfam_dom	-				
UPI000013	tolerated(0	benign(0.0	hmmpanth	-				
UPI000013	-	-	hmmpanth	1				
UPI000013	-	-	hmmpanth	-				
UPI000013	-	-	hmmpanth	2				
UPI000013	-	-	Low_comp	4				
UPI000016	-	-	Gene3D:1.	2				
UPI000016	-	-	hmmpanth	-				
UPI000016	deleterious	probably_c	hmmpanth	-				
UPI000016	-	-	Gene3D:1.	-				
UPI000016	-	-	hmmpanth	3				
UPI000016	deleterious	probably_c	Pfam_dom	-				
UPI00001D	tolerated(1	benign(0.0	hmmpanth	-				
UPI000003	-	-	-	-				
UPI0000DE	-	-	-	-				
UPI0000DE	-	-	-	-				
UPI0000DE	-	-	Superfamily	-				
UPI000003	-	-	-	-				
UPI000003	-	-	-	-				
UPI000003	-	-	hmmpanth	-				
UPI000003	tolerated(0	benign(0)	hmmpanth	-				
UPI000003	-	-	-	-				
UPI000003	-	-	-	-				
UPI000016	-	-	-	-				
UPI000013	-	-	hmmpanth	-				
UPI000013	-	-	Gene3D:1.	-				
UPI000013	-	-	-	-				
UPI00001A	tolerated(0	benign(0.1	-	-				
UPI00001A	tolerated(0	benign(0.0	-	-				
UPI000012	-	-	-	-				
UPI000012	-	-	-	-				
UPI000013	-	-	-	-				

UPI000012	tolerated(0	benign(0)	hmmpanth	-	
UPI000013	-	-	hmmpanth	-	
UPI000013	-	-	-	-	
UPI000000	tolerated(0	benign(0.0	Gene3D:1.1	-	
UPI000000	tolerated(0	benign(0)	Gene3D:1.1	-	
UPI000000	-	-	Coiled-coils	-	
UPI000000	tolerated(0	benign(0.2	hmmpanth	-	
UPI000000	deleterious	probably_c	hmmpanth	-	
UPI000013	-	-	-	-	
UPI000013	-	-	-	-	
UPI000012	tolerated(0	benign(0.0	-	-	
UPI000012	deleterious	benign(0.1	PROSITE_p	-	
UPI000006	-	-	-	-	
UPI000013	-	-	-	-	
UPI000013	-	-	-	-	
UPI000013	-	-	-	-	
UPI00003F	tolerated(0	benign(0.1	hmmpanth	-	
UPI0000E5	tolerated(0	benign(0.1	hmmpanth	-	
UPI0000E5	-	-	-	4	
UPI000013	-	-	-		10
UPI000002	-	-	-	-	
UPI000002	-	-	-	-	
UPI000003	-	-	-	-	
UPI000013	-	-	-	-	
UPI000013	-	-	-	-	
UPI000013	tolerated(0	benign(0)	hmmpanth	-	
UPI000013	tolerated(0	benign(0.0	Gene3D:4.1	-	
UPI000013	tolerated_l	benign(0.1	Low_comp	-	
UPI000011	-	-	-	-	
UPI000045	-	-	-	-	
UPI000013	-	-	hmmpanth	-	
UPI000013	-	-	hmmpanth	-	
UPI000013	-	-	-		-1
UPI000006	deleterious	benign(0.0	hmmpanth	-	
UPI000006	-	-	-	-	
UPI000000	-	-	hmmpanth	-	
UPI00001E	-	-	-	-	
UPI00001E	-	-	-	-	
UPI00001E	-	-	-	-	
UPI00001E	-	-	-	-	
UPI00001E	-	-	-	-	
UPI000012	tolerated(0	benign(0.0	hmmpanth	-	
UPI000012	-	-	hmmpanth	5	
UPI000012	deleterious	probably_c	Gene3D:1.1	-	
UPI000013	tolerated(0	benign(0.0	Gene3D:1.1	-	
UPI000015	tolerated(0	benign(0.0	hmmpanth	-	
UPI00001A	tolerated(0	benign(0.0	Low_comp	-	

UPI00001A	deleterious	possibly_da	PROSITE_p	-
UPI00001A	deleterious	possibly_da	hmmpanth	-
UPI00001A	-	-	-	-
UPI000013	-	-	-	-
UPI000013	-	-	-	-
UPI000013	deleterious	benign(0.3(	Gene3D:1.:	-
UPI000016	-	-	hmmpanth	-
UPI000000	-	-	-	-
UPI000000	tolerated_l	probably_c	hmmpanth	-
UPI000045	-	-	-	-
UPI000045	-	-	-	-
UPI000045	-	-	-	-
UPI000045	-	-	-	-
UPI00001C	deleterious	possibly_da	Gene3D:1.:	-
UPI00001C	deleterious	probably_c	Gene3D:1.:	-
UPI00001C	-	-	Gene3D:1.:	-
UPI00001C	deleterious	benign(0.0!	Gene3D:1.:	-
UPI00001C	deleterious	probably_c	hmmpanth	-
UPI00001C	-	-	-	-
UPI00001C	-	-	-	-
UPI00001C	-	-	-	-
UPI00001A	-	-	-	6
UPI00001A	-	-	-	-
UPI000045	deleterious	probably_c	hmmpanth	-
UPI000045	tolerated(C	benign(0.0:	hmmpanth	-
UPI000045	-	-	PROSITE_p	-
UPI000045	-	-	-	-
UPI000045	deleterious	probably_c	hmmpanth	-
UPI000045	tolerated_l	benign(0.0:	hmmpanth	-
UPI000016	-	-	-	-
UPI000016	-	-	-	-
UPI000016	tolerated(C	benign(0.2(	hmmpanth	-
UPI000004	-	-	-	-
UPI000004	-	-	-	-
UPI000004	-	-	-	-
UPI000012	-	-	hmmpanth	-
UPI000003	-	-	-	-
UPI000003	-	-	-	-
UPI000003	-	-	PIRSF_dom	-
UPI000012	-	-	-	-
UPI000004	-	-	PIRSF_dom	-
UPI000004	-	-	-	-
UPI000004	-	-	-	-
UPI000018	-	-	hmmpanth	-
UPI000012	-	-	-	-
UPI000000	tolerated(C	benign(0.0(	Pfam_dom	-
UPI000012	deleterious	probably_c	Superfamil	-

UPI000012	-	-	Pfam_dom	-
UPI0000EE	-	-	-	5
UPI000006	-	-	-	-
UPI0000ED	-	-	Pfam_dom	-
UPI000052	-	-	-	-
UPI000052	-	-	-	-
UPI000006	deleterious	probably_c	hmmpanth	-
UPI000006	-	-	hmmpanth	6
UPI000013	-	-	-	-
UPI000007	-	-	SMART_do	-
UPI0001CE	-	-	PROSITE_p	-
UPI000002	deleterious	probably_c	PROSITE_p	-
UPI000002	-	-	-	2
UPI000002	-	-	Gene3D:1.:	4
UPI000012	-	-	Low_comp	-
UPI00001F	-	-	-	-1
UPI000014	tolerated(0	benign(0.0:	-	-
UPI0000E5	-	-	-	-
UPI0000E5	tolerated(0	possibly_da	hmmpanth	-
UPI0000E5	-	-	-	-
UPI000013	tolerated(0	probably_c	Low_comp	-
UPI000012	tolerated(0	benign(0.0(	hmmpanth	-
UPI000020	-	-	-	-
UPI000007	deleterious	probably_c	Superfamil	-
UPI00001D	-	-	-	-
UPI000007	-	-	PROSITE_p	-
UPI0000E4	-	-	Low_comp	-
UPI00004A	-	-	-	-
UPI000016	deleterious	probably_c	hmmpanth	-
UPI000002	-	-	Gene3D:3.:	-
UPI000006	-	-	-	8
UPI000040	tolerated_l	benign(0.2	hmmpanth	-
UPI000040	-	-	-	-
UPI000012	deleterious	possibly_da	hmmpanth	-
UPI000012	-	-	-	-
UPI000012	-	-	-	-
UPI000007	-	-	-	-
UPI000007	tolerated(0	benign(0.0:	hmmpanth	-
UPI000003	-	-	-	-
UPI000003	-	-	-	-
UPI000013	tolerated(0	benign(0.0(	-	-
UPI000013	tolerated(0	possibly_da	hmmpanth	-
UPI000013	-	-	-	-5
UPI000013	tolerated(0	benign(0.2!	PROSITE_p	-
UPI000013	-	-	-	-
UPI000012	-	-	hmmpanth	-
UPI000006	-	-	hmmpanth	-

UPI000013	-	-	Gene3D:3n	-	
UPI000013	-	-	-	-	
UPI000013	deleterious	probably_c	Pfam_dom	-	
UPI000013	-	-	Gene3D:3n	3	
UPI00001A	-	-	-	-	
UPI00001A	-	-	-	-	
UPI000016	-	-	-	-	
UPI000013	tolerated(0	possibly_d	hmmpanth	-	
UPI000013	-	-	-	-	
UPI000013	deleterious	probably_c	hmmpanth	-	
UPI000013	tolerated(0	benign(0.3	hmmpanth	-	
UPI000012	-	-	PIRSF_dom	-	
UPI000012	-	-	-	-	
UPI000013	-	-	hmmpanth	-	
UPI000013	-	-	hmmpanth		5
UPI000013	tolerated(0	benign(0.3	hmmpanth	-	
UPI000013	-	-	hmmpanth	5	
UPI000013	deleterious	probably_c	hmmpanth	-	
UPI000013	tolerated(0	probably_c	hmmpanth	-	
UPI000013	tolerated(0	benign(0.0	hmmpanth	-	
UPI000013	-	-	-	-	
UPI000003	-	-	-	-	
UPI000003	tolerated(0	benign(0.0	PROSITE_p	-	
UPI000003	tolerated(0	probably_c	PROSITE_p	-	
UPI000003	-	-	hmmpanth	-	
UPI000007	deleterious	probably_c	hmmpanth	-	
UPI000000	tolerated(0	benign(0.0	hmmpanth	-	
UPI000013	-	-	Gene3D:3.	-	
UPI000004	tolerated(0	benign(0.0	hmmpanth	-	
UPI000004	-	-	-	-	
UPI000004	-	-	-	-	
UPI000004	-	-	-	-	
UPI000004	tolerated(0	benign(0.0	Superfamil	-	
UPI000162	deleterious	probably_c	PROSITE_p	-	
UPI000162	deleterious	probably_c	PROSITE_p	-	
UPI00001B	-	-	-	-	
UPI00001B	-	-	-	-	
UPI00001B	-	-	Pfam_dom	-	
UPI00001B	-	-	-	-	
UPI00001B	tolerated(0	benign(0)	hmmpanth	-	
UPI00001B	tolerated(0	possibly_d	PROSITE_p	-	
UPI00001B	-	-	-	-	
UPI00001B	tolerated(0	probably_c	PROSITE_p	-	
UPI00001B	-	-	PROSITE_p	-	
UPI00001B	-	-	-	-	
UPI00001B	-	-	-	-	
UPI00001B	-	-	-	-	

UPI000016	-	-	-	-	
UPI000045	-	-	hmmpanth	-	
UPI00001B	tolerated(C	benign(0)	hmmpanth	-	
UPI00001B	-	-	hmmpanth	-	
UPI00001B	deleterious	probably_c	hmmpanth	-	
UPI000013	tolerated(C	benign(0.0(	Gene3D:1.:	-	
UPI000004	-	-	-	-	
UPI000004	deleterious	benign(0.0:	PROSITE_p	-	
UPI000014	-	-	-	-	
UPI0001F9	-	-	-	-	
UPI000006	deleterious	probably_c	PROSITE_p	-	
UPI000024	-	-	hmmpanth	-	
UPI000014	deleterious	possibly_da	PROSITE_p	-	
UPI000014	deleterious	probably_c	hmmpanth	-	
UPI000002	-	-	-	-	
UPI000002	-	-	-	-	
UPI000000	deleterious	probably_c	hmmpanth	-	
UPI0001E5	deleterious	benign(0.3·	PIRSF_dom	-	
UPI0001E5	tolerated(C	probably_c	PIRSF_dom	-	
UPI0000EE	-	-	-	-	
UPI000014	-	-	hmmpanth	-	
UPI000014	-	-	hmmpanth	-	
UPI000014	-	-	-	-	
UPI000014	-	-	-	-	
UPI000014	-	-	-	-	
UPI000014	tolerated_l	benign(0.0(	-	-	
UPI000014	-	-	-	-	
UPI00001F	-	-	-		5
UPI00001F	-	-	-	-	
UPI00001F	-	-	-	-	
UPI000002	-	-	-	-	
UPI000002	-	-	-	-	
UPI000012	deleterious	probably_c	hmmpanth	-	
UPI000004	-	-	Gene3D:3.:	-	
UPI000004	-	-	-	-	
UPI000004	deleterious	benign(0.3:	Gene3D:1.:	-	
UPI000004	-	-	Gene3D:3.4	2	
UPI000012	-	-	-	-	
UPI000003	tolerated(C	benign(0.2:	Gene3D:3.:	-	
UPI000003	-	-	-	-	
UPI000003	-	-	-	-	
UPI000003	deleterious	probably_c	Gene3D:2c	-	
UPI000003	-	-	-	-	
UPI000003	-	-	hmmpanth	-	
UPI000006	-	-	hmmpanth	-	
UPI0000D6	-	-	Coiled-coils	-	
UPI0000D6	-	-	-	-	

UPI000047	deleterious	possibly_da	hmmpanth	-	
UPI000012	-	-	Gene3D:4.1	-	
UPI00005B	tolerated(0	benign(0.15	Coiled-coils	-	
UPI00005B	-	-	-	-	
UPI000012	-	-	-	-	
UPI000012	-	-	hmmpanth	-	
UPI000012	deleterious	possibly_da	hmmpanth	-	
UPI000012	-	-	-	-	
UPI000012	deleterious	probably_c	hmmpanth	-	
UPI000012	tolerated(0	possibly_da	hmmpanth	-	
UPI000193	tolerated(0	probably_c	hmmpanth	-	
UPI000193	-	-	-	1	
UPI000012	-	-	-	-	
UPI000012	-	-	-		1
UPI000012	tolerated_l	probably_c	hmmpanth	-	
UPI000012	deleterious	probably_c	hmmpanth	-	
UPI000019	tolerated(0	benign(0.01	hmmpanth	-	
UPI000019	-	-	-	-	
UPI000019	tolerated(0	benign(0.05	hmmpanth	-	
UPI000019	deleterious	possibly_da	-	-	
UPI000016	-	-	-	-	
UPI0000DE	deleterious	benign(0.34	Gene3D:1.2	-	
UPI0000DE	deleterious	probably_c	Coiled-coils	-	
UPI000003	tolerated(0	benign(0.01	Gene3D:1.1	-	
UPI000013	-	-	-	-	
UPI000013	tolerated(0	benign(0.30	Gene3D:2g	-	
UPI000013	-	-	PROSITE_p	-	
UPI000013	tolerated(0	benign(0.03	hmmpanth	-	
UPI000006	tolerated_l	benign(0)	hmmpanth	-	
UPI000012	deleterious	possibly_da	PROSITE_p	-	
UPI000013	deleterious	possibly_da	PROSITE_p	-	
UPI000006	tolerated(0	benign(0.31	hmmpanth	-	
UPI00001B	-	-	Low_comp	-	
UPI000013	-	-	-	-	
UPI0000D6	-	-	-	-	
UPI000013	-	-	-	-	
UPI000004	tolerated(0	benign(0.41	PROSITE_p	-	
UPI000013	deleterious	probably_c	Pfam_dom	-	
UPI000013	deleterious	probably_c	Pfam_dom	-	
UPI000045	-	-	-	-	
UPI000045	tolerated(0	probably_c	hmmpanth	-	
UPI000045	tolerated(1	benign(0.00	Coiled-coils	-	
UPI000045	-	-	Coiled-coils	-	
UPI000045	-	-	Coiled-coils	-	
UPI000045	deleterious	probably_c	Coiled-coils	-	
UPI000045	deleterious	probably_c	Coiled-coils	-	
UPI000045	deleterious	probably_c	Coiled-coils	-	

UPI000045	deleterious	possibly_da	hmmpanth	-
UPI000045	-	-	hmmpanth	-
UPI000045	deleterious	probably_c	hmmpanth	-
UPI000045	tolerated(0	probably_c	Coiled-coils	-
UPI000045	-	-	-	-
UPI000045	-	-	hmmpanth	-
UPI000045	deleterious	benign(0.2	Coiled-coils	-
UPI000045	-	-	-	-
UPI000045	-	-	-	-
UPI000045	-	-	-	-
UPI000045	tolerated(0	benign(0.3	Pfam_dom	-
UPI000045	deleterious	probably_c	Pfam_dom	-
UPI000045	deleterious	probably_c	Pfam_dom	-
UPI000013	-	-	Gene3D:2.(	-
UPI000013	tolerated(0	benign(0)	Gene3D:2.(	-
UPI000013	-	-	-	-
UPI000013	tolerated(0	benign(0.0	Gene3D:2.(	-
UPI000013	-	-	-	-
UPI000020	tolerated_l	possibly_da	hmmpanth	-
UPI000013	-	-	-	-
UPI000013	-	-	-	-
UPI000004	tolerated(0	benign(0.2	Gene3D:3.	-
UPI000007	-	-	-	-
UPI000013	-	-	Gene3D:2.	-
UPI000013	-	-	Coiled-coils	-
UPI000013	tolerated(0	benign(0)	hmmpanth	-
UPI000013	deleterious	probably_c	Gene3D:2.	-
UPI000013	-	-	hmmpanth	-
UPI000013	tolerated(0	benign(0)	-	-
UPI000013	-	-	-	-
UPI000013	tolerated(1	benign(0)	-	-
UPI000004	-	-	hmmpanth	-
UPI000004	tolerated(0	benign(0.1	Gene3D:1.	-
UPI000013	-	-	hmmpanth	-
UPI000013	-	-	Superfamil	-
UPI000013	-	-	-	-
UPI000007	-	-	-	-
UPI000000	-	-	-	-
UPI000045	-	-	PROSITE_p	-
UPI000045	deleterious	possibly_da	PROSITE_p	-
UPI000045	-	-	Pfam_dom	-
UPI000045	-	-	Gene3D:1.	-
UPI000045	-	-	-	-
UPI00001A	tolerated(0	probably_c	Superfamil	-
UPI00001A	-	-	-	-
UPI000000	-	-	-	-
UPI000000	-	-	-	-

UPI000013	-	-	Gene3D:3.!	4
UPI000013	-	-	-	-
UPI000013	-	-	Superfamil	-
UPI000013	tolerated((	benign(0.0:	Superfamil	-
UPI000024	-	-	-	-
UPI000006	deleterious	possibly_da	hmmpanth	-
UPI000004	-	-	Superfamil	-
UPI000004	-	-	Superfamil	-
UPI000004	-	-	-	-
UPI000012	deleterious	probably_c	Superfamil	-
UPI000013	-	-	-	-
UPI000000	-	-	-	-
UPI000013	-	-	hmmpanth	5
UPI000013	deleterious	probably_c	hmmpanth	-
UPI000007	-	-	-	-
UPI000007	-	-	-	5
UPI0001D3	tolerated((	benign(0)	hmmpanth	-
UPI0001D3	-	-	-	-
UPI0001D3	tolerated((	benign(0.0:	hmmpanth	-
UPI0001D3	-	-	hmmpanth	-
UPI0001D3	-	-	-	-
UPI0001D3	-	-	hmmpanth	2
UPI000013	tolerated((	probably_c	hmmpanth	-
UPI000020	-	-	-	-
UPI0001D5	deleterious	probably_c	Pfam_dom	-
UPI0001D5	deleterious	possibly_da	hmmpanth	-
UPI0001D5	deleterious	probably_c	hmmpanth	-
UPI000000	-	-	-	-
UPI00017E	deleterious	probably_c	PROSITE_p	-
UPI00017E	tolerated_l	benign(0)	hmmpanth	-
UPI000013	-	-	Gene3D:1.:	-
UPI000013	-	-	-	-
UPI000013	tolerated((	benign(0.0:	hmmpanth	-
UPI000005	deleterious	probably_c	Superfamil	-
UPI000005	-	-	Gene3D:2.(	-
UPI000006	-	-	Superfamil	-
UPI000000	-	-	-	3
UPI000000	-	-	-	-
UPI000013	-	-	hmmpanth	-
UPI000003	tolerated((	benign(0.0:	hmmpanth	-
UPI000000	-	-	-	-
UPI000000	-	-	hmmpanth	-
UPI000003	tolerated((	benign(0.0	hmmpanth	-
UPI000007	-	-	Pfam_dom	-
UPI000012	deleterious	probably_c	PROSITE_p	-
UPI000204	-	-	Gene3D:1.:	-
UPI000204	-	-	Gene3D:1.:	-

UPI000204	-	probably_c	hmmpanth	-
UPI000204	-	-	hmmpanth	-
UPI000204	-	possibly_da	Gene3D:1.2	-
UPI000204	-	probably_c	hmmpanth	-
UPI000204	-	-	Coiled-coils	-
UPI000204	-	benign(0.1	hmmpanth	-
UPI000204	-	-	hmmpanth	-
UPI000204	-	benign(0.1	Gene3D:1.2	-
UPI000204	-	benign(0)	hmmpanth	-
UPI000204	-	benign(0.0	Gene3D:1.2	-
UPI000204	-	-	-	-
UPI000013	tolerated(0	benign(0)	Low_comp	-
UPI000013	tolerated_l	benign(0.14	PIRSF_dom	-
UPI000007	tolerated(0	benign(0.09	Low_comp	-
UPI000013	tolerated(0	benign(0.0	Gene3D:1h	-
UPI000013	-	-	-	-
UPI000013	-	-	Gene3D:1.1	-
UPI000002	deleterious	possibly_da	Gene3D:4.1	-
UPI000002	deleterious	probably_c	PROSITE_p	-
UPI000013	deleterious	benign(0.0	hmmpanth	-
UPI000013	-	-	hmmpanth	-
UPI000013	deleterious	possibly_da	hmmpanth	-
UPI00001D	tolerated(0	benign(0.0	hmmpanth	-
UPI00001D	-	-	hmmpanth	3
UPI000011	tolerated(0	benign(0.0	hmmpanth	-
UPI00001F	deleterious	probably_c	Superfamily	-
UPI000002	-	-	-	-
UPI000046	deleterious	benign(0.2	hmmpanth	-
UPI000046	-	-	Coiled-coils	-
UPI000046	-	-	-	-
UPI000013	-	-	-	-
UPI000013	-	-	-	-
UPI000013	-	-	-	-
UPI000013	-	-	-	-
UPI000013	deleterious	possibly_da	Coiled-coils	-
UPI000045	-	-	-	-
UPI000045	tolerated(0	benign(0.0	hmmpanth	-
UPI000045	-	-	hmmpanth	-
UPI000045	deleterious	probably_c	hmmpanth	-
UPI000045	-	-	-	-
UPI000013	-	-	-	-
UPI000013	-	-	-	-
UPI000013	deleterious	probably_c	Prints_dom	-
UPI000013	-	-	Superfamily	-
UPI000012	deleterious	possibly_da	hmmpanth	-
UPI000012	tolerated(0	benign(0.0	Coiled-coils	-
UPI000012	-	-	hmmpanth	-

UPI000012	deleterious	benign(0.2(	hmmpanth	-
UPI000012	-	-	-	-
UPI000012	-	-	hmmpanth	-
UPI00001A	-	-	-	-
UPI000007	-	-	PROSITE_p	-
UPI000007	-	-	-	-
UPI000007	-	-	-	-
UPI000007	-	-	-	-
UPI000007	-	-	-	-
UPI000013	-	-	Low_comp	3
UPI000013	-	-	-	-
UPI000013	-	-	-	-
UPI000013	deleterious	benign(0.2(	-	-
UPI00001A	-	-	Coiled-coils	-
UPI00001A	-	-	hmmpanth	-

EUR_AF	SAS_AF	AA_AF	EA_AF	gnomAD_A	gnomAD_A	gnomAD_A	gnomAD_A	gnomAD_E

gnomAD_F	gnomAD_N	gnomAD_C	gnomAD_S	empty	CLIN_SIG	SOMATIC	PHENO	PUBMED
				0	-	-	-	-
				0	-	-	-	-
				0	-	-	-	-
				0	-	-	-	-
				0	-	-	-	-
				0	-	-	-	-
				0	-	-	-	-
				0	-	-	-	-
				0	-	-	-	-
				0	-	-	-	-
				0	-	-	-	-
				0	-	-	-	-
				0	-	-	-	-
				0	-	-	-	-
				0	-	-	-	-
				0	-	-	-	-
				0	-	-	-	-
				0	-	-	-	-
				0	-	-	-	-
				0	-	-	-	-
				0	-	-	-	-
				0	-	-	-	-
				0	-	-	-	-
				0	-	-	-	-
				0	-	-	-	-
				0	-	-	-	-
				0	-	-	-	-
				0	-	-	-	-
				0	-	-	-	-
				0	-	-	-	-
				0	-	-	-	-
				0	-	-	-	-
				0	-	-	-	-
				0	-	-	-	-
				0	-	-	-	-
				0	-	-	-	-
				0	-	-	-	-
				0	-	-	-	-
				0	-	-	-	-
				0	-	-	-	-
				0	-	-	-	-
				0	-	-	-	-
				0	-	-	-	-
				0	-	-	-	-
				0	-	-	-	-

	0	-	-	-	-
	0	-	-	-	-
	0	-	-	-	-
	0	-	-	-	-
	0	-	-	-	-
	0	-	-	-	-
	0	-	-	-	-
	0	-	-	-	-
	0	-	-	-	-
	0	-	-	-	-
	0	-	-	-	-
	0	-	-	-	-
	0	-	-	-	-
	0	-	-	-	-
	0	-	-	-	-
	0	-	-	-	-
	0	-	-	-	-
	0	-	-	-	-
	0	-	-	-	-
	0	-	-	-	-
	0	-	-	-	-
	0	-	-	-	-
	0	-	-	-	-
	0	-	-	-	-
	0	-	-	-	-
	0	-	-	-	-
	0	-	-	-	-
	0	-	-	-	-
	0	-	-	-	-
	0	-	-	-	-
	0	-	-	-	-
	0	-	-	-	-
	0	-	-	-	-
	0	-	-	-	-
	0	-	-	-	-
	0	-	-	-	-
	0	-	-	-	-
	0	-	-	-	-
	0	-	-	-	-
	0	-	-	-	-
	0	-	-	-	-
	0	-	-	-	-
	0	-	-	-	-
	0	-	-	-	-
	0	-	-	-	-
	0	-	-	-	-
	0	-	-	-	-

0	-	-	-	-
0	-	-	-	-
0	-	-	-	-
0	-	-	-	-
0	-	-	-	-
0	-	-	-	-
0	-	-	-	-
0	-	-	-	-
0	-	-	-	-
0	-	-	-	-
0	-	-	-	-
0	-	-	-	-
0	-	-	-	-
0	-	-	-	-
0	-	-	-	-
0	-	-	-	-
0	-	-	-	-
0	-	-	-	-
0	-	-	-	-
0	-	-	-	-
0	-	-	-	-
0	-	-	-	-
0	-	-	-	-
0	-	-	-	-
0	-	-	-	-
0	-	-	-	-
0	-	-	-	-
0	-	-	-	-
0	-	-	-	-
0	-	-	-	-
0	-	-	-	-
0	-	-	-	-
0	-	-	-	-
0	-	-	-	-
0	-	-	-	-
0	-	-	-	-
0	-	-	-	-
0	-	-	-	-
0	-	-	-	-
0	-	-	-	-
0	-	-	-	-
0	-	-	-	-
0	-	-	-	-
0	-	-	-	-
0	-	-	-	-
0	-	-	-	-
0	-	-	-	-

0	-	-	-	-
0	-	-	-	-
0	-	-	-	-
0	-	-	-	-
0	-	-	-	-
0	-	-	-	-
0	-	-	-	-
0	-	-	-	-
0	-	-	-	-
0	-	-	-	-
0	-	-	-	-
0	-	-	-	-
0	-	-	-	-
0	-	-	-	-
0	-	-	-	-
0	-	-	-	-
0	-	-	-	-
0	-	-	-	-
0	-	-	-	-
0	-	-	-	-
0	-	-	-	-
0	-	-	-	-
0	-	-	-	-
0	-	-	-	-
0	-	-	-	-
0	-	-	-	-
0	-	-	-	-
0	-	-	-	-
0	-	-	-	-
0	-	-	-	-
0	-	-	-	-
0	-	-	-	-
0	-	-	-	-
0	-	-	-	-
0	-	-	-	-
0	-	-	-	-
0	-	-	-	-
0	-	-	-	-
0	-	-	-	-
0	-	-	-	-
0	-	-	-	-
0	-	-	-	-
0	-	-	-	-
0	-	-	-	-
0	-	-	-	-
0	-	-	-	-
0	-	-	-	-

0	-	-	-	-
0	-	-	-	-
0	-	-	-	-
0	-	-	-	-
0	-	-	-	-
0	-	-	-	-
0	-	-	-	-
0	-	-	-	-
0	-	-	-	-
0	-	-	-	-
0	-	-	-	-
0	-	-	-	-
0	-	-	-	-
0	-	-	-	-
0	-	-	-	-
0	-	-	-	-
0	-	-	-	-
0	-	-	-	-
0	-	-	-	-
0	-	-	-	-
0	-	-	-	-
0	-	-	-	-
0	-	-	-	-
0	-	-	-	-
0	-	-	-	-
0	-	-	-	-
0	-	-	-	-
0	-	-	-	-
0	-	-	-	-
0	-	-	-	-
0	-	-	-	-
0	-	-	-	-
0	-	-	-	-
0	-	-	-	-
0	-	-	-	-
0	-	-	-	-
0	-	-	-	-
0	-	-	-	-
0	-	-	-	-
0	-	-	-	-
0	-	-	-	-
0	-	-	-	-
0	-	-	-	-
0	-	-	-	-
0	-	-	-	-
0	-	-	-	-
0	-	-	-	-

0	-	-	-	-
0	-	-	-	-
0	-	-	-	-
0	-	-	-	-
0	-	-	-	-
0	-	-	-	-
0	-	-	-	-
0	-	-	-	-
0	-	-	-	-
0	-	-	-	-
0	-	-	-	-
0	-	-	-	-
0	-	-	-	-
0	-	-	-	-
0	-	-	-	-
0	-	-	-	-
0	-	-	-	-
0	-	-	-	-
0	-	-	-	-
0	-	-	-	-
0	-	-	-	-
0	-	-	-	-
0	-	-	-	-
0	-	-	-	-
0	-	-	-	-
0	-	-	-	-
0	-	-	-	-
0	-	-	-	-
0	-	-	-	-
0	-	-	-	-
0	-	-	-	-
0	-	-	-	-
0	-	-	-	-
0	-	-	-	-
0	-	-	-	-
0	-	-	-	-
0	-	-	-	-
0	-	-	-	-
0	-	-	-	-
0	-	-	-	-
0	-	-	-	-
0	-	-	-	-
0	-	-	-	-
0	-	-	-	-
0	-	-	-	-
0	-	-	-	-
0	-	-	-	-

0	-	-	-	-
0	-	-	-	-
0	-	-	-	-
0	-	-	-	-
0	-	-	-	-
0	-	-	-	-
0	-	-	-	-
0	-	-	-	-
0	-	-	-	-
0	-	-	-	-
0	-	-	-	-
0	-	-	-	-
0	-	-	-	-
0	-	-	-	-
0	-	-	-	-
0	-	-	-	-
0	-	-	-	-
0	-	-	-	-
0	-	-	-	-
0	-	-	-	-
0	-	-	-	-
0	-	-	-	-
0	-	-	-	-
0	-	-	-	-
0	-	-	-	-
0	-	-	-	-
0	-	-	-	-
0	-	-	-	-
0	-	-	-	-
0	-	-	-	-
0	-	-	-	-
0	-	-	-	-
0	-	-	-	-
0	-	-	-	-
0	-	-	-	-
0	-	-	-	-
0	-	-	-	-
0	-	-	-	-
0	-	-	-	-
0	-	-	-	-
0	-	-	-	-
0	-	-	-	-
0	-	-	-	-
0	-	-	-	-
0	-	-	-	-
0	-	-	-	-
0	-	-	-	-

0	-	-	-	-
0	-	-	-	-
0	-	-	-	-
0	-	-	-	-
0	-	-	-	-
0	-	-	-	-
0	-	-	-	-
0	-	-	-	-
0	-	-	-	-
0	-	-	-	-
0	-	-	-	-
0	-	-	-	-
0	-	-	-	-
0	-	-	-	-
0	-	-	-	-
0	-	-	-	-
0	-	-	-	-
0	-	-	-	-
0	-	-	-	-
0	-	-	-	-
0	-	-	-	-
0	-	-	-	-
0	-	-	-	-
0	-	-	-	-
0	-	-	-	-
0	-	-	-	-
0	-	-	-	-
0	-	-	-	-
0	-	-	-	-
0	-	-	-	-
0	-	-	-	-
0	-	-	-	-
0	-	-	-	-
0	-	-	-	-
0	-	-	-	-
0	-	-	-	-
0	-	-	-	-
0	-	-	-	-
0	-	-	-	-
0	-	-	-	-
0	-	-	-	-
0	-	-	-	-
0	-	-	-	-
0	-	-	-	-
0	-	-	-	-
0	-	-	-	-
0	-	-	-	-

0	-	-	-	-
0	-	-	-	-
0	-	-	-	-
0	-	-	-	-
0	-	-	-	-
0	-	-	-	-
0	-	-	-	-
0	-	-	-	-
0	-	-	-	-
0	-	-	-	-
0	-	-	-	-
0	-	-	-	-
0	-	-	-	-
0	-	-	-	-
0	-	-	-	-
0	-	-	-	-
0	-	-	-	-
0	-	-	-	-
0	-	-	-	-
0	-	-	-	-
0	-	-	-	-
0	-	-	-	-
0	-	-	-	-
0	-	-	-	-
0	-	-	-	-
0	-	-	-	-
0	-	-	-	-
0	-	-	-	-
0	-	-	-	-
0	-	-	-	-
0	-	-	-	-
0	-	-	-	-
0	-	-	-	-
0	-	-	-	-
0	-	-	-	-
0	-	-	-	-
0	-	-	-	-
0	-	-	-	-
0	-	-	-	-
0	-	-	-	-
0	-	-	-	-
0	-	-	-	-
0	-	-	-	-
0	-	-	-	-
0	-	-	-	-
0	-	-	-	-
0	-	-	-	-

0	-	-	-	-
0	-	-	-	-
0	-	-	-	-
0	-	-	-	-
0	-	-	-	-
0	-	-	-	-
0	-	-	-	-
0	-	-	-	-
0	-	-	-	-
0	-	-	-	-
0	-	-	-	-
0	-	-	-	-
0	-	-	-	-
0	-	-	-	-
0	-	-	-	-
0	-	-	-	-
0	-	-	-	-
0	-	-	-	-
0	-	-	-	-
0	-	-	-	-
0	-	-	-	-
0	-	-	-	-
0	-	-	-	-
0	-	-	-	-
0	-	-	-	-
0	-	-	-	-
0	-	-	-	-
0	-	-	-	-
0	-	-	-	-
0	-	-	-	-
0	-	-	-	-
0	-	-	-	-
0	-	-	-	-
0	-	-	-	-
0	-	-	-	-
0	-	-	-	-
0	-	-	-	-
0	-	-	-	-
0	-	-	-	-
0	-	-	-	-
0	-	-	-	-
0	-	-	-	-
0	-	-	-	-
0	-	-	-	-
0	-	-	-	-
0	-	-	-	-
0	-	-	-	-

0	-	-	-	-
0	-	-	-	-
0	-	-	-	-
0	-	-	-	-
0	-	-	-	-
0	-	-	-	-
0	-	-	-	-
0	-	-	-	-
0	-	-	-	-
0	-	-	-	-
0	-	-	-	-
0	-	-	-	-
0	-	-	-	-
0	-	-	-	-
0	-	-	-	-

MOTIF_NA	MOTIF_PO	HIGH_INF_	MOTIF_SC	MaxEntSca	MaxEntSca	MaxEntSca	ada_score	rf_score
-	-	-	-	-	-	-	-	-
-	-	-	-	-	-	-	-	-
-	-	-	-	-	-	-	-	-
-	-	-	-	-	-	-	-	-
-	-	-	-	-	-	-	-	-
-	-	-	-	-	-	-	-	-
-	-	-	-	-	-	-	-	-
-	-	-	-	-	-	-	-	-
-	-	-	-	-	-	-	-	-
-	-	-	-	-	-	-	-	-
-	-	-	-	-	-	-	-	-
-	-	-	-	-	-	-	-	-
-	-	-	-	-	-	-	-	-
-	-	-	-	-	-	-	-	-
-	-	-	-	-	-	-	-	-
-	-	-	-	-	-	-	-	-
-	-	-	-	-	-	-	-	-
-	-	-	-	-	-	-	-	-
-	-	-	-	-	-	-	-	-
-	-	-	-	-	-	-	-	-
-	-	-	-	-	-	-	-	-
-	-	-	-	-	-	-	-	-
-	-	-	-	-	-	-	-	-
-	-	-	-	-	-	-	-	-
-	-	-	-	-	-	-	-	-
-	-	-	-	-	-	-	-	-
-	-	-	-	9.397	1.084	10.481	9.6048870	0.018
-	-	-	-	-	-	-	-	-
-	-	-	-	-	-	-	-	-
-	-	-	-	-	-	-	-	-
-	-	-	-	-	-	-	-	-
-	-	-	-	-	-	-	-	-
-	-	-	-	-	-	-	-	-
-	-	-	-	-	-	-	-	-
-	-	-	-	-	-	-	-	-
-	-	-	-	-	-	-	-	-
-	-	-	-	0.274	8.182	8.456	0.9999882	0.93
-	-	-	-	-	-	-	-	-
-	-	-	-	-	-	-	-	-
-	-	-	-	-	-	-	-	-
-	-	-	-	-	-	-	-	-
-	-	-	-	-	-	-	-	-
-	-	-	-	-	-	-	-	-
-	-	-	-	8.837	0.179	9.016	0.1173378	0.272
-	-	-	-	-	-	-	-	-

-	-	-	-	-	-	-	-	-
-	-	-	-	-	-	-	-	-
-	-	-	-	-	-	-	-	-
-	-	-	-	-	-	-	-	-
-	-	-	-	-	-	-	-	-
-	-	-	-	-	-	-	-	-
-	-	-	-	-	-	-	-	-
-	-	-	-	-	-	-	-	-
-	-	-	-	-	-	-	-	-
-	-	-	-	-	-	-	-	-
-	-	-	-	-	-	-	-	-
-	-	-	-	-	-	-	-	-
-	-	-	-	-	-	-	-	-
-	-	-	-	-	-	-	-	-
-	-	-	-	-	-	-	-	-
-	-	-	-	-	-	-	-	-
-	-	-	-	-	-	-	-	-
-	-	-	-	-	-	-	-	-
-	-	-	-	-	-	-	-	-
-	-	-	-	-0.795	3.901	3.106	0.9986473	0.986
-	-	-	-	-	-	-	-	-
-	-	-	-	8.465	0.068	8.533	-	-
-	-	-	-	-	-	-	-	-
-	-	-	-	-	-	-	-	-
-	-	-	-	-	-	-	-	-
-	-	-	-	-	-	-	-	-
-	-	-	-	-	-	-	-	-
-	-	-	-	-	-	-	-	-
-	-	-	-	-	-	-	-	-
-	-	-	-	-	-	-	-	-
-	-	-	-	1.169	5.290	6.459	0.9996948	0.972
-	-	-	-	-	-	-	-	-
-	-	-	-	11.579	-2.455	9.124	2.6623750	0.142
-	-	-	-	-	-	-	-	-
-	-	-	-	-	-	-	-	-
-	-	-	-	-	-	-	-	-
-	-	-	-	-	-	-	-	-
-	-	-	-	-1.289	8.042	6.754	0.9999867	0.934
-	-	-	-	12.056	-0.650	11.406	2.8229072	0.0
-	-	-	-	-	-	-	-	-
-	-	-	-	-	-	-	-	-
-	-	-	-	-	-	-	-	-
-	-	-	-	-	-	-	-	-
-	-	-	-	-	-	-	-	-
-	-	-	-	-	-	-	-	-
-	-	-	-	-	-	-	-	-

-	-	-	-	-	-	-	-	-
-	-	-	-	3.374	5.886	9.260	0.9995448	0.992
-	-	-	-	-	-	-	-	-
-	-	-	-	5.280	3.878	9.158	0.9978555	0.896
-	-	-	-	-	-	-	-	-
-	-	-	-	-	-	-	-	-
-	-	-	-	-	-	-	-	-
-	-	-	-	-	-	-	-	-
-	-	-	-	-	-	-	-	-
-	-	-	-	8.922	-0.546	8.376	2.6144024	0.026
-	-	-	-	-	-	-	-	-
-	-	-	-	6.457	0.600	7.057	0.0939294	0.462
-	-	-	-	-	-	-	-	-
-	-	-	-	-	-	-	-	-
-	-	-	-	-	-	-	-	-
-	-	-	-	-	-	-	-	-
-	-	-	-	-	-	-	-	-
-	-	-	-	-	-	-	-	-
-	-	-	-	-	-	-	-	-
-	-	-	-	-	-	-	-	-
-	-	-	-	-	-	-	-	-
-	-	-	-	-	-	-	-	-
-	-	-	-	-	-	-	-	-
-	-	-	-	6.596	3.916	10.512	0.9997878	0.942
-	-	-	-	-	-	-	-	-
-	-	-	-	-	-	-	-	-
-	-	-	-	-	-	-	-	-
-	-	-	-	-	-	-	-	-
-	-	-	-	-	-	-	-	-
-	-	-	-	4.647	0.106	4.754	4.8606412	0.04
-	-	-	-	8.571	-0.045	8.526	-	-
-	-	-	-	-	-	-	-	-
-	-	-	-	-1.262	7.955	6.693	0.9999882	0.93
-	-	-	-	-	-	-	-	-
-	-	-	-	9.158	1.355	10.512	0.6908177	0.43
-	-	-	-	-	-	-	-	-
-	-	-	-	-	-	-	-	-
-	-	-	-	-	-	-	-	-
-	-	-	-	-	-	-	-	-
-	-	-	-	-	-	-	-	-
-	-	-	-	-	-	-	-	-
-	-	-	-	-	-	-	-	-
-	-	-	-	-	-	-	-	-
-	-	-	-	-	-	-	-	-
-	-	-	-	8.381	1.148	9.529	1.6593870	0.0
-	-	-	-	-	-	-	-	-
-	-	-	-	-	-	-	-	-

-	-	-	-	-	-	-	-	-
-	-	-	-	-	-	-	-	-
-	-	-	-	-	-	-	-	-
-	-	-	-	-	-	-	-	-
-	-	-	-	-	-	-	-	-
-	-	-	-	-	-	-	-	-
-	-	-	-	-	-	-	-	-
-	-	-	-	-	-	-	-	-
-	-	-	-	7.299	-1.545	5.754	-	-
-	-	-	-	-	-	-	-	-
-	-	-	-	-	-	-	-	-
-	-	-	-	-	-	-	-	-
-	-	-	-	-	-	-	-	-
-	-	-	-	-	-	-	-	-
-	-	-	-	-	-	-	-	-
-	-	-	-	-	-	-	-	-
-	-	-	-	-	-	-	-	-
-	-	-	-	-	-	-	-	-
-	-	-	-	-	-	-	-	-
-	-	-	-	-	-	-	-	-
-	-	-	-	-	-	-	-	-
-	-	-	-	-	-	-	-	-
-	-	-	-	-	-	-	-	-
-	-	-	-	-	-	-	-	-
-	-	-	-	-	-	-	-	-
-	-	-	-	-	-	-	-	-
-	-	-	-	-	-	-	-	-
-	-	-	-	-	-	-	-	-
-	-	-	-	-	-	-	-	-
-	-	-	-	-	-	-	-	-
-	-	-	-	-	-	-	-	-
-	-	-	-	-	-	-	-	-
-	-	-	-	6.973	0.542	7.515	-	-
-	-	-	-	-	-	-	-	-
-	-	-	-	-	-	-	-	-
-	-	-	-	-	-	-	-	-
-	-	-	-	-	-	-	-	-
-	-	-	-	-	-	-	-	-
-	-	-	-	-	-	-	7.2941717	0.008
-	-	-	-	-	-	-	-	-
-	-	-	-	-	-	-	-	-
-	-	-	-	-	-	-	-	-
-	-	-	-	-	-	-	-	-
-	-	-	-	-	-	-	-	-
-	-	-	-	-	-	-	-	-
-	-	-	-	-	-	-	-	-
-	-	-	-	-	-	-	-	-

-	-	-	-	-	-	-	-	-
-	-	-	-	9.256	-0.984	8.272	1.7682378	0.0
-	-	-	-	-	-	-	-	-
-	-	-	-	-	-	-	-	-
-	-	-	-	1.732	8.042	9.774	0.9999882	0.932
-	-	-	-	-	-	-	-	-
-	-	-	-	-	-	-	-	-
-	-	-	-	-	-	-	-	-
-	-	-	-	-	-	-	-	-
-	-	-	-	-	-	-	-	-
-	-	-	-	-	-	-	-	-
-	-	-	-	-	-	-	-	-
-	-	-	-	-6.156	12.522	6.366	0.1789392	0.454
-	-	-	-	-	-	-	-	-
-	-	-	-	-	-	-	-	-
-	-	-	-	-	-	-	-	-
-	-	-	-	-	-	-	-	-
-	-	-	-	-	-	-	-	-
-	-	-	-	-	-	-	-	-
-	-	-	-	-	-	-	-	-
-	-	-	-	-	-	-	-	-
-	-	-	-	-	-	-	-	-
-	-	-	-	-	-	-	-	-
-	-	-	-	5.127	5.066	10.193	0.9999000	0.994
-	-	-	-	-	-	-	-	-
-	-	-	-	-	-	-	-	-
-	-	-	-	-	-	-	-	-
-	-	-	-	-	-	-	-	-
-	-	-	-	-	-	-	-	-
-	-	-	-	7.616	1.018	8.634	4.7563193	0.074
-	-	-	-	4.676	1.359	6.035	0.0084925	0.358
-	-	-	-	-	-	-	-	-
-	-	-	-	-	-	-	-	-
-	-	-	-	-	-	-	-	-
-	-	-	-	-	-	-	-	-
-	-	-	-	-	-	-	-	-
-	-	-	-	-	-	-	-	-
-	-	-	-	-	-	-	-	-
-	-	-	-	10.167	-0.772	9.395	-	-
-	-	-	-	8.591	-0.027	8.564	0.0070353	0.096
-	-	-	-	-	-	-	-	-
-	-	-	-	8.668	0.781	9.449	0.0110941	0.138
-	-	-	-	-	-	-	-	-
-	-	-	-	-	-	-	-	-
-	-	-	-	-	-	-	-	-
-	-	-	-	9.684	1.102	10.786	0.2638011	0.514
-	-	-	-	-	-	-	-	-

-	-	-	-	0.948	8.598	9.547	0.9999892	0.934
-	-	-	-	-	-	-	-	-
-	-	-	-	-	-	-	-	-
-	-	-	-	-	-	-	-	-
-	-	-	-	-	-	-	-	-
-	-	-	-	-	-	-	-	-
-	-	-	-	-	-	-	-	-
-	-	-	-	-	-	-	-	-
-	-	-	-	-	-	-	-	-
-	-	-	-	-	-	-	-	-
-	-	-	-	10.749	-0.510	10.239	0.0112124	0.27
-	-	-	-	-	-	-	-	-
-	-	-	-	-	-	-	-	-
-	-	-	-	-	-	-	-	-
-	-	-	-	-	-	-	-	-
-	-	-	-	-	-	-	-	-
-	-	-	-	-	-	-	-	-
-	-	-	-	-	-	-	-	-
-	-	-	-	-	-	-	-	-
-	-	-	-	-	-	-	-	-
-	-	-	-	-	-	-	-	-
-	-	-	-	-	-	-	-	-
-	-	-	-	-	-	-	-	-
-	-	-	-	-	-	-	-	-
-	-	-	-	-	-	-	-	-
-	-	-	-	-	-	-	-	-
-	-	-	-	-	-	-	-	-
-	-	-	-	-	-	-	-	-
-	-	-	-	-	-	-	-	-
-	-	-	-	-	-	-	-	-
-	-	-	-	-	-	-	-	-
-	-	-	-	-	-	-	-	-
-	-	-	-	-	-	-	-	-
-	-	-	-	-	-	-	-	-
-	-	-	-	-	-	-	-	-
-	-	-	-	-	-	-	-	-
-	-	-	-	-	-	-	-	-
-	-	-	-	-	-	-	-	-
-	-	-	-	-	-	-	-	-
-	-	-	-	-	-	-	-	-
-	-	-	-	4.472	2.336	6.808	0.0402041	0.28
-	-	-	-	-0.871	-0.714	-1.584	-	-
-	-	-	-	-	-	-	-	-
-	-	-	-	-	-	-	-	-
-	-	-	-	-	-	-	-	-
-	-	-	-	-	-	-	-	-
-	-	-	-	-	-	-	-	-
-	-	-	-	-	-	-	-	-

-	-	-	-	-	-	-	-	-
-	-	-	-	-	-	-	-	-
-	-	-	-	-	-	-	-	-
-	-	-	-	6.465	0.029	6.493	6.2061654	0.002
-	-	-	-	-	-	-	-	-
-	-	-	-	-	-	-	-	-
-	-	-	-	-	-	-	-	-
-	-	-	-	-	-	-	-	-
-	-	-	-	-	-	-	-	-
-	-	-	-	-	-	-	-	-
-	-	-	-	-	-	-	-	-
-	-	-	-	-	-	-	-	-
-	-	-	-	-	-	-	-	-
-	-	-	-	-	-	-	-	-
-	-	-	-	-	-	-	-	-
-	-	-	-	-	-	-	-	-
-	-	-	-	-	-	-	-	-
-	-	-	-	-	-	-	-	-
-	-	-	-	-	-	-	-	-
-	-	-	-	-	-	-	-	-
-	-	-	-	-	-	-	-	-
-	-	-	-	-	-	-	-	-
-	-	-	-	6.530	1.942	8.471	-	-
-	-	-	-	-	-	-	-	-
-	-	-	-	-	-	-	-	-
-	-	-	-	-	-	-	-	-
-	-	-	-	-	-	-	-	-
-	-	-	-	-	-	-	-	-
-	-	-	-	-	-	-	-	-
-	-	-	-	-	-	-	-	-
-	-	-	-	-	-	-	-	-
-	-	-	-	-	-	-	-	-
-	-	-	-	-	-	-	-	-
-	-	-	-	-	-	-	-	-
-	-	-	-	-	-	-	-	-
-	-	-	-	-	-	-	-	-
-	-	-	-	-	-	-	-	-
-	-	-	-	10.648	-1.077	9.571	1.6794498	0.0
-	-	-	-	-	-	-	-	-
-	-	-	-	-	-	-	-	-
-	-	-	-	-	-	-	-	-
-	-	-	-	-	-	-	-	-
-	-	-	-	-	-	-	-	-
-	-	-	-	-	-	-	-	-
-	-	-	-	-	-	-	-	-
-	-	-	-	-	-	-	-	-
-	-	-	-	-	-	-	-	-
-	-	-	-	-	-	-	-	-
-	-	-	-	-	-	-	-	-

-	-	-	-	-	-	-	-	-
-	-	-	-	-	-	-	-	-
-	-	-	-	-	-	-	-	-
-	-	-	-	-	-	-	-	-
-	-	-	-	6.683	3.103	9.786	0.97249029	0.73
-	-	-	-	-	-	-	-	-
-	-	-	-	-	-	-	-	-
-	-	-	-	-	-	-	-	-
-	-	-	-	-	-	-	-	-
-	-	-	-	-	-	-	-	-
-	-	-	-	-	-	-	-	-
-	-	-	-	-	-	-	-	-
-	-	-	-	-	-	-	-	-
-	-	-	-	-	-	-	-	-
-	-	-	-	-	-	-	-	-
-	-	-	-	-	-	-	-	-
-	-	-	-	-	-	-	-	-
-	-	-	-	-	-	-	-	-
-	-	-	-	-	-	-	-	-
-	-	-	-	-	-	-	-	-
-	-	-	-	-	-	-	-	-
-	-	-	-	-	-	-	-	-
-	-	-	-	-	-	-	-	-
-	-	-	-	-	-	-	-	-
-	-	-	-	-	-	-	-	-
-	-	-	-	-	-	-	-	-
-	-	-	-	-	-	-	-	-
-	-	-	-	-	-	-	-	-
-	-	-	-	-	-	-	-	-
-	-	-	-	-	-	-	-	-
-	-	-	-	-	-	-	-	-
-	-	-	-	-	-	-	-	-
-	-	-	-	-	-	-	-	-
-	-	-	-	-	-	-	-	-
-	-	-	-	-	-	-	-	-
-	-	-	-	11.289	-1.317	9.972	-	-
-	-	-	-	9.486	1.929	11.415	4.14730358	0.118
-	-	-	-	-	-	-	-	-
-	-	-	-	-	-	-	-	-
-	-	-	-	-	-	-	-	-
-	-	-	-	-	-	-	-	-
-	-	-	-	-	-	-	-	-
-	-	-	-	-	-	-	-	-
-	-	-	-	-	-	-	-	-
-	-	-	-	-	-	-	-	-
-	-	-	-	1.498	4.217	5.715	0.99868670	0.886

-	-	-	-	-	-	-	-	-
-	-	-	-	-	-	-	-	-
-	-	-	-	-	-	-	-	-
-	-	-	-	-	-	-	-	-
-	-	-	-	-	-	-	-	-
-	-	-	-	-	-	-	-	-
-	-	-	-	-	-	-	-	-
-	-	-	-	-	-	-	-	-
-	-	-	-	-	-	-	-	-
-	-	-	-	-	-	-	-	-
-	-	-	-	-	-	-	4.3307777	0.0
-	-	-	-	-	-	-	-	-
-	-	-	-	-	-	-	-	-
-	-	-	-	7.630	0.005	7.635	0.5006906	0.456
-	-	-	-	8.897	0.629	9.526	-	-
-	-	-	-	-	-	-	-	-
-	-	-	-	-	-	-	-	-
-	-	-	-	6.958	0.656	7.614	0.0281327	0.342
-	-	-	-	-	-	-	-	-
-	-	-	-	-	-	-	-	-
-	-	-	-	-	-	-	-	-
-	-	-	-	-	-	-	-	-
-	-	-	-	-	-	-	-	-
-	-	-	-	-	-	-	-	-
-	-	-	-	-	-	-	-	-
-	-	-	-	-	-	-	-	-
-	-	-	-	-	-	-	-	-
-	-	-	-	-	-	-	-	-
-	-	-	-	-	-	-	-	-
-	-	-	-	-	-	-	-	-
-	-	-	-	-	-	-	-	-
-	-	-	-	-	-	-	-	-
-	-	-	-	-	-	-	-	-
-	-	-	-	-	-	-	-	-
-	-	-	-	-	-	-	-	-
-	-	-	-	-	-	-	-	-
-	-	-	-	-	-	-	-	-
-	-	-	-	-	-	-	-	-
-	-	-	-	-	-	-	-	-
-	-	-	-	-	-	-	-	-
-	-	-	-	-	-	-	-	-
-	-	-	-	-	-	-	-	-
-	-	-	-	-	-	-	-	-
-	-	-	-	-	-	-	-	-
-	-	-	-	-	-	-	-	-
-	-	-	-	-	-	-	-	-
-	-	-	-	-	-	-	-	-

-	-	-	-	-	-	-	-	-
-	-	-	-	-	-	-	-	-
-	-	-	-	-	-	-	-	-
-	-	-	-	-	-	-	-	-
-	-	-	-	-	-	-	-	-
-	-	-	-	-	-	-	-	-
-	-	-	-	-	-	-	-	-
-	-	-	-	-	-	-	-	-
-	-	-	-	-	-	-	-	-
-	-	-	-	-	-	-	-	-
-	-	-	-	9.349	1.225	10.575	-	-
-	-	-	-	-	-	-	-	-
-	-	-	-	-	-	-	-	-
-	-	-	-	-	-	-	-	-
-	-	-	-	-	-	-	-	-
-	-	-	-	-	-	-	-	-
-	-	-	-	-	-	-	-	-
-	-	-	-	-	-	-	-	-
-	-	-	-	-	-	-	-	-
-	-	-	-	-	-	-	-	-
-	-	-	-	-	-	-	-	-
-	-	-	-	-	-	-	-	-
-	-	-	-	-	-	-	-	-
-	-	-	-	-	-	-	-	-
-	-	-	-	-	-	-	-	-
-	-	-	-	-	-	-	-	-
-	-	-	-	-	-	-	-	-
-	-	-	-	2.639	0.402	3.041	-	-
-	-	-	-	-	-	-	-	-
-	-	-	-	-	-	-	-	-
-	-	-	-	-	-	-	-	-
-	-	-	-	6.621	3.044	9.664	0.8036078	0.634
-	-	-	-	-	-	-	-	-
-	-	-	-	-	-	-	-	-
-	-	-	-	-	-	-	-	-
-	-	-	-	-	-	-	-	-
-	-	-	-	-	-	-	-	-
-	-	-	-	-	-	-	-	-
-	-	-	-	-	-	-	-	-
-	-	-	-	-	-	-	-	-
-	-	-	-	-	-	-	-	-
-	-	-	-	4.362	0.399	4.761	5.2025051	0.026
-	-	-	-	-	-	-	-	-
-	-	-	-	-	-	-	-	-
-	-	-	-	-	-	-	-	-
-	-	-	-	-	-	-	-	-
-	-	-	-	-	-	-	-	-

-	-	-	-	-	-	-	-	-
-	-	-	-	-	-	-	-	-
-	-	-	-	-	-	-	-	-
-	-	-	-	-	-	-	-	-
-	-	-	-	-	-	-	-	-
-	-	-	-	-	-	-	-	-
-	-	-	-	-	-	-	-	-
-	-	-	-	-	-	-	-	-
-	-	-	-	-	-	-	-	-
-	-	-	-	-	-	-	-	-
-	-	-	-	8.967	-0.674	8.293	8.7161744	0.0
-	-	-	-	-	-	-	-	-
-	-	-	-	-	-	-	-	-
-	-	-	-	-	-	-	-	-
-	-	-	-	-	-	-	-	-

LoFtool	miRNA	BLOSUM62	CADD_PHR	CADD_RAW	Condel	GTEx_V6p_	GTEx_V6p_	Interpro_do
0.0781	-	-	39	7.406226	-	-	-	Activin_typ
0.85	-	-	3.862	0.075802	-	-	-	-
0.657	-	-	1.954	-0.048597	-	-	-	-
0.657	-	-	0.115	-0.462847	-	-	-	-
0.933	-	-2	25.8	3.677638	deleterious	-	-	ELK_domai
0.933	-	-	-	-	-	-	-	-
0.933	-	-1	24.7	3.394578	deleterious	-	-	-
0.933	-	-	9.341	0.495403	-	-	-	-
0.933	-	-	-	-	-	-	-	-
0.933	-	-2	22.8	2.605057	deleterious	-	-	-
0.933	-	-1	9.977	0.566458	neutral(0.2	-	-	-
0.324	-	-1	22.8	2.627499	neutral(0.0	-	-	Protein_Kir
0.341	-	-	-	-	-	-	-	-
0.202	-	-	-	-	-	-	-	-
0.00386	-	3	22.9	2.629020	neutral(0.0	-	-	Armadillo-l
0.00386	-	-	-	-	-	-	-	-
0.00386	-	-	-	-	-	-	-	-
0.00386	-	-	-	-	-	-	-	-
0.00386	-	-	-	-	-	-	-	-
0.0125	-	-	-	-	-	-	-	-
0.0215	-	-	-	-	-	-	-	-
0.0215	-	3	27.3	3.924020	deleterious	-	-	-
0.0215	-	-	35	6.027999	-	-	-	ARID_DNA-
0.0215	-	-	-	-	-	-	-	-
0.0215	-	-2	32	4.243295	deleterious	-	-	Armadillo-l
0.259	-	-1	19.88	2.064992	neutral(0.0	-	-	-
0.54	-	-	14.16	1.148492	-	-	-	-
0.782	-	-	-	-	-	-	-	-
0.782	-	-	7.001	0.283820	-	-	-	-
0.782	-	-	15.00	1.317277	-	-	-	-
0.718	-	-	-	-	-	-	-	-
0.718	-	-	0.402	-0.285761	-	-	-	-
0.718	-	-	-	-	-	-	-	-
0.718	-	1	19.01	1.979767	neutral(0.0	-	-	Armadillo-l
0.718	-	-	9.228	0.483378	-	-	-	-
0.718	-	-	1.225	-0.119947	-	-	-	-
0.00517	-	-	34	4.525380	-	-	-	-
0.674	-	-	-	-	-	-	-	-
0.635	-	-	10.85	0.669733	-	-	-	-
0.907	-	-	10.43	0.619176	-	-	-	-
0.364	-	1	22.8	2.583007	neutral(0.0	-	-	-
0.364	-	1	22.0	2.306643	neutral(0.2	-	-	-
0.289	-	-	16.72	1.688332	-	-	-	-
0.289	-	-	16.05	1.553495	-	-	-	-
0.0194	-	-	18.87	1.965485	-	-	-	-

0.155	-	-1	19.94	2.070122	neutral(0.0	-	-	-		
0.25	-	-	37	7.053725	-	-	-	-		
0.25	-	-	14.67	1.247520	-	-	-	-		
0.509	-	0	7.528	0.324729	neutral(0.0	-	-	-		
0.509	-	-3	0.945	-0.158338	neutral(0.0	-	-	-		
0.509	-	-	4.924	0.140265	-	-	-	-		
0.509	-	-1	17.75	1.838520	neutral(0.3	-	-	Zinc_finger		
0.509	-	2	25.5	3.612242	deleterious	-	-	Zinc_finger		
0.889	-	-	10.03	0.571227	-	-	-	-		
0.889	-	-	8.399	0.400995	-	-	-	-		
0.959	-	0	10.94	0.681812	neutral(0.2	-	-	Bloom_syn		
0.959	-	2	24.7	3.401847	neutral(0.4	-	-	HRDC_dom		
TA	0.0565	AGAMMAG	-	1.243	-0.11783	-	-	-		
0.64	-	-	12.78	0.925681	-	-	-	-		
0.64	-	-	1.466	-0.093081	-	-	-	-		
0.64	-	-	8.957	0.455416	-	-	-	-		
0.251	-	3	19.79	2.056444	neutral(0.2	-	-	-		
0.729	-	2	14.03	1.124374	neutral(0.0	-	-	-		
0.729	-	-	-	-	-	-	-	-		
TT	0.302	Noonan_sy	-	3.427	0.049922	-	-	-		
0.112	-	-	16.98	1.731263	-	-	-	-		
0.112	-	-	0.729	-0.196982	-	-	-	-		
0.329	-	-	13.80	1.084741	-	-	-	-		
-	-	-	9.404	0.502186	-	-	-	-		
0.499	-	-	6.275	0.230097	-	-	-	-		
0.499	-	2	15.67	1.468705	neutral(0.0	-	-	Cadherin_p		
0.23	-	1	23.4	2.901114	neutral(0.0	-	-	Cadherin,_		
0.134	-	-2	22.1	2.339211	neutral(0.3	-	-	-		
0.303	-	-	-	-	-	-	-	-		
-	-	-	21.0	2.162886	-	-	-	-		
0.0161	-	-	10.93	0.679899	-	-	-	-		
0.0161	-	-	23.0	2.699524	-	-	-	-		
CCCCAGgcc	0.00406	-	-	-	-	-	-	-		
0.219	-	-1	22.3	2.405989	neutral(0.3	-	-	CULT_dom		
0.219	-	-	9.094	0.469422	-	-	-	-		
0.0011	-	-	10.84	0.668680	-	-	-	-		
0.585	-	-	-	-	-	-	-	-		
0.585	-	-	-	-	-	-	-	-		
0.585	-	-	33	4.504386	-	-	-	-		
0.585	-	-	3.665	0.064129	-	-	-	-		
0.585	-	-	1.349	-0.105685	-	-	-	-		
0.34	-	1	23.6	2.975696	neutral(0.3	-	-	-		
0.34	-	-	-	-	-	-	-	-		
0.208	-	0	24.0	3.162167	deleterious	-	-	Armadillo-l		
0.923	-	1	6.551	0.249920	neutral(0.2	-	-	-		
-	-	0	15.68	1.469832	neutral(0.3	-	-	-		
0.313	-	-2	22.2	2.375680	neutral(0.0	-	-	Immunoglc		

0.313	-	-1	27.5	3.943797	deleterious	-	-	Immunoglo	
0.313	-	-3	34	4.734982	deleterious	-	-	-	
0.313	-	-	6.121	0.219197	-	-	-	-	
0.0426	-	-	22.0	2.313914	-	-	-	-	
0.0426	-	-	-	-	-	-	-	-	
0.0426	-	1	24.3	3.257354	deleterious	-	-	Protein_kir	
0.233	-	-	11.03	0.692801	-	-	-	-	
0.15	-	-	14.93	1.302455	-	-	-	S-adenosyl-	
0.15	-	-1	23.8	3.068783	neutral(0.4	-	-	-	
0.11	-	-	13.73	1.071748	-	-	-	-	
0.11	-	-	0.251	-0.353513	-	-	-	-	
0.11	-	-	11.05	0.694998	-	-	-	-	
0.11	-	-	13.64	1.057698	-	-	-	-	
0.554	-	-2	27.6	3.956325	deleterious	-	-	-	
0.554	-	-3	32	4.273851	deleterious	-	-	-	
0.554	-	-	10.37	0.612156	-	-	-	-	
T	0.554	NEUROPAT	-	22.3	2.384179	-	-	-	
0.554	-	-2	32	4.246285	deleterious	-	-	-	
0.554	-	-	-	-	-	-	-	-	
0.554	-	-	18.00	1.869520	-	-	-	-	
0.554	-	-	-	-	-	-	-	-	
0.000482	-	-	-	-	-	-	-	-	
0.000482	-	-	1.602	-0.079592	-	-	-	-	
0.00889	-	-2	34	4.772741	deleterious	-	-	-	
0.00889	-	1	17.89	1.856615	neutral(0.2	-	-	-	
0.00889	-	-	5.977	0.209217	-	-	-	-	
0.00889	-	-	7.223	0.300684	-	-	-	-	
0.00889	-	2	28.5	4.047229	deleterious	-	-	-	
0.00889	-	-1	20.6	2.122317	neutral(0.2	-	-	-	
0.291	-	-	13.78	1.080875	-	-	-	-	
0.291	-	-	6.802	0.268680	-	-	-	-	
0.291	-	-1	22.3	2.394410	neutral(0.0	-	-	Ephrin_rec	
0.223	-	-	34	4.705756	-	-	-	-	
0.223	-	-	10.77	0.660535	-	-	-	-	
0.223	-	-	22.7	2.536341	-	-	-	-	
0.19	-	-	12.51	0.887231	-	-	-	-	
0.149	-	-	5.346	0.167281	-	-	-	-	
0.149	-	-	10.12	0.582008	-	-	-	-	
0.149	-	-	22.1	2.335405	-	-	-	-	
0.31	-	-	0.728	-0.197352	-	-	-	-	
0.147	-	-	6.256	0.228688	-	-	-	-	
0.147	-	-	2.108	-0.036405	-	-	-	-	
0.147	-	-	11.08	0.698708	-	-	-	-	
0.0739	-	-	10.84	0.668915	-	-	-	-	
-	-	-	8.688	0.428553	-	-	-	-	
0.868	-	0	15.91	1.522045	neutral(0.0	-	-	PEA3-type_	
0.00765	-	-1	29.7	4.156915	deleterious	-	-	Exostosin_	

0.00765	-	-	18.70	1.947023	-	-	-	-
0.0377	-	-	-	-	-	-	-	-
0.0135	-	-	5.465	0.174987	-	-	-	-
-	-	-	-	-	-	-	-	-
0.0821	-	-	0.638	-0.217210	-	-	-	-
0.0821	-	-	0.215	-0.375976	-	-	-	-
0.989	-	-2	27.1	3.902580	deleterious	-	-	-
0.989	-	-	-	-	-	-	-	-
0.0141	-	-	1.818	-0.059955	-	-	-	-
0.0563	-	-	38	7.225216	-	-	-	WD40-repe
0.00524	-	-	8.608	0.420816	-	-	-	-
0.00179	-	-2	32	4.342552	deleterious	-	-	Protein_kir
0.0225	-	-	-	-	-	-	-	-
0.0225	-	-	-	-	-	-	-	-
0.0501	-	-	3.467	0.052311	-	-	-	-
0.218	-	-	-	-	-	-	-	-
0.0227	-	1	12.18	0.842662	neutral(0.0	-	-	-
0.719	-	-	7.440	0.317651	-	-	-	-
0.719	-	1	24.8	3.403721	deleterious	-	-	Fibronectir
0.719	-	-	12.69	0.913562	-	-	-	-
-	-	-2	25.6	3.640798	deleterious	-	-	-
-	-	1	14.60	1.233916	neutral(0.0	-	-	Fork_head_
-	0.0611	Mental_ret	-	-	-	-	-	-
0.0749	-	0	32	4.301314	deleterious	-	-	Fork_head_
0.0431	-	-	0.660	-0.212137	-	-	-	-
-	-	-	12.45	0.879744	-	-	-	-
0.00834	-	-	21.7	2.256372	-	-	-	-
0.682	-	-	0.983	-0.152403	-	-	-	-
0.471	-	-2	29.8	4.168297	deleterious	-	-	-
0.0879	-	-	4.803	0.132698	-	-	-	-
0.17	-	-	-	-	-	-	-	-
0.567	-	2	22.8	2.627633	neutral(0.3	-	-	-
0.567	-	-	0.889	-0.167359	-	-	-	-
0.307	-	-3	25.9	3.709981	deleterious	-	-	Isopropylm
0.107	-	-	4.558	0.117619	-	-	-	-
0.107	-	-	4.557	0.117545	-	-	-	-
0.339	-	-	7.126	0.293305	-	-	-	-
0.339	-	1	17.05	1.743266	neutral(0.0	-	-	Mannose-6
0.698	-	-	11.91	0.805703	-	-	-	-
0.68	-	-	9.461	0.508429	-	-	-	-
0.442	-	1	15.27	1.376163	neutral(0.0	-	-	-
0.442	-	1	23.5	2.963806	deleterious	-	-	-
0.442	-	-	-	-	-	-	-	-
0.442	-	1	22.9	2.652036	neutral(0.0	-	-	Fibronectir
0.442	-	-	1.310	-0.110062	-	-	-	-
0.136	-	-	8.649	0.424760	-	-	-	-
-	-	-	12.05	0.825487	-	-	-	-

0.788	-	-	34	4.792552	-	-	-	-
0.788	-	-	17.26	1.774085	-	-	-	-
0.788	-	-1	25.8	3.683552	deleterious	-	-	von_Willeb
0.788	-	-	-	-	-	-	-	-
0.555	-	-	34	4.735569	-	-	-	-
0.555	-	-	3.487	0.053510	-	-	-	-
0.0333	-	-	-	-	-	-	-	-
0.127	-	-2	23.9	3.119135	deleterious	-	-	Integrin_be
0.127	-	-	5.928	0.205845	-	-	-	-
0.127	-	-3	29.1	4.107675	deleterious	-	-	Integrin_be
0.127	-	2	9.285	0.489452	neutral(0.3	-	-	Integrin_be
0.189	-	-	-	-	-	-	-	-
0.189	-	-	18.02	1.871992	-	-	-	-
-	-	-	-	-	-	-	-	-
T	-	blepharopl	-	-	-	-	-	-
-	-	2	22.9	2.658825	neutral(0.1	-	-	-
-	-	-	-	-	-	-	-	-
-	-	-2	25.6	3.628204	deleterious	-	-	Acyl-CoA_N
-	-	-2	24.6	3.353220	deleterious	-	-	Acyl-CoA_N
0.0636	-	1	21.2	2.185731	neutral(0.0	-	-	-
0.187	-	-	9.465	0.508878	-	-	-	-
0.196	-	-	-	-	-	-	-	-
0.196	-	-1	22.1	2.320052	neutral(0.3	-	-	Immunoglo
0.196	-	0	26.0	3.714263	deleterious	-	-	Immunoglo
0.196	-	-	-	-	-	-	-	-
0.303	-	-1	27.8	3.983225	deleterious	-	-	Kelch-type_
-	-	2	16.12	1.570001	neutral(0.0	-	-	-
0.19	-	-	-	-	-	-	-	-
0.794	-	1	14.37	1.187971	neutral(0.2	-	-	-
0.794	-	-	13.44	1.025156	-	-	-	-
0.794	-	-	9.779	0.543742	-	-	-	-
0.794	-	-	6.080	0.216398	-	-	-	-
0.794	-	1	16.18	1.581649	neutral(0.0	-	-	Fibronectir
0.1	-	-2	27.8	3.982775	deleterious	-	-	Olfactomec
0.1	-	2	29.4	4.131108	deleterious	-	-	GPCR,_fam
0.00916	-	-	3.691	0.065654	-	-	-	-
0.00916	-	-	-	-	-	-	-	-
0.00916	-	-	7.501	0.322523	-	-	-	-
0.00916	-	-	6.327	0.233782	-	-	-	-
0.00916	-	1	21.4	2.214939	neutral(0.0	-	-	Six-bladed_
0.00916	-	-2	19.89	2.066164	neutral(0.2	-	-	Six-bladed_
0.00916	-	-	14.54	1.222358	-	-	-	-
0.00916	-	1	22.2	2.353637	deleterious	-	-	Low-densit
0.00916	-	-	9.974	0.566107	-	-	-	-
0.00916	-	-	0.546	-0.240307	-	-	-	-
0.00916	-	-	17.08	1.746742	-	-	-	-
0.00916	-	-	-	-	-	-	-	-

0.943	-	-	26.4	3.792239	-	-	-	-
0.519	-	-	7.918	0.357593	-	-	-	-
-	-	0	20.4	2.101275	neutral(0.0	-	-	-
-	-	-	10.03	0.571590	-	-	-	-
-	-	-1	26.5	3.808703	deleterious	-	-	-
0.0532	-	0	23.5	2.955567	neutral(0.0	-	-	Protein_kir
0.503	-	-	-	-	-	-	-	-
0.0914	-	3	24.2	3.211385	neutral(0.3	-	-	KA1_doma
0.881	-	-	5.554	0.180810	-	-	-	-
0.0163	-	-	9.155	0.475720	-	-	-	-
-	-	-3	28.5	4.051832	deleterious	-	-	Apoptosis_
-	-	-	7.137	0.294141	-	-	-	-
0.442	-	2	22.6	2.535191	deleterious	-	-	Sema_dom
0.442	-	-2	24.2	3.210616	deleterious	-	-	IPT_domai
0.203	-	-	14.72	1.258585	-	-	-	-
0.203	-	-	3.594	0.059874	-	-	-	-
0.00165	-	-2	31	4.201806	deleterious	-	-	DNA_mism
-	-	-1	23.6	2.979099	deleterious	-	-	-
-	-	0	27.9	3.991116	deleterious	-	-	-
-	-	-	-	-	-	-	-	-
-	-	-	0.079	-0.513979	-	-	-	-
-	-	-	-	-	-	-	-	-
-	-	-	9.015	0.461254	-	-	-	-
-	-	-	3.939	0.080365	-	-	-	-
TT	-	Intellectual	-	-	-	-	-	-
-	-	1	22.7	2.566067	neutral(0.3	-	-	-
-	-	-	3.826	0.073684	-	-	-	-
-	0.336	Precursor_	-	-	-	-	-	-
0.336	-	-	3.475	0.052780	-	-	-	-
0.336	-	-	10.91	0.678125	-	-	-	-
0.343	-	-	16.85	1.710423	-	-	-	-
0.343	-	-	6.987	0.282799	-	-	-	-
0.21	-	2	23.8	3.088828	deleterious	-	-	Calcineurin
0.00971	-	-	36	6.503360	-	-	-	DNA_mism
0.00971	-	-	0.191	-0.392294	-	-	-	-
0.00971	-	-1	24.5	3.325596	neutral(0.4	-	-	DNA_mism
0.00971	-	-	-	-	-	-	-	-
0.246	-	-	7.909	0.356795	-	-	-	-
0.507	-	-2	-	-	neutral(0.3	-	-	-
0.507	-	-	9.003	0.460082	-	-	-	-
0.507	-	-	0.074	-0.522855	-	-	-	-
0.507	-	-2	29.5	4.137936	deleterious	-	-	Vitamin_B1
-	-	-	-	-	-	-	-	-
-	-	-	7.305	0.307075	-	-	-	-
0.0243	-	-	35	5.707665	-	-	-	-
-	-	-	-	-	-	-	-	-
-	-	-	6.258	0.228872	-	-	-	-

-	-	-1	23.0	2.704582	deleterious	-	-	Transcriptic
0.0238	-	-	19.97	2.073351	-	-	-	-
0.00613	-	-1	22.5	2.450798	neutral(0.0	-	-	Myosin_tai
0.00613	-	-	5.592	0.183322	-	-	-	-
0.00606	-	-	-	-	-	-	-	-
-	-	-	0.782	-0.186567	-	-	-	-
-	-	-1	29.5	4.135182	deleterious	-	-	Nuclear_re
-	-	-	0.947	-0.158037	-	-	-	-
-	-	-2	22.7	2.554222	deleterious	-	-	-
-	-	-3	23.8	3.076697	deleterious	-	-	-
0.729	-	1	22.9	2.641645	deleterious	-	-	-
0.729	-	-	-	-	-	-	-	-
0.116	-	-	0.502	-0.252733	-	-	-	-
-	0.116	Familial_sp	-	-	-	-	-	-
0.116	-	1	23.7	3.049200	neutral(0.4	-	-	Armadillo-l
0.199	-	-1	27.6	3.960079	deleterious	-	-	-
0.307	-	0	14.81	1.276887	neutral(0.0	-	-	Ankyrin_re
0.307	-	-	5.720	0.191786	-	-	-	-
0.307	-	-1	21.7	2.251376	neutral(0.0	-	-	Ankyrin_re
0.307	-	-3	24.8	3.421374	deleterious	-	-	Ankyrin_re
0.18	-	-	12.28	0.856663	-	-	-	-
0.943	-	-1	22.7	2.538545	deleterious	-	-	-
0.943	-	-2	29.5	4.134348	deleterious	-	-	-
0.926	-	-2	13.34	1.008306	neutral(0.0	-	-	DAPIN_dor
0.0156	-	-	2.212	-0.028634	-	-	-	-
0.0156	-	1	21.6	2.232939	neutral(0.3	-	-	EGF-like_ca
0.138	-	-	22.0	2.311136	-	-	-	-
0.138	-	-1	22.7	2.565874	neutral(0.3	-	-	-
0.00186	-	-3	7.774	0.345199	neutral(0.0	-	-	PWWP_dor
0.239	-	1	24.8	3.423621	deleterious	-	-	Protein_kir
0.0395	-	0	25.6	3.637826	deleterious	-	-	Immunoglo
0.0394	-	0	23.5	2.950345	neutral(0.1	-	-	-
0.247	-	-	18.92	1.971145	-	-	-	-
0.00393	-	-	14.17	1.150139	-	-	-	-
0.173	-	-	8.905	0.450156	-	-	-	-
0.0277	-	-	3.724	0.067609	-	-	-	-
0.00758	-	-1	22.7	2.542591	neutral(0.1	-	-	Homeobox
0.0745	-	-1	29.0	4.094349	deleterious	-	-	Bromo_adj
0.0745	-	-2	32	4.266889	deleterious	-	-	Bromo_adj
0.984	-	-	11.21	0.714842	-	-	-	-
0.984	-	1	19.91	2.068006	deleterious	-	-	-
0.984	-	3	18.90	1.968324	neutral(0.0	-	-	-
0.984	-	-	17.73	1.836965	-	-	-	-
0.984	-	-	35	6.029872	-	-	-	-
0.984	-	-2	-	-	deleterious	-	-	-
0.984	-	-2	29.3	4.121785	deleterious	-	-	-
0.984	-	-3	26.7	3.841075	deleterious	-	-	-

0.984	-	-2	23.6	2.999948	deleterious	-	-	-	
0.984	-	-	-	-	-	-	-	-	
0.984	-	0	25.1	3.509040	deleterious	-	-	-	
0.984	-	-3	27.4	3.931768	deleterious	-	-	-	
0.984	-	-	16.46	1.639154	-	-	-	-	
0.984	-	-	0.283	-0.336353	-	-	-	-	
0.984	-	1	24.0	3.145100	neutral(0.4	-	-	-	
0.984	-	-	11.73	0.782126	-	-	-	-	
0.984	-	-	13.89	1.100022	-	-	-	-	
0.984	-	-	22.1	2.339766	-	-	-	-	
0.984	-	0	24.2	3.225818	neutral(0.3	-	-	Centrosom	
0.984	-	-2	33	4.348391	deleterious	-	-	Centrosom	
0.984	-	1	26.1	3.743393	deleterious	-	-	Centrosom	
0.125	-	-	3.624	0.061688	-	-	-	-	
0.125	-	-1	15.96	1.533929	neutral(0.0	-	-	Immunoglc	
0.123	-	-	7.155	0.295555	-	-	-	-	
0.123	-	-3	21.8	2.262483	neutral(0.2	-	-	Immunoglc	
0.55	-	-	-	-	-	-	-	-	
0.455	-	-1	23.5	2.921353	neutral(0.2	-	-	-	
0.268	-	-	13.34	1.009527	-	-	-	-	
0.268	-	-	-	-	-	-	-	-	
0.313	-	-2	22.5	2.453366	neutral(0.0	-	-	Ubiquitin-r	
0.49	-	-	2.599	-0.001577	-	-	-	-	
0.295	-	-	40	7.633873	-	-	-	Phosphatid	
0.295	-	-	39	7.558851	-	-	-	PI3K_p85_	
0.00772	-	-3	2.935	0.019984	neutral(0.0	-	-	-	
0.00772	-	-2	24.5	3.313009	deleterious	-	-	Pectin_lyas	
0.00772	-	-	35	5.402483	-	-	-	-	
0.00772	-	0	1.813	-0.060414	neutral(0.0	-	-	-	
0.00772	-	-	-	-	-	-	-	-	
0.00772	-	3	14.48	1.209674	neutral(0.0	-	-	-	
0.0827	-	-	2.237	-0.026767	-	-	-	-	
0.0827	-	1	16.18	1.582987	neutral(0.0	-	-	High_mobi	
0.382	-	-	0.258	-0.349763	-	-	-	-	
0.382	-	-	41	7.841557	-	-	-	DNA_mism	
0.382	-	-	5.678	0.189001	-	-	-	-	
0.832	-	-	0.131	-0.444509	-	-	-	-	
0.0537	-	-	0.516	-0.248736	-	-	-	-	
-	-	-	5.377	0.169296	-	-	-	-	
-	-	-2	28.2	4.021655	deleterious	-	-	PIK-related	
-	-	-	9.229	0.483530	-	-	-	-	
-	-	-	6.896	0.275833	-	-	-	-	
-	-	-	5.897	0.203741	-	-	-	-	
0.000344	-	-2	25.7	3.648875	deleterious	-	-	-	
0.000344	-	-	3.141	0.032690	-	-	-	-	
-	-	-	1.575	-0.082245	-	-	-	-	
-	-	-	23.5	2.931204	-	-	-	-	

0.0482	-	-	-	-	-	-	-	-
0.0868	-	-	-	-	-	-	-	-
0.0868	-	-	9.257	0.486494	-	-	-	-
0.0868	-	0	22.7	2.565591	neutral(0.2	-	-	Fibronectir
0.0898	-	-	3.980	0.082794	-	-	-	-
0.987	-	-2	26.8	3.853747	deleterious	-	-	-
0.184	-	-	21.1	2.177613	-	-	-	-
0.184	-	-	47	8.606048	-	-	-	Protein_kir
0.184	-	-	-	-	-	-	-	-
0.515	-	1	24.4	3.278076	deleterious	-	-	Ras_guanir
0.0723	-	-	5.141	0.154077	-	-	-	-
0.43	-	-	3.057	0.027540	-	-	-	-
0.286	-	-	-	-	-	-	-	-
0.286	-	2	29.1	4.105709	deleterious	-	-	-
0.223	-	-	5.507	0.177746	-	-	-	-
0.223	-	-	-	-	-	-	-	-
0.208	-	2	0.379	-0.294293	neutral(0.0	-	-	-
0.208	-	-	3.854	0.075321	-	-	-	-
0.208	-	-2	5.790	0.196477	neutral(0.3	-	-	-
0.208	-	-	35	5.110141	-	-	-	-
0.208	-	-	5.113	0.152244	-	-	-	-
0.208	-	-	-	-	-	-	-	-
0.134	-	2	23.0	2.701035	deleterious	-	-	-
-	-	-	3.880	0.076908	-	-	-	-
0.31	-	-2	32	4.286540	deleterious	-	-	Zinc_finger
0.31	-	-2	28.6	4.059482	deleterious	-	-	-
0.31	-	-3	28.5	4.047912	deleterious	-	-	-
0.432	-	-	6.616	0.254695	-	-	-	-
0.0294	-	-3	29.7	4.151683	deleterious	-	-	AWS_doma
0.0294	-	0	15.81	1.500287	neutral(0.2	-	-	-
-	-	-	12.16	0.840407	-	-	-	-
0.43	-	-	8.602	0.420246	-	-	-	-
0.43	-	-1	21.4	2.210603	neutral(0.2	-	-	Phox_hom
0.116	-	3	26.2	3.759026	deleterious	-	-	MAD_hom
0.116	-	-	11.75	0.785729	-	-	-	-
0.000689	-	-	10.16	0.586993	-	-	-	-
-	-	-	-	-	-	-	-	-
-	-	-	7.265	0.303968	-	-	-	-
-	-	-	-	-	-	-	-	-
-	-	0	23.5	2.944635	neutral(0.0	-	-	-
0.0217	-	-	4.082	0.088868	-	-	-	-
0.0217	-	-	6.038	0.213436	-	-	-	-
0.886	-	-3	13.15	0.980109	neutral(0.3	-	-	Armadillo-l
-	-	-	12.47	0.881866	-	-	-	-
0.00792	-	-1	26.0	3.719458	deleterious	-	-	SH2_doma
0.881	-	-	8.632	0.423201	-	-	-	-
0.881	-	-	57	9.847167	-	-	-	-

0.881	-	1	26.3	3.770044	-	-	-	-
0.881	-	-	7.620	0.332311	-	-	-	-
0.881	-	2	23.9	3.118009	-	-	-	-
0.881	-	0	26.9	3.874711	-	-	-	-
0.881	-	-	2.362	-0.017757	-	-	-	-
0.881	-	0	21.6	2.230064	-	-	-	-
0.881	-	-	0.143	-0.433131	-	-	-	-
0.881	-	-1	16.65	1.676785	-	-	-	-
0.881	-	1	16.22	1.591346	-	-	-	-
0.881	-	-3	22.8	2.589760	-	-	-	-
0.881	-	-	8.548	0.415087	-	-	-	-
0.152	-	1	12.57	0.895578	neutral(0.0	-	-	-
0.152	-	-2	21.3	2.193336	neutral(0.0	-	-	-
0.582	-	-3	17.63	1.823622	neutral(0.3	-	-	Bromodom
0.0586	-	0	5.445	0.173697	neutral(0.0	-	-	p53-like_tr
0.497	-	-	-	-	-	-	-	-
0.118	-	-	-	-	-	-	-	-
0.0268	-	1	23.6	3.002021	deleterious	-	-	CTNNB1_bi
0.0268	-	-1	26.5	3.818797	deleterious	-	-	CTNNB1_bi
0.247	-	1	22.8	2.590147	neutral(0.4	-	-	-
0.247	-	-	6.859	0.273003	-	-	-	-
0.247	-	-3	26.6	3.820431	deleterious	-	-	-
0.998	-	-2	13.74	1.074132	neutral(0.3	-	-	-
0.998	-	-	-	-	-	-	-	-
0.192	-	0	18.56	1.932384	neutral(0.0	-	-	-
0.104	-	0	23.4	2.882177	deleterious	-	-	-
C	0.000965	ADRENOCC	-	-	-	-	-	-
0.361	-	1	23.4	2.897938	neutral(0.3	-	-	-
0.361	-	-	11.81	0.793127	-	-	-	-
0.361	-	-	-	-	-	-	-	-
0.028	-	-	16.25	1.598130	-	-	-	-
0.028	-	-	16.32	1.612783	-	-	-	-
0.9	-	-	-	-	-	-	-	-
0.9	-	-	4.369	0.106133	-	-	-	-
0.9	-	1	23.6	2.970558	deleterious	-	-	-
0.0406	-	-	17.56	1.814685	-	-	-	-
0.0406	-	2	18.37	1.911581	neutral(0.0	-	-	Armadillo-l
0.0406	-	-	43	8.183281	-	-	-	Armadillo-l
0.0406	-	0	28.8	4.075952	deleterious	-	-	Armadillo-l
0.0406	-	-	0.790	-0.184942	-	-	-	-
0.000276	-	-	-	-	-	-	-	-
0.272	-	-	4.415	0.108894	-	-	-	-
0.272	-	-2	26.7	3.841200	deleterious	-	-	Leucine-ric
0.272	-	-	16.87	1.713805	-	-	-	-
0.0612	-	1	32	4.240419	deleterious	-	-	HECT_dom
0.0612	-	1	21.7	2.248015	neutral(0.0	-	-	-
0.0612	-	-	12.38	0.870098	-	-	-	-

0.0612	-	1	23.6	3.002175	neutral(0.4	-	-	-
0.0612	-	-	-	-	-	-	-	-
0.0612	-	-	10.76	0.658659	-	-	-	-
-	-	-	12.56	0.895252	-	-	-	-
0.00276	-	-	38	7.200462	-	-	-	AWS_doma
0.00276	-	-	12.54	0.891654	-	-	-	-
0.00276	-	-	13.54	1.041425	-	-	-	-
0.00276	-	-	-	-	-	-	-	-
0.00276	-	-	12.68	0.911763	-	-	-	-
0.988	-	-	-	-	-	-	-	-
0.988	-	-	0.333	-0.312875	-	-	-	-
0.988	-	-	8.520	0.412391	-	-	-	-
0.988	-	-3	8.988	0.458480	neutral(0.4	-	-	-
0.197	-	-	-	-	-	-	-	-
0.197	-	-	9.900	0.557496	-	-	-	-

clinvar_cln:	clinvar_gol	clinvar_rs	clinvar_trai	codon_deg	PHENOTYP	AA	MPC	large_intes
-	-	-	-	0	-	G	-	NR
-	-	-	-	-	-	G	-	NR
-	-	-	-	-	-	A	-	NR
-	-	-	-	-	-	C	-	NR
-	-	-	-	0	-	A	0.2906501(	NR
-	-	-	-	-	LONG_QT_	CAA	-	NR
-	-	-	-	0	Long_QT_s	T	0.3477824	NR
-	-	-	-	-	-	T	-	NR
-	-	-	-	-	LONG_QT_	-	-	NR
-	-	-	-	0	-	T	0.3090637	NR
-	-	-	-	0	-	C	0.2533757	NR
-	-	-	-	0	-	G	0.8409546	NR
-	-	-	-	-	Hemimega	T	-	NR
-	-	-	-	-	-	A	-	NR
-	-	-	-	0	-	G	0.0700097	NR
-	-	-	-	-	DESMOID_	T	-	NR
-	-	-	-	-	DESMOID_	GT	-	NR
-	-	-	-	-	-	A	-	NR
-	-	-	-	-	Hereditary_	ACCACCTC(	-	NR
-	-	-	-	-	-	A	-	NR
-	-	-	-	-	COFFIN-SIR	A	-	NR
-	-	-	-	0	-	A	0.4713935	NR
-	-	-	-	2	-	T	-	NR
-	-	-	-	-	-	G	-	NR
-	-	-	-	0	-	C	1.5228717(	NR
-	-	-	-	0	-	A	0.1531653	NR
-	-	-	-	-	-	G	-	NR
-	-	-	-	-	ATAXIA-TEI	ATT	-	NR
-	-	-	-	-	-	T	-	NR
-	-	-	-	-	-	T	-	NR
-	-	-	-	-	SECKEL_SY	T	-	NR
-	-	-	-	-	-	C	-	NR
-	-	-	-	-	-	T	-	NR
-	-	-	-	0	-	C	0.1763426	NR
-	-	-	-	-	-	T	-	NR
-	-	-	-	-	-	C	-	NR
-	-	-	-	-	-	C	-	NR
-	-	-	-	-	-	-	-	NR
-	-	-	-	-	-	T	-	NR
-	-	-	-	-	-	C	-	NR
-	-	-	-	0	-	G	0.2164381	NR
-	-	-	-	0	-	A	0.1351329(	NR
-	-	-	-	-	MESOTHEL	A	-	NR
-	-	-	-	-	-	G	-	NR
-	-	-	-	-	-	A	-	NR

-	-	-	-	0	-	G	0.1769168	NR
-	-	-	-	0	-	C	-	NR
-	-	-	-	-	-	A	-	NR
-	-	-	-	0	MALT_lym	A	0.2731034	NR
-	-	-	-	0	-	G	0.4682574	NR
-	-	-	-	-	-	T	-	NR
-	-	-	-	0	-	C	0.6778984	NR
-	-	-	-	2	-	A	0.6207210	NR
-	-	-	-	-	-	G	-	NR
-	-	-	-	-	-	C	-	NR
-	-	-	-	2	-	A	0.1092634	NR
-	-	-	-	0	-	C	0.4957713	NR
-	-	-	-	-	-	-	-	NR
-	-	-	-	-	FANCONI_	T	-	NR
-	-	-	-	-	-	G	-	NR
-	-	-	-	-	-	T	-	NR
-	-	-	-	0	-	T	0.5194465	NR
-	-	-	-	2	-	G	0.0745868	NR
-	-	-	-	-	-	TTTT	-	NR
-	-	-	-	-	-	-	-	NR
-	-	-	-	-	HYPERPAR	A	-	NR
-	-	-	-	-	-	g	-	NR
-	-	-	-	-	-	C	-	NR
-	-	-	-	-	-	T	-	NR
-	-	-	-	-	-	G	-	NR
-	-	-	-	2	-	C	0.2701084	NR
-	-	-	-	0	-	G	0.3653853	NR
-	-	-	-	0	-	T	1.2840433	NR
-	-	-	-	-	MELANOM	AGG	-	NR
-	-	-	-	-	-	T	-	NR
-	-	-	-	-	CAPICUA__	C	-	NR
-	-	-	-	-	-	A	-	NR
-	-	-	-	-	-	-	-	NR
-	-	-	-	2	-	A	0.0989413	NR
-	-	-	-	-	-	C	-	NR
-	-	-	-	-	-	T	-	NR
-	-	-	-	-	-	------Cg	-	NR
-	-	-	-	-	-	ATT	-	NR
-	-	-	-	-	-	T	-	NR
-	-	-	-	-	-	A	-	NR
-	-	-	-	-	-	T	-	NR
-	-	-	-	0	-	A	1.3629024	NR
-	-	-	-	-	Butterfly-s	AGA	-	NR
-	-	-	-	2	-	C	2.2137141	NR
-	-	-	-	0	-	A	0.0059104	NR
-	-	-	-	0	-	C	0.1638847	NR
-	-	-	-	0	-	T	0.2893329	NR

-	-	-	-	0	-	G	0.7372215	NR
-	-	-	-	0	-	G	0.7217725	NR
-	-	-	-	-	-	A	-	NR
-	-	-	-	-	-	A	-	NR
-	-	-	-	-	SPONDYLO	GC	-	NR
-	-	-	-	0	-	G	0.9148740	NR
-	-	-	-	-	-	G	-	NR
-	-	-	-	2	Overgrowt	T	-	NR
-	-	-	-	0	-	G	0.9638527	NR
-	-	-	-	-	-	T	-	NR
-	-	-	-	-	-	A	-	NR
-	-	-	-	-	-	A	-	NR
-	-	-	-	-	-	C	-	NR
-	-	-	-	0	-	T	1.3485295	NR
-	-	-	-	0	-	A	1.5162887	NR
-	-	-	-	-	-	A	-	NR
-	-	-	-	0	neutral(0.4	-1	0.167898	NR
-	-	-	-	0	-	A	0.6866158	NR
-	-	-	-	-	-	AAA	-	NR
-	-	-	-	-	-	G	-	NR
-	-	-	-	-	-	TTACTC	-	NR
-	-	-	-	-	RUBINSTEI	T	-	NR
-	-	-	-	-	-	T	-	NR
-	-	-	-	0	-	G	0.1804570	NR
-	-	-	-	0	-	G	0.4499171	NR
-	-	-	-	-	-	C	-	NR
-	-	-	-	-	-	A	-	NR
-	-	-	-	0	-	A	0.3657255	NR
-	-	-	-	0	-	A	1.5168048	NR
-	-	-	-	-	-	T	-	NR
-	-	-	-	-	-	T	-	NR
-	-	-	-	0	-	A	0.2473641	NR
-	-	-	-	-	-	T	-	NR
-	-	-	-	-	-	T	-	NR
-	-	-	-	-	-	T	-	NR
-	-	-	-	-	-	C	-	NR
-	-	-	-	-	-	G	-	NR
-	-	-	-	-	-	G	-	NR
-	-	-	-	-	-	C	-	NR
-	-	-	-	-	-	C	-	NR
-	-	-	-	-	-	G	-	NR
-	-	-	-	-	-	G	-	NR
-	-	-	-	-	-	G	-	NR
-	-	-	-	-	-	t	-	NR
-	-	-	-	-	-	G	-	NR
-	-	-	-	2	-	C	0.3247939	NR
-	-	-	-	0	-	T	1.1959237	NR

-	-	-	-	-	-	G	-	NR
-	-	-	-	-	Exostoses_	A	-	NR
-	-	-	-	-	-	T	-	NR
-	-	-	-	-	OSTEOPATI	-	-	NR
-	-	-	-	-	-	G	-	NR
-	-	-	-	-	-	C	-	NR
-	-	-	-	0	-	a	0.5695223	NR
-	-	-	-	-	FANCONI_	A	-	NR
-	-	-	-	-	-	T	-	NR
-	-	-	-	0	-	C	-	NR
-	-	-	-	-	Encephaloc	T	-	NR
-	-	-	-	0	-	T	1.9310558	NR
-	-	-	-	-	ACHONDR(	c	-	NR
-	-	-	-	-	-	c	-	NR
-	-	-	-	-	-	G	-	NR
-	-	-	-	-	-	CT	-	NR
-	-	-	-	0	-	C	0.1145077	NR
-	-	-	-	-	-	A	-	NR
-	-	-	-	2	-	G	0.9135416	NR
-	-	-	-	-	-	C	-	NR
-	-	-	-	0	-	A	1.0181911	NR
-	-	-	-	0	-	A	-	NR
-	-	-	-	-	-	-	-	NR
-	-	-	-	0	-	C	2.2043923	NR
-	-	-	-	-	-	C	-	NR
-	-	-	-	-	-	G	-	NR
-	-	-	-	-	-	C	-	NR
-	-	-	-	-	-	C	-	NR
-	-	-	-	0	-	G	1.2823345	NR
-	-	-	-	-	-	g	-	NR
-	-	-	-	-	-	TTTTTT	-	NR
-	-	-	-	2	-	.	-	NR
-	-	-	-	-	-	.	-	NR
-	-	-	-	0	-	C	1.1614020	NR
-	-	-	-	-	INSULIN-LII	A	-	NR
-	-	-	-	-	-	G	-	NR
-	-	-	-	-	-	A	-	NR
-	-	-	-	0	-	A	0.4566986	NR
-	-	-	-	-	-	T	-	NR
-	-	-	-	-	-	A	-	NR
-	-	-	-	0	-	A	0.1014807	NR
-	-	-	-	0	-	C	0.3698293	NR
-	-	-	-	-	-	GAAA	-	NR
-	-	-	-	0	-	C	0.1605978	NR
-	-	-	-	-	-	T	-	NR
-	-	-	-	-	-	C	-	NR
-	-	-	-	-	-	.	-	NR

-	-	-	-	0	-	G	-	NR
-	-	-	-	-	-	G	-	NR
-	-	-	-	0	-	C	0.7427756(	NR
-	-	-	-	-	-	C	-	NR
-	-	-	-	-	-	A	-	NR
-	-	-	-	-	-	C	-	NR
-	-	-	-	-	LEUKOCYTE	-	-	NR
-	-	-	-	0	-	C	1.0143853	NR
-	-	-	-	-	-	G	-	NR
-	-	-	-	0	-	T	1.6734562	NR
-	-	-	-	2	-	C	0.6795728	NR
-	-	-	-	-	BUDD-CHIA	CT	-	NR
-	-	-	-	-	-	A	-	NR
-	-	-	-	-	MENTAL_R	-	-	NR
-	-	-	-	-	-	-	-	NR
-	-	-	-	0	-	T	0.6413880	NR
-	-	-	-	-	blepharoph	A	-	NR
-	-	-	-	0	-	A	2.0894183(	NR
-	-	-	-	0	-	A	1.3614556	NR
-	-	-	-	0	-	C	1.3134170	NR
-	-	-	-	-	-	T	-	NR
-	-	-	-	-	-	CA	-	NR
-	-	-	-	0	-	T	0.1914086(	NR
-	-	-	-	2	-	C	0.2259192(	NR
-	-	-	-	-	HEMANGIC	-	-	NR
-	-	-	-	0	-	G	2.1456674	NR
-	-	-	-	0	Gastric_car	T	1.7162338	NR
-	-	-	-	-	CARDIOFA(	-	-	NR
-	-	-	-	0	Stuve-Wiec	T	0.0896114	NR
-	-	-	-	-	-	G	-	NR
-	-	-	-	-	-	A	-	NR
-	-	-	-	-	-	G	-	NR
-	-	-	-	0	-	G	0.0776255	NR
-	-	-	-	0	-	A	1.1262604(	NR
-	-	-	-	2	-	C	0.8299129	NR
-	-	-	-	-	-	T	-	NR
-	-	-	-	-	-	AT	-	NR
-	-	-	-	-	-	C	-	NR
-	-	-	-	-	-	T	-	NR
-	-	-	-	0	-	C	0.2585019	NR
-	-	-	-	0	-	A	0.7189395	NR
-	-	-	-	-	-	A	-	NR
-	-	-	-	2	-	A	0.7103614	NR
-	-	-	-	-	-	C	-	NR
-	-	-	-	-	-	G	-	NR
-	-	-	-	-	-	A	-	NR
-	-	-	-	-	-	GG	-	NR

-	-	-	-	-	-	C	-	NR
-	-	-	-	-	-	C	-	NR
-	-	-	-	0	-	T	0.0572773	NR
-	-	-	-	-	-	G	-	NR
-	-	-	-	0	-	G	0.3860696	NR
-	-	-	-	0	-	C	0.3789311	NR
-	-	-	-	-	-	TT	-	NR
-	-	-	-	0	-	T	0.7752389	NR
-	-	-	-	-	-	A	-	NR
-	-	-	-	-	-	A	-	NR
-	-	-	-	0	-	A	2.0413507	NR
-	-	-	-	-	-	G	-	NR
-	-	-	-	2	-	T	0.2384410	NR
-	-	-	-	0	-	C	0.9213357	NR
-	-	-	-	-	-	T	-	NR
-	-	-	-	-	-	T	-	NR
-	-	-	-	0	-	T	0.5094316	NR
-	-	-	-	0	-	G	1.3286609	NR
-	-	-	-	0	-	G	2.6264660	NR
-	-	-	-	-	-	-	-	NR
-	-	-	-	-	-	G	-	NR
-	-	-	-	-	-	T	-	NR
-	-	-	-	-	-	a	-	NR
-	-	-	-	-	-	A	-	NR
-	-	-	-	-	-	-	-	NR
-	-	-	-	0	-	C	0.0402290	NR
-	-	-	-	-	-	G	-	NR
-	-	-	-	-	-	-	-	NR
-	-	-	-	-	-	G	-	NR
-	-	-	-	-	-	A	-	NR
-	-	-	-	-	AMEGAKAF	C	-	NR
-	-	-	-	-	-	C	-	NR
-	-	-	-	2	-	T	0.4466681	NR
-	-	-	-	2	Lynch_sync	A	-	NR
-	-	-	-	-	-	A	-	NR
-	-	-	-	0	-	A	0.0104087	NR
-	-	-	-	-	Endometri	ACTTCT	-	NR
-	-	-	-	-	Epileptic_e	A	-	NR
-	-	-	-	-	METHYLCO	GG	-	NR
-	-	-	-	-	-	C	-	NR
-	-	-	-	-	-	A	-	NR
-	-	-	-	0	-	A	1.3526383	NR
-	-	-	-	-	MEDULLAR	ACT	-	NR
-	-	-	-	-	-	A	-	NR
-	-	-	-	2	-	G	-	NR
-	-	-	-	-	BURKITT_L	-	-	NR
-	-	-	-	-	-	G	-	NR

-	-	-	-	2	-	C	1.6806760[illegible]	NR
-	-	-	-	-	-	G	-	NR
-	-	-	-	0	-	A	0.3338483[illegible]	NR
-	-	-	-	-	-	G	-	NR
-	-	-	-	-	DEAFNESS_	GT	-	NR
-	-	-	-	-	-	C	-	NR
-	-	-	-	0	-	T	0.5413976[illegible]	NR
-	-	-	-	-	-	A	-	NR
-	-	-	-	2	-	G	2.3080619[illegible]	NR
-	-	-	-	0	-	A	2.3684937[illegible]	NR
-	-	-	-	0	-	.	0.1924514[illegible]	NR
-	-	-	-	-	THYROID_[illegible]	-	-	NR
-	-	-	-	-	-	G	-	NR
-	-	-	-	-	-	-	-	NR
-	-	-	-	2	-	T	1.3981076[illegible]	NR
-	-	-	-	0	-	A	0.2201344[illegible]	NR
-	-	-	-	2	-	T	0.4513656[illegible]	NR
-	-	-	-	-	-	C	-	NR
-	-	-	-	0	-	C	0.4830688[illegible]	NR
-	-	-	-	0	-	G	0.5458653[illegible]	NR
-	-	-	-	-	Common_[illegible]	C	-	NR
-	-	-	-	0	-	C	0.4693517[illegible]	NR
-	-	-	-	2	-	T	0.6482529[illegible]	NR
-	-	-	-	0	-	T	0.2903945[illegible]	NR
-	-	-	-	-	-	G	-	NR
-	-	-	-	0	-	G	1.4443601[illegible]	NR
-	-	-	-	-	-	G	-	NR
-	-	-	-	0	-	G	1.6277975[illegible]	NR
-	-	-	-	0	-	T	0.0907135[illegible]	NR
-	-	-	-	0	-	G	0.3222460[illegible]	NR
-	-	-	-	0	-	G	0.8365473[illegible]	NR
-	-	-	-	0	-	G	0.5017323[illegible]	NR
-	-	-	-	-	-	A	-	NR
-	-	-	-	-	-	T	-	NR
-	-	-	-	-	-	g	-	NR
-	-	-	-	-	-	C	-	NR
-	-	-	-	0	-	C	0.8428833[illegible]	NR
-	-	-	-	0	-	A	1.1870528[illegible]	NR
-	-	-	-	0	-	A	1.4622042[illegible]	NR
-	-	-	-	-	-	.	-	NR
-	-	-	-	0	-	.	-	NR
-	-	-	-	0	-	.	-	NR
-	-	-	-	-	-	.	-	NR
-	-	-	-	0	-	.	-	NR
-	-	-	-	-	-	..	-	NR
-	-	-	-	0	-	.	-	NR
-	-	-	-	0	-	.	-	NR

-	-	-	-	-	-	.	-	NR
-	-	-	-	-	-	.	-	NR
-	-	-	-	0	-	.	-	NR
-	-	-	-	0	-	.	-	NR
-	-	-	-	-	-	.	-	NR
-	-	-	-	-	-	.	-	NR
-	-	-	-	0	-	T	-	NR
-	-	-	-	-	-	C	-	NR
-	-	-	-	-	-	A	-	NR
-	-	-	-	-	-	T	-	NR
-	-	-	-	0	-	C	-	NR
-	-	-	-	0	-	C	-	NR
-	-	-	-	0	-	T	-	NR
-	-	-	-	-	-	T	-	NR
-	-	-	-	0	-	A	0.4239555(	NR
-	-	-	-	-	-	A	-	NR
-	-	-	-	0	-	C	0.6045177:	NR
-	-	-	-	-	-	AA	-	NR
-	-	-	-	0	-	A	0.8044782:	NR
-	-	-	-	-	-	C	-	NR
-	-	-	-	-	-	CA	-	NR
-	-	-	-	0	-	A	1.4797054:	NR
-	-	-	-	-	-	A	-	NR
-	-	-	-	0	-	G	-	NR
-	-	-	-	0	AGAMMA(	G	-	NR
-	-	-	-	0	-	A	0.1022885!	NR
-	-	-	-	0	-	G	0.4195438(	NR
-	-	-	-	2	-	T	-	NR
-	-	-	-	2	-	C	0.0577316(	NR
-	-	-	-	-	POLYCYSTI(	-	-	NR
-	-	-	-	0	-	C	0.0512959(	NR
-	-	-	-	-	-	A	-	NR
-	-	-	-	0	-	G	0.0756139!	NR
-	-	-	-	-	Hereditary_	A	-	NR
-	-	-	-	0	-	A	-	NR
-	-	-	-	-	MISMATCH	t	-	NR
-	-	-	-	-	-	T	-	NR
-	-	-	-	-	Acrodysost	C	-	NR
-	-	-	-	-	Severe_cor	T	-	NR
-	-	-	-	0	-	C	0.7375075!	NR
-	-	-	-	-	-	A	-	NR
-	-	-	-	-	-	G	-	NR
-	-	-	-	-	-	A	-	NR
-	-	-	-	0	-	G	1.3254360	NR
-	-	-	-	-	-	T	-	NR
-	-	-	-	-	-	C	-	NR
-	-	-	-	-	-	A	-	NR

-	-	-	-	-	LEOPARD_	-	-	NR
-	-	-	-	-	-	A	-	NR
-	-	-	-	-	-	A	-	NR
-	-	-	-	0	-	C	-	NR
-	-	-	-	-	-	T	-	NR
-	-	-	-	0	-	C	0.7868148	NR
-	-	-	-	-	-	C	-	NR
-	-	-	-	2	-	G	-	NR
-	-	-	-	-	NOONAN_	AA	-	NR
-	-	-	-	0	-	C	0.2981488	NR
-	-	-	-	-	-	T	-	NR
-	-	-	-	-	-	G	-	NR
-	-	-	-	-	-	A	-	NR
-	-	-	-	0	-	A	1.5441570	NR
-	-	-	-	-	-	T	-	NR
-	-	-	-	-	-	T	-	NR
-	-	-	-	2	-	A	0.3205994	NR
-	-	-	-	-	-	T	-	NR
-	-	-	-	0	-	a	0.3843406	NR
-	-	-	-	0	-	C	-	NR
-	-	-	-	-	-	A	-	NR
-	-	-	-	-	MOYAMOY	-	-	NR
-	-	-	-	2	-	T	0.0255764	NR
-	-	-	-	-	-	C	-	NR
-	-	-	-	2	Colorectal_	G	2.0327957	NR
-	-	-	-	0	-	G	1.2888031	NR
-	-	-	-	0	-	C	2.2975458	NR
-	-	-	-	-	-	T	-	NR
-	-	-	-	2	-	G	2.6048549	NR
-	-	-	-	0	-	A	0.3306858	NR
-	-	-	-	-	-	T	-	NR
-	-	-	-	-	-	C	-	NR
-	-	-	-	0	-	G	0.0424580	NR
-	-	-	-	0	Juvenile_p	A	2.6732094	NR
-	-	-	-	-	-	T	-	NR
-	-	-	-	-	-	C	-	NR
-	-	-	-	-	COFFIN_SI	-	-	NR
-	-	-	-	-	-	T	-	NR
-	-	-	-	-	MENTAL_R	-	-	NR
-	-	-	-	0	-	T	1.4487529	NR
-	-	-	-	-	-	C	-	NR
-	-	-	-	-	-	C	-	NR
-	-	-	-	0	-	C	0.3118358	NR
-	-	-	-	-	-	C	-	NR
-	-	-	-	0	-	C	0.7632342	NR
-	-	-	-	-	-	G	-	NR
-	-	-	-	0	-	G	-	NR

-	-	-	-	0	-	T	0.5470817!	NR
-	-	-	-	-	-	G	-	NR
-	-	-	-	0	-	G	0.1943807	NR
-	-	-	-	0	-	G	0.6304281	NR
-	-	-	-	-	Emery-Drei	C	-	NR
-	-	-	-	2	-	C	0.4273184	NR
-	-	-	-	-	-	C	-	NR
-	-	-	-	2	-	A	0.2046020	NR
-	-	-	-	0	-	T	0.1034632!	NR
-	-	-	-	0	-	C	0.2396578	NR
-	-	-	-	-	-	A	-	NR
-	-	-	-	0	-	G	0.7347409	NR
-	-	-	-	0	-	c	0.5276987	NR
-	-	-	-	0	-	G	0.2871464	NR
-	-	-	-	0	Cleft_palat	G	0.1694729	NR
-	-	-	-	-	-	-	-	NR
-	-	-	-	-	-	GAGG	-	NR
-	-	-	-	0	DIABETES_	G	-	NR
-	-	-	-	0	-	c	2.5626962	NR
-	-	-	-	0	-	A	0.1081536	NR
-	-	-	-	-	-	C	-	NR
-	-	-	-	0	-	T	1.2535165	NR
-	-	-	-	0	-	A	0.0702743	NR
-	-	-	-	-	MYELODYS	T	-	NR
-	-	-	-	0	-	C	0.8869098	NR
-	-	-	-	2	-	G	1.2511757!	NR
-	-	-	-	-	-	-	-	NR
-	-	-	-	0	-	A	0.3481558!	NR
-	-	-	-	-	-	A	-	NR
-	-	-	-	-	THYROID_(	-	-	NR
-	-	-	-	-	THYROID_(	G	-	NR
-	-	-	-	-	-	C	-	NR
-	-	-	-	-	Achondrog	-	-	NR
-	-	-	-	-	-	a	-	NR
-	-	-	-	0	-	c	0.2470957!	NR
-	-	-	-	-	-	A	-	NR
-	-	-	-	2	-	A	0.8519863	NR
-	-	-	-	2	-	C	-	NR
-	-	-	-	0	-	C	2.0305426	NR
-	-	-	-	-	-	C	-	NR
-	-	-	-	-	LYMPHANG	-	-	NR
-	-	-	-	-	-	T	-	NR
-	-	-	-	0	-	T	0.7996643	NR
-	-	-	-	-	-	G	-	NR
-	-	-	-	0	-	C	1.6118683!	NR
-	-	-	-	0	-	T	0.9600627	NR
-	-	-	-	-	-	T	-	NR

-	-	-	-	0	-	T	1.1360345[illegible]	NR
-	-	-	-	-	-	T	-	NR
-	-	-	-	-	-	G	-	NR
-	-	-	-	-	-	A	-	NR
-	-	-	-	0	-	C	-	NR
-	-	-	-	-	-	T	-	NR
-	-	-	-	-	-	C	-	NR
-	-	-	-	-	Wolf-Hirscl	AC	-	NR
-	-	-	-	-	-	C	-	NR
-	-	-	-	-	Colorectal_	T	-	NR
-	-	-	-	-	-	A	-	NR
-	-	-	-	-	-	T	-	NR
-	-	-	-	0	WERNER_S	g	0.1420064[illegible]	NR
-	-	-	-	-	-	C	-	NR
-	-	-	-	-	-	T	-	NR

all_pathoge	pathogenic	large_intes	all_somatic	Somatic	SNP
NR	none	NR	NR	none	chr1:27105931
NR	none	NR	NR	none	chr3:181430901
NR	none	NR	NR	none	chr5:112175211
NR	none	NR	NR	none	chr5:112175603
NR	none	NR	NR	none	chr5:112176559
NR	none	NR	NR	none	chr7:128843396
NR	none	NR	NR	none	chr1:11181327
NR	none	NR	NR	none	chr1:11313993
NR	none	NR	NR	none	chr1:144918984
NR	none	NR	NR	none	chr1:147091495
NR	none	NR	NR	none	chr1:162740264
NR	none	NR	NR	none	chr1:162743362
NR	none	NR	NR	none	chr1:193111048
NR	none	NR	NR	none	chr1:204433727
NR	none	NR	NR	none	chr1:226552729
NR	none	NR	NR	none	chr1:27106648
NR	none	NR	NR	none	chr1:45798440
NR	none	NR	NR	none	chr10:114903764
NR	none	NR	NR	none	chr10:114910882
NR	none	NR	NR	none	chr10:123260408
NR	none	NR	NR	none	chr10:123325158
NR	none	NR	NR	none	chr10:22021981
NR	none	NR	NR	none	chr10:70432654
NR	none	NR	NR	none	chr10:88676945
NR	none	NR	NR	none	chr10:89624245
NR	none	NR	NR	none	chr10:89692928
NR	none	NR	NR	none	chr10:89711992
NR	none	NR	NR	none	chr11:108175462
NR	none	NR	NR	none	chr11:108175463
NR	none	NR	NR	none	chr11:108216568
NR	none	NR	NR	none	chr11:4148290
NR	none	NR	NR	none	chr12:121416650
NR	none	NR	NR	none	chr12:121435342
NR	none	NR	NR	none	chr12:121435427
NR	none	NR	NR	none	chr12:132502873
NR	none	NR	NR	none	chr12:25362777
NR	none	NR	NR	none	chr12:46230604
NR	none	NR	NR	none	chr13:28979994
NR	none	NR	NR	none	chr14:51204959
NR	none	NR	NR	none	chr14:81609692
NR	none	NR	NR	none	chr14:95595941
NR	none	NR	NR	none	chr15:99250839
NR	none	NR	NR	none	chr16:3788618
NR	none	NR	NR	none	chr17:12016675
NR	none	NR	NR	none	chr17:37665992

NR	none	NR	NR	none	chr17:37880988
NR	none	NR	NR	none	chr17:37881000
NR	none	NR	NR	none	chr17:37884037
NR	none	NR	NR	none	chr17:48273335
NR	none	NR	NR	none	chr17:7574003
NR	none	NR	NR	none	chr17:7574012
NR	none	NR	NR	none	chr17:7574035
NR	none	NR	NR	none	chr17:7576851
NR	none	NR	NR	none	chr17:7577082
NR	none	NR	NR	none	chr17:7577121
NR	none	NR	NR	none	chr17:7577498
NR	none	NR	NR	none	chr17:7577538
NR	none	NR	NR	none	chr17:7577539
NR	none	NR	NR	none	chr17:7577548
NR	none	NR	NR	none	chr17:7577556
NR	none	NR	NR	none	chr17:7577581
NR	none	NR	NR	none	chr17:7578263
NR	none	NR	NR	none	chr17:7578395
NR	none	NR	NR	none	chr17:7578404
NR	none	NR	NR	none	chr17:7578406
NR	none	NR	NR	none	chr18:45374881
NR	none	NR	NR	none	chr18:47800596
NR	none	NR	NR	none	chr18:48591919
NR	none	NR	NR	none	chr18:48593405
NR	none	NR	NR	none	chr18:48604701
NR	none	NR	NR	none	chr19:11101993
NR	none	NR	NR	none	chr19:45858976
NR	none	NR	NR	none	chr2:141707868
NR	none	NR	NR	none	chr2:178095644
NR	none	NR	NR	none	chr2:209108311
NR	none	NR	NR	none	chr2:212570063
NR	none	NR	NR	none	chr2:212812179
NR	none	NR	NR	none	chr2:216243903
NR	none	NR	NR	none	chr2:47643551
NR	none	NR	NR	none	chr20:57484421
NR	none	NR	NR	none	chr22:41548008
NR	none	NR	NR	none	chr3:128202753
NR	none	NR	NR	none	chr3:128204951
NR	none	NR	NR	none	chr3:134880871
NR	none	NR	NR	none	chr3:142222284
NR	none	NR	NR	none	chr3:142281298
NR	none	NR	NR	none	chr3:178916876
NR	none	NR	NR	none	chr3:178916924
NR	none	NR	NR	none	chr3:30732970
NR	none	NR	NR	none	chr3:37053568
NR	none	NR	NR	none	chr3:41277275
NR	none	NR	NR	none	chr3:69928509

NR	none	NR	NR	none	chr4:153249510
NR	none	NR	NR	none	chr4:153250883
NR	none	NR	NR	none	chr4:1807478
NR	none	NR	NR	none	chr4:1808286
NR	none	NR	NR	none	chr4:55152040
NR	none	NR	NR	none	chr4:55593464
NR	none	NR	NR	none	chr4:55599268
NR	none	NR	NR	none	chr4:55979558
NR	none	NR	NR	none	chr5:112102978
NR	none	NR	NR	none	chr5:112151204
NR	none	NR	NR	none	chr5:112154969
NR	none	NR	NR	none	chr5:112162854
NR	none	NR	NR	none	chr5:112162891
NR	none	NR	NR	none	chr5:112164616
NR	none	NR	NR	none	chr5:112173917
NR	none	NR	NR	none	chr5:112173953
NR	none	NR	NR	none	chr5:112175216
NR	none	NR	NR	none	chr5:112175322
NR	none	NR	NR	none	chr5:112175390
NR	none	NR	NR	none	chr5:112175639
NR	none	NR	NR	none	chr6:134491471
NR	none	NR	NR	none	chr6:152510429
NR	none	NR	NR	none	chr6:152706918
NR	none	NR	NR	none	chr6:18236780
NR	none	NR	NR	none	chr7:106509723
NR	none	NR	NR	none	chr7:140481411
NR	none	NR	NR	none	chr7:148525904
NR	none	NR	NR	none	chr7:2979448
NR	none	NR	NR	none	chr7:55214348
NR	none	NR	NR	none	chr7:55268916
NR	none	NR	NR	none	chr7:6043355
NR	none	NR	NR	none	chr8:103357685
NR	none	NR	NR	none	chr9:135985831
NR	none	NR	NR	none	chr9:8485834
NR	none	NR	NR	none	chr9:93640009
NR	none	NR	NR	none	chr9:98211572
NR	none	NR	NR	none	chrX:41075279
NR	none	NR	NR	none	chrX:44969370
NR	none	NR	NR	none	chrX:44970644
NR	none	NR	NR	none	chr1:11168337
NR	none	NR	NR	none	chr1:120478125
NR	none	NR	NR	none	chr1:120539904
NR	none	NR	NR	none	chr1:144854558
NR	none	NR	NR	none	chr1:144859889
NR	none	NR	NR	none	chr1:204397289
NR	none	NR	NR	none	chr1:204426879
NR	none	NR	NR	none	chr1:220825377

NR	none	NR	NR	none		chr1:220835212
NR	none	NR	NR	none		chr1:27105838
NR	none	NR	NR	none		chr1:65303659
NR	none	NR	NR	none		chr1:65311214
NR	none	NR	NR	none		chr1:65321250
NR	none	NR	NR	none		chr1:9777666
NR	none	NR	NR	none		chr10:104357020
NR	none	NR	NR	none		chr10:43606856
NR	none	NR	NR	none		chr10:43613843
NR	none	NR	NR	none		chr11:108123551
NR	none	NR	NR	none		chr11:108138003
NR	none	NR	NR	none		chr11:108143456
NR	none	NR	NR	none		chr11:32417842
NR	none	NR	NR	none		chr11:44129546
NR	none	NR	NR	none		chr11:64577147
NR	none	NR	NR	none		chr12:121416864
NR	none	NR	NR	none		chr12:121432117
NR	none	NR	NR	none		chr12:43770052
NR	none	NR	NR	none		chr12:43825284
NR	none	NR	NR	none		chr12:56494998
NR	none	NR	NR	none		chr12:56495016
NR	none	NR	NR	none		chr13:103528002
NR	none	NR	NR	none		chr14:105239894
NR	none	NR	NR	none		chr14:62194239
NR	none	NR	NR	none		chr14:62207327
NR	none	NR	NR	none		chr14:62207557
NR	none	NR	NR	none		chr14:92441008
NR	none	NR	NR	none		chr14:92461859
NR	none	NR	NR	none		chr14:95590698
NR	none	NR	NR	none		chr15:66729108
NR	none	NR	NR	none		chr15:99442820
NR	none	NR	NR	none		chr16:15814883
NR	none	NR	NR	none		chr16:2124250
NR	none	NR	NR	none		chr16:2133701
NR	none	NR	NR	none		chr16:3778882
NR	none	NR	NR	none		chr16:3795292
NR	none	NR	NR	none		chr17:29509641
NR	none	NR	NR	none		chr17:29677228
NR	none	NR	NR	none		chr17:37650845
NR	none	NR	NR	none		chr17:48274561
NR	none	NR	NR	none		chr17:66511578
NR	none	NR	NR	none		chr17:78355494
NR	none	NR	NR	none		chr17:8110130
NR	none	NR	NR	none		chr18:25568610
NR	none	NR	NR	none		chr18:48591923
NR	none	NR	NR	none		chr18:50832036
NR	none	NR	NR	none		chr19:11123688

NR	none	NR	NR	none	chr19:1207176
NR	none	NR	NR	none	chr19:1221293
NR	none	NR	NR	none	chr19:45860637
NR	none	NR	NR	none	chr2:141092084
NR	none	NR	NR	none	chr2:141116447
NR	none	NR	NR	none	chr2:141201978
NR	none	NR	NR	none	chr2:141232800
NR	none	NR	NR	none	chr2:141245207
NR	none	NR	NR	none	chr2:141274576
NR	none	NR	NR	none	chr2:141625410
NR	none	NR	NR	none	chr2:141773397
NR	none	NR	NR	none	chr2:142567910
NR	none	NR	NR	none	chr2:209108317
NR	none	NR	NR	none	chr2:209113192
NR	none	NR	NR	none	chr2:219545345
NR	none	NR	NR	none	chr2:29416481
NR	none	NR	NR	none	chr2:29940529
NR	none	NR	NR	none	chr2:47637507
NR	none	NR	NR	none	chr2:47698108
NR	none	NR	NR	none	chr2:60773146
NR	none	NR	NR	none	chr2:61717816
NR	none	NR	NR	none	chr20:39788754
NR	none	NR	NR	none	chr20:39792063
NR	none	NR	NR	none	chr20:57474021
NR	none	NR	NR	none	chr20:57485812
NR	none	NR	NR	none	chr22:36684816
NR	none	NR	NR	none	chr22:41513774
NR	none	NR	NR	none	chr22:41547910
NR	none	NR	NR	none	chr22:41574892
NR	none	NR	NR	none	chr3:12626080
NR	none	NR	NR	none	chr3:142242985
NR	none	NR	NR	none	chr3:142281353
NR	none	NR	NR	none	chr3:178916917
NR	none	NR	NR	none	chr3:187440264
NR	none	NR	NR	none	chr3:187440364
NR	none	NR	NR	none	chr3:187443314
NR	none	NR	NR	none	chr3:188327409
NR	none	NR	NR	none	chr3:195594858
NR	none	NR	NR	none	chr3:37090407
NR	none	NR	NR	none	chr3:41275179
NR	none	NR	NR	none	chr3:47059214
NR	none	NR	NR	none	chr3:47162886
NR	none	NR	NR	none	chr3:52440855
NR	none	NR	NR	none	chr3:52613209
NR	none	NR	NR	none	chr3:89390150
NR	none	NR	NR	none	chr3:89391112
NR	none	NR	NR	none	chr3:89521664

NR	none	NR	NR	none		chr4:106155199
NR	none	NR	NR	none		chr4:106156187
NR	none	NR	NR	none		chr4:106157698
NR	none	NR	NR	none		chr4:106164916
NR	none	NR	NR	none		chr4:106196829
NR	none	NR	NR	none		chr4:55593481
NR	none	NR	NR	none		chr4:55602724
NR	none	NR	NR	none		chr4:55960993
NR	none	NR	NR	none		chr4:87968524
NR	none	NR	NR	none		chr5:131927610
NR	none	NR	NR	none		chr5:180043918
NR	none	NR	NR	none		chr5:38489330
NR	none	NR	NR	none		chr5:67575548
NR	none	NR	NR	none		chr5:67588148
NR	none	NR	NR	none		chr5:7889295
NR	none	NR	NR	none		chr6:106547325
NR	none	NR	NR	none		chr6:106547372
NR	none	NR	NR	none		chr6:117724379
NR	none	NR	NR	none		chr6:135518349
NR	none	NR	NR	none		chr6:138196066
NR	none	NR	NR	none		chr6:152527386
NR	none	NR	NR	none		chr6:152712428
NR	none	NR	NR	none		chr6:152712559
NR	none	NR	NR	none		chr6:152841662
NR	none	NR	NR	none		chr6:160493913
NR	none	NR	NR	none		chr6:395895
NR	none	NR	NR	none		chr6:51497503
NR	none	NR	NR	none		chr6:51609303
NR	none	NR	NR	none		chr6:51889474
NR	none	NR	NR	none		chr6:51907698
NR	none	NR	NR	none		chr6:51907900
NR	none	NR	NR	none		chr7:100411556
NR	none	NR	NR	none		chr7:100417397
NR	none	NR	NR	none		chr7:106508539
NR	none	NR	NR	none		chr7:106508919
NR	none	NR	NR	none		chr7:106509331
NR	none	NR	NR	none		chr7:116340262
NR	none	NR	NR	none		chr7:126173716
NR	none	NR	NR	none		chr7:2959172
NR	none	NR	NR	none		chr7:2979559
NR	none	NR	NR	none		chr7:55249063
NR	none	NR	NR	none		chr7:55270249
NR	none	NR	NR	none		chr8:103266602
NR	none	NR	NR	none		chr8:103326128
NR	none	NR	NR	none		chr8:113364696
NR	none	NR	NR	none		chr8:113841946
NR	none	NR	NR	none		chr8:114111084

NR	none	NR	NR	none		chr8:114186003
NR	none	NR	NR	none		chr8:118817119
NR	none	NR	NR	none		chr8:118834761
NR	none	NR	NR	none		chr8:38282202
NR	none	NR	NR	none		chr8:48866189
NR	none	NR	NR	none		chr8:57079634
NR	none	NR	NR	none		chr8:71050488
NR	none	NR	NR	none		chr9:120475547
NR	none	NR	NR	none		chr9:135787796
NR	none	NR	NR	none		chr9:135985796
NR	none	NR	NR	none		chr9:35076755
NR	none	NR	NR	none		chr9:37006494
NR	none	NR	NR	none		chr9:8518052
NR	none	NR	NR	none		chr9:93650096
NR	none	NR	NR	none		chr9:97888854
NR	none	NR	NR	none		chr9:98231085
NR	none	NR	NR	none		chr9:98231110
NR	none	NR	NR	none		chr9:98240410
NR	none	NR	NR	none		chrX:44938563
NR	none	NR	NR	none		
NR	none	NR	NR	none		
NR	none	NR	NR	none		
NR	none	NR	NR	none		
NR	none	NR	NR	none		
NR	none	NR	NR	none		
NR	none	NR	NR	none		
NR	none	NR	NR	none		
NR	none	NR	NR	none		
NR	none	NR	NR	none		
NR	none	NR	NR	none		
NR	none	NR	NR	none		
NR	none	NR	NR	none		
NR	none	NR	NR	none		
NR	none	NR	NR	none		
NR	none	NR	NR	none		
NR	none	NR	NR	none		
NR	none	NR	NR	none		
NR	none	NR	NR	none		
NR	none	NR	NR	none		
NR	none	NR	NR	none		
NR	none	NR	NR	none		
NR	none	NR	NR	none		
NR	none	NR	NR	none		
NR	none	NR	NR	none		
NR	none	NR	NR	none		
NR	none	NR	NR	none		
NR	none	NR	NR	none		

NR	none	NR	NR	none
NR	none	NR	NR	none
NR	none	NR	NR	none
NR	none	NR	NR	none
NR	none	NR	NR	none
NR	none	NR	NR	none
NR	none	NR	NR	none
NR	none	NR	NR	none
NR	none	NR	NR	none
NR	none	NR	NR	none
NR	none	NR	NR	none
NR	none	NR	NR	none
NR	none	NR	NR	none
NR	none	NR	NR	none
NR	none	NR	NR	none
NR	none	NR	NR	none
NR	none	NR	NR	none
NR	none	NR	NR	none
NR	none	NR	NR	none
NR	none	NR	NR	none
NR	none	NR	NR	none
NR	none	NR	NR	none
NR	none	NR	NR	none
NR	none	NR	NR	none
NR	none	NR	NR	none
NR	none	NR	NR	none
NR	none	NR	NR	none
NR	none	NR	NR	none
NR	none	NR	NR	none
NR	none	NR	NR	none
NR	none	NR	NR	none
NR	none	NR	NR	none
NR	none	NR	NR	none
NR	none	NR	NR	none
NR	none	NR	NR	none
NR	none	NR	NR	none
NR	none	NR	NR	none
NR	none	NR	NR	none
NR	none	NR	NR	none
NR	none	NR	NR	none
NR	none	NR	NR	none
NR	none	NR	NR	none
NR	none	NR	NR	none
NR	none	NR	NR	none
NR	none	NR	NR	none
NR	none	NR	NR	none
NR	none	NR	NR	none

NR	none	NR	NR	none
NR	none	NR	NR	none
NR	none	NR	NR	none
NR	none	NR	NR	none
NR	none	NR	NR	none
NR	none	NR	NR	none
NR	none	NR	NR	none
NR	none	NR	NR	none
NR	none	NR	NR	none
NR	none	NR	NR	none
NR	none	NR	NR	none
NR	none	NR	NR	none
NR	none	NR	NR	none
NR	none	NR	NR	none
NR	none	NR	NR	none
NR	none	NR	NR	none
NR	none	NR	NR	none
NR	none	NR	NR	none
NR	none	NR	NR	none
NR	none	NR	NR	none
NR	none	NR	NR	none
NR	none	NR	NR	none
NR	none	NR	NR	none
NR	none	NR	NR	none
NR	none	NR	NR	none
NR	none	NR	NR	none
NR	none	NR	NR	none
NR	none	NR	NR	none
NR	none	NR	NR	none
NR	none	NR	NR	none
NR	none	NR	NR	none
NR	none	NR	NR	none
NR	none	NR	NR	none
NR	none	NR	NR	none
NR	none	NR	NR	none
NR	none	NR	NR	none
NR	none	NR	NR	none
NR	none	NR	NR	none
NR	none	NR	NR	none
NR	none	NR	NR	none
NR	none	NR	NR	none
NR	none	NR	NR	none
NR	none	NR	NR	none
NR	none	NR	NR	none
NR	none	NR	NR	none
NR	none	NR	NR	none
NR	none	NR	NR	none

NR	none	NR	NR	none
NR	none	NR	NR	none
NR	none	NR	NR	none
NR	none	NR	NR	none
NR	none	NR	NR	none
NR	none	NR	NR	none
NR	none	NR	NR	none
NR	none	NR	NR	none
NR	none	NR	NR	none
NR	none	NR	NR	none
NR	none	NR	NR	none
NR	none	NR	NR	none
NR	none	NR	NR	none
NR	none	NR	NR	none
NR	none	NR	NR	none
NR	none	NR	NR	none
NR	none	NR	NR	none
NR	none	NR	NR	none
NR	none	NR	NR	none
NR	none	NR	NR	none
NR	none	NR	NR	none
NR	none	NR	NR	none
NR	none	NR	NR	none
NR	none	NR	NR	none
NR	none	NR	NR	none
NR	none	NR	NR	none
NR	none	NR	NR	none
NR	none	NR	NR	none
NR	none	NR	NR	none
NR	none	NR	NR	none
NR	none	NR	NR	none
NR	none	NR	NR	none
NR	none	NR	NR	none
NR	none	NR	NR	none
NR	none	NR	NR	none
NR	none	NR	NR	none
NR	none	NR	NR	none
NR	none	NR	NR	none
NR	none	NR	NR	none
NR	none	NR	NR	none
NR	none	NR	NR	none
NR	none	NR	NR	none
NR	none	NR	NR	none
NR	none	NR	NR	none
NR	none	NR	NR	none
NR	none	NR	NR	none
NR	none	NR	NR	none

NR	none	NR	NR	none
NR	none	NR	NR	none
NR	none	NR	NR	none
NR	none	NR	NR	none
NR	none	NR	NR	none
NR	none	NR	NR	none
NR	none	NR	NR	none
NR	none	NR	NR	none
NR	none	NR	NR	none
NR	none	NR	NR	none
NR	none	NR	NR	none
NR	none	NR	NR	none
NR	none	NR	NR	none
NR	none	NR	NR	none
NR	none	NR	NR	none
