# Supplementary material for: Identification of Novel Mutations in Colorectal Cancer Patients Using AmpliSeq Comprehensive Cancer Panel
Source: J Pers Med. 2021 Jun 9;11(6):535. doi: 10.3390/jpm11060535 (PMC8230213; doi:10.3390/jpm11060535)
Supplement: Supplementary file 1 [file jpm-11-00535-s001.zip › jpm-1202870-supplementary/supplementary tables/Supplementary Table S3.pdf]

Supplementary Table S3

Gene ID_CI	Variant	Max of VAF	Min of VAF	Average of VAF
KDR	chr4:55953747	100	72.7	96.93333333
FBN3	chr19:8146019	100	99	99.88571429
COL1A1	chr17:48277061	100	10.4	82.30967742
PDE4DIP	chr1:144886092	58.3	27.5	47.34137931
ZNF384	chr12:6777111	100	96.5	99.51111111
SMARCB1	chr22:24145672	100	97.8	99.8
AR	chrX:66943601	100	99.2	99.86923077
ARID1A	chr1:27099010	100	100	100
PDE4DIP	chr1:144930571	58.1	31.1	47.59885057
MLL3	chr7:151879244	100	100	100
PDE4DIP	chr1:144852315	100	32	65.34615385
TSC2	chr16:2129453	100	96.2	99.57777778
WHSC1	chr4:1976481	100	95.7	99.01818182
WHSC1	chr4:1976483	100	95.7	98.98181818
EP300	chr22:41542838	100	55.8	88
GREB1	chr2:11751005	100	92	97.87777778
FLT3	chr13:28597449	100	32.4	60.40769231
LRP1B	chr2:141202026	99.7	96.9	98.68571429
ITGB2	chr21:46327067	100	99.6	99.9
PKHD1	chr6:51921815	100	90	97.22
PTPRD	chr9:8460647	100	82.5	94.825
GATA2	chr3:128202760	99.9	25.6	66.53478261
PDGFRA	chr4:55129894	99.7	99.5	99.6
KDM6A	chrX:44942869	100	49.6	74.8
ITGA10	chr1:145530939	64.1	28.8	46.9
ITGA10	chr1:145533894	51	38.4	43.42
MUC1	chr1:155160052	99.7	98.6	99
NTRK1	chr1:156811985	58.2	50.7	54.45
DDR2	chr1:162737153	99.5	20.9	38.1
RNF2	chr1:185056670	54.1	40.3	48.16666667
IKBKE	chr1:206647652	50.4	50.4	50.4
ARID1A	chr1:27097699	99	29.7	55.475
BLNK	chr10:97975168	91.2	8.8	58.89333333
EP400	chr12:132562069	100	100	100
KRAS	chr12:25398279	50.5	0	2.048
ZNF384	chr12:6788242	49.4	49.4	49.4
NIN	chr14:51221316	99.6	98.9	99.25
TRIP11	chr14:92454746	53.6	50.7	52.5
THBS1	chr15:39884882	83.3	30.4	55.33829787
CDH11	chr16:65026991	49.9	49.9	49.9
BRIP1	chr17:59760648	50.2	50.2	50.2
PRKAR1A	chr17:66511530	100	100	100
TP53	chr17:7579634	57	20.6	38.21428571
RNF213	chr17:78357518	49.5	49.5	49.5
PER1	chr17:8045566	50.8	50.8	50.8

FZR1	chr19:3531825	50.5	50.5	50.5
LRP1B	chr2:141232924	49.5	49.5	49.5
LRP1B	chr2:141607906	50.8	50.8	50.8
SOX11	chr2:5833816	99.5	99.5	99.5
PTPRT	chr20:40743814	100	100	100
AURKA	chr20:54959349	50.6	50.6	50.6
ERG	chr21:39817470	99.7	99.7	99.7
FANCD2	chr3:10132031	49.2	29.3	39.22
EPHA3	chr3:89448462	49.5	49.5	49.5
NFKB1	chr4:103533242	49.8	49.8	49.8
TET2	chr4:106193885	67.4	46.2	54.7
PDGFRA	chr4:55129892	99.5	99.5	99.5
PDGFRB	chr5:149499530	60.3	11.9	29.90333333
IL6ST	chr5:55237596	50.2	48.4	49.3
IL6ST	chr5:55247262	50.4	50.4	50.4
SYNE1	chr6:152529254	49.9	49.9	49.9
DST	chr6:56485091	99.3	96.8	98.05
MLL3	chr7:151902163	100	16	55.76666667
TRRAP	chr7:98543325	50.3	50.3	50.3
UBR5	chr8:103341456	77.5	49.5	63.76666667
MYC	chr8:128753001	99.7	99.7	99.7
NCOA2	chr8:71128917	49	49	49
NUP214	chr9:134090676	99	99	99
PAK3	chrX:110463549	50.9	50.9	50.9
MTOR	chr1:11167502	24.7	24.7	24.7
TRIM33	chr1:114940315	28.2	28.2	28.2
TRIM33	chr1:114976234	46.3	46.3	46.3
NOTCH2	chr1:120477935	22.2	22.2	22.2
NOTCH2	chr1:120539655	48.8	48.8	48.8
PDE4DIP	chr1:144859929	27	22	24.18571429
PDE4DIP	chr1:144863360	39.3	21.2	28.1
PDE4DIP	chr1:144863366	25.1	25.1	25.1
PDE4DIP	chr1:144863384	25.2	25.2	25.2
PDE4DIP	chr1:144863388	25.1	25.1	25.1
PDE4DIP	chr1:144866660	23.9	23.9	23.9
PDE4DIP	chr1:144866673	27.6	24.1	25.85
PDE4DIP	chr1:144879054	43.1	15.3	26.36666667
PDE4DIP	chr1:144879086	47.7	47.7	47.7
PDE4DIP	chr1:144881463	29.2	24.3	26.86
PDE4DIP	chr1:144881547	27.5	18.7	23.7
PDE4DIP	chr1:144886267	37.2	23.3	27.075
PDE4DIP	chr1:144923712	35.2	35.2	35.2
PDE4DIP	chr1:144930977	28.7	25.7	27.125
PDE4DIP	chr1:144931423	20.6	20.6	20.6
PDE4DIP	chr1:144994617	46.5	20.9	30.53333333
PDE4DIP	chr1:144994670	29.4	19.5	24.8
PDE4DIP	chr1:144994694	14.4	14.4	14.4

ITGA10	chr1:145528319	37.6	37.6	37.6
ITGA10	chr1:145528348	74.6	54.7	64.65
BCL9	chr1:147090697	33.8	33.8	33.8
BCL9	chr1:147094034	21.4	21.4	21.4
MCL1	chr1:150550722	33	33	33
ARNT	chr1:150808978	48.6	48.6	48.6
MUC1	chr1:155160768	29.1	29.1	29.1
NTRK1	chr1:156841496	25	25	25
DDR2	chr1:162740326	15	11.6	13.35
DDR2	chr1:162748385	25.5	25.5	25.5
RNF2	chr1:185067185	45.3	45.3	45.3
TPR	chr1:186301473	51.9	51.9	51.9
TPR	chr1:186304551	27	27	27
TPR	chr1:186315243	16.2	9.9	12.06666667
PTGS2	chr1:186646996	27.5	27.5	27.5
PTGS2	chr1:186647541	26.2	26.2	26.2
PAX7	chr1:19018422	51.2	51.2	51.2
CDC73	chr1:193110968	54.2	54.2	54.2
CDC73	chr1:193111251	29.2	29.2	29.2
PIK3C2B	chr1:204438894	41.4	41.4	41.4
MARK1	chr1:220808683	47.4	44	45.7
MTR	chr1:236966821	55.2	37.4	48.45
MTR	chr1:237016242	30.5	30.5	30.5
MTR	chr1:237038012	17.8	15.1	16.45
MTR	chr1:237058719	27.3	27.3	27.3
AKT3	chr1:243727211	40.2	40.2	40.2
ARID1A	chr1:27092820	37.3	37.3	37.3
ARID1A	chr1:27092822	38.8	38.8	38.8
ARID1A	chr1:27106343	37.6	37.6	37.6
MYCL1	chr1:40363605	56.5	56.5	56.5
MPL	chr1:43803502	38.2	38.2	38.2
MPL	chr1:43818486	46.5	46.5	46.5
MUTYH	chr1:45799181	92.2	92.2	92.2
BCL10	chr1:85733370	20.8	20.8	20.8
BCL10	chr1:85736596	30.7	30.7	30.7
DPYD	chr1:97564040	26.1	26.1	26.1
DPYD	chr1:98144767	21.2	21.2	21.2
DPYD	chr1:98206046	21.8	21.8	21.8
DPYD	chr1:98386487	31.8	31.8	31.8
NFKB2	chr10:104156192	25.3	25.3	25.3
SUFU	chr10:104357022	46	33.9	39.95
TCF7L2	chr10:114710518	23.3	23.3	23.3
TCF7L2	chr10:114911539	20	20	20
FGFR2	chr10:123246886	21.2	21.2	21.2
MLLT10	chr10:21901248	86	82.3	83.975
MLLT10	chr10:21901253	17.7	13.5	15.52
MLLT10	chr10:22022026	25.8	25.8	25.8

KLF6	chr10:3824270	43.5	43.5	43.5
NCOA4	chr10:51584910	51.2	40.5	45.85
NCOA4	chr10:51586458	39.7	39.7	39.7
TET1	chr10:70332342	23.8	23.8	23.8
TET1	chr10:70406386	98.8	95.7	97.25
TET1	chr10:70451510	23.8	23.8	23.8
KAT6B	chr10:76735416	79.1	12.1	47.68571429
KAT6B	chr10:76736004	20.8	20.8	20.8
KAT6B	chr10:76744954	85.6	85.6	85.6
KAT6B	chr10:76744958	14.4	14.4	14.4
KAT6B	chr10:76744965	20.9	17	19.33333333
CYP2C19	chr10:96541627	46.2	46.2	46.2
BIRC3	chr11:102195293	38.2	38.2	38.2
BIRC3	chr11:102195716	21	21	21
BIRC3	chr11:102201890	33.1	33.1	33.1
BIRC3	chr11:102207645	62	55	58.5
BIRC3	chr11:102207653	35.3	35.3	35.3
GUCY1A2	chr11:106579288	27.1	27.1	27.1
ATM	chr11:108117897	16	12.5	14.25
ATM	chr11:108175596	23	23	23
ATM	chr11:108201010	25.8	25.8	25.8
MLL	chr11:118376178	24.8	24.8	24.8
MLL	chr11:118390491	37.5	37.5	37.5
CBL	chr11:119145464	78.7	38	64.3
NUP98	chr11:3794852	23.6	23.6	23.6
EXT2	chr11:44228576	59	59	59
MEN1	chr11:64572073	57.8	57.8	57.8
MRE11A	chr11:94224026	27.5	27.5	27.5
MAML2	chr11:95724845	71.2	71.2	71.2
MAML2	chr11:95826226	23.8	23.8	23.8
MAML2	chr11:95826390	24.8	24.8	24.8
PTPN11	chr12:112910806	47.2	32.7	41.525
EP400	chr12:132466141	18.5	18.5	18.5
EP400	chr12:132490800	40.1	40.1	40.1
EP400	chr12:132497625	24.5	24.5	24.5
EP400	chr12:132529321	51.2	51.2	51.2
EP400	chr12:132535176	22	22	22
ADAMTS20	chr12:43846296	22.4	22.4	22.4
ARID2	chr12:46285667	46.2	46.2	46.2
MLL2	chr12:49433170	77.6	77.6	77.6
ERBB3	chr12:56479046	21.2	21.2	21.2
CDK4	chr12:58143112	46.8	46.8	46.8
IRS2	chr13:110434840	46.9	46.9	46.9
FOXO1	chr13:41134840	20.8	20.8	20.8
HSP90AA1	chr14:102548088	26.5	26.5	26.5
HSP90AA1	chr14:102548797	72	72	72
NIN	chr14:51225320	68.3	68.3	68.3

TSHR	chr14:81554286	22	22	22
TSHR	chr14:81554303	34.8	22.9	28.85
TSHR	chr14:81606071	20.2	20.2	20.2
TRIP11	chr14:92461911	32.5	19.3	27.4
TRIP11	chr14:92471880	20.8	20.8	20.8
DICER1	chr14:95569893	39.6	39.6	39.6
CASC5	chr15:40913884	29.7	29.7	29.7
CASC5	chr15:40949531	20.8	8.2	14.5
MAP2K1	chr15:66782073	51.1	51.1	51.1
NTRK3	chr15:88669573	27.3	27.3	27.3
IDH2	chr15:90628243	28.3	28.3	28.3
BLM	chr15:91304145	46.3	39.4	42.85
BLM	chr15:91347493	27.8	27.8	27.8
IGF1R	chr15:99250742	23.5	23.5	23.5
IGF1R	chr15:99460151	35.8	35.8	35.8
MYH11	chr16:15808865	44.7	44.7	44.7
MYH11	chr16:15826573	38.2	38.2	38.2
CREBBP	chr16:3843610	44.5	44.5	44.5
CDH1	chr16:68844081	64.2	64.2	64.2
FANCA	chr16:89857743	31.6	31.6	31.6
FANCA	chr16:89877320	23.8	23.8	23.8
NF1	chr17:29527684	48.5	48.5	48.5
NF1	chr17:29563074	60	22.2	36.1
NF1	chr17:29684016	21.8	21.8	21.8
CDK12	chr17:37627782	65.7	32.8	49.25
ERBB2	chr17:37868327	34.7	34.7	34.7
ERBB2	chr17:37868741	58.1	58.1	58.1
ERBB2	chr17:37883680	31	31	31
RARA	chr17:38504723	25.8	25.8	25.8
ETV4	chr17:41610590	43.8	43.8	43.8
ITGB3	chr17:45360865	29.8	29.8	29.8
ITGB3	chr17:45361773	24.3	24.3	24.3
ITGB3	chr17:45377857	54.8	34.8	44.8
ITGB3	chr17:45377881	31.3	31.3	31.3
NLRP1	chr17:5487204	57.1	40.4	48.75
BRIP1	chr17:59858190	34.2	34.2	34.2
BRIP1	chr17:59861596	25.1	25.1	25.1
BIRC5	chr17:76210389	29	29	29
BIRC5	chr17:76219645	24.2	11.1	17.91764706
RNF213	chr17:78262057	32.8	32.8	32.8
RNF213	chr17:78264358	24.8	17.3	20
RNF213	chr17:78268717	41.5	41.5	41.5
RNF213	chr17:78319102	41.9	14.3	24.5
RNF213	chr17:78351613	34.2	20.1	26.23333333
AURKB	chr17:8109871	19	19	19
CDH2	chr18:25589869	45.8	45.8	45.8
CDH2	chr18:25593716	26	26	26

MBD1	chr18:47793930	43.4	22.1	32.86428571
MBD1	chr18:47801260	97.2	97.1	97.15
SMAD4	chr18:48575204	25.2	25.2	25.2
SMAD4	chr18:48593440	20.9	20.9	20.9
DCC	chr18:50451642	37.3	37.3	37.3
DCC	chr18:50451662	58.1	58.1	58.1
DCC	chr18:50942526	66.6	66.6	66.6
DCC	chr18:50976845	34.6	34.6	34.6
CDH20	chr18:59221876	30.7	11	22.26666667
KEAP1	chr19:10600522	51.4	51.4	51.4
SMARCA4	chr19:11170500	42	42	42
TCF3	chr19:1646325	46.8	28.9	37.85
AKT2	chr19:40741244	34.2	34.2	34.2
CIC	chr19:42791062	59.5	59.5	59.5
CIC	chr19:42798455	61.9	61.9	61.9
MARK4	chr19:45805895	48.4	48.4	48.4
AURKC	chr19:57744792	21.5	21.5	21.5
LRP1B	chr2:141079482	26	26	26
LRP1B	chr2:141214069	51.9	51.9	51.9
LRP1B	chr2:141299350	26	26	26
LRP1B	chr2:141598592	30.4	30.4	30.4
LRP1B	chr2:141680645	29.3	29.3	29.3
LRP1B	chr2:141819743	30.5	30.5	30.5
LRP1B	chr2:141986696	56.1	56.1	56.1
LRP1B	chr2:142004935	24.5	24.5	24.5
LRP1B	chr2:142888193	21.4	21.4	21.4
ACVR2A	chr2:148672817	22.3	22.3	22.3
MYCN	chr2:16086048	29.2	29.2	29.2
NFE2L2	chr2:178096253	21.6	21.6	21.6
PMS1	chr2:190719333	36.8	36.8	36.8
PMS1	chr2:190719788	48.7	48.7	48.7
SF3B1	chr2:198262720	52.5	52.5	52.5
ERBB4	chr2:212248634	24.4	24.4	24.4
ERBB4	chr2:212495163	23.8	23.8	23.8
ERBB4	chr2:212989451	25.8	25.8	25.8
FN1	chr2:216232553	25.1	25.1	25.1
FN1	chr2:216237084	51.8	51.8	51.8
FN1	chr2:216272993	23.8	23.8	23.8
STK36	chr2:219544800	35.1	35.1	35.1
DNMT3A	chr2:25457148	31.9	31.9	31.9
DNMT3A	chr2:25523013	39	39	39
ALK	chr2:29498305	41.9	41.9	41.9
MSH2	chr2:47639586	30.8	30.8	30.8
MSH2	chr2:47641570	30.4	15	20.37142857
MSH2	chr2:47643502	95	23.8	52.6
MSH2	chr2:47705460	53.2	53.2	53.2
BCL11A	chr2:60773062	28.7	28.7	28.7

REL	chr2:61147771	87.3	83.8	85.61428571
REL	chr2:61147775	16.2	12.7	14.38571429
TCF7L1	chr2:85534779	11.9	11.9	11.9
SRC	chr20:36031361	38.8	38.8	38.8
SRC	chr20:36031767	67.4	23.1	48.5
GNAS	chr20:57428437	44.9	16.5	35.87419355
SMARCB1	chr22:24167632	37.5	17.8	24.74
MYH9	chr22:36690119	15.5	15.5	15.5
EP300	chr22:41550944	23.6	16.9	20.25
CYP2D6	chr22:42524893	21	21	21
FANCD2	chr3:10088295	30	30	30
RAF1	chr3:12626400	57.7	57.7	57.7
RAF1	chr3:12626737	51.1	51.1	51.1
RAF1	chr3:12641349	67.6	51.7	59.65
EPHB1	chr3:134885838	29.6	29.6	29.6
PIK3CB	chr3:138461524	44.2	44.2	44.2
ATR	chr3:142176618	47	47	47
ATR	chr3:142176649	27.5	27.5	27.5
ATR	chr3:142186845	37.3	37.3	37.3
ATR	chr3:142269112	20.4	20.4	20.4
ATR	chr3:142272647	28.7	19.8	25.175
ATR	chr3:142274652	25.6	25.6	25.6
PIK3CA	chr3:178916986	27.6	27.6	27.6
PIK3CA	chr3:178922273	57.8	53.5	55.65
SOX2	chr3:181430876	35	35	35
BCL6	chr3:187447019	25.9	25.9	25.9
CRBN	chr3:3194271	25.3	25.3	25.3
CRBN	chr3:3197988	45.2	45.2	45.2
MLH1	chr3:37090047	24.2	24.2	24.2
ITGA9	chr3:37567515	29.1	29.1	29.1
ITGA9	chr3:37791907	31.8	31.8	31.8
CTNNB1	chr3:41267196	23.8	23.8	23.8
LTF	chr3:46501310	31.1	31.1	31.1
SETD2	chr3:47155487	23	23	23
SETD2	chr3:47163994	25.8	25.8	25.8
PBRM1	chr3:52610602	22.2	22.2	22.2
PBRM1	chr3:52610619	42.8	42.8	42.8
MAGI1	chr3:65376926	30.8	30.8	30.8
MITF	chr3:69985829	47.1	47.1	47.1
MITF	chr3:70000910	25	25	25
FOXP1	chr3:71179709	62.5	25.8	44.53333333
EPHA3	chr3:89462276	20	20	20
EPHA3	chr3:89462294	23.3	23.3	23.3
NFKB1	chr4:103533291	29	29	29
NFKB1	chr4:103533656	29.4	29.4	29.4
NFKB1	chr4:103537741	22	22	22
TET2	chr4:106158026	45.9	45.9	45.9

FBXW7	chr4:153244077	29.2	29.2	29.2
FGFR3	chr4:1803326	47.8	47.8	47.8
FGFR3	chr4:1807545	30.1	11.6	19.63333333
WHSC1	chr4:1961342	30.7	30.7	30.7
WHSC1	chr4:1976485	89.2	89.2	89.2
WHSC1	chr4:1976486	10.5	10.5	10.5
KDR	chr4:55970908	36	36	36
KDR	chr4:55980293	23.5	23.5	23.5
KDR	chr4:55981509	47.7	47.7	47.7
LPHN3	chr4:62599099	20	20	20
LPHN3	chr4:62897317	25.2	25.2	25.2
AFF1	chr4:87967279	25.5	25.5	25.5
AFF1	chr4:87967936	32.6	32.6	32.6
APC	chr5:112164622	22.2	22.2	22.2
APC	chr5:112164623	69.1	69.1	69.1
APC	chr5:112173971	19	19	19
APC	chr5:112175208	29.3	29.3	29.3
APC	chr5:112175611	61.3	61.3	61.3
RAD50	chr5:131924445	24.8	24.8	24.8
CTNNA1	chr5:138266523	45.5	45.5	45.5
CTNNA1	chr5:138269717	42	42	42
PDGFRB	chr5:149514424	30.3	30.3	30.3
FGFR4	chr5:176520463	36.8	36.8	36.8
NSD1	chr5:176637204	56.4	45.5	50.95
FLT4	chr5:180035995	47.6	47.6	47.6
SDHA	chr5:233504	23.1	14.5	18
LIFR	chr5:38481879	23.5	23.5	23.5
LIFR	chr5:38486091	32.2	32.2	32.2
LIFR	chr5:38499715	23.5	23.5	23.5
LIFR	chr5:38502822	46.4	46.4	46.4
IL6ST	chr5:55237092	27.6	27.6	27.6
IL6ST	chr5:55256341	23.6	23.6	23.6
IL6ST	chr5:55264283	29	29	29
PIK3R1	chr5:67522726	26.8	26.8	26.8
PIK3R1	chr5:67590496	41.2	41.2	41.2
FOXO3	chr6:108985258	21.4	21.4	21.4
ROS1	chr6:117710658	28.3	28.3	28.3
SGK1	chr6:134498787	32.2	32.2	32.2
SGK1	chr6:134528524	51.7	51.7	51.7
SYNE1	chr6:152457915	48.2	48.2	48.2
SYNE1	chr6:152470784	25	25	25
SYNE1	chr6:152472812	51.5	40.8	46.15
SYNE1	chr6:152542688	27	27	27
SYNE1	chr6:152631545	23.8	23.8	23.8
SYNE1	chr6:152631970	35.2	35.2	35.2
SYNE1	chr6:152639288	26.8	26.8	26.8
SYNE1	chr6:152644654	19.3	19.3	19.3

SYNE1	chr6:152652197	23.5	23.5	23.5
SYNE1	chr6:152674791	22.1	22.1	22.1
SYNE1	chr6:152712677	27.3	27.3	27.3
SYNE1	chr6:152823897	24	24	24
IGF2R	chr6:160412166	37	16.7	21.56363636
IGF2R	chr6:160491086	34.5	34.5	34.5
RPS6KA2	chr6:166952128	31.5	28.1	29.8
NOTCH4	chr6:32166734	56.4	56.4	56.4
IRF4	chr6:397155	33.2	33.2	33.2
FOXP4	chr6:41559050	27	27	27
PKHD1	chr6:51524653	21	21	21
PKHD1	chr6:51613058	34.1	34.1	34.1
PKHD1	chr6:51889459	51.9	51.9	51.9
PKHD1	chr6:51890498	29.5	29.5	29.5
PKHD1	chr6:51923126	23.9	23.9	23.9
DST	chr6:56341107	41	11.7	23.21219512
DST	chr6:56347533	24.3	24.3	24.3
DST	chr6:56350158	21.5	21.5	21.5
DST	chr6:56417148	68.2	28.2	48.2
DST	chr6:56417448	71.5	71.5	71.5
DST	chr6:56485266	22.9	22.9	22.9
DST	chr6:56489467	45.8	45.8	45.8
BAI3	chr6:69349192	29	29	29
BAI3	chr6:70071243	34.8	34.8	34.8
EPHA7	chr6:93968004	97.3	83.3	88.79333333
EPHA7	chr6:93973530	27.8	27.8	27.8
EPHA7	chr6:94128960	29.3	29.3	29.3
PIK3CG	chr7:106545513	25.6	25.6	25.6
MET	chr7:116339867	26	26	26
MET	chr7:116397717	38.3	21.2	29.45454545
POT1	chr7:124475481	45.2	45.2	45.2
ETV1	chr7:13950941	29	29	29
EZH2	chr7:148523504	31.6	23.5	28.63333333
MLL3	chr7:151859655	23.9	23.9	23.9
MLL3	chr7:151902164	30.4	13.8	21.37
MLL3	chr7:151902167	85.5	15.6	47.15
MLL3	chr7:151947941	40.9	40.9	40.9
MLL3	chr7:151971024	14.8	14.8	14.8
CARD11	chr7:2968269	23	23	23
PMS2	chr7:6027040	42.2	42.2	42.2
PMS2	chr7:6029511	30.7	22.2	25.53333333
PMS2	chr7:6035156	35	35	35
AKAP9	chr7:91631234	52.1	52.1	52.1
AKAP9	chr7:91641671	10.2	10.2	10.2
AKAP9	chr7:91670064	51.4	51.4	51.4
AKAP9	chr7:91672096	28	28	28
AKAP9	chr7:91699372	43.8	43.8	43.8

AKAP9	chr7:91709156	25.2	25.2	25.2
AKAP9	chr7:91712728	56.8	56.8	56.8
TRRAP	chr7:98552756	21.9	21.9	21.9
TRRAP	chr7:98552760	28.2	28.2	28.2
TRRAP	chr7:98552779	27.3	27.3	27.3
TRRAP	chr7:98579357	26.5	26.5	26.5
UBR5	chr8:103284936	20.1	20.1	20.1
UBR5	chr8:103289351	22.9	22.9	22.9
UBR5	chr8:103297404	23	23	23
UBR5	chr8:103307466	51.5	51.5	51.5
UBR5	chr8:103354860	48.1	48.1	48.1
CSMD3	chr8:113254080	11.6	9.4	10.5
CSMD3	chr8:113301795	11.8	11.8	11.8
CSMD3	chr8:113323397	23.9	23.9	23.9
CSMD3	chr8:113516216	39.2	30.8	33.7
CSMD3	chr8:113568949	22.4	22.4	22.4
EXT1	chr8:118825146	59.6	59.6	59.6
EXT1	chr8:119122431	66	66	66
MYC	chr8:128753260	42.2	42.2	42.2
RECQL4	chr8:145737307	70.7	70.7	70.7
WRN	chr8:30938754	67.4	65	66.2
WRN	chr8:30989869	27.4	27.4	27.4
WRN	chr8:31004834	43.4	43.4	43.4
WRN	chr8:31024684	73.8	47.5	60.65
GPR124	chr8:37691418	41.4	41.4	41.4
FGFR1	chr8:38272357	41.4	16.6	27.38333333
KAT6A	chr8:41791041	97.4	97.4	97.4
IKBKB	chr8:42175297	28.1	28.1	28.1
HOOK3	chr8:42868456	9.1	9.1	9.1
PRKDC	chr8:48686929	24.8	24.8	24.8
PRKDC	chr8:48736499	40.9	40.9	40.9
PRKDC	chr8:48776046	23.5	23.5	23.5
PRKDC	chr8:48825021	20.2	20.2	20.2
PRKDC	chr8:48828031	44.7	32.6	38.65
NCOA2	chr8:71041018	25.8	25.8	25.8
NCOA2	chr8:71041202	40.8	40.8	40.8
NCOA2	chr8:71075661	56.2	56.2	56.2
NCOA2	chr8:71075779	93.2	93.2	93.2
RUNX1T1	chr8:92972668	97.2	97.2	97.2
RUNX1T1	chr8:93026955	53.4	53.4	53.4
RUNX1T1	chr8:93029480	35.5	35.5	35.5
RALGDS	chr9:135981338	27	27	27
CDKN2A	chr9:21994158	52	52	52
TAF1L	chr9:32631748	23.3	23.3	23.3
PAX5	chr9:37006542	21.2	21.2	21.2
JAK2	chr9:5081778	89.7	89.7	89.7
JAK2	chr9:5090443	31.8	31.8	31.8

PTPRD	chr9:8485904	25.2	25.2	25.2
PTPRD	chr9:8485984	26.3	26.3	26.3
SYK	chr9:93606313	26.5	26.5	26.5
PTCH1	chr9:98212211	32.3	32.3	32.3
PTCH1	chr9:98241253	32.5	32.5	32.5
USP9X	chrX:41088723	30.6	26.7	28.56666667
TFE3	chrX:48896874	33.5	33.5	33.5
KDM5C	chrX:53245059	24.5	19.2	21.85
FAM123B	chrX:63411640	10.9	10.9	10.9
TAF1	chrX:70597573	63.4	63.4	63.4
TAF1	chrX:70683793	44.9	44.9	44.9
ATRX	chrX:76849165	33.4	33.4	33.4
TBX22	chrX:79279609	48.2	48.2	48.2
LIFR	**chr5:38482324**	0	0	0
NOTCH4	**chr6:32164107**	0	0	0

Count of patients with this variant	Number of patient with VAF 100 or 50	A1	A2
39	24	-	CG
21	21	G	T
31	18	-	CCCCAGGC
87	15	T	C
18	15	-	C
14	13	C	-
13	13	-	A
11	11	-	G
87	10	A	C
10	10	-	T
26	9	T	C
9	8	T	-
11	8	G	T
11	7	G	C
11	6	-	T
9	5	T	G
13	4	-	CT
7	4	CA	AT
4	4	A	-
10	4	GCCTT	-
12	4	-	A
23	3	C	G
2	2	G	A
2	2	C	T
7	1	T	C
5	1	-	C
3	1	GAC	ACT
2	1	C	G
54	1	T	A
3	1	G	T
1	1	T	A
4	1	A	T
45	1	-	TA
1	1	C	A
25	1	CGC	-
1	1	C	T
2	1	G	C
3	1	T	-
47	1	T	G
1	1	G	T
1	1	C	T
1	1	G	C
7	1	-	C
1	1	C	-
1	1	GC	AA

1	1	G	C
1	1	C	T
1	1	T	A
1	1	T	-
1	1	G	T
1	1	T	-
1	1	C	T
5	1	-	A
1	1	C	T
1	1	G	T
3	1	-	T
1	1	C	T
30	1	T	A
2	1	A	C
1	1	-	GAAA
1	1	A	G
2	1	-	AAA
12	1	T	A
1	1	G	A
3	1	-	T
1	1	TGA	-
1	1	G	A
1	1	G	A
1	1	T	G
1	0	C	A
1	0	A	G
1	0	T	C
1	0	A	G
1	0	C	G
7	0	T	C
6	0	C	T
1	0	A	G
1	0	A	C
1	0	TT	GA
1	0	T	G
2	0	A	G
9	0	C	T
1	0	-	T
5	0	T	C
11	0	A	G
4	0	T	G
1	0	C	T
4	0	C	A
1	0	C	T
3	0	T	C
9	0	A	C
1	0	C	T

1	0	T	G
2	0	A	G
1	0	T	C
1	0	T	A
1	0	G	A
1	0	A	G
1	0	G	A
1	0	A	G
6	0	CG	GT
1	0	T	G
1	0	-	T
1	0	T	A
1	0	G	A
3	0	T	-
1	0	A	C
1	0	C	A
1	0	T	C
1	0	G	A
1	0	A	G
1	0	G	A
2	0	GC	TT
6	0	TT	GG
1	0	A	C
2	0	T	A
1	0	G	A
1	0	-	T
1	0	-	A
1	0	T	A
1	0	T	C
1	0	A	C
1	0	T	C
1	0	T	C
1	0	T	G
1	0	C	A
1	0	T	G
1	0	G	T
1	0	T	A
1	0	C	A
1	0	A	C
1	0	A	C
2	0	T	C
1	0	T	G
1	0	T	C
1	0	C	T
4	0	T	-
5	0	T	G
1	0	T	A

1	0	C	T
2	0	A	G
1	0	ATAT	-
1	0	G	A
2	0	A	C
1	0	A	T
21	0	-	T
1	0	C	T
1	0	-	A
1	0	T	A
3	0	G	A
1	0	G	A
1	0	C	A
1	0	T	G
1	0	C	T
2	0	A	C
1	0	C	A
1	0	A	G
2	0	TTA	ATT
1	0	G	T
1	0	C	T
1	0	T	G
1	0	A	G
3	0	-	TT
1	0	A	T
1	0	-	A
1	0	A	G
1	0	G	T
1	0	C	T
1	0	A	G
1	0	A	G
4	0	A	-
1	0	A	G
1	0	A	G
1	0	T	C
1	0	C	A
1	0	G	A
1	0	T	G
1	0	C	A
1	0	C	-
1	0	T	C
1	0	-	AGG
1	0	T	C
1	0	C	A
1	0	G	T
1	0	C	G
1	0	C	T

1	0	C	T
2	0	G	T
1	0	A	G
3	0	T	A
1	0	T	C
1	0	A	G
1	0	T	G
2	0	-	TTTT
1	0	T	C
1	0	A	G
1	0	A	C
2	0	T	A
1	0	A	C
1	0	C	A
1	0	T	G
1	0	G	A
1	0	A	G
1	0	G	T
1	0	T	C
1	0	C	G
1	0	A	C
1	0	A	G
8	0	TTG	-
1	0	G	T
2	0	G	T
1	0	A	G
1	0	C	G
1	0	T	C
1	0	C	T
1	0	G	C
1	0	A	C
1	0	T	G
2	0	C	T
1	0	A	C
2	0	A	T
1	0	T	G
1	0	A	T
1	0	A	G
17	0	T	C
1	0	T	A
5	0	G	T
1	0	T	A
5	0	T	C
6	0	T	A
1	0	C	T
1	0	A	G
1	0	A	C

14	0	G	A
2	0	C	A
1	0	T	A
1	0	C	T
1	0	G	T
1	0	C	G
1	0	T	G
1	0	C	A
9	0	A	G
1	0	A	G
1	0	T	C
2	0	CGAG	-
1	0	A	G
1	0	T	C
1	0	G	A
1	0	A	T
1	0	T	C
1	0	G	T
1	0	G	C
1	0	T	C
1	0	C	A
1	0	C	A
1	0	T	C
1	0	A	G
1	0	C	A
1	0	TA	GG
1	0	T	G
1	0	A	G
1	0	G	A
1	0	C	A
1	0	A	G
1	0	G	T
1	0	T	G
1	0	T	G
1	0	T	G
1	0	G	A
1	0	C	G
1	0	A	C
1	0	T	C
1	0	G	T
1	0	A	G
1	0	-	A
1	0	T	A
14	0	G	A
3	0	C	A
1	0	-	ACTTCT
1	0	G	A

7	0	-	A
7	0	G	A
1	0	-	GAGG
1	0	T	C
3	0	G	C
31	0	G	C
25	0	C	T
1	0	CC	GT
2	0	A	T
1	0	T	C
1	0	G	A
1	0	T	C
1	0	A	G
2	0	GG	AA
1	0	T	C
1	0	G	A
1	0	-	T
1	0	A	C
1	0	-	T
1	0	G	C
4	0	A	T
1	0	A	C
1	0	A	C
2	0	AA	CC
1	0	C	T
1	0	A	G
1	0	C	A
1	0	G	C
1	0	A	T
1	0	C	A
1	0	A	C
1	0	A	C
1	0	A	C
1	0	A	G
1	0	G	A
1	0	C	A
1	0	G	A
1	0	T	C
1	0	C	T
1	0	G	T
3	0	C	-
1	0	C	T
1	0	C	A
1	0	A	C
1	0	A	C
1	0	T	G
1	0	T	A

1	0	A	C
1	0	-	C
6	0	-	C
1	0	T	C
1	0	GG	AC
1	0	G	C
1	0	G	T
1	0	A	C
1	0	T	-
1	0	G	A
1	0	A	C
1	0	C	A
1	0	T	C
1	0	A	G
1	0	-	T
1	0	TA	GT
1	0	-	A
1	0	-	ACCACCTC[illegible]
1	0	A	C
1	0	G	A
1	0	-	AGA
1	0	A	C
1	0	A	G
2	0	C	T
1	0	G	C
8	0	C	T
1	0	G	T
1	0	T	A
1	0	A	G
1	0	C	G
1	0	C	A
1	0	T	C
1	0	G	T
1	0	T	G
1	0	T	G
1	0	G	A
1	0	G	T
1	0	T	C
1	0	A	G
1	0	T	G
1	0	T	G
2	0	C	T
1	0	C	G
1	0	A	G
1	0	A	C
1	0	A	C
1	0	A	C

1	0	C	A
1	0	C	T
1	0	A	C
1	0	T	A
11	0	T	A
1	0	G	A
2	0	T	C
1	0	A	G
1	0	T	C
1	0	T	C
1	0	T	A
1	0	T	G
1	0	A	T
1	0	A	C
1	0	T	C
41	0	A	T
1	0	C	A
1	0	C	A
2	0	G	T
1	0	C	A
1	0	C	G
1	0	-	TTACTC
1	0	A	G
1	0	G	A
15	0	C	T
1	0	G	T
1	0	C	T
1	0	G	A
1	0	G	T
11	0	G	C
1	0	G	T
1	0	T	G
3	0	A	T
1	0	A	G
10	0	T	A
12	0	-	TT
1	0	T	C
1	0	A	G
1	0	C	T
1	0	C	A
3	0	T	A
1	0	G	T
1	0	C	A
1	0	TGC	CAA
1	0	C	T
1	0	G	T
1	0	A	-

1	0	G	T
1	0	A	C
1	0	C	A
1	0	T	C
1	0	T	C
1	0	T	C
1	0	T	C
1	0	C	T
1	0	G	T
1	0	C	T
1	0	C	G
2	0	-	CACACACG
1	0	TTA	ATT
1	0	G	T
3	0	G	A
1	0	G	T
1	0	A	T
1	0	A	G
1	0	T	G
1	0	A	G
2	0	-	T
1	0	C	A
1	0	A	T
2	0	T	G
1	0	A	C
12	0	A	T
1	0	A	-
1	0	C	T
1	0	-	TTTTTT
1	0	C	T
1	0	G	C
1	0	G	A
1	0	C	G
2	0	C	A
1	0	A	C
1	0	C	T
1	0	G	A
1	0	C	G
1	0	C	G
1	0	A	G
1	0	A	C
1	0	T	C
1	0	C	T
1	0	A	G
1	0	A	C
1	0	TA	CT
1	0	G	A

1	0	T	A
1	0	T	C
1	0	A	C
1	0	T	G
1	0	C	T
3	0	T	A
1	0	T	C
2	0	T	C
1	0	ATAGAACT	-
1	0	C	G
1	0	T	C
1	0	T	C
1	0	C	G
0	0	T	G
0	0	A	G

MAF
3.33%
1.11%
1.67%
36.67%
6.67%
3.33%
8.80%
1.11%
42.22%
4.44%
4.44%
2.22%
4.44%
3.33%
3.33%
1.11%
2.22%
7.78%
2.22%
2.22%
1.11%
3.89%
2.22%
0.80%
2.22%
1.67%
1.11%
0.56%
1.11%
1.11%
0.56%
2.22%
3.33%
1.11%
0.56%
0.56%
1.11%
0.56%
1.11%
0.56%
0.56%
1.11%
0.56%
0.56%
0.56%

0.56%
0.56%
0.56%
1.11%
1.11%
0.56%
1.11%
1.11%
0.56%
0.56%
0.56%
1.11%
11.11%
0.56%
0.56%
0.56%
1.11%
1.11%
0.56%
1.11%
1.11%
0.56%
1.11%
0.00%
0.56%
0.56%
0.56%
0.56%
0.56%
2.78%
2.22%
0.56%
0.56%
0.56%
0.56%
1.11%
2.22%
0.56%
2.78%
5.00%
2.22%
0.56%
1.11%
0.56%
1.67%
3.33%
0.56%

0.56%
0.56%
0.56%
0.56%
0.56%
0.56%
0.56%
0.56%
1.11%
0.56%
0.56%
0.56%
0.56%
1.11%
0.56%
0.56%
0.56%
0.56%
0.56%
0.56%
1.11%
2.78%
0.56%
0.56%
0.56%
0.56%
0.56%
0.56%
0.56%
0.56%
0.56%
0.56%
1.11%
0.56%
0.56%
0.56%
0.56%
0.56%
0.56%
0.56%
0.56%
0.56%
0.56%
0.56%
1.67%
1.67%
0.56%

0.56%
1.11%
0.56%
0.56%
1.11%
0.56%
6.67%
0.56%
0.56%
0.56%
1.11%
0.56%
0.56%
0.56%
0.56%
1.11%
0.56%
0.56%
0.56%
0.56%
0.56%
0.56%
0.56%
0.56%
0.56%
0.56%
0.56%
0.56%
0.56%
0.56%
0.56%
0.56%
0.56%
0.56%
0.56%
0.56%
0.56%
0.56%
0.56%
1.11%
0.56%
0.56%
0.56%
0.56%
0.56%
0.56%
0.56%

0.56%
0.56%
0.56%
1.11%
0.56%
0.56%
0.56%
0.56%
0.56%
0.56%
0.56%
1.11%
0.56%
0.56%
0.56%
0.56%
0.56%
0.56%
0.56%
0.56%
0.56%
0.56%
1.11%
0.56%
0.56%
0.56%
0.56%
0.56%
0.56%
0.56%
0.56%
0.56%
0.56%
0.56%
0.56%
0.56%
0.56%
0.56%
2.78%
0.56%
0.56%
0.56%
0.56%
1.11%
0.56%
0.56%
0.56%

3.33%
1.11%
0.56%
0.56%
0.56%
0.56%
0.56%
0.56%
2.78%
0.56%
0.56%
1.11%
0.56%
0.56%
0.56%
0.56%
0.56%
0.56%
0.56%
0.56%
0.56%
0.56%
0.56%
0.56%
0.56%
0.56%
0.56%
0.56%
0.56%
0.56%
0.56%
0.56%
0.56%
0.56%
0.56%
0.56%
0.56%
0.56%
0.56%
0.56%
0.56%
0.56%
0.56%
0.56%
2.22%
0.56%
0.56%

1.11%
1.11%
0.56%
0.56%
1.11%
12.22%
3.33%
0.56%
0.56%
0.56%
0.56%
0.56%
0.56%
0.56%
0.56%
0.56%
0.56%
0.56%
0.56%
0.56%
1.11%
0.56%
0.56%
0.56%
0.56%
0.56%
0.56%
0.56%
0.56%
0.56%
0.56%
0.56%
0.56%
0.56%
0.56%
0.56%
0.56%
0.56%
0.56%
0.56%
0.56%
0.56%
0.56%
0.56%
0.56%
0.56%
0.56%

0.56%
0.56%
1.11%
0.56%
0.56%
0.56%
0.56%
0.56%
0.56%
0.56%
0.56%
0.56%
0.56%
0.56%
0.56%
0.56%
0.56%
0.56%
0.56%
0.56%
0.56%
0.56%
0.56%
1.11%
0.56%
1.67%
0.56%
0.56%
0.56%
0.56%
0.56%
0.56%
0.56%
0.56%
0.56%
0.56%
0.56%
0.56%
0.56%
0.56%
0.56%
0.56%
0.56%
0.56%
0.56%
0.56%
0.56%

0.56%
0.56%
0.56%
0.56%
1.11%
0.56%
0.56%
0.56%
0.56%
0.56%
0.56%
0.56%
0.56%
0.56%
0.56%
8.89%
0.56%
0.56%
0.56%
0.56%
0.56%
0.56%
0.56%
0.56%
5.56%
0.56%
0.56%
0.56%
0.56%
6.11%
0.56%
0.56%
1.67%
0.56%
0.56%
0.56%
0.56%
0.56%
0.56%
0.56%
1.11%
0.56%
0.56%
0.56%
0.56%
0.56%
0.56%

0.56%
0.56%
0.56%
0.56%
0.56%
0.56%
0.56%
0.56%
0.56%
0.56%
0.56%
1.11%
0.56%
0.56%
1.67%
0.56%
0.56%
0.56%
0.56%
0.56%
0.56%
0.56%
0.56%
0.56%
0.56%
4.44%
1.11%
0.56%
0.56%
0.56%
0.56%
0.56%
0.56%
0.56%
0.56%
0.56%
0.56%
1.11%
1.11%
0.56%
0.56%
0.56%
0.56%
0.56%
0.56%
1.11%
0.56%

0.56%
0.56%
0.56%
0.56%
0.56%
0.80%
0.80%
0.00%
0.80%
0.80%
0.00%
0.80%
0.80%
0.56%
0.56%
