# Supplementary material for: Identification of Novel Mutations in Colorectal Cancer Patients Using AmpliSeq Comprehensive Cancer Panel
Source: J Pers Med. 2021 Jun 9;11(6):535. doi: 10.3390/jpm11060535 (PMC8230213; doi:10.3390/jpm11060535)
Supplement: Supplementary file 1 [file jpm-11-00535-s001.zip › jpm-1202870-supplementary/supplementary tables/Supplementary Table S6.pdf]

Supplementary table S6

sn	CHR	SNP	Gene ID_CRC_P	A1	A2	EXON	INTRON
1309	5	chr5:112175216	APC	T	G	16/16	-
2910	12	chr12:46230604	ARID2	T	C	8/21	-
2831	11	chr11:108175462	ATM	A	G	37/63	-
2832	11	chr11:108175463	ATM	T	A	37/63	-
392	1	chr1:193111048	CDC73	T	G	7/17	-
3162	14	chr14:95595941	DICER1	T	C	8/29	-
2089	7	chr7:148525904	EZH2	G	C	6/20	-
4207	23	chrX:44969370	KDM6A	A	G	28/29	-
1112	4	chr4:55593464	KIT	C	A	10/21	-
1116	4	chr4:55599268	KIT	T	C	17/21	-
1106	4	chr4:55152040	PDGFRA	T	C	18/23	-
1774	6	chr6:152706918	SYNE1	A	G	55/146	-
3556	17	chr17:7577498	TP53	T	C	-	7/10
3557	17	chr17:7577538	TP53	T	C	7/11	-
3558	17	chr17:7577539	TP53	A	G	7/11	-
3560	17	chr17:7577556	TP53	T	C	7/11	-
3566	17	chr17:7578404	TP53	T	A	5/11	-
3567	17	chr17:7578406	TP53	T	C	5/11	-

cDNA_positio	CDS_positi	Protein_po	Amino_aci	Codons	Existing_va	large_intes	all_pathog	pathogenic
4305	3925	1309	E/*	Gaa/Taa	COSM1877	PATHOGEN	PATHOGEN	PATHOGEN
1025	853	285	R/W	Cgg/Tgg	COSM1890	PATHOGEN	PATHOGEN	PATHOGEN
5942	5557	1853	D/N	Gat/Aat	COSM4159	PATHOGEN	PATHOGEN	PATHOGEN
5943	5558	1853	D/V	gAt/gTt	COSM2162	PATHOGEN	PATHOGEN	PATHOGEN
765	581	194	R/I	aGa/aTa	,COSM901	PATHOGEN	PATHOGEN	PATHOGEN
894	602	201	R/H	cGc/cAc	COSM1238	PATHOGEN	PATHOGEN	PATHOGEN
675	553	185	D/H	Gac/Cac	COSM3762	PATHOGEN	PATHOGEN	PATHOGEN
4093	4052	1351	R/Q	cGa/cAa	rs3707654	PATHOGEN	PATHOGEN	PATHOGEN
1718	1621	541	M/L	Atg/Ctg	rs3822214,	PATHOGEN	PATHOGEN	PATHOGEN
2491	2394	798	I	atC/atT	rs5578961	PATHOGEN	PATHOGEN	PATHOGEN
2803	2472	824	V	gtC/gtT	rs2228230,	PATHOGEN	PATHOGEN	PATHOGEN
9145	8543	2848	A/V	gCg/gTg	rs3688323	PATHOGEN	PATHOGEN	PATHOGEN
-	-	-	-	-	TP53_g.13	PATHOGEN	PATHOGEN	PATHOGEN
933	743	248	R/Q	cGg/cAg	rs1154065	PATHOGEN	PATHOGEN	PATHOGEN
932	742	248	R/W	Cgg/Tgg	rs1219126	PATHOGEN	PATHOGEN	PATHOGEN
915	725	242	C/Y	tGc/tAc	rs1219126	PATHOGEN	PATHOGEN	PATHOGEN
716	526	176	C/S	Tgc/Agc	rs9674618	PATHOGEN	PATHOGEN	PATHOGEN
714	524	175	R/H	cGc/cAc	CM062017	PATHOGEN	PATHOGEN	PATHOGEN

large_intes	all_somatic	Somatic	other cancer type
Reported ir	Reported ir	none	Endometrioid carcinoma
Confirmed	Reported ir	Confirmed somatic variant	endometrium,pancreatic and stomach
Confirmed	Reported ir	Confirmed somatic variant	Prostate and breast
Confirmed	Reported ir	Confirmed somatic variant	prostate and lung
Confirmed	Reported ir	Confirmed somatic variant	endometrium
Confirmed	Reported ir	Confirmed somatic variant	Oesophagus
Confirmed	Reported ir	Confirmed somatic variant	Prostate
Confirmed	Reported ir	Confirmed somatic variant	Central nervous system
Reported ir	Reported ir	none	Lung, Breast and Bone
Confirmed	Reported ir	Confirmed somatic variant	Haematopoietic and lymphoid, soft tissue,Bor
Confirmed	Reported ir	Confirmed somatic variant	Haematopoietic and lymphoid, soft tissue,Bor
Confirmed	Reported ir	Confirmed somatic variant	Liver and Kidney
Reported ir	Reported ir	none	Breast, Lung , Stomach and Upperaerodigestiv
Confirmed	Reported ir	Confirmed somatic variant	Breast, Haematopoietic and lymphoid, Oesopl
Confirmed	Reported ir	Confirmed somatic variant	Prostate, biliary tract, Central Nervous Systen
Confirmed	Reported ir	Confirmed somatic variant	Breast, Pancreas, Lung and Upperaerodigestiv
Reported ir	Reported ir	none	Breast, Haematopoietic and lymphoid, Oesopl
Confirmed	Reported ir	Confirmed somatic variant	Breast, Stomachs and Central Nervous Systen

ıe and Testis
ıe and Testis

ve tract
hagus and Upperaerodigestive tract
ı and Upperaerodigestive tract
ve tract
hagus and Central Nervous System
ı
